# Comparative transcriptomic analysis of global gene expression mediated by (p) ppGpp reveals common regulatory networks in *Pseudomonas syringae*

**DOI:** 10.1186/s12864-020-6701-2

**Published:** 2020-04-10

**Authors:** Jun Liu, Menghao Yu, Tiyakhon Chatnaparat, Jae Hoon Lee, Yanli Tian, Baishi Hu, Youfu Zhao

**Affiliations:** 1 College of Plant Protection, Key Laboratory of Integrated Management of Crop Diseases and Pests, Nanjing Agricultural University, Nanjing, 210095 P. R. China; 2 Department of Crop Sciences, University of Illinois at Urbana-Champaign, 1201 W. Gregory Dr., Urbana, IL 61801 USA

**Keywords:** RNA-seq, Secondary messenger, Stringent response, Virulence factors, DC3000, B728a

## Abstract

**Background:**

*Pseudomonas syringae* is an important plant pathogen, which could adapt many different environmental conditions. Under the nutrient-limited and other stress conditions, *P. syringae* produces nucleotide signal molecules, i.e., guanosine tetra/pentaphosphate ((p)ppGpp), to globally regulate gene expression. Previous studies showed that (p) ppGpp played an important role in regulating virulence factors in *P. syringae* pv. *tomato* DC3000 (*Pst*DC3000) and *P. syringae* pv. *syringae* B728a (*Pss*B728a). Here we present a comparative transcriptomic analysis to uncover the overall effects of (p)ppGpp-mediated stringent response in *P. syringae*.

**Results:**

In this study, we investigated global gene expression profiles of *Pst*DC3000 and *Pss*B728a and their corresponding (p)ppGpp^0^ mutants in *hrp*-inducing minimal medium (HMM) using RNA-seq. A total of 1886 and 1562 differentially expressed genes (DEGs) were uncovered between the (p)ppGpp^0^ mutants and the wild-type in *Pst*DC3000 and *Pss*B728a, respectively. Comparative transcriptomics identified 1613 common DEGs, as well as 444 and 293 unique DEGs in *Pst*DC3000 and *Pss*B728a, respectively. Functional cluster analysis revealed that (p) ppGpp positively regulated a variety of virulence-associated genes, including type III secretion system (T3SS), type VI secretion system (T6SS), cell motility, cell division, and alginate biosynthesis, while negatively regulated multiple basic physiological processes, including DNA replication, RNA processes, nucleotide biosynthesis, fatty acid metabolism, ribosome protein biosynthesis, and amino acid metabolism in both *Pst*DC3000 and *Pss*B728a. Furthermore, (p) ppGpp had divergent effects on other processes in *Pst*DC3000 and *Pss*B728a, including phytotoxin, nitrogen regulation and general secretion pathway (GSP).

**Conclusion:**

In this study, comparative transcriptomic analysis reveals common regulatory networks in both *Pst*DC3000 and *Pss*B728a mediated by (p) ppGpp in HMM. In both *P. syringae* systems, (p) ppGpp re-allocate cellular resources by suppressing multiple basic physiological activities and enhancing virulence gene expression, suggesting a balance between growth, survival and virulence. Our research is important in that due to similar global gene expression mediated by (p) ppGpp in both *Pst*DC3000 and *Pss*B728a, it is reasonable to propose that (p) ppGpp could be used as a target to develop novel control measures to fight against important plant bacterial diseases.

## Background

*Pseudomonas syringae* is a widely-distributed gram negative plant pathogenic bacterium, which can adapt many different environmental conditions, and cause diseases on many different host plants, including bean, cabbage, cucumber, tomato, tobacco and rice [1, 2]. *P. syringae* can be classified into more than 50 pathovars (pv) based on host specificities or symptoms [3]. Among them, *P. syringae* pv. *tomato* (*Pst*) causes bacterial speck disease of tomato [4], and has served as a model system in plant-microbe interactions [5]. Whereas *P. syringae* pv. *syringae* (*Pss*) is the causal agent of brown spot on bean and an excellent epiphyte, which serves as a model system to study epiphytic fitness of pathogens on plant surfaces [6, 7]. *P. syringae* utilizes many virulence factors, including phytotoxins, exopolysaccharide [8], and the type III secretion system (T3SS) [9, 10]. The T3SS in *P. syringae* is transcriptionally regulated by a RNA polymerase sigma factor HrpL, which is activated by an alternative sigma factor RpoN, along with bacterial enhancer-binding proteins HrpS and HrpR [11, 12]. The T3SS genes are rapidly induced under limited nutrition (minimal medium), low pH and relatively low temperature; and are induced in planta or by iron, but inhibited in rich medium [13, 14].

Nucleotide second messengers are the major signal transduction molecules of bacteria, including c-di-GMP, c-di-AMP, cGMP, cAMP and (p) ppGpp [15]. These nucleotides control diverse cellular processes in response to environmental stresses for survival and virulence [15]. The guanosine tetraphosphate (ppGpp) and pentaphosphate (pppGpp), thereafter referred to as (p) ppGpp, are first discovered as bacterial ‘alarmone’ compounds produced under nutrient starvation [16]. When bacteria are under fatty acid, amino acid, phosphate, carbon or iron starvation, the RelA-SpoT homologue (RSH) proteins are activated to produce (p) ppGpp [16, 17], where bacteria re-allocate cellular resources by inhibiting DNA synthesis, RNA stability, ribosomal protein synthesis and membrane modules, and at the same time, by promoting key factors for stress resistance, glycolysis and amino acid biosynthesis. This process is referred to as the “stringent response” [16].

When amino acids are limited, uncharged tRNAs bind to the ribosomal A-site to activate the ribosome-associated protein RelA, which synthesizes (p) ppGpp [18]. In contrast, SpoT is a bifunctional enzyme that synthesizes and degrades (p)ppGpp. SpoT synthesizes (p) ppGpp in response to a lack of fatty acids, carbon, phosphorus, or iron, as well as hyperosmotic shock and oxidative stress [19, 20]. In *Escherichia coli*, (p) ppGpp regulates target genes through two mechanisms [21, 22]. On one hand, (p) ppGpp directly binds to the active sites of RNA polymerase (RNAP) to inhibit transcription involved in cell growth, cell division and to activate amino acid biosynthesis [23–25]. On the other hand, (p) ppGpp indirectly reduces the affinity of core RNAP and σ^70^, which leads to an increased availability of free RNAP. Alternative sigma factors in turn bind to RNAP to activate stress response genes, including oxidative and osmotic stress genes [16, 24].

Previous studies have shown that (p) ppGpp plays a central role in many processes related to survival and virulence [16, 17]. The (p)ppGpp^0^ mutants of *Pst*DC3000 and *Pss*B728a were both non-pathogenic and their growth in planta was significantly reduced [26, 27]. Furthermore, (p) ppGpp deficiency led to decreased expression of T3SS, loss of swarming motility, reduction of pyoverdine production, increased sensitivity to oxidative stress and antibiotic tolerance, as well as reduced ability to utilize γ-amino butyric acid [26, 27]. Moreover, cell sizes of the (p)ppGpp^0^ mutants were increased and their survival on plant surfaces was significantly decreased at 24 h after inoculation [26, 27], indicating that (p) ppGpp plays a major role in regulating gene expression for growth, survival and virulence. In this study, we performed a global transcriptomic analysis to compare gene expression between *Pst*DC3000 and its *relA*/*spoT*/*fpRel* triple mutant ((p)ppGpp^0^_*Pst*DC3000_), and between *Pss*B728a and its *relA*/*spoT* double mutant ((p)ppGpp^0^_*Pss*B728a_). The purpose was to determine the global effects of (p) ppGpp and to compare the two *P. syringae* systems to illustrate their similarities or differences in the global effects of the (p)ppGpp-mediated stringent response.

## Results and discussion

### Overview of gene expression profiles and transcriptomic analysis

In previous studies, we reported that (p) ppGpp production in *Pss*B728a depended on two enzymes, i.e., RelA and SpoT; whereas its production in *Pst*DC3000 relied on three enzymes, i.e., RelA, SpoT and fpRel [26, 27]. We also demonstrated that the stringent response mediated by (p) ppGpp plays a major role in virulence and survival in both PstDC3000 and PssB728a [26, 27]. In order to further understand its global effect, RNA-seq comparing the wild-type and the (p)ppGpp^0^ mutant was conducted in both *P. syringae* systems. In total, 11,261,275 to 23,836,829 reads for each biological sample were generated for *Pst*DC3000 and its triple mutant ((p)ppGpp^0^_*Pst*DC3000_), and the percentage of reads mapped to *Pst*DC3000 genome ranged from 96.4 to 97.5%; whereas 13,562,836 to 22,961,086 reads for each biological sample were obtained for *Pss*B728a and its double mutants ((p)ppGpp^0^_*Pss*B728a_), and the percentage of reads mapped to *Pss*B728a genome were from 96.8 to 97.3%.

**Fig. 1:**
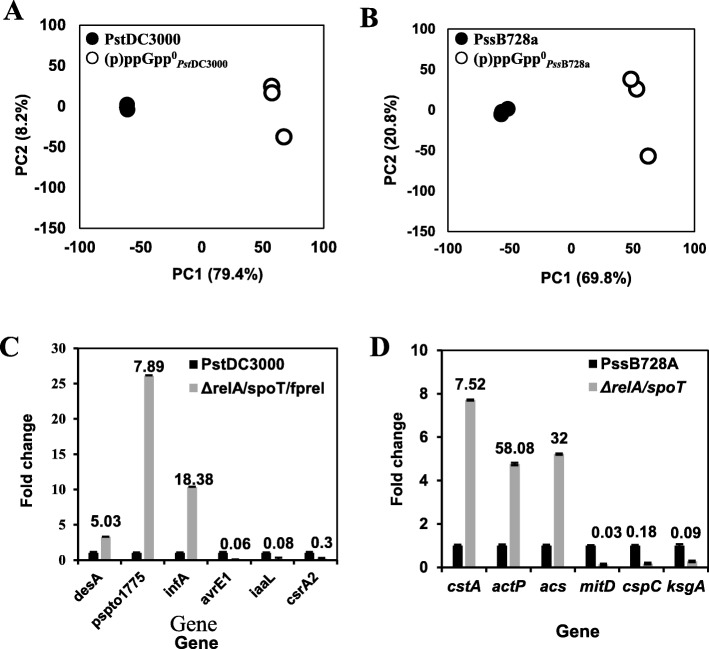
Principal component analysis (PCA) and verification of differential expressed genes (DEGs) by Quantitative real-time PCR. **a** PC1 and PC2 explain data variability of 79.4 and 8.2%, respectively, for *Pst*DC3000 and its (p)ppGpp^0^ mutant. **b** PC1 and PC2 explain data variability of 69.8 and 20.8%, respectively, for *Pss*B728a and its (p)ppGpp^0^ mutant. **c** Expression of the *desA*, *pspto_1775*, *infA*, *avrE1*, *iaaL* and *csrA2* genes in the *relA*/*spoT*/*fpRel* ((p)ppGpp^0^) mutant strain as compared with *Pst*DC3000 grown in *hrp-*inducing medium at 3 h post-inoculation. **d** Expression of the *cstA*, *actP*, *acsA*, *mtiD*, *cspC* and *ksgA* genes in the *relA*/*spoT* ((p)ppGpp^0^) mutant strain as compared with *Pss*B728a grown in *hrp*-inducing medium at 3 h post-inoculation. Numbers on the bar indicate fold change by RNA-seq

To explore the similarities and differences between these samples, principal component analysis (PCA) was conducted within the two *P. syringae* systems. PCA plot clearly showed that the first two components (PC1 and PC2) explained about 91 and 87% of the variability in the datasets for *Pst*DC3000 and *Pss*B728a and their corresponding mutant strains, respectively (Fig. 1a & b). In both cases, the three biological samples of each strain clustered together, suggesting that variation mainly came from the difference between the wild-type and the (p)ppGpp^0^ mutant. Heatmap also showed that the three biological samples were very consistent (Additional file 1: Figure S1).

A total of 1886 and 1562 differentially expressed genes (DEGs), which displayed a |log_2_FC| value ≥1 and a corrected *p* value < 0.05 between *Pst*DC3000 and *Pss*B728a and its corresponding (p)ppGpp^0^ mutant, were respectively identified. (Additional file 1: Figure S1), representing about one third of genes in both *P. syringae* genomes. Among them, 945 genes were up-regulated and 941 genes were down-regulated in the (p)ppGpp^0^_*Pst*DC3000_ mutant, whereas 701 genes were up-regulated and 861 genes were down-regulated in the (p)ppGpp^0^_*Pss*B728a_ mutant (Additional file 1: Figure S2). To verify the RNA-seq data, 12 genes were selected, including genes encoding indoleacetate-lysine ligase (IaaL), fatty acid desaturase (DesA), ATP-dependent RNA helicase, DEAD box family (Pspto_1775), carbon storage regulator (CsrA2), type III effector protein (AvrE1) and translation initiation factor IF-1 (InfA) from *Pst*DC3000. Genes encoding mannitol dehydrogenase (MtiD), acetate permease (ActP), acetyl-CoA synthetase (AcsA), carbon starvation protein (CstA), cold-shock protein (CspC) and dimethyladenosine transferase (KsgA) were selected for *Pss*B728a. The qRT-PCR results showed that expression of selected genes was mostly in similar trend as those of the RNA-seq data (Fig. 1c & d). In addition, expression of T3SS and toxin biosynthesis genes was previously verified [26, 27].

**Fig. 2:**
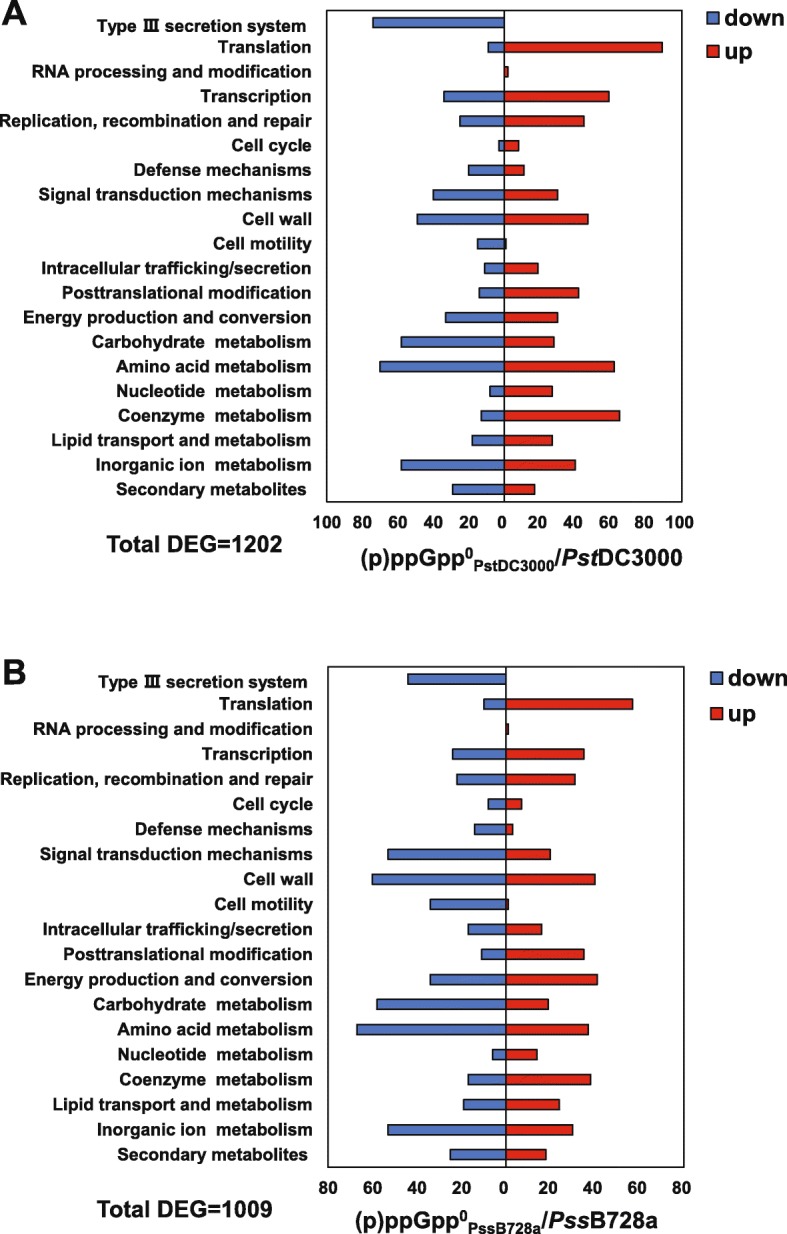
Functional classification of differential expressed genes (DEGs) based on the clusters of orthologous groups (COGs). **a** *Pst*DC3000 and (p)ppGpp^0^_*Pst*DC3000_. **b** *Pss*B728a and (p)ppGpp^0^_*Pss*B728a_. DEGs were defined as genes with a |log_2_FC| value ≥1 and a p value < 0.05. Up and down regulated genes were indicated by red and blue bars, respectively

DEGs were then functionally categorized based on the clusters of orthologous groups (COGs) (Additional file 2: Table S1 & S2). In total, 1202 DEGs for *Pst*DC3000 and 1009 DEGs for *Pss*B728a were functionally separated into 20 out of 21 known function categories (Fig. 2a & b), supporting the notion that (p) ppGpp is a global regulator. In general, most of the DEGs categorized as T3SS, signal transduction, cell motility, and carbohydrate metabolism were positively regulated by (p)ppGpp. In contrast, most DEGs belonging to translation, transcription, replication/recombination/repair, posttranslational modification, coenzyme metabolism, and nucleotide metabolism were negatively regulated by (p) ppGpp (Fig. 2a & b).

### Comparative transcriptomic analysis reveals common regulatory networks in two pathovars of *Pseudomonas syringae*

**Fig. 3:**
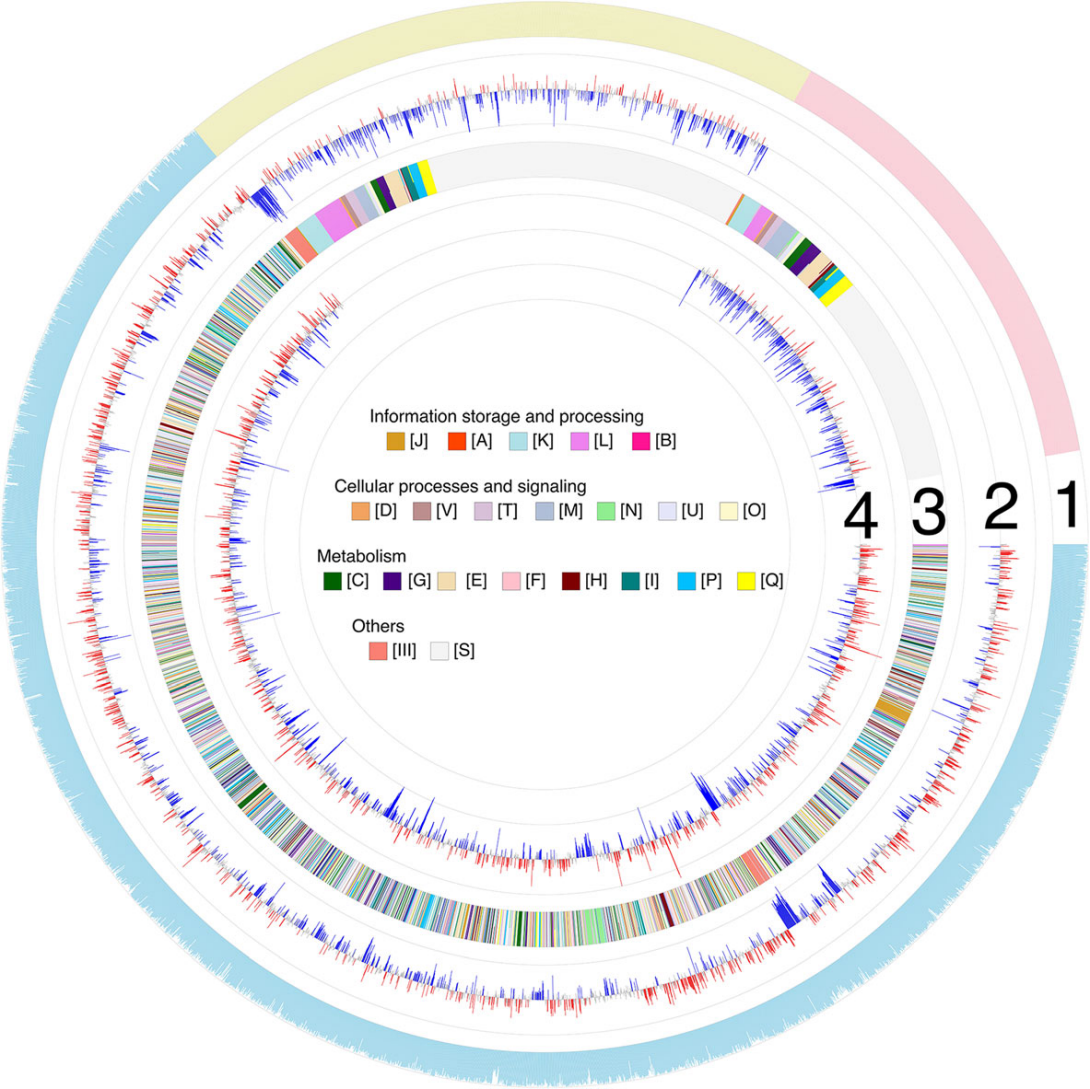
Circular representation of RNA-seq data. The first ring (outside ring) showed *Pst*DC3000 and *Pss*B728a genome. The sky blue region represents homologues genes between *Pst*DC3000 and *Pss*B728a; the height of the plot represents the percentage of sequence identity (40 to 100%). The yellow region represents unique genes of *Pst*DC3000. The pink region represents unique genes of *Pss*B728a. The second ring represents log_2_ FC of (p)ppGpp^0^_*Pst*DC3000_ versus *Pst*DC3000, red and blue bar represent log_2_FC ≥ 1 and log_2_FC ≤ -1 with a corrected *p* value < 0.05, respectively. The third ring shows that the clusters of orthologous groups (COG) of each genes. Difference function classification is represented by 22 colors. The fourth ring represents log_2_ FC of the (p)ppGpp^0^_*Pss*B728a_ versus *Pss*B728a, red and blue bar represents log_2_FC ≥ 1 and log_2_FC ≤ -1 with a corrected p value < 0.05, respectively

In order to determine the similarities and differences between effects of (p) ppGpp in *Pst*DC3000 and *Pss*B728a, a comparative transcriptomic analysis was performed. In the genomes of *Pst*DC3000 and *Pss*B728a, there are 5483 and 5137 protein-coding genes, respectively [6]. Among them, 4210 homologous genes with more than 40% sequence identity (E < 10^− 2^) are shared (Fig. 3, outside ring 1, sky blue; Additional file 3: Table S3), representing about three fourths of the genomes. The remaining 1273 and 927 genes are unique for *Pst*DC3000 and *Pss*B728a, respectively (Fig. 3, outside ring 1, yellow and pink; Additional file 3: Table S3). When DEGs of the (p)ppGpp^0^_*Pst*DC3000_ mutant versus *Pst*DC3000 (Fig. 3, Ring 2) and the (p)ppGpp^0^_*Pss*B728a_ mutant versus *Pst*B728a (Fig. 3, Ring 4) were mapped to the corresponding genes in the genome, similar expression patterns for homologous genes in *Pst*DC3000 and *Pss*B728a were observed. Interestingly, unique genes belonging to the same COGs in the two *P. syringae* systems were also shown similar expression profiles (Fig. 3, Ring 3). These results suggested that (p) ppGpp exhibited similar effects on global gene expression in both *Pst*DC3000 and *Pss*B728a in HMM. However, one caveat of this research was that we only determined gene expression at one time point.

**Fig. 4:**
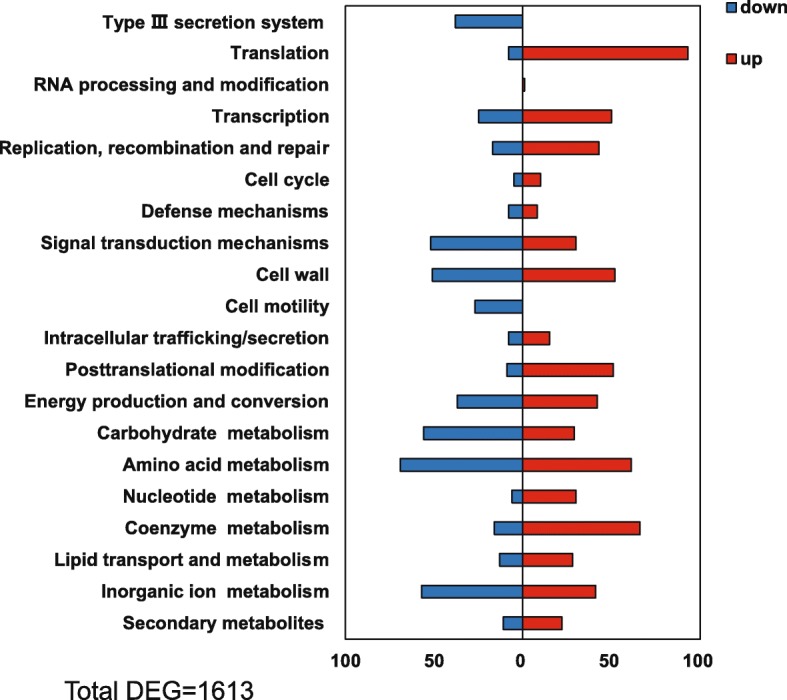
Functional classification of homologues DEGs regulated by (p) ppGpp in both *Pst*DC3000 and *Pss*B728a based on the clusters of orthologous groups (COGs). DEGs were defined as genes with a |log_2_FC| value ≥1 and a p value < 0.05. Up and down regulated genes were indicated by red and blue bars, respectively

A total of 1613 homologous genes were simultaneously differentially expressed (with adjusted *p*-values < 0.05 and at least one |log_2_FC| value ≥1) in comparison of the (p)ppGpp^0^_*Pst*DC3000_ mutant versus *Pst*DC3000 and the (p)ppGpp^0^_*Pss*B728a_ mutant versus *Pst*B728a (Additional file 3: Table S4). These genes were further functionally categorized based on COGs, thus reflecting the common global effects of (p) ppGpp in *P. syringae* (Fig. 4). Furthermore, transcriptomic analysis identified 255 homologues DEGs inversely regulated by (p) ppGpp (Additional file 3: Table S5). A total of 444 and 293 unique DEGs, which were regulated by (p) ppGpp in *Pst*DC3000 and *Pss*B728a, respectively, were also identified (Additional file 4: Table S6 & S7).

### Positive regulation of virulence-associated genes in *P. syringae*

#### Type III and type VI secretion system (T3SS and T6SS)

**Fig. 5:**
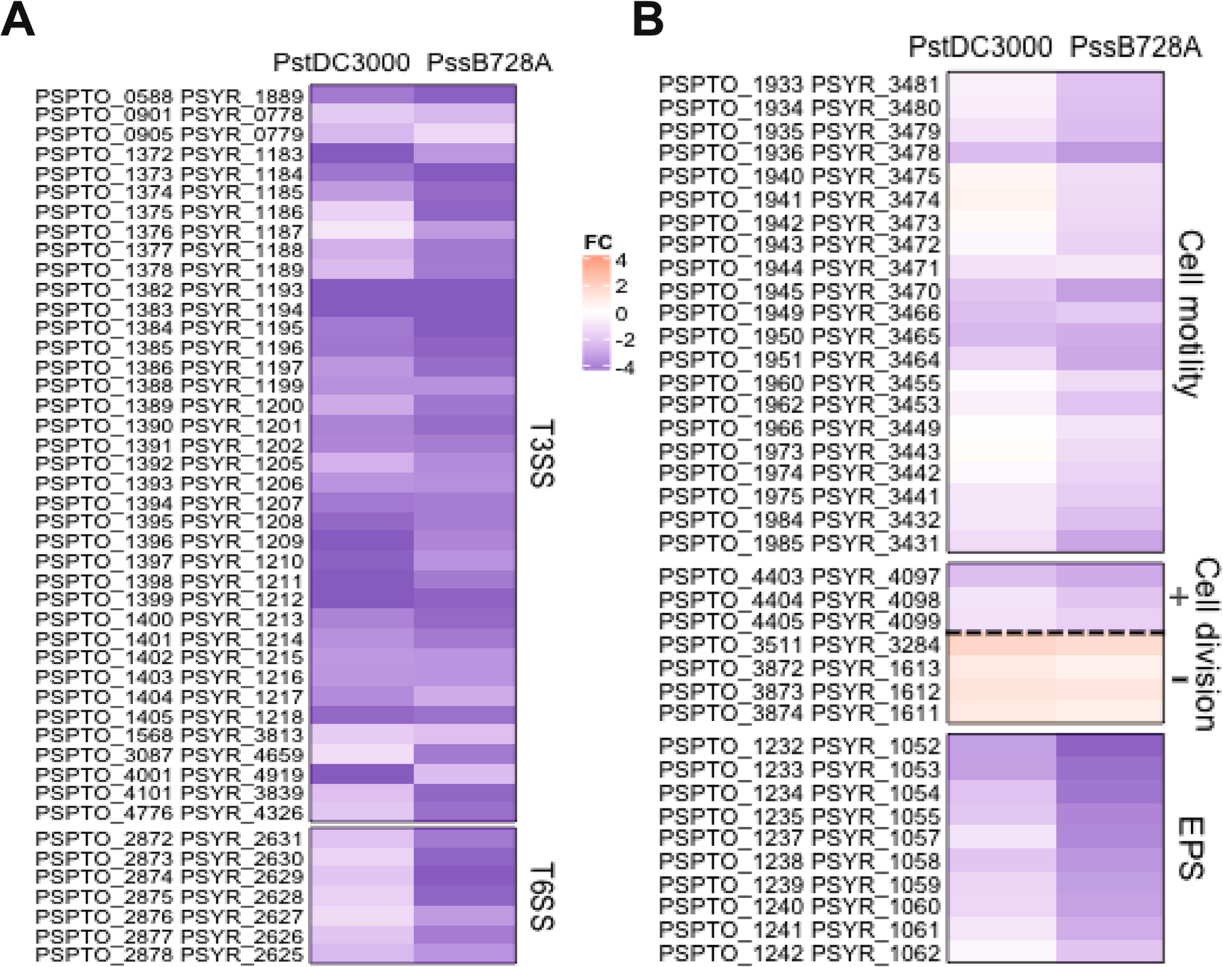
(p) ppGpp positively regulates expression of virulence factors in *Pseudomonas syringae.* **a** Heatmap showing expression for T3SS and T6SS genes. **b** Heatmap showing expression for cell motility, cell division and EPS genes

Bacteria rely on protein secretion systems to acquire essential nutrients and suppress or evade the host immune system during infection of the host plants [28]. Among the six different secretion systems [28–30], T3SS specifically secretes and translocates effector proteins from the bacteria to the cytoplasm of the host cell through the needle-like apparatus [31, 32]. T3SS is a key pathogenicity factor in both *Pst*DC3000 and *Pss*B728a and injects effector proteins into host cells to suppress host defense and cause disease [7, 33, 34]. In the regulatory networks of *P. syringae*, a sigma factor cascade (RpoN-HrpL) quickly activates T3SS under nutrient-limited conditions [11, 12, 35]. Previous studies have reported that accumulation of (p) ppGpp leads to increased transcription of genes regulated by alternative sigma factors, such as RpoN, which interacts with bacterial enhancer-binding proteins HrpS and HrpR [12, 35]. Chatnaparat et al. reported that the *hrpL*, *hrpR*, *hrpS* and *hrpZ* genes were significantly down-regulated in the (p)ppGpp^0^_*Pst*DC3000_ and (p)ppGpp^0^_*Pss*B728a_ mutants, indicating that (p) ppGpp is required for activating T3SS in both *Pst*DC3000 and *Pss*B728a. In this study, 38 T3SS-related genes, including *hrpL*, *hrp-hrc* gene clusters and many effector genes, were down-regulated by more than twofold in the (p)ppGpp^0^_*Pst*DC3000_ and (p)ppGpp^0^_*Pss*B728a_ mutants (Fig. 5a; Additional file 5: Table S8).

T6SS is implicated in bacterial interaction, competition and fitness [30, 36, 37]. Putative T6SS clusters have been reported previously in *P. syringae* pathovars [30]. One of the three T6SSs in *Pseudomonas aeruginosa* and T3SS were inversely regulated by the *retS* gene [38]. However, (p) ppGpp in *P. syringae* regulate transcription expression of T3SS and T6SS in a similar way. Several other genes such as quorum sensing as well as the RpoN also control T6SS in various bacteria [39]. In this study, 28 putative T6SS-associated genes of *Pst*DC3000 and 25 T6SS-related genes of *Pss*B728a were down-regulated in the (p)ppGpp^0^_*Pst*DC3000_ and (p)ppGpp^0^_*Pss*B728a_ mutants, respectively (Fig. 5a; Additional file 5: Table S8). These results confirm that (p) ppGpp influences virulence via positive regulation of both T3SS and T6SS in *P. syringae*.

#### Cell motility, cell division and exopolysaccharides (EPSs)

Motility might enable bacterial cells to avoid environmental stresses [40, 41]. Transcriptomic data showed that 27 genes involved in cell motility were down-regulated in *Pst*DC3000 and *Pss*B728a, including type IV pilus biogenesis (*pil*), flagellar (*flg*) and chemotaxis (*che*) genes (Additional file 3: Table S4). Flagella-mediated motility is an important factor in affecting the virulence of *P. syringae* [42]. The (p) ppGpp positively regulated expression of 16 flagellar-related genes (Additional file 5: Table S9), which is consistent with previous reports that motility of the (p)ppGpp^0^_*Pst*DC3000_ and (p)ppGpp^0^_*Pss*B728a_ mutants was suppressed [26, 27].

Previous studies have reported that cells of the (p)ppGpp^0^_*Pst*DC3000_ and (p)ppGpp^0^_*Pss*B728a_ mutants was much longer than those of *Pst*DC3000 and *Pss*B728a in HMM medium and on plant surfaces [26, 27]. Bacterial cells protect themselves by adjusting their cell sizes to adapt to a variety of environment conditions [16, 43]. The products of the *ftsA*, *ftsQ* and *ftsZ* genes are responsible for controlling cell division and cell wall metabolism [44]. A ring-like macro-molecular complex called the Z ring is formed by FtsZ polymers, in conjunction with FtsA, ZapA, ZapB and other factors [45]. Bacterial cell size is primarily controlled by the assembly and maturation of FtsZ ring [46]. Mutations in the formation and division of FtsZ ring increase average cell size [47–49]. On the other hand, cell division inhibitor (SulA) prevents cell division by directly interacting with FtsZ [50, 51], and MinCDE inhibits FtsZ ring assembly, thus blocking the FtsZ ring formation [51, 52]. In this study, RNA-seq data showed that the *ftsAQZ* cell division-related genes were down-regulated by more than two-fold, whereas the cell division inhibitor gene *sulA* (*pspto_3511*, *psyr_3284*) and *minCDE* were up-regulated in the (p)ppGpp^0^_*Pst*DC3000_ and (p)ppGpp^0^_*Pss*B728a_ mutants (Additional file 5: Table S9). These results might explain why cell sizes of the (p)ppGpp^0^_*Pst*DC3000_ and (p)ppGpp^0^_*Pss*B728a_ mutants were increased under such stress conditions.

In addition, EPSs play an important role in both the survival and virulence of *P. syrinage* under stress conditions [53]. *P. syringae* produces EPS molecules to avoid cell recognition of host plant, resist to desiccation, and enhance its epiphytic fitness [54, 55]. Alginate is one of the EPS molecules reported to be a virulence factor in *P. syringae* and *P. aeruginosa* [56–58]. In this study, RNA-seq data showed that 10 genes for alginate biosynthesis, including *algAEFGIJK*-*alg44*-*alg8* were down-regulated in both the (p)ppGpp^0^_*Pst*DC3000_ and (p)ppGpp^0^_*Pss*B728a_ mutants (Additional file 5: Table S9), indicating that (p) ppGpp activates gene expression of alginate biosynthesis. Heatmap analysis displayed the relative expression profile of genes involved in flagellar, cell division and alginate biosynthesis for (p)ppGpp^0^_*Pst*DC3000_ versus *Pst*DC3000 and (p)ppGpp^0^_*Pss*B728a_ versus *Pss*B728a (Fig. 5b). These results suggest that (p) ppGpp acts as an important internal signal to positively regulate virulence-associated factors in both *Pst*DC3000 and *Pss*B728a.

### Negative regulation of basic physiological processes in *P. syringae*

#### DNA replication, RNA processes and ribosomal protein biosynthesis

**Fig. 6:**
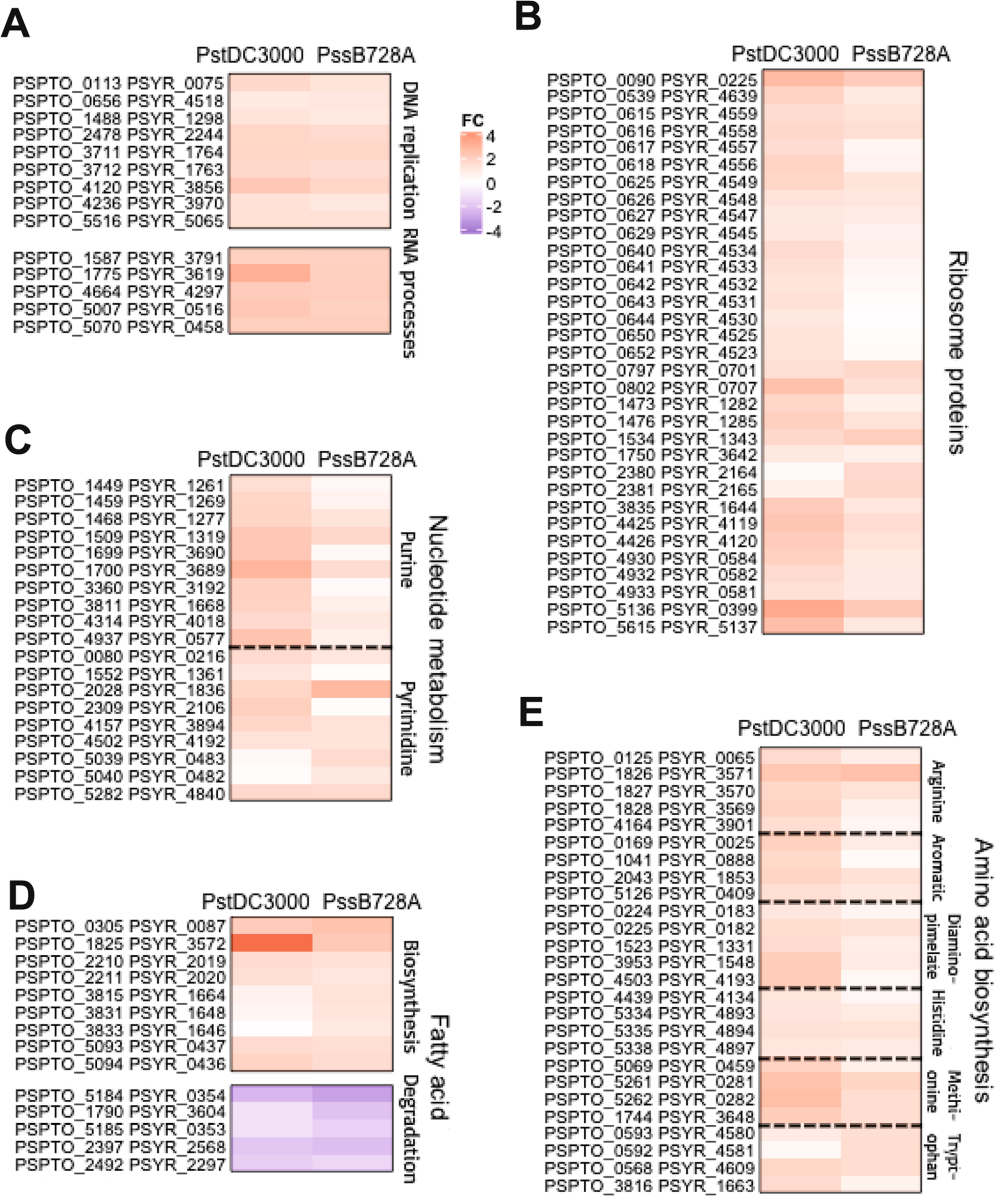
(p) ppGpp negatively regulates expression of DNA replication, RNA processes, ribosome protein biosynthesis, amino acid, fatty acid, and nucleotide metabolism-relate genes in *Pseudomonas syringae*. **a** Heatmap showing expression for DNA replication and RNA processes relate genes. **b** Heatmap showing expression for ribosome protein biosynthesis genes. **c** Heatmap showing expression for nucleotide metabolism genes. **d** Heatmap showing expression for fatty acid metabolism genes. **e** Heatmap showing expression for amino acid biosynthesis genes

As a global regulator, (p) ppGpp of *P. syringae* affects not only virulence factors, but also a variety of basic physiological processes, e.g., DNA replication, RNA processes, ribosome protein synthesis, nucleotide, fatty acid, and amino acid metabolism. In the transcriptomic profiles, only a few genes categorized as replication, recombination and repair were down-regulated in both the (p)ppGpp^0^_*Pst*DC3000_ and (p)ppGpp^0^_*Pss*B728a_ mutants; whereas most of these genes were up-regulated (Fig. 4), including *dinG*, *dnaQ*, *rnhA*, *rep*, *uvrD, recJ*, *ssB*, *ung*, and *tob*B (Fig. 6a; Additional file 5: Table S10). The *dinG* gene is a DNA damage-inducible gene that will increase significantly after exposure to DNA damage factors [59, 60]. In addition, in *E. coli*, DNA helicases, DinG, Rep and UvrD work together to promote replication [61]. The single-stranded DNA-binding proteins (SSB) interacts directly with a number of proteins involved in DNA metabolism, such as DinG [60], uracyl-DNA glycosylase [62], nucleases RecJ and SbcB, DNA topoisomerase III TopB, and helicase RecG and RecQ [63]. Furthermore, DNA polymerase III subunit epsilon DnaQ [64], ribonuclease RnhA [65], and TobB [66] are essential for DNA replication and DNA repair. It is possible that DNA damage might occur in cells due to the absence of (p) ppGpp in *P. syringae*, thus promoting expression of DNA repair and replication-related genes.

The DEAD box proteins mediate virtually all phases of RNA metabolism, such as RNA processing, transport, degradation, translation and ribosome biogenesis [67, 68]. The *deaD-dbpA-rhlE-rhlB-srmB* gene cluster, which encodes an ATP-dependent RNA helicase, is involved in important cellular processes such as mRNA decay or ribosome biogenesis [69]. Among them, SrmB [70] and CsdA (DeaD) [67] are involved in ribosome biogenesis. In this study, we found that gene clusters of *pspto_1775-dbpA-rhlE* (*pspto_4664*)*-rhlE* (*pspto_5070*)*-srmB* in *Pst*DC3000 and *psyr_3619-dbpA-rhlE-srmB-psyr_4297* in *Pss*B728a were up-regulated in the (p)ppGpp^0^_*Pst*DC3000_ and (p)ppGpp^0^_*Pss*B728a_ mutants, respectively (Fig. 6a; Additional file 5: Table S10).

Ribosomes provide the basis for carrying out protein synthesis, thus cell growth [71]. However, protein biosynthesis is the largest consumer of energy during cell proliferation [72]. Therefore, overproduction of ribosomal proteins not only will not benefit the cell, but also could be harmful [73]. Our results showed that 93 translation factors were up-regulated in the (p)ppGpp^0^_*Pst*DC3000_ and (p)ppGpp^0^_*Pss*B728a_ mutants, including 30S and 50S ribosome protein synthesis genes (*rps*, *rpm* and *rpl* gene clusters) (Additional file 5: Table S10). Heatmap analysis displayed the expression profile of 33 genes encoding ribosomal proteins (Fig. 6b), indicating that (p) ppGpp negatively regulated ribosomal protein synthesis in HMM.

#### Nucleotide, fatty acid and amino acid metabolism

Purine and pyrimidine nucleotides are the basis of cellular activities involved in almost all physiological processes [74]. The purine nucleotide synthesis uses phosphoribosyl pyrophosphate (PRPP) as the initial substrate via both the de novo and salvage biosynthetic pathways [75]. The process of de novo synthesis is divided into two stages, i.e., inosine monophosphate (IMP) synthesis by products of the *purBCDEFHKLMN* genes, and IMP converted into GMP and AMP by products of the *guaAB* and *purAB* genes, respectively [75, 76]. The adenylate kinase (Adk) catalyzes the conversion of AMP and ATP to two molecules of ADP in the purine nucleotide synthesis pathway [77, 78]. The first step in the pyrimidine biosynthesis pathway is the formation of carbamyl phosphate (CP) by *carA*, followed by the formation of UMP via the *pyrBCDEF* gene cluster [79]. The final product is UMP, which is formed by six steps using carbon dioxide, ammonia, ATP, aspartic acid, PRPP and phosphate [79]. In addition, thymidylate synthase (ThyA) involves a key step in the de novo synthesis of thymidine triphosphate (dTTP) that converts dUMP to dTMP [80]. In this study, RNA-seq data showed that 30 genes of nucleotide metabolism were up-regulated in the (p)ppGpp^0^_*Pst*DC3000_ and (p)ppGpp^0^_*Pss*B728a_ mutants, including *purABFLMNTU-3*, *guaB* and *adk* genes for purine biosynthesis, and the *pyr* gene cluster, the *tyrA* and *carA* genes for pyrimidine biosynthesis (Additional file 5: Table S11). Heatmap displayed expression profiles of genes involved in purine and pyrimidine biosynthesis in both *P. syringae* systems (Fig. 6c), indicating that (p) ppGpp inhibited nucleotide metabolism, probably influencing the redistribution of cell resources in *P. syringae.*

Fatty acid plays an important role in maintaining normal membrane structure and functions under various growth conditions [81]. In this study, our results showed that 13 lipid transport and metabolism-related genes were down-regulated in both the (p)ppGpp^0^_*Pst*DC3000_ and (p)ppGpp^0^_*Pss*B728a_ mutants (Additional file 5: Table S11), including genes encoding acyl-CoA dehydrogenase family protein and short chain dehydrogenase protein involved in the degradation of branched-chain fatty acid [82]. Furthermore, (p) ppGpp negatively regulated 28 fatty acid biosynthesis-related genes, such as *acsA*, *desA*, *acpP*, *accD*, and *fabABD* (Additional file 5: Table S11). Overexpression of the acetyl-CoA carboxylase (*acc*) and acetyl-CoA synthetase (*acsA*) genes could lead to increased fatty acid biosynthesis [83, 84]. A three-carbon precursor malonyl-CoA is required for fatty acid biosynthesis in bacteria, while malonyl-CoA is transferred to the acyl carrier protein (ACP) by malonyl-CoA:ACP acyltransferase (FabD) [85, 86]. In addition, bacteria control the production of unsaturated fatty acids (UFA) or branched-chain fatty acids (BFA) [81] to maintain correct physical state of the membrane lipids [87]. UFAs, as signaling molecules, are involved in important cellular processes, such as cell differentiation and DNA replication [88, 89]. The *fabA* and *fabB* genes are require to produce UFAs in *E. coli* and *P. aeruginosa* [81], whereas two desaturases DesA and DesB in *P. aeruginosa* supplement the anaerobic mechanism for UFA synthesis [81]. In *P. syringae*, (p) ppGpp suppressed fatty acid metabolism by decreasing expression of biosynthesis-related genes, but increasing expression of degradation-related genes (Fig. 6d).

Previous reports showed that the (p)ppGpp^0^ mutants are auxotrophic for 11 amino acids, indicating that (p) ppGpp is required for transcription of amino acid biosynthetic genes [20, 90]. In *E. coli*, (p) ppGpp directly binds to RNAP to activate transcription of amino acid biosynthesis genes [16, 17]. In this study, 61 up-regulated genes were involved in amino acid biosynthesis, including arginine (*arg*), aromatic (*aro*), diaminopimelate (*dap*), histidine (*his*), methionine (*met*), tryptophan (*trp*), whereas 69 down-regulated genes were mainly amino acid ABC transporters in both the (p)ppGpp^0^_*Pst*DC3000_ and (p)ppGpp^0^_*Pss*B728a_ mutants (Fig. 6e, Additional file 5: Table S11). The inconsistency between our results and previously reported results might be due to that these amino acid biosynthesis genes may be indirectly regulated by (p) ppGpp in *P. syringae* or due to the experimental conditions used. We also found similar results in *E. amylovora* (Yang et al. BMC Genom.). Taken together, our results suggested that (p) ppGpp played an important role by inhibiting multiple basic physiological processes, including DNA replication, RNA processes, ribosomal protein biosynthesis, and metabolisms of nucleotide, fatty acid and amino acid, to re-allocate cellular resources for virulence-associated gene expression and survival.

### Regulation of other cellular processes in *P. syringae*

#### Coenzyme and carbon metabolism

Coenzyme is an organic non-protein compound that binds with an enzyme to initiate or aid its function [91]. Our results showed that (p) ppGpp suppressed 66 coenzyme metabolism-related genes in both *Pst*DC3000 and *Pss*B728a, including the *ribDEF* gene cluster, which is involved in riboflavin biosynthesis [92–94], *nadACD* gene cluster (regulate niacin biosynthesis) and the *hemACFHL* gene cluster (Additional file 5: Table S12). The latter regulates heme biosynthesis pathway [95], which are essential for the function of diverse proteins, including cytochromes, globins, peroxidases, catalases, and sensors [96]. In contrast, 16 genes involved in coenzyme metabolism were positively controlled by (p) ppGpp, including *ssuD* (encoding alkanesulfonate monooxygenase), *phaG-1* (encoding 3-hydroxyacyl-CoA-ACP transferase) and *chiI* (encoding magnesium chelatase) (Additional file 5: Table S12).

Furthermore, RNA-seq results revealed that (p) ppGpp in *Pst*DC3000 and *Pss*B728a positively regulated 56 genes involved in carbohydrate metabolism, such as mannitol (*mtl*) [97], xylose (*xyl*) [98] and glycogen (*glg*) [99] (Additional file 5: Table S12). On the other hand, 29 carbohydrate metabolism-related genes in *Pst*DC3000 and *Pss*B728a were negatively controlled by (p) ppGpp, including *rpiA* (encoding ribose 5-phosphate isomerase), *mqo* (encoding malate:quinone oxidoreductase), and *tpiA* (encoding triosephosphate isomerase) (Additional file 5: Table S12).

#### Transcriptional regulators and signal transduction

Bacterial cells regulate the expression of a series of genes involved in transporter, signal transduction, and transcription in response to changes in the extracellular environment [100]. In this study, (p) ppGpp negatively regulated 50 transcription factors and 30 signal transduction-related genes. These genes encode carbon starvation protein CstA, BolA-like protein, phosphohistidine phosphatase SixA, transcriptional activator MetR, alkylphosphonate utilization operon protein PhnA, and transcriptional regulator NrdR (Additional file 5: Table S13). The CstA is involved in nutrient scavenging and peptide transport [101, 102]. The BolA regulates expression of many cell wall proteins and is partially responsible for the coccoid morphology of stationary phase cells in *E. coli* [19, 103]. Phosphorylation of histidine and aspartate is very important in bacterial regulatory systems, and SixA (signal inhibitor X) is one of the earliest discovered phosphohistidine phosphatases [104]. On the other hand, 52 signal transduction-related genes and 25 transcription factors were down-regulated in both the (p)ppGpp^0^_*Pst*DC3000_ and (p)ppGpp^0^_*Pss*B728a_ mutants. These genes included carbon storage regulator CsrA2, diguanylate cyclase, HrpL and ribose operon repressor RbsR (Additional file 5: Table S13). These results indicated that (p) ppGpp positively and negatively controlled transcription factors and signal transduction-related genes, thereby regulating global gene expression for adapting to changes in the extracellular environments.

### Differential regulation by (p) ppGpp in *Pst*DC3000 and *Pss*B728a

Comparative transcriptomic analysis also identified unique and homologous DEGs similarly or inversely regulated by (p) ppGpp in *Pst*DC3000 and *Pss*B728a (Table 1). Phytotoxin is very important for *P. syringae* pathogenesis. However, different phytotoxins are produced in *Pst*DC3000 and *Pss*B728a. *Pst*DC3000 produces the non-host-specific and chlorosis-inducing coronatine (COR) [105], whereas *Pss*B728a produces syringomycins and syringopeptins, which could form pores in plasma membranes, resulting in necrotic symptoms [106]. Our data showed that coronatine (*corR* and *corS*) and coronamic acid synthetase genes (*cmaBCTU* gene cluster) in the (p)ppGpp^0^_*Pst*DC3000_ mutant, and syringomycin synthesis genes (*syrB1CDEFP* gene cluster) in the ppGpp^0^_*Pss*B728a_ mutant were all down-regulated (Table 2). These results indicated that (p) ppGpp positively activate toxin gene expression to promote bacterial virulence in both *Pst*DC3000 and *Pss*B728a.

**Table 1:** Differential gene regulation by (p) ppGpp in *Pst*DC3000 and *Pss*B728a

		Homolog genes inversely regulated	***Pst***DC3000 (unique genes)		***Pss***B728a (unique genes)	
Symbol	Functional classification (COGs)		Up	Down	Up	Down
	**Type III secretion system**	**0**	**0**	**37**	**0**	**7**
**J**	**Translation, ribosomal structure and biogenesis**	**4**	**0**	**2**	**0**	**3**
**A**	**RNA processing and modification**	**0**	**0**	**0**	**0**	**0**
**K**	**Transcription**	**13**	**9**	**8**	**1**	**4**
**L**	**Replication, recombination and repair**	**4**	**8**	**15**	**0**	**8**
**D**	**Cell cycle control, cell division, chromosome partitioning**	**2**	**1**	**1**	**0**	**1**
**V**	**Defense mechanisms**	**1**	**0**	**11**	**0**	**6**
**T**	**Signal transduction mechanisms**	**16**	**3**	**10**	**0**	**9**
**M**	**Cell wall/membrane/envelope biogenesis**	**14**	**1**	**8**	**2**	**14**
**N**	**Cell motility**	**5**	**0**	**1**	**0**	**3**
**U**	**Intracellular trafficking, secretion, and vesicular transport**	**15**	**1**	**1**	**1**	**9**
**O**	**Posttranslational modification, protein turnover, chaperones**	**4**	**0**	**5**	**0**	**2**
**C**	**Energy production and conversion**	**15**	**0**	**7**	**1**	**3**
**G**	**Carbohydrate transport and metabolism**	**9**	**0**	**9**	**1**	**8**
**E**	**Amino acid transport and metabolism**	**20**	**1**	**15**	**1**	**9**
**F**	**Nucleotide transport and metabolism**	**1**	**0**	**3**	**0**	**0**
**H**	**Coenzyme transport and metabolism**	**6**	**0**	**2**	**0**	**1**
**I**	**Lipid transport and metabolism**	**9**	**3**	**3**	**1**	**4**
**P**	**Inorganic ion transport and metabolism**	**16**	**0**	**10**	**0**	**4**
**Q**	**Secondary metabolites biosynthesis, transport and catabolism**	**3**	**0**	**16**	**2**	**19**
**S**	**Function unknown**	**101**	**64**	**199**	**29**	**145**
**Total**		**258**	**91**	**362**	**39**	**259**

*COG* Clusters of orthologous groups (http://www.ncbi.nlm.nih.gov/COG). See methods for description

**Table 2:** List of phytotoxin genes differentially regulated by (p) ppGpp in *Pst*DC3000 and *Pss*B728a

Locus tag	Description	(p)ppGpp^**0**^_***Pst*DC3000**_ /***Pst***DC3000	(p)ppGpp^**0**^_***Pss*B728a**_ /***Pss***B728a
**DC3000**			
**Phytotoxin**			
**Coronatine**			
*PSPTO_4704*	DNA-binding response regulator CorR	**− 2.59**	**/**
*PSPTO_4705*	sensor histidine kinase CorS	**− 1.81**	**/**
**Coronamic acid**			
*PSPTO_4710*	coronamic acid synthetase CmaB	**− 2.02**	**/**
*PSPTO_4711*	coronamic acid synthetase CmaC	**−1.53**	**/**
*PSPTO_4712*	coronamic acid synthetase, CmaT	**−1.68**	**/**
*PSPTO_4714*	CmaU protein	**−1.15**	**/**
**B728a Syringomycin**			
*PSYR_2607*	regulatory protein SyrF	**/**	**−2.16**
*PSYR_2608*	amino acid adenylation SyrE	**/**	**−1.81**
*PSYR_2609*	Alpha/beta hydrolase fold SyrC	**/**	**−1.34**
*PSYR_2611*	amino acid adenylation SyrB1	**/**	**−1.23**
*PSYR_2612*	SyrP protein	**/**	**−2.97**
*PSYR_2613*	cyclic peptide transporter SyrD	**/**	**−2.03**

DEGs were differentially expressed genes in the (p)ppGpp^0^_*Pst*DC3000_ and (p)ppGpp^0^_*Pss*B728a_ with *p*-value < 0.05 between the WT and the ppGpp^0^ mutants. ‘/’ represents that gene is not present

Previous report showed that *Pst*DC3000 carried three putative T6SS clusters (*HSI-I*, *HSI-II*, and *ppkA*), whereas *Pss*B728a only carried two (*HIS-I* and *ppkA*) [30]. In this study, only 7 homologous genes belonging to the *ppkA* locus were down-regulated in both the (p)ppGpp^0^_*Pst*DC3000_ and (p)ppGpp^0^_*Pss*B728a_ mutants (Fig. 5a). Fifteen T6SS genes on *HSI-I* cluster in *Pss*B728a, and 18 T6SS genes on *HSI-II* cluster, but no genes on *HSI-I* in *Pst*DC3000, were positively regulated by (p) ppGpp (Table 3), indicating that different T6SSs may be regulated differently by (p) ppGpp in *Pst*DC3000 and *Pss*B728a. Furthermore, we found that some homologous genes in *Pst*DC3000 and *Pss*B728a were inversely regulated by (p)ppGpp. For example, cell motility genes (*pilF*, *fliQ*, *flgHIG*) and cobalamin biosynthetic genes (*cobOPST*) were up-regulated in the (p)ppGpp^0^_*Pst*DC3000_ mutant, but down-regulated in the (p)ppGpp^0^_*Pss*B728a_ mutant. In contrast, nitrogen regulation (*ntrBC*) and general secretion pathway (GSP) (*gspDEFGHIJ*) genes were down-regulated in the (p)ppGpp^0^_*Pst*DC3000_ mutant, but up-regulated in the (p)ppGpp^0^_*Pss*B728a_ mutant (Table 4).

**Table 3:** List of T6SS genes differentially regulated by (p) ppGpp in *Pst*DC3000 and *Pss*B728a

Locus tag	Description	(p)ppGpp^**0**^_***Pst*DC3000**_ /***Pst***DC3000	(p)ppGpp^**0**^_***Pss*B728a**_ /***Pss***B728a
**DC3000**			
*PSPTO_2539*	secreted protein Hcp-1	**−1.37**	**/**
*PSPTO_2550*	hypothetical protein PSPTO_2550	**−1.83**	**/**
*PSPTO_2553*	hypothetical protein PSPTO_2553	**−1.38**	**/**
*PSPTO_4385*	Rhs element Vgr protein	**−1.26**	**/**
*PSPTO_5415*	Rhs element Vgr protein	**−1.79**	**/**
*PSPTO_5416*	serine/threonine protein kinase	**−1.63**	**/**
*PSPTO_5417*	serine/threonine phosphoprotein phosphatase	**−2.81**	**/**
*PSPTO_5418*	hypothetical protein PSPTO_5418	**−2.85**	**/**
*PSPTO_5419*	hypothetical protein PSPTO_5419	**−2.85**	**/**
*PSPTO_5420*	hypothetical protein PSPTO_5420	**−2.48**	**/**
*PSPTO_5421*	lipoprotein	**−2.84**	**/**
*PSPTO_5422*	FHA domain-containing protein	**−2.25**	**/**
*PSPTO_5423*	hypothetical protein PSPTO_5423	**−2.01**	**/**
*PSPTO_5424*	sigma-54 dependent transcriptional regulator	**−1.67**	**/**
*PSPTO_5425*	ClpB protein	**−1.82**	**/**
*PSPTO_5426*	hypothetical protein PSPTO_5426	**−1.96**	**/**
*PSPTO_5427*	hypothetical protein PSPTO_5427	**−2.16**	**/**
*PSPTO_5430*	hypothetical protein PSPTO_5430	**−1.75**	**/**
*PSPTO_5435*	secreted protein Hcp-2	**−1.29**	**/**
*PSPTO_5436*	Rhs element Vgr protein	**−2.59**	**/**
*PSPTO_5437*	hypothetical protein PSPTO_5437	**−2.91**	**/**
*PSPTO_5438*	Rhs family protein	**−1.77**	**/**
**B728a**			
*PSYR_1935*	hypothetical protein PSYR_1935	**/**	**−1.04**
*PSYR_2632*	virulence protein SrfB	**/**	**−3.45**
*PSYR_2633*	hypothetical protein PSYR_2633	**/**	**−1.76**
*PSYR_4955*	hypothetical protein PSYR_4955	**/**	**−1.57**
*PSYR_4956*	hypothetical protein PSYR_4956	**/**	**−2.04**
*PSYR_4957*	hypothetical protein PSYR_4957	**/**	**−2.66**
*PSYR_4958*	ATPase AAA	**/**	**−3.3**
*PSYR_4959*	hypothetical protein PSYR_4959	**/**	**−3.23**
*PSYR_4960*	hypothetical protein PSYR_4960	**/**	**−3.61**
*PSYR_4961*	hypothetical protein PSYR_4961	**/**	**−4.21**
*PSYR_4962*	hypothetical protein PSYR_4962	**/**	**−3.88**
*PSYR_4963*	hypothetical protein PSYR_4963	**/**	**−3.12**
*PSYR_4964*	OmpA/MotB protein	**/**	**−2.83**
*PSYR_4965*	hypothetical protein PSYR_4965	**/**	**−4.22**
*PSYR_4966*	ImpA-like protein	**/**	**−3.72**
*PSYR_4967*	hypothetical protein PSYR_4967	**/**	**−2.8**
*PSYR_4974*	Rhs element Vgr protein	**/**	**−1.91**
*PSYR_4983*	Rhs element Vgr protein	**/**	**−2.54**

DEGs were differentially expressed genes in the (p)ppGpp^0^_*Pst*DC3000_ and (p)ppGpp^0^_*Pss*B728a_ with p-value < 0.05 between the WT and the ppGpp^0^ mutants. ‘/’ represents that gene is not present or not significantly regulated

**Table 4:** List of homologous genes regulated by (p) ppGpp in opposite ways in *Pst*DC3000 and *Pss*B728a

Locus tag		Gene description	(p)ppGpp^**0**^_***Pst*DC3000**_/***Pst***DC3000	(p)ppGpp^**0**^_***Pss*B728a**_/***Pss***B728a
**DC3000**	**B728A**			
*PSPTO_1432*	*PSYR_1246*	type IV pilus biogenesis protein PilF	**1.29**	**−0.85**
*PSPTO_1940*	*PSYR_3475*	flagellar basal-body rod protein FlgG	**0.45**	**−1.27**
*PSPTO_1941*	*PSYR_3474*	flagellar L-ring protein FlgH	**0.61**	**−1.36**
*PSPTO_1973*	*PSYR_3443*	flagellar biosynthetic protein FliQ	**0.22**	**−1.36**
*PSPTO_1942*	*PSYR_3473*	flagellar P-ring protein FlgI	**0.36**	**−1.43**
*PSPTO_1713*	*PSYR_3676*	cobyric acid synthase CobQ	**1.82**	**− 0.20**
*PSPTO_1714*	*PSYR_3675*	cobinamide kinase/cobinamide phosphate guanylyltransferase CobP	**1.56**	**−0.57**
*PSPTO_1717*	*PSYR_3672*	cobalamin (5\’-phosphate) synthase CobS	**1.02**	**−1.04**
*PSPTO_1715*	*PSYR_3674*	nicotinate-nucleotide--dimethylbenzimidazole phosphoribosyltransferase CobT	**1.66**	**−1.08**
*PSPTO_0352*	*PSYR_4822*	nitrogen regulation protein NR(I) NtrC	**−0.60**	**1.61**
*PSPTO_0353*	*PSYR_4821*	nitrogen regulation protein NtrB	**−0.29**	**2.47**
*PSPTO_3315*	*PSYR_3149*	general secretion pathway protein G, GspG	**−0.67**	**1.69**
*PSPTO_3314*	*PSYR_3148*	general secretion pathway protein H, GspH	**−0.70**	**1.40**
*PSPTO_3317*	*PSYR_3151*	general secretion pathway protein E	**−0.36**	**1.36**
*PSPTO_3316*	*PSYR_3150*	general secretion pathway protein F, GspE	**−0.69**	**1.34**
*PSPTO_3313*	*PSYR_3147*	general secretion pathway protein I, GspI	**−0.93**	**1.09**
*PSPTO_3312*	*PSYR_3146*	general secretion pathway protein J, GspJ	**−1.01**	**0.80**
*PSPTO_3307*	*PSYR_3141*	general secretion pathway protein D, GspD	**−1.18**	**0.11**

DEGs were differentially expressed genes in the (p)ppGpp^0^_*Pst*DC3000_ and (p)ppGpp^0^_*PssB*728a_ with p-value < 0.05 between the WT and the (p)ppGpp^0^ mutants

## Conclusions

**Fig. 7:**
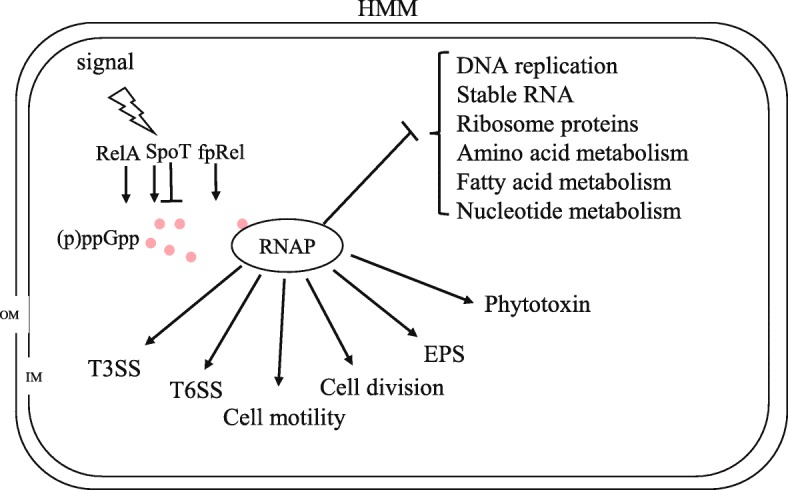
Proposed model illustrating the global effect of (p) ppGpp in *Pseudomonas syringae*. (p) ppGpp binds to RNAP and switches transcription from growth-related genes to virulence factors and stress relate genes. Symbols:↓, positive effect;⊥, negative effect; RNAP, RNA polymerase

The model presented in Fig. 7 is based on our global transcription data as well as previously reported results in *Pst*DC3000 and *Pss*728a [16, 17]. In HMM, the RelA/SpoT/FpRel systems are activated in *Pst*DC3000 and *Pss*B728a, leading to accumulation of (p)ppGpp. Thereafter, the (p)ppGpp-mediated stringent response indirectly promotes alternative sigma factor activities, such as RpoN and HrpL, leading to the expression of T3SS and other virulence factors, as well as stress-related genes. In both *Pst*DC3000 and *Pss*B728a, (p) ppGpp suppresses biosynthesis of DNA replication, RNA processes, ribosome proteins, nucleotide metabolism, amino acid metabolism and fatty acid metabolism and other basic physical processes, and at the same time, activates the expression of T3SS, T6SS, cell motility, cell division, EPS and phytotoxin to promote virulence and survival.

## Methods

### Bacterial strains and growth conditions

The wild-type *Pst*DC3000 and its *relA*/*spoT*/*fpRel* triple mutant ((p)ppGpp^0^_*Pst*DC3000_), and the wild-type *Pss*B728a and its *relA*/*spoT* double mutant ((p)ppGpp^0^_*Pss*B728a_) [26, 27] were routinely cultured on King’s medium B (KB) plate, or 5 ml of fresh KB broth at 28 °C with shaking at 250 rpm. The *hrp*-inducing medium (HMM) supplemented with 10 mM fructose as carbon source was used for RNA isolation [26, 27, 107]. Bacterial growth was monitored by measuring absorbance of cell suspensions at 600 nm. Antibiotics were supplied at the following final concentrations: rifampicin, 100 μg ml^− 1^ and kanamycin, 50 μg ml^− 1^.

### RNA extraction for RNA-seq and cDNA synthesis

Overnight cultures of the bacterial strains were collected by centrifugation, and washed with HMM for three time. The suspensions were adjusted to OD_600_ = 0.2 in HMM and incubated in 5 ml HMM at 18 °C for 3 h. Four ml of RNA protect reagent (Qiagen, Hilden, Germany) was added to 2 ml of bacterial culture mixed by vortex and incubated at room temperature for 5 min. Cells were harvested by centrifugation and total RNAs were extracted using RNeasy® mini kit (Qiagen) according to the manufacturer’s instructions. DNase I treatment was performed with TURBO DNA-free kit (Ambion, TX, USA). The quantity and quality of RNA samples was determined using Nano-drop ND100 spectrophotometer (Nano-Drop Technologies; Wilmington, DE, USA), and/or using Agilent RNA 6000 Nano Chip Bioanalyzer (Agilent, Santa Clara, CA, USA).

### Illumina sequencing and RNA-seq analysis

Library construction and sequencing of three biological samples of *Pst*DC3000, *Pss*B728a and their mutants using the Illumina HiSeq 2500 (Illumina, San Diego, CA, USA) were performed by the Keck Center at the University of Illinois at Urbana-Champaign (UIUC). A total of twelve stranded libraries were constructed using TruSeq Stranded RNA Sample Prep kit following the manufacturer’s instructions (Illumina, San Diego, CA, USA). The sequence reads were aligned to the genome of *Pst*DC3000 [108] and *Pss*B728a [6] using Bowtie 0.12.7 [109]. Samtools and bedtools were performed for getting the read counts per CDS. Normalized log2-based count per million values (log_2_CPM) were calculated after trimmed mean of M values (TMM) normalization in the edgeR package [110, 111].

To examine the gene expression dynamics among all the samples, a PCA was conducted by using prcomp in R. To perform normalization and statistical analysis on the raw read counts, the R package edgeR was used as described previously [110, 111]. DEGs were defined as genes with a |log_2_FC (fold change)| value ≥1 and a corrected *p* value < 0.05 from three biological samples. To visualize overall expression pattern of individual genes, the MA plots (log_2_FC versus Average Log_2_CPM; FC, fold change; CPM, counts per million reads) were generated. For functionally categorization of DEGs using COGs, protein sequence of all coding genes in *Pst*DC3000 (accession #: AE016853.1) and *Pss*B728a (accession #: CP000075.1) were downloaded from NCBI (https://www.ncbi.nlm.nih.gov/). The two FASTA protein files were used as input for protein annotation using eggNOG-mapper (http://eggnogdb.embl.de/#/app/emapper). COG information for DEGs was extracted from eggNOG output file. In addition, genes involved in type III secretion system (T3SS) were manually grouped into an additional orthologous categorization. The RNA-seq data files have been submitted to Gene Expression Omnibus (GEO) at the National Center for Biotechnology Information (NCBI) with an accession number GSE143325.

### Identification of homologous and unique genes in *Pst*DC3000 and *Pss*B728a

Coding sequences in the genomes of *Pst*DC3000 and *Pss*B728a were downloaded from the NCBI database. BLASTP cutoff scores of E < 10^− 2^ and 40% identity were used to distinguish homologues and unique genes between *Pst*DC3000 and *Pss*B728a.

### Quantitative real-time PCR (qRT-PCR)

For qRT-PCR, 1 μg of RNA was reverse transcribed to cDNA following the manufacturer’s instruction for the SperScript™ III Reverse Transcriptase (Invitrogen, Carlsbad, CA, USA). Concentration of cDNA was adjusted to 100 ng/μl as template and the Power Up SYBR® Green PCR master mix (Applied Biosystems, CA, USA) was used for qRT-PCR to detect gene expression of selected genes. The qRT-PCR amplifications were conducted in the StepOnePlus Real-Time PCR system (Applied Biosystems) under the following conditions: 50 °C for 2 min, and 95 °C for 2 min followed by 40 cycles of 95 °C for 15 s and 60 °C for 1 min. The *gltB* gene was used as an endogenous control to calculate relative quantification (ΔΔC_t_) [112]. All primers are listed in Additional file 6. The experiment was repeated and three biological replicates were performed for each gene. Comparative ΔΔC_t_ method was used to analyze the data and statistical analysis was performed using student’s t test with *P* < 0.05.

## Supplementary information

**Additional file 1: Figure S1** Heatmap. (A) (p)ppGpp^0^_*Pst*DC3000_ versus *Pst*DC3000: 1886 differentially expressed genes (DEGs). (B) (p)ppGpp^0^_*Pss*B728a_ versus *Pss*B728a: 1562 DEGs. Up and down regulated genes were indicated by red and blue lines. **Figure S2 MA plots.** (A) (p)ppGpp^0^_*Pst*DC3000_ versus *Pst*DC3000. (B) (p)ppGpp^0^_*Pss*B728a_ versus *Pss*B728a. M: log_2_FC, A: Average Log_2_CPM, counts per million reads. Dots between two purple lines represent |log2FC| value ≤1, and outside dots represent |log_2_FC| value ≥1. Up and down regulated genes were indicated by red and blue dot with *p* value < 0.05, black dot represents no signification difference and p value > 0.05.

**Additional file 2: Table S1.** List of differentially expressed genes (DEGs) of (p)ppGpp^0^_*Pst*DC3000_ versus *Pst*DC3000. **Table S2.** List of DEGs of (p)ppGpp^0^_*B728a*_ versus *Pss*B728a.

**Additional file 3: Table S3.** List of homologues and unique genes via comparative analysis of *Pst*DC3000 and *Pss*B728a genomes. **Table S4.** List of homologues genes in both *Pst*DC3000 and *Pss*B728a regulated by (p) ppGpp in a similar way. **Table S5.** List of homologues genes in both *Pst*DC3000 and *Pss*B728a inversely regulated by (p)ppGpp.

**Additional file 4: Table S6.** List of unique genes in *Pst*DC3000 regulated by (p)ppGpp. **Table S7.** List of unique genes of *Pss*B728a regulated by (p)ppGpp.

**Additional file 5: Table S8.** List of differentially expressed genes (DEGs) related to type III secretion system (T3SS) and type VI secretion system (T6SS). **Table S9.** List of DEGs related to cell motility, division, and exopolysaccharides (EPS). **Table S10.** List of DEGs related to DNA replication, RNA processes and ribosomal protein biosynthesis. **Table S11.** List of DEGs related to nucleotide, amino acid and fatty acid metabolism. **Table S12.** List of DEGs related to coenzyme and carbon metabolism. **Table S13.** List of DEGs related to signal transduction and transcription.

**Additional file 6: Table S14.** Primers for qRT-PCR used in this study.

## Data Availability

The datasets generated during the current study are available in the Gene Expression Omnibus (GEO) at the National Center for Biotechnology Information (NCBI) with an accession number GSE143325.

## Abbreviations

(p)ppGpp: guanosine tetra/pentaphosphate
*Pst*DC3000: *P. syringae* pv. *tomato* DC3000
*Pss*B728a: *P. syringae* pv. *syringae* B728a
RSH: RelA-SpoT homologue
RNAP: RNA polymerase
TMM: Trimmed mean of M values
CPM: Counts per million reads
FC: Fold change
GEO: Gene Expression Omnibus
PCA: Principal component analysis
COGs: Clusters of orthologous groups
DEGs: Differentially expressed genes
T3SS: Type III secretion system
T6SS: Type VI secretion system
EPSs: Exopolysaccharides
GSP: General secretion pathway
