# Supplementary material for: Comparative transcriptomic analysis of global gene expression mediated by (p) ppGpp reveals common regulatory networks in Pseudomonas syringae
Source: BMC Genomics. 2020 Apr 10;21:296. doi: 10.1186/s12864-020-6701-2 (PMC7146990; doi:10.1186/s12864-020-6701-2)
Supplement: Supplementary file 1 — Additional file 1: Figure S1 Heatmap. (A) (p)ppGpp0PstDC3000 versus PstDC3000: 1886 differentially expressed genes (DEGs). (B) (p)ppGpp0PssB728a versus PssB728a: 1562 DEGs. Up and down regulated genes were indicated by red and blue lines. Figure S2 MA plots. (A) (p)ppGpp0PstDC3000 versus PstDC3000. (B) (p)ppGpp0PssB728a versus PssB728a. M: log2FC, A: Average Log2CPM, counts per million reads. Dots between two purple lines represent |log2FC| value ≤1, and outside dots represent |log2FC| value ≥1. Up and down regulated genes were indicated by red and blue dot with p value < 0.05, black dot represents no signification difference and p value > 0.05. [file 12864_2020_6701_MOESM1_ESM.pdf]

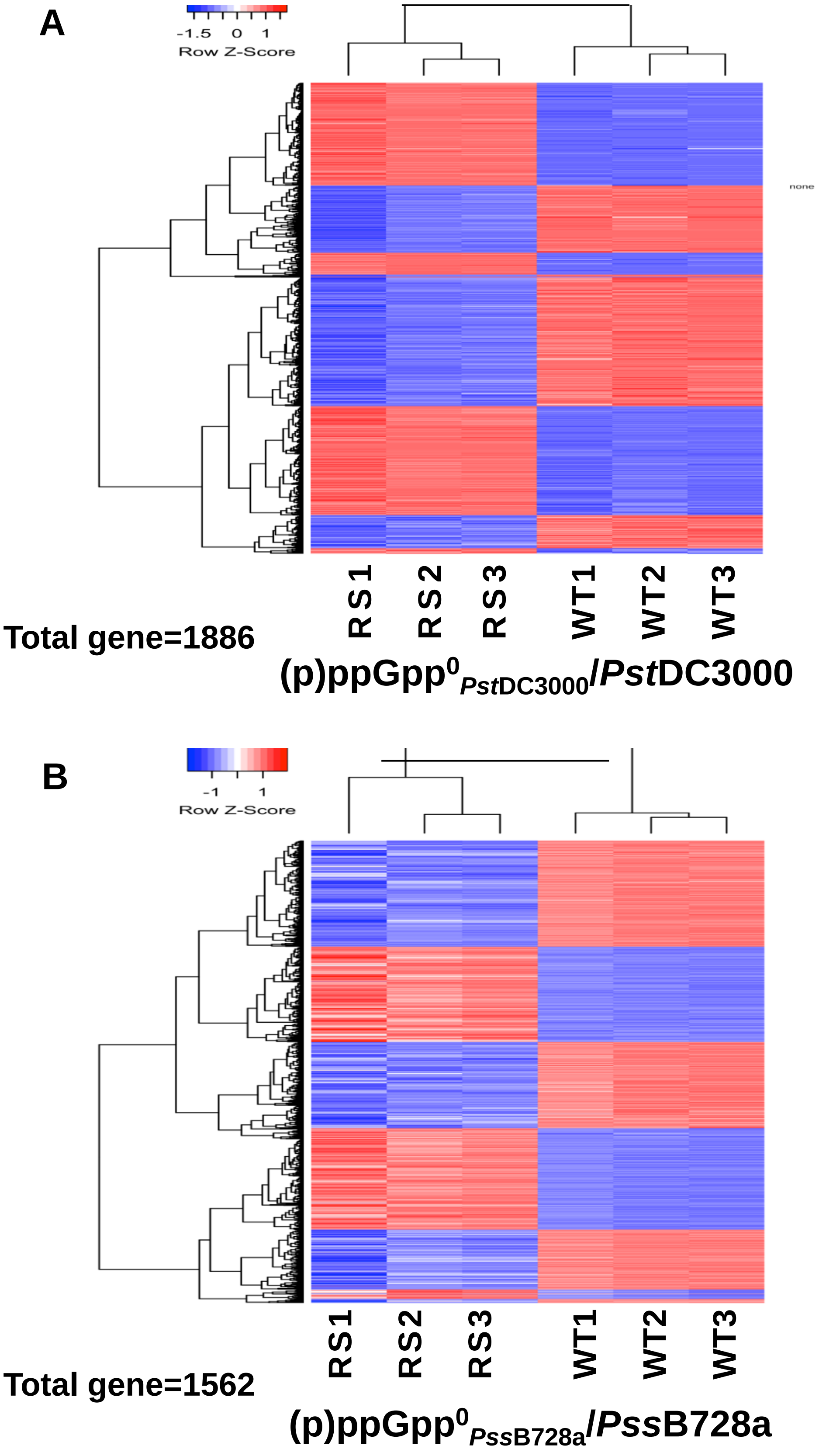

Additional file 1: Figure S1

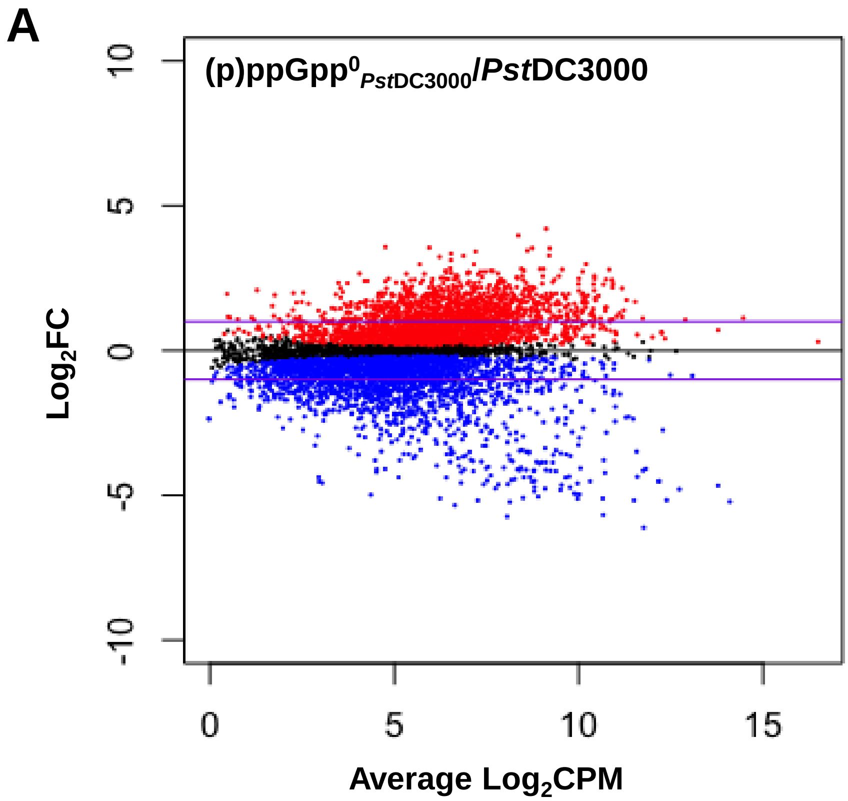

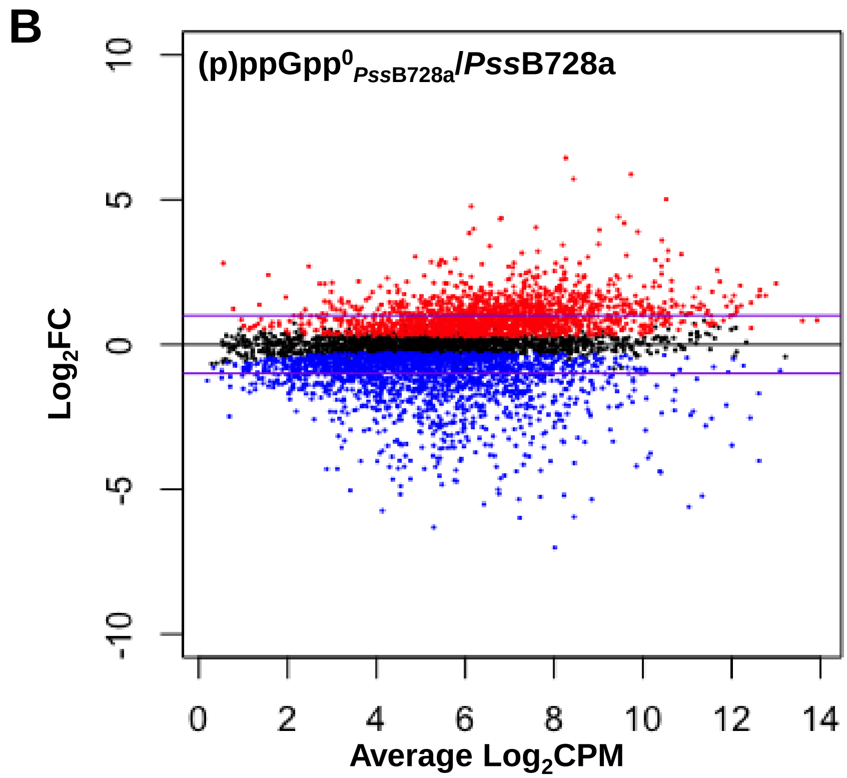

Additional File 1: Figure S2
