# Supplementary material for: Comparative transcriptomic analysis of global gene expression mediated by (p) ppGpp reveals common regulatory networks in Pseudomonas syringae
Source: BMC Genomics. 2020 Apr 10;21:296. doi: 10.1186/s12864-020-6701-2 (PMC7146990; doi:10.1186/s12864-020-6701-2)
Supplement: Supplementary file 2 — Additional file 2: Table S1. List of differentially expressed genes (DEGs) of (p)ppGpp0PstDC3000 versus PstDC3000. Table S2. List of DEGs of (p)ppGpp0B728a versus PssB728a. [file 12864_2020_6701_MOESM2_ESM.pdf]

Table S1. List of differentially expressed genes of (p) ppGpp$^{0}_{Pst\mathrm{DC3000}}$ versus *Pst*DC3000 with |log$_2$FC| value ≥1 and an adjusted p-value <0.05.

Locus tag	Gene description	(p)ppGpp$^{0}_{Pst\mathrm{DC3000}}$ /*Pst*DC3000
**Type III secretion system**		
PSPTO_4594	type III effector HopO1-2	**-1.01**
PSPTO_4592	type III effector HopO1-3	**-1.13**
PSPTO_3292	type III effector HopAH2-1	**-1.14**
PSPTO_0906	type III effector HopAI1	**-1.18**
PSPTO_4593	type III effector HopT1-2	**-1.36**
PSPTO_0474	type III effector HopAS1, partial	**-1.41**
PSPTO_0905	*hopAH1*, type III effector HopAH1	**-1.5**
PSPTO_1568	*hopAF1*, type III effector HopAF1	**-2.25**
PSPTO_4724	type III effector HopD	**-2.33**
PSPTO_4001	*avrPto1*, type III effector protein AvrPto1	**-2.36**
PSPTO_0901	*hopAG1*, type III effector HopAG1	**-2.53**
PSPTO_1404	*hrpL*, RNA polymerase sigma factor HrpL	**-2.82**
PSPTO_4703	type III effector HopAQ1	**-2.98**
PSPTO_0501	type III effector HopU1	**-3.09**
PSPTO_1376	*shcE*, type III chaperone ShcE	**-3.2**
PSPTO_1372	hopAA1-1, type III effector HopAA1-1	**-3.37**
PSPTO_1402	*hrcV*, type III secretion protein HrcV	**-3.45**
PSPTO_1403	*hrpJ*, type III secretion protein HrpJ	**-3.46**
PSPTO_1387	type III secretion protein HrpF	**-3.5**
PSPTO_1397	*hrcQa*, type III secretion protein HrcQa	**-3.54**
PSPTO_1388	*hrpG*, type III secretion protein HrpG	**-3.55**
PSPTO_1393	*hrcT*, type III secretion protein HrcT	**-3.58**
PSPTO_0852	type III helper protein HopAJ1	**-3.58**
PSPTO_0883	type III effector HopR1	**-3.6**
PSPTO_1392	*hrcU*, type III secretion protein HrcU	**-3.73**
PSPTO_1396	*hrcQb*, type III secretion protein HrcQb	**-3.82**
PSPTO_0503	type III chaperone protein ShcF	**-3.82**
PSPTO_0061	type III effector HopY1	**-3.85**
PSPTO_4599	type III chaperone ShcS1	**-3.87**
PSPTO_4718	type III effector HopAA1-2	**-3.87**
PSPTO_0877	type III effector HopQ1-1	**-3.87**
PSPTO_1394	*hrcS*, type III secretion protein HrcS	**-3.98**
PSPTO_4691	type III effector HopAD1	**-3.99**
PSPTO_1391	*hrpV*, negative regulator of hrp expression HrpV	**-4.04**
PSPTO_1395	*hrcR*, type III secretion protein HrcR	**-4.05**
PSPTO_0589	type III effector HopC1	**-4.07**
PSPTO_5353	type III chaperone protein ShcA	**-4.07**
PSPTO_1378	*hrpH*, membrane-bound lytic murein transglycosylase D	**-4.11**
PSPTO_1377	*avrE1*, type III effector protein AvrE1	**-4.13**
PSPTO_3087	*hopAB2*, type III effector HopAB2	**-4.14**
PSPTO_1401	*hrpQ*, type III secretion protein HrpQ	**-4.15**
PSPTO_1398	*hrpP*, type III secretion protein HrpP	**-4.17**
PSPTO_4722	type III effector HopAO1	**-4.17**
PSPTO_0502	type III effector HopF2	**-4.19**
PSPTO_1389	*hrcC*, outer-membrane type III secretion protein HrcC	**-4.22**

PSPTO_4589	type III chaperone ShcS2	**-4.31**
PSPTO_1405	*hrpK1*, type III helper protein HrpK1	**-4.36**
PSPTO_4776	*hopI1*, type III effector HopI1	**-4.36**
PSPTO_1369	type III chaperone protein ShcN	**-4.38**
PSPTO_4597	type III effector HopS1	**-4.38**
PSPTO_1386	*hrpE*, type III secretion protein HrpE	**-4.43**
PSPTO_1390	*hrpT*, type III secretion protein	**-4.47**
PSPTO_1374	*shcM*, type III chaperone ShcM	**-4.49**
PSPTO_1400	*hrcN*, type III secretion cytoplasmic ATPase HrcN	**-4.51**
PSPTO_1022	type III effector HopAM1-1	**-4.51**
PSPTO_2678	type III helper protein HopP1	**-4.52**
PSPTO_4101	*hopAK1*, type III helper protein HopAK1	**-4.59**
PSPTO_4720	type III effector HopV1	**-4.62**
PSPTO_4588	type III effector HopS2	**-4.63**
PSPTO_1375	*hopM1*, type III effector HopM1	**-4.64**
PSPTO_1381	type III helper protein HrpA1	**-4.67**
PSPTO_1399	*hrpO*, type III secretion protein HrpO	**-4.72**
PSPTO_5354	type III effector HopA1	**-4.73**
PSPTO_1385	*hrpD*, type III secretion protein HrpD	**-4.76**
PSPTO_1370	type III effector HopN1	**-4.82**
PSPTO_0588	hopH1, type III effector HopH1	**-4.94**
PSPTO_1406	type III effector HopB1	**-4.96**
PSPTO_1384	*hrcJ*, type III secretion protein HrcJ	**-5.11**
PSPTO_0876	type III effector HopD1	**-5.11**
PSPTO_4721	type III chaperone ShcV	**-5.11**
PSPTO_1383	*hrpB*, type III secretion protein HrpB	**-5.17**
PSPTO_1382	*hrpZ1*, type III restriction system endonuclease	**-5.22**
PSPTO_4331	type III effector HopE1	**-5.68**
PSPTO_4727	type III effector HopG1	**-5.73**
PSPTO_1373	*hrpW1*, type III helper protein HrpW1	**-6.12**
**Translation, ribosomal structure and biogenesis**		
PSPTO_3352	*infA*, translation initiation factor IF-1	**4.2**
PSPTO_5136	*rpmE-2*, 50S ribosomal protein L31	**3.27**
PSPTO_0090	*rpmG*, 50S ribosomal protein L33	**2.71**
PSPTO_3750	hypothetical protein PSPTO_3750	**2.63**
PSPTO_5615	*rpmH*, 50S ribosomal protein L34	**2.56**
PSPTO_0802	*rpsT*, 30S ribosomal protein S20	**2.47**
PSPTO_0089	*rpmB*, 50S ribosomal protein L28	**2.44**
PSPTO_5138	*argS*, arginyl-tRNA synthetase	**2.43**
PSPTO_1420	RNA methyltransferase, TrmH family, group 1	**2.37**
PSPTO_4425	*rpsI*, 30S ribosomal protein S9	**2.31**
PSPTO_1146	*rsmC*, ribosomal RNA small subunit methyltransferase C	**2.3**
PSPTO_4019	ribosomal protein S12 methylthiotransferase	**2.25**
PSPTO_0072	endoribonuclease L-PSP	**2.19**
PSPTO_4645	methyltransferase	**2.19**
PSPTO_3835	*rpmF*, 50S ribosomal protein L32	**2.14**
PSPTO_4862	*prmA*, ribosomal protein L11 methyltransferase	**2.14**
PSPTO_4426	*rplM*, 50S ribosomal protein L13	**2.14**
PSPTO_0184	*glyQ*, glycyl-tRNA synthetase subunit alpha	**2.14**

PSPTO_0177	*def-1*, polypeptide deformylase	**2.12**
PSPTO_1476	*rplS*, 50S ribosomal protein L19	**1.97**
PSPTO_4930	*rplI*, 50S ribosomal protein L9	**1.94**
PSPTO_1744	initiation factor 2 subunit family	**1.94**
PSPTO_1501	*lysS*, lysyl-tRNA synthetase	**1.93**
PSPTO_0963	*pcnB*, poly(A) polymerase	**1.92**
PSPTO_0827	*rluD*, ribosomal large subunit pseudouridine synthase D	**1.91**
PSPTO_4183	*rpmE-1*, 50S ribosomal protein L31 type B	**1.9**
PSPTO_0539	*rpsU*, 30S ribosomal protein S21	**1.87**
PSPTO_1818	hypothetical protein PSPTO_1818	**1.87**
PSPTO_0618	*rplL*, 50S ribosomal protein L7/L12	**1.84**
PSPTO_1412	*queA*, S-adenosylmethionine--tRNA ribosyltransferase-isomerase	**1.83**
PSPTO_1561	hypothetical protein PSPTO_1561	**1.82**
PSPTO_3922	*rnd*, ribonuclease D	**1.79**
PSPTO_4021	S4 domain-containing protein	**1.78**
PSPTO_1473	*rpsP*, 30S ribosomal protein S16	**1.76**
PSPTO_0077	*rph*, ribonuclease PH	**1.76**
PSPTO_1413	*tgt*, queuine tRNA-ribosyltransferase	**1.73**
PSPTO_0625	*rpsJ*, 30S ribosomal protein S10	**1.73**
PSPTO_3358	*trmU*, tRNA (5-methyl aminomethyl-2-thiouridylate)-methyltransferase	**1.72**
PSPTO_1103	ribosomal 5S rRNA E-loop binding protein Ctc/L25/TL5	**1.7**
PSPTO_1534	*rpsB*, 30S ribosomal protein S2	**1.68**
PSPTO_0616	*rplA*, 50S ribosomal protein L1	**1.66**
PSPTO_3817	*truA*, tRNA pseudouridine synthase A	**1.65**
PSPTO_0640	*rpsH*, 30S ribosomal protein S8	**1.62**
PSPTO_4932	*rpsR*, 30S ribosomal protein S18	**1.61**
PSPTO_1431	radical SAM enzyme, Cfr family	**1.61**
PSPTO_0185	*glyS*, glycyl-tRNA synthetase subunit beta	**1.59**
PSPTO_4812	*leuS*, leucyl-tRNA synthetase	**1.59**
PSPTO_5323	TrmH family RNA methyltransferase	**1.57**
PSPTO_3840	*rluC*, ribosomal large subunit pseudouridine synthase C	**1.51**
PSPTO_1475	*trmD*, tRNA (guanine-N1)-methyltransferase	**1.49**
PSPTO_0617	*rplJ*, 50S ribosomal protein L10	**1.49**
PSPTO_0615	*rplK*, 50S ribosomal protein L11	**1.49**
PSPTO_1474	*rimM*, 16S rRNA-processing protein RimM	**1.47**
PSPTO_1268	*valS*, valyl-tRNA synthetase	**1.43**
PSPTO_1533	*map-1*, methionine aminopeptidase	**1.43**
PSPTO_0797	*rplU*, 50S ribosomal protein L21	**1.4**
PSPTO_2378	*thrS*, threonyl-tRNA synthetase	**1.38**
PSPTO_1500	*prfB*, hypothetical protein PSPTO_1500	**1.37**
PSPTO_0641	*rplF*, 50S ribosomal protein L6	**1.36**
PSPTO_5614	*rnpA*, ribonuclease P protein component	**1.36**
PSPTO_0806	*ileS*, isoleucyl-tRNA synthetase	**1.36**
PSPTO_1535	*tsf*, translation elongation factor Ts	**1.35**
PSPTO_4499	hypothetical protein PSPTO_4499	**1.34**
PSPTO_4804	*miaB*, tRNA-i(6)A37 modification enzyme MiaB	**1.34**
PSPTO_4933	*rpsF*, 30S ribosomal protein S6	**1.33**
PSPTO_0544	*cca*, tRNA nucleotidyltransferase	**1.32**
PSPTO_0643	*rpsE*, 30S ribosomal protein S5	**1.32**

PSPTO_4158	*rnt*, ribonuclease T	**1.31**
PSPTO_1811	Sua5/YciO/YrdC/YwlC family protein	**1.29**
PSPTO_0626	*rplC*, 50S ribosomal protein L3	**1.28**
PSPTO_0652	*rplQ*, 50S ribosomal protein L17	**1.24**
PSPTO_3981	*aspS*, aspartyl-tRNA synthetase	**1.2**
PSPTO_2311	23S rRNA m(2)G2445 methyltransferase	**1.19**
PSPTO_0642	*rplR*, 50S ribosomal protein L18	**1.19**
PSPTO_0650	*rpsD*, 30S ribosomal protein S4	**1.18**
PSPTO_4654	*trmA*, tRNA (uracil-5-)-methyltransferase	**1.17**
PSPTO_1815	*rluB*, ribosomal large subunit pseudouridine synthase B	**1.15**
PSPTO_4247	*rsuA*, ribosomal small subunit pseudouridine synthase A	**1.12**
PSPTO_1765	*efp*, translation elongation factor P	**1.12**
PSPTO_0629	*rplB*, 50S ribosomal protein L2	**1.11**
PSPTO_1753	hypothetical protein PSPTO_1753	**1.11**
PSPTO_0102	endoribonuclease L-PSP family protein	**1.1**
PSPTO_3988	*proS*, prolyl-tRNA synthetase	**1.1**
PSPTO_2166	*gltX*, glutamyl-tRNA synthetase	**1.09**
PSPTO_0644	*rpmD*, 50S ribosomal protein L30	**1.09**
PSPTO_0627	*rplD*, 50S ribosomal protein L4	**1.08**
PSPTO_1750	*rpsA*, 30S ribosomal protein S1	**1.06**
PSPTO_4841	translation initiation factor Sui1	**1.05**
PSPTO_0967	glutamyl-Q tRNA(Asp) synthetase	**1.04**
PSPTO_0178	*fmt*, methionyl-tRNA formyltransferase	**1**
PSPTO_2964	hypothetical protein PSPTO_2964	**-1.03**
PSPTO_4490	*infB*, translation initiation factor IF-2	**-1.09**
PSPTO_5387	stability cassette protein	**-1.12**
PSPTO_0551	*ksgA*, dimethyladenosine transferase	**-1.31**
PSPTO_4489	*rbfA*, ribosome-binding factor A	**-1.72**
PSPTO_2421	*amdA*, amidase	**-2.06**
PSPTO_2310	*rmf,* ribosome modulation factor-related protein	**-2.74**
PSPTO_0836	hypothetical protein PSPTO_0836	**-3.58**
**RNA processing and modification**		
PSPTO_4950	*orn*, oligoribonuclease	**1.3**
**Transcription**		
PSPTO_1768	MarR family transcriptional regulator	**3.27**
PSPTO_0327	S1 RNA binding domain-containing protein	**2.79**
PSPTO_4180	*metR*, transcriptional activator MetR	**2.51**
PSPTO_0689	*nrdR*, hypothetical protein PSPTO_0689	**2.44**
PSPTO_3510	*lexA-2*, LexA repressor	**2.34**
PSPTO_5267	zinc uptake regulation protein	**2.32**
PSPTO_0537	*rpoD*, RNA polymerase sigma-70 factor	**2.17**
PSPTO_1216	LysR family transcriptional regulator	**2.14**
PSPTO_2444	GNAT family acetyltransferase	**1.96**
PSPTO_3021	LysR family transcriptional regulator	**1.86**
PSPTO_5638	hypothetical protein PSPTO_5638	**1.8**
PSPTO_1857	AraC family transcriptional regulator	**1.78**
PSPTO_4755	hypothetical protein PSPTO_4755	**1.77**
PSPTO_3822	TetR family transcriptional regulator	**1.72**
PSPTO_2808	LysR family transcriptional regulator	**1.71**

PSPTO_1336	ArsR family transcriptional regulator	**1.7**
PSPTO_0074	*rpoZ*, DNA-directed RNA polymerase subunit omega	**1.69**
PSPTO_1733	*bolA*, bolA protein	**1.64**
PSPTO_1158	TetR family transcriptional regulator	**1.62**
PSPTO_0749	heavy metal-dependent transcriptional regulator	**1.6**
PSPTO_2404	ArsR family transcriptional regulator	**1.49**
PSPTO_4132	repressor protein c2	**1.46**
PSPTO_4104	ATP-dependent helicase HepA	**1.45**
PSPTO_0362	DeoR family transcriptional regulator	**1.44**
PSPTO_0430	*rpoH*, RNA polymerase sigma-32 factor	**1.43**
PSPTO_1299	*hexR*, Hex regulon repressor	**1.42**
PSPTO_0096	GntR family transcriptional regulator/aminotransferase, class I	**1.41**
PSPTO_3162	hypothetical protein PSPTO_3162	**1.36**
PSPTO_4920	LysR family transcriptional regulator	**1.34**
PSPTO_4550	GntR family transcriptional regulator	**1.34**
PSPTO_3698	TetR family transcriptional regulator	**1.34**
PSPTO_3046	sigma-54 dependent transcriptional regulator	**1.34**
PSPTO_2533	GNAT family acetyltransferase	**1.33**
PSPTO_4523	AsnC family transcriptional regulator	**1.33**
PSPTO_3040	LysR family transcriptional regulator	**1.27**
PSPTO_4010	repressor protein cI	**1.24**
PSPTO_3161	protein kinase	**1.23**
PSPTO_3773	GntR family transcriptional regulator	**1.22**
PSPTO_2265	*greB-1*, transcription elongation factor GreB	**1.2**
PSPTO_0614	*nusG*, transcription antitermination protein NusG	**1.2**
PSPTO_1422	rrf2 family protein	**1.19**
PSPTO_3861	MarR family transcriptional regulator	**1.18**
PSPTO_0134	algQ, transcriptional regulator AlgQ	**1.13**
PSPTO_3319	TetR family transcriptional regulator	**1.12**
PSPTO_2172	LysR family transcriptional regulator	**1.11**
PSPTO_0212	DNA-binding transcriptional regulator LysR	**1.09**
PSPTO_5242	*rho*, transcription termination factor Rho	**1.08**
PSPTO_1813	hypothetical protein PSPTO_1813	**1.08**
PSPTO_3017	LacI family transcriptional regulator	**1.07**
PSPTO_5311	TetR family transcriptional regulator	**1.06**
PSPTO_3076	TetR family transcriptional regulator	**1.06**
PSPTO_3617	MarR family transcriptional regulator	**1.06**
PSPTO_2945	MarR family transcriptional regulator	**1.04**
PSPTO_3980	hypothetical protein PSPTO_3980	**1.04**
PSPTO_1704	NAD-dependent deacetylase	**1.03**
PSPTO_2671	GntR family transcriptional regulator	**1.03**
PSPTO_0756	transcriptional regulator	**1.01**
PSPTO_2549	sigma-54 dependent transcriptional regulator	**-1.04**
PSPTO_1502	TetR family transcriptional regulator	**-1.05**
PSPTO_1645	MarR family transcriptional regulator	**-1.12**
PSPTO_2951	sigma-54 dependent transcriptional regulator	**-1.14**
PSPTO_4262	TetR family transcriptional regulator	**-1.15**
PSPTO_2370	*rbsR*, ribose operon repressor	**-1.16**
PSPTO_4528	AraC family transcriptional regulator	**-1.17**

PSPTO_2780	IclR family transcriptional regulator	**-1.18**
PSPTO_3741	sigma-54 dependent transcriptional regulator	**-1.19**
PSPTO_2827	hypothetical protein PSPTO_2827, partial	**-1.19**
PSPTO_0465	AraC family transcriptional regulator	**-1.23**
PSPTO_0619	*rpoB*, DNA-directed RNA polymerase subunit beta	**-1.25**
PSPTO_3355	cold shock domain family protein	**-1.26**
PSPTO_3056	AraC family transcriptional regulator	**-1.26**
PSPTO_0157	*trpI*, trpBA operon transcriptional activator	**-1.27**
PSPTO_3549	*aefR*, transcriptional regulator AefR	**-1.3**
PSPTO_2828	*syrR*, transcriptional regulator SyrR	**-1.35**
PSPTO_4302	TetR family transcriptional regulator	**-1.36**
PSPTO_2751	LysR family transcriptional regulator	**-1.38**
PSPTO_1286	RNA polymerase sigma-70 family protein	**-1.4**
PSPTO_4491	*nusA*, N utilization substance protein A	**-1.42**
PSPTO_2833	LuxR family transcriptional regulator	**-1.44**
PSPTO_4224	*rpoE*, RNA polymerase sigma-24 factor	**-1.45**
PSPTO_0032	hypothetical protein PSPTO_0032	**-1.45**
PSPTO_0620	*rpoC*, DNA-directed RNA polymerase subunit beta\'	**-1.52**
PSPTO_3025	DNA-binding protein	**-1.55**
PSPTO_3540	LysR family transcriptional regulator	**-1.69**
PSPTO_5424	sigma-54 dependent transcriptional regulator	**-1.74**
PSPTO_0033	ParB family protein	**-2.74**
PSPTO_4775	GntR family transcriptional regulator/aminotransferase, class I	**-2.79**
PSPTO_3467	sigma-54 dependent transcriptional regulator	**-2.86**
PSPTO_3086	transcriptional regulator	**-4.13**
**Replication, recombination and repair**		
PSPTO_1775	ATP-dependent RNA helicase, DEAD box family	**2.98**
PSPTO_3638	*recR*, recombination protein RecR	**2.48**
PSPTO_0470	exonuclease	**2.27**
PSPTO_4120	DinG family ATP-dependent helicase	**2.16**
PSPTO_5007	*dbpA*, ATP-independent RNA helicase DbpA	**2.15**
PSPTO_4058	*mutS*, DNA mismatch repair protein MutS	**2.04**
PSPTO_4664	ATP-dependent RNA helicase rhlE	**2.01**
PSPTO_5070	ATP-dependent RNA helicase rhlE	**1.95**
PSPTO_2101	*mfd*, transcription-repair coupling factor	**1.94**
PSPTO_4181	mutT/nudix family protein	**1.91**
PSPTO_0700	*xseB*, exodeoxyribonuclease VII small subunit	**1.9**
PSPTO_4830	3-methyladenine DNA glycosylase	**1.86**
PSPTO_1587	*srmB*, ATP-dependent RNA helicase SrmB	**1.86**
PSPTO_1745	*gyrA*, DNA gyrase subunit A	**1.85**
PSPTO_2255	TatD family hydrolase	**1.83**
PSPTO_4033	*recA*, recA protein	**1.69**
PSPTO_3897	recombination associated protein rdgC	**1.68**
PSPTO_3711	*dnaQ*, DNA polymerase III subunit epsilon	**1.66**
PSPTO_5137	*priA*, primosomal protein N`	**1.65**
PSPTO_2478	*topB*, DNA topoisomerase III	**1.62**
PSPTO_3712	*rnhA*, ribonuclease HI	**1.62**
PSPTO_1703	DNA replication initiation factor	**1.6**
PSPTO_0113	*rep*, ATP-dependent DNA helicase Rep	**1.59**

PSPTO_4773	ATP-dependent helicase, N-terminal domain protein, partial	**1.52**
PSPTO_1446	*xseA*, exodeoxyribonuclease VII large subunit	**1.48**
PSPTO_3220	ISPsy5, transposase	**1.48**
PSPTO_2747	*xthA*, exodeoxyribonuclease III	**1.47**
PSPTO_0079	exodeoxyribonuclease III	**1.45**
PSPTO_4507	*recN*, DNA repair protein RecN	**1.39**
PSPTO_1418	ISPsy6, transposase	**1.27**
PSPTO_5516	*uvrD*, DNA helicase II	**1.26**
PSPTO_4665	exonuclease	**1.26**
PSPTO_4236	*ung*, uracil-DNA glycosylase	**1.24**
PSPTO_3161	protein kinase	**1.23**
PSPTO_4735	ATP-dependent helicase HrpB	**1.23**
PSPTO_5443	ISPsy5, transposase	**1.21**
PSPTO_1439	ISPsy6, transposase	**1.21**
PSPTO_5304	ISPsy5, transposase	**1.17**
PSPTO_1488	*recJ*, single-stranded-DNA-specific exonuclease RecJ	**1.13**
PSPTO_4419	lipoprotein	**1.13**
PSPTO_3221	ISPsy5, Orf1	**1.09**
PSPTO_0344	*polI*, DNA polymerase I	**1.07**
PSPTO_3734	ISPsy6, transposase	**1.06**
PSPTO_0183	*tag*, DNA-3-methyladenine glycosidase I	**1.02**
PSPTO_0414	*mutM*, formamidopyrimidine-DNA glycosylase	**1**
PSPTO_4751	UvrD/REP helicase family protein	**-1.01**
PSPTO_5472	HU family DNA-binding protein	**-1.07**
PSPTO_4748	site-specific recombinase, phage integrase family	**-1.07**
PSPTO_0778	*recD*, exodeoxyribonuclease V subunit alpha	**-1.09**
PSPTO_2857	site-specific recombinase, phage integrase family	**-1.09**
PSPTO_3357	mutT/nudix family protein	**-1.11**
PSPTO_2624	hypothetical protein PSPTO_2624	**-1.15**
PSPTO_3656	*ligA*, NAD-dependent DNA ligase LigA	**-1.16**
PSPTO_3465	KU domain protein	**-1.17**
PSPTO_5629	insertion sequence	**-1.17**
PSPTO_2860	helicase domain-containing protein	**-1.19**
PSPTO_4397	mutT/nudix family protein	**-1.22**
PSPTO_3427	C-5 cytosine-specific DNA methylase family protein	**-1.29**
PSPTO_2856	site-specific recombinase, phage integrase family	**-1.33**
PSPTO_1407	ISPssy transposase or derivative	**-1.37**
PSPTO_1270	*holC*, DNA polymerase III subunit chi	**-1.45**
PSPTO_0028	transposase	**-1.46**
PSPTO_0587	site-specific recombinase, phage integrase family	**-1.48**
PSPTO_2388	ISPsy4, transposition helper protein	**-1.49**
PSPTO_0037	helicase domain-containing protein	**-1.51**
PSPTO_3326	group II intron, maturase	**-1.58**
PSPTO_4604	site-specific recombinase, phage integrase family	**-1.59**
PSPTO_2976	DNA topoisomerase, type I	**-1.62**
PSPTO_0047	UvrD/REP helicase family protein	**-1.76**
PSPTO_3930	retron reverse transcriptase	**-2.15**
**Cell cycle control, cell division, chromosome partitioning**		
PSPTO_3837	*maf-1*, maf protein	**2.17**

PSPTO_3511	*sulA*, cell division inhibitor	**1.75**
PSPTO_5139	hypothetical protein PSPTO_5139	**1.72**
PSPTO_1684	hypothetical protein PSPTO_1684	**1.37**
PSPTO_0855	ParA family protein	**1.22**
PSPTO_3873	*minD*, septum site-determining protein MinD	**1.18**
PSPTO_3874	*minE*, cell division topological specificity factor MinE	**1.06**
PSPTO_3872	*minC*, septum site-determining protein MinC	**1.03**
PSPTO_1555	hypothetical protein PSPTO_1555	**-1.12**
PSPTO_5387	stability cassette protein	**-1.12**
PSPTO_4403	cell division protein FtsZ	**-2.33**
**Defense mechanisms**		
PSPTO_2110	*lolD*, lipoprotein releasing system, ATP-binding protein LolD	**1.94**
PSPTO_4984	*msbA*, lipid A ABC transporter, ATP-binding/permease protein	**1.82**
PSPTO_4119	*estC*, carboxylesterase	**1.64**
PSPTO_3172	membrane protein	**1.52**
PSPTO_0951	AmpE protein	**1.35**
PSPTO_0681	permease	**1.25**
PSPTO_3855	ABC transporter ATP-binding protein	**1.21**
PSPTO_3141	undecaprenyl pyrophosphate phosphatase	**1.15**
PSPTO_0950	*ampD*, N-acetyl-anhydromuranmyl-L-alanine amidase	**1.06**
PSPTO_1089	type I restriction-modification enzyme, R subunit	**-1.03**
PSPTO_0005	type I restriction-modification system, M subunit	**-1.06**
PSPTO_0008	HsdR family type I site-specific deoxyribonuclease	**-1.12**
PSPTO_2832	*syfD*, syringafactin efflux protein SyfD	**-1.16**
PSPTO_1087	type I restriction-modification system subunit S	**-1.16**
PSPTO_1653	hypothetical protein PSPTO_1653	**-1.42**
PSPTO_0285	hypothetical protein PSPTO_0285	**-1.43**
PSPTO_4303	RND family efflux transporter MFP subunit	**-1.45**
PSPTO_2831	*syfC*, syringafactin efflux protein SyfC	**-1.46**
PSPTO_0011	hypothetical protein PSPTO_0011	**-1.47**
PSPTO_0006	type I restriction-modification system, S subunit, EcoA family	**-1.47**
PSPTO_2428	multidrug resistance protein NorM	**-1.51**
PSPTO_0370	MATE efflux family protein	**-1.55**
PSPTO_2755	AcrB/AcrD/AcrF family protein	**-1.57**
PSPTO_2875	ABC transporter ATP-binding protein	**-1.73**
PSPTO_4304	*saxB*, isothiocyanate resistance protein SaxB; isochorismatase family	**-2.16**
PSPTO_2603	ABC transporter ATP-binding protein/permease	**-2.54**
PSPTO_2604	ABC transporter ATP-binding protein/permease	**-2.57**
PSPTO_1654	hypothetical protein PSPTO_1654	**-2.79**
**Signal transduction mechanisms**		
PSPTO_0471	nucleotidyltransferase	**3.55**
PSPTO_0969	*dksA*, dnaK suppressor protein	**3.52**
PSPTO_4638	*cstA*, carbon starvation protein CstA	**2.07**
PSPTO_0306	sensory box/GGDEF domain/EAL domain-containing protein	**2.04**
PSPTO_3900	sensory box histidine kinase/response regulator	**2.04**
PSPTO_0114	GGDEF domain/EAL domain protein	**1.7**
PSPTO_2118	anti-anti-sigma factor	**1.7**
PSPTO_5398	sensor histidine kinase	**1.65**
PSPTO_2117	response regulator	**1.61**

PSPTO_4027	LuxR family DNA-binding response regulator	**1.57**
PSPTO_0536	sensory box/GGDEF domain/EAL domain-containing protein	**1.54**
PSPTO_4896	sensor histidine kinase	**1.42**
PSPTO_4374	*colR*, DNA-binding response regulator ColR	**1.35**
PSPTO_2245	*kdpD*, sensor protein KdpD	**1.34**
PSPTO_2358	hypothetical protein PSPTO_2358	**1.34**
PSPTO_0897	LuxR family DNA-binding response regulator	**1.29**
PSPTO_3566	carbon storage regulator	**1.24**
PSPTO_3161	protein kinase	**1.23**
PSPTO_4554	sensor histidine kinase	**1.2**
PSPTO_1629	*csrA-1*, carbon storage regulator	**1.15**
PSPTO_3520	universal stress protein family	**1.11**
PSPTO_4373	*colS*, sensor histidine kinase ColS	**1.1**
PSPTO_0965	sensor histidine kinase	**1.1**
PSPTO_2215	*sixA*, phosphohistidine phosphatase SixA	**1.09**
PSPTO_2223	DNA-binding response regulator	**1.03**
PSPTO_1803	sensor histidine kinase	**1.01**
PSPTO_3603	DNA-binding heavy metal response regulator	**1.01**
PSPTO_0361	*typA*, GTP-binding protein TypA	**1**
PSPTO_4222	*mucB*, sigma factor algU regulatory protein MucB	**-1.01**
PSPTO_3246	hypothetical protein PSPTO_3246	**-1.03**
PSPTO_4293	sensory box DNA/response regulator	**-1.11**
PSPTO_2642	sensor histidine kinase	**-1.14**
PSPTO_2896	sensory box histidine kinase/response regulator	**-1.18**
PSPTO_4223	*mucA*, sigma factor algU negative regulatory protein MucA	**-1.2**
PSPTO_0915	*cheY-1*, chemotaxis protein CheY	**-1.21**
PSPTO_3699	methyl-accepting chemotaxis protein	**-1.21**
PSPTO_0339	diguanylate cyclase	**-1.22**
PSPTO_2591	diguanylate cyclase	**-1.27**
PSPTO_1348	sensory box/GGDEF domain/EAL domain-containing protein	**-1.27**
PSPTO_2131	sensor histidine kinase	**-1.33**
PSPTO_4365	GGDEF domain/EAL domain protein	**-1.34**
PSPTO_4784	diguanylate cyclase	**-1.34**
PSPTO_1278	sensory box protein/response regulator	**-1.35**
PSPTO_2128	response regulator	**-1.4**
PSPTO_0406	sensory box/GGDEF domain/EAL domain-containing protein	**-1.42**
PSPTO_2448	methyl-accepting chemotaxis protein	**-1.52**
PSPTO_0913	*cheA-1*, chemotaxis sensor histidine kinase CheA	**-1.56**
PSPTO_4079	sensor histidine kinase/response regulator	**-1.58**
PSPTO_0910	*cheR-1*, chemotaxis protein methyltransferase CheR	**-1.65**
PSPTO_5416	serine/threonine protein kinase	**-1.71**
PSPTO_1844	*csrA-2*, carbon storage regulator	**-1.76**
PSPTO_4705	sensor histidine kinase CorS	**-1.88**
PSPTO_1870	sensory box histidine kinase/response regulator	**-1.93**
PSPTO_0911	*cheW-1*, chemotaxis protein CheW	**-1.97**
PSPTO_4371	*inaA*, inaA protein	**-2.1**
PSPTO_0912	methyl-accepting chemotaxis protein	**-2.11**
PSPTO_5422	FHA domain-containing protein	**-2.33**
PSPTO_2129	sensory box histidine kinase/response regulator	**-2.37**

PSPTO_1246	PhoH-like protein	**-2.47**
PSPTO_4704	DNA-binding response regulator CorR	**-2.68**
PSPTO_2130	LuxR family DNA-binding response regulator	**-2.73**
PSPTO_5417	serine/threonine phosphoprotein phosphatase	**-2.9**
PSPTO_0547	hypothetical protein PSPTO_0547	**-3.02**
PSPTO_4080	LuxR family DNA-binding response regulator	**-3.07**
**Cell wall/membrane/envelope biogenesis**		
PSPTO_0977	penicillin-binding protein	**2.53**
PSPTO_2109	*lolC*, lipoprotein releasing system transmembrane protein LolC	**2.38**
PSPTO_0431	*mtgA*, monofunctional biosynthetic peptidoglycan transglycosylase	**2.2**
PSPTO_2189	ompA family protein	**2.02**
PSPTO_4448	sugar isomerase	**1.99**
PSPTO_4220	*lepA*, GTP-binding protein LepA	**1.94**
PSPTO_5283	*lgt,* prolipoprotein diacylglyceryl transferase	**1.92**
PSPTO_4509	*omlA*, outer membrane lipoprotein OmlA	**1.7**
PSPTO_4182	D-alanine--D-alanine ligase	**1.69**
PSPTO_4813	lipoprotein	**1.66**
PSPTO_4945	N-acetylmuramoyl-L-alanine amidase family protein	**1.61**
PSPTO_4115	lipoprotein SlyB	**1.61**
PSPTO_5003	*rfaF*, ADP-heptose--LPS heptosyltransferase II	**1.6**
PSPTO_2056	membrane protein	**1.59**
PSPTO_3871	*htrB*, lipid A biosynthesis lauroyl acyltransferase	**1.59**
PSPTO_4470	rod shape-determining protein MreD	**1.58**
PSPTO_1330	glycosyl transferase family protein	**1.52**
PSPTO_0338	N-acetylmuramoyl-L-alanine amidase family protein	**1.51**
PSPTO_4392	mechanosensitive ion channel family protein	**1.49**
PSPTO_4471	*mreC*, rod shape-determining protein MreC	**1.49**
PSPTO_4985	toluene tolerance protein	**1.48**
PSPTO_0139	hypothetical protein PSPTO_0139	**1.45**
PSPTO_3113	*galU*, UTP-glucose-1-phosphate uridylyltransferase	**1.36**
PSPTO_3952	lipoprotein	**1.33**
PSPTO_1025	transglycosylase	**1.32**
PSPTO_0826	competence lipoprotein ComL	**1.31**
PSPTO_0182	lipid A biosynthesis lauroyl acyltransferase	**1.3**
PSPTO_5133	penicillin-binding protein	**1.29**
PSPTO_1067	glycosyl transferase family protein	**1.29**
PSPTO_2111	*lolE*, lipoprotein releasing system transmembrane protein LolE	**1.23**
PSPTO_4978	*kdtA*, 3-deoxy-D-manno-octulosonic-acid transferase	**1.2**
PSPTO_2509	penicillin-binding protein	**1.2**
PSPTO_4026	*dgkA*, diacylglycerol kinase	**1.18**
PSPTO_1574	mechanosensitive ion channel family protein	**1.18**
PSPTO_1705	NLP/P60 family protein	**1.16**
PSPTO_5341	hypothetical protein PSPTO_5341	**1.15**
PSPTO_3845	*lpxK*, tetraacyldisaccharide 4\'-kinase	**1.14**
PSPTO_5050	*metW*, metW protein	**1.13**
PSPTO_5193	RND family efflux transporter MFP subunit	**1.12**
PSPTO_1458	periplasmic binding domain/transglycosylase SLT domain fusion protein	**1.11**
PSPTO_2749	hypothetical protein PSPTO_2749	**1.1**
PSPTO_5002	*waaC*, lipopolysaccharide heptosyltransferase	**1.1**

PSPTO_4825	penicillin-binding protein	**1.05**
PSPTO_4414	penicillin-binding protein	**1.04**
PSPTO_2681	*pbpG*, penicillin-binding protein 7	**1.03**
PSPTO_1445	peptidase, M23/M37 family	**1.02**
PSPTO_1231	insecticidal toxin protein	**-1.02**
PSPTO_2554	hypothetical protein PSPTO_2554	**-1.05**
PSPTO_3328	*tliF,* ABC transporter permease	**-1.08**
PSPTO_4405	cell division protein FtsQ	**-1.08**
PSPTO_2343	outer membrane porin, OprD family	**-1.1**
PSPTO_0373	Rhs family protein	**-1.22**
PSPTO_3885	*prc*, tail-specific protease	**-1.24**
PSPTO_1506	ompA family protein	**-1.26**
PSPTO_3536	glycosyl transferase, group 1 family protein PslI	**-1.32**
PSPTO_4385	Rhs element Vgr protein	**-1.33**
PSPTO_2481	*tonB-2*, tonB protein	**-1.36**
PSPTO_4305	outer membrane efflux protein	**-1.37**
PSPTO_1071	glycosyl transferase family protein	**-1.42**
PSPTO_4344	insecticidal toxin protein	**-1.44**
PSPTO_4343	insecticidal toxin protein	**-1.45**
PSPTO_3531	lipoprotein PslD	**-1.49**
PSPTO_1947	glycosyl transferase family protein	**-1.58**
PSPTO_2011	autotransporter	**-1.62**
PSPTO_3535	glycosyl transferase, group 1 family protein PslH	**-1.69**
PSPTO_3533	glycosyl transferase, group 1 family protein PslF	**-1.73**
PSPTO_3290	outer membrane porin, OprD family	**-1.76**
PSPTO_4340	insecticidal toxin protein	**-1.79**
PSPTO_5415	Rhs element Vgr protein	**-1.86**
PSPTO_1632	GNAT family acetyltransferase	**-1.87**
PSPTO_3238	tonB protein	**-1.91**
PSPTO_4560	outer membrane porin, OprD family	**-1.96**
PSPTO_3532	exopolysaccharide biosynthesis protein PslE	**-1.97**
PSPTO_1243	*algD*, GDP-mannose 6-dehydrogenase AlgD	**-1.98**
PSPTO_1242	alginate biosynthesis protein Alg8	**-1.99**
PSPTO_2756	outer membrane efflux protein	**-2**
PSPTO_3648	*plcA1*, acid phosphatase	**-2.19**
PSPTO_1918	glycosyl transferase family protein	**-2.27**
PSPTO_1296	*oprB*, porin B	**-2.29**
PSPTO_3987	*oprD*, porin D	**-2.45**
PSPTO_1946	glycosyl transferase family protein	**-2.6**
PSPTO_5436	Rhs element Vgr protein	**-2.67**
PSPTO_1241	alginate biosynthesis protein Alg44	**-2.72**
PSPTO_5418	hypothetical protein PSPTO_5418	**-2.93**
PSPTO_1240	*algK*, alginate biosynthesis protein AlgK	**-3.36**
PSPTO_1239	*algE*, alginate biosynthesis protein AlgE	**-3.49**
PSPTO_1238	*algG*, alginate biosynthesis protein AlgG	**-3.61**
PSPTO_1237	*algX*, alginate biosynthesis protein AlgX	**-3.65**
PSPTO_1235	*algI*, alginate biosynthesis protein AlgI	**-4.1**
PSPTO_1234	*algJ*, alginate biosynthesis protein AlgJ	**-4.56**
PSPTO_1233	*algF*, alginate biosynthesis protein AlgF	**-4.74**

PSPTO_1232	*algA*, alginate biosynthesis protein AlgA	**-5.04**
**Cell motility**		
PSPTO_1432	*pilF*, type IV pilus biogenesis protein PilF	**1.29**
PSPTO_3237	methyl-accepting chemotaxis protein	**-1.02**
PSPTO_1944	*flgK*, flagellar hook-associated protein FlgK	**-1.11**
PSPTO_1935	*flgD*, basal-body rod modification protein FlgD	**-1.17**
PSPTO_1985	motB protein	**-1.28**
PSPTO_1951	*fliD*, flagellar hook-associated protein FliD	**-1.4**
PSPTO_0910	*cheR-1*, chemotaxis protein methyltransferase CheR	**-1.65**
PSPTO_0815	type IV pilus-associated protein	**-1.7**
PSPTO_0911	*cheW-1*, chemotaxis protein CheW	**-1.97**
PSPTO_1945	*flgL*, flagellar hook-associated protein FlgL	**-2.07**
PSPTO_0912	methyl-accepting chemotaxis protein	**-2.11**
PSPTO_0924	*pilD*, type IV pilus prepilin peptidase PilD	**-2.12**
PSPTO_1949	*fliC*, flagellin	**-2.32**
PSPTO_1936	*flgE-1*, flagellar hook protein FlgE	**-2.36**
PSPTO_1950	flagellin FlaG	**-2.52**
**Intracellular trafficking, secretion, and vesicular transport**		
PSPTO_4219	*lepB*, signal peptidase I	**1.75**
PSPTO_3975	tolQ protein	**1.67**
PSPTO_3632	*ccmD*, heme exporter protein CcmD	**1.66**
PSPTO_4493	*secG*, preprotein translocase subunit SecG	**1.54**
PSPTO_4852	hypothetical protein PSPTO_4852	**1.3**
PSPTO_1432	*pilF*, type IV pilus biogenesis protein PilF	**1.29**
PSPTO_1416	*secF,* protein-export membrane protein SecF	**1.25**
PSPTO_4869	membrane protein, MarC family	**1.2**
PSPTO_5612	inner membrane protein, 60 kDa	**1.18**
PSPTO_1415	*secD*, protein-export membrane protein SecD	**1.18**
PSPTO_0810	type IV pilus biogenesis protein	**1.18**
PSPTO_5324	*secB*, protein-export protein SecB	**1.14**
PSPTO_5125	hypothetical protein PSPTO_5125	**1.12**
PSPTO_4853	type II/IV secretion system protein	**1.09**
PSPTO_4400	*secA*, preprotein translocase subunit SecA	**1.02**
PSPTO_3312	general secretion pathway protein J	**-1.01**
PSPTO_3307	*gspD*, general secretion pathway protein D	**-1.18**
PSPTO_0811	pillin	**-1.29**
PSPTO_3229	filamentous hemagglutinin, intein-containing	**-1.59**
PSPTO_2011	autotransporter	**-1.62**
PSPTO_0816	type IV pilus biogenesis protein	**-2**
PSPTO_0924	*pilD*, type IV pilus prepilin peptidase PilD	**-2.12**
PSPTO_0925	*pilC*, type IV pilus biogenesis protein PilC	**-2.39**
**Posttranslational modification, protein turnover, chaperones**		
PSPTO_4515	*smpB*, SsrA-binding protein	**2.97**
PSPTO_3028	*ppiC-1*, peptidyl-prolyl cis-trans isomerase C	**2.59**
PSPTO_1479	*dsbC*, thiol:disulfide interchange protein DsbC	**2.13**
PSPTO_4424	*sspA*, stringent starvation protein A	**2.07**
PSPTO_2285	hypothetical protein PSPTO_2285	**1.99**
PSPTO_3631	*ccmE*, cytochrome c-type biogenesis protein CcmE	**1.96**
PSPTO_1471	membrane protein	**1.95**

PSPTO_3107	*ahpF*, alkyl hydroperoxide reductase subunit F	**1.86**
PSPTO_2529	FKBP-type peptidyl-prolyl cis-trans isomerase	**1.82**
PSPTO_3856	glutathione S-transferase family protein	**1.81**
PSPTO_3727	*tig*, trigger factor	**1.81**
PSPTO_4435	trypsin domain-containing protein	**1.8**
PSPTO_3259	peptidase, U32 family	**1.8**
PSPTO_0540	*gcp*, O-sialoglycoprotein endopeptidase	**1.78**
PSPTO_3634	*ccmB*, heme exporter protein CcmB	**1.73**
PSPTO_4504	*dnaJ*, dnaJ protein	**1.62**
PSPTO_4581	FKBP-type peptidyl-prolyl cis-trans isomerase	**1.51**
PSPTO_4640	*radA*, DNA repair protein RadA	**1.47**
PSPTO_3633	*ccmC*, heme exporter protein CcmC	**1.46**
PSPTO_4992	carbamoyltransferase family protein	**1.45**
PSPTO_2034	hypothetical protein PSPTO_2034	**1.44**
PSPTO_4161	glutaredoxin-like protein	**1.4**
PSPTO_3890	FKBP-type peptidyl-prolyl cis-trans isomerase	**1.39**
PSPTO_5243	*trx-2*, thioredoxin	**1.38**
PSPTO_0341	*dsbA*, thiol:disulfide interchange protein DsbA	**1.38**
PSPTO_1178	*trxB*, thioredoxin reductase	**1.3**
PSPTO_4858	*dsbD*, thiol:disulfide interchange protein DsbD	**1.3**
PSPTO_3034	*ppiC-2*, peptidyl-prolyl cis-trans isomerase C	**1.29**
PSPTO_3635	*ccmA*, heme exporter protein CcmA	**1.29**
PSPTO_1778	*htpX*, heat shock protein HtpX	**1.29**
PSPTO_4898	*yegD*, heat shock protein YegD	**1.28**
PSPTO_1689	lipoprotein	**1.23**
PSPTO_5325	glutaredoxin	**1.21**
PSPTO_3955	AhpC/Tsa family protein	**1.21**
PSPTO_3630	*ccmF*, cytochrome c-type biogenesis protein CcmF	**1.17**
PSPTO_5140	*hslV,* heat shock protein HslV	**1.13**
PSPTO_0135	FKBP-type peptidyl-prolyl cis-trans isomerase	**1.09**
PSPTO_3901	hypothetical protein PSPTO_3901	**1.01**
PSPTO_0238	*hslO*, chaperonin, 33 kDa	**1.01**
PSPTO_2548	clpB protein	**-1.01**
PSPTO_1365	glutathione S-transferase	**-1.02**
PSPTO_4210	*lon-2*, ATP-dependent protease La	**-1.02**
PSPTO_4940	*hflK*, hflK protein	**-1.15**
PSPTO_0606	hypothetical protein PSPTO_0606	**-1.2**
PSPTO_5317	antioxidant, AhpC/Tsa family	**-1.33**
PSPTO_4398	glutathione S-transferase family protein	**-1.34**
PSPTO_4295	DnaJ domain-containing protein	**-1.47**
PSPTO_4254	glutathione reductase	**-1.5**
PSPTO_1688	thioredoxin	**-1.59**
PSPTO_3560	GDA1/CD39 family protein	**-1.62**
PSPTO_4260	thioredoxin	**-1.73**
PSPTO_5425	clpB protein	**-1.89**
PSPTO_0924	*pilD*, type IV pilus prepilin peptidase PilD	**-2.12**
**Energy production and conversion**		
PSPTO_4059	*fdxA*, ferredoxin	**2.81**
PSPTO_0722	*ppa-1*, inorganic pyrophosphatase	**2.29**

PSPTO_1136	*mqo*, malate:quinone oxidoreductase	**2.28**
PSPTO_5605	*atpB*, F0F1 ATP synthase subunit A	**2.07**
PSPTO_3920	D-isomer specific 2-hydroxyacid dehydrogenase family protein	**1.99**
PSPTO_0239	*pckA*, phosphoenolpyruvate carboxykinase	**1.96**
PSPTO_4981	oxidoreductase, aldo/keto reductase family	**1.86**
PSPTO_2194	*gltA*, citrate synthase I	**1.75**
PSPTO_5606	*atpI*, F0F1 ATP synthase subunit I	**1.62**
PSPTO_1325	*cyoA*, cytochrome o ubiquinol oxidase subunit II	**1.51**
PSPTO_5293	oxidoreductase, FAD-binding protein	**1.48**
PSPTO_1802	nitroreductase family protein	**1.34**
PSPTO_2107	glycerophosphoryl diester phosphodiesterase	**1.29**
PSPTO_5598	*atpC*, F0F1 ATP synthase subunit epsilon	**1.28**
PSPTO_0416	ferredoxin	**1.28**
PSPTO_5603	*atpF,* F0F1 ATP synthase subunit B	**1.26**
PSPTO_3039	alcohol dehydrogenase	**1.22**
PSPTO_4928	hypothetical protein PSPTO_4928	**1.22**
PSPTO_1173	oxidoreductase FAD/FMN-binding protein	**1.19**
PSPTO_5602	*atpH*, F0F1 ATP synthase subunit delta	**1.17**
PSPTO_2182	oxidoreductase zinc-binding protein	**1.13**
PSPTO_5601	*atpA*, F0F1 ATP synthase subunit alpha	**1.07**
PSPTO_0754	alcohol dehydrogenase	**1.06**
PSPTO_2195	*sdhC*, succinate dehydrogenase, cytochrome b556 subunit	**1.03**
PSPTO_2070	cytochrome c2	**1.01**
PSPTO_3924	*sfcA*, malate dehydrogenase	**1.01**
PSPTO_2957	methanol dehydrogenase, NAD-dependent	**-1**
PSPTO_2288	*prpC*, 2-methylcitrate synthase	**-1.04**
PSPTO_2435	oxidoreductase, FAD-binding	**-1.07**
PSPTO_2519	oxidoreductase, FAD-binding protein	**-1.09**
PSPTO_4615	*ppa-2*, inorganic pyrophosphatase	**-1.11**
PSPTO_3075	oxidoreductase, aldo/keto reductase family	**-1.13**
PSPTO_0986	membrane protein	**-1.16**
PSPTO_4358	oxidoreductase FAD/FMN-binding protein	**-1.16**
PSPTO_3323	aldehyde dehydrogenase family protein	**-1.18**
PSPTO_5006	*aceF*, pyruvate dehydrogenase complex, E2 component, dihydrolipoamide acetyltransferase	**-1.23**
PSPTO_5186	DszA family monooxygenase	**-1.29**
PSPTO_4689	crotonyl-CoA reductase	**-1.33**
PSPTO_2510	*poxB*, pyruvate dehydrogenase	**-1.36**
PSPTO_2861	4Fe-4S binding protein, partial	**-1.37**
PSPTO_0396	NADH:flavin oxidoreductase/NADH oxidase family protein	**-1.38**
PSPTO_2697	oxidoreductase zinc-binding protein	**-1.38**
PSPTO_3372	*nuoI*, NADH dehydrogenase subunit I	**-1.41**
PSPTO_2289	aconitate hydratase	**-1.42**
PSPTO_4256	NADH:flavin oxidoreductase/NADH oxidase family protein	**-1.42**
PSPTO_2203	*sucD*, succinyl-CoA synthetase subunit alpha	**-1.43**
PSPTO_3559	malate synthase G	**-1.45**
PSPTO_5240	CDP-6-deoxy-delta-3,4-glucoseen reductase	**-1.46**
PSPTO_2676	*fdhA-2*, glutathione-independent formaldehyde dehydrogenase	**-1.47**
PSPTO_3601	hypothetical protein PSPTO_3601	**-1.49**
PSPTO_2202	*sucC*, succinyl-CoA synthetase subunit beta	**-1.5**

PSPTO_2943	*vdh*, vanillin dehydrogenase	**-1.58**
PSPTO_1489	xenobiotic reductase	**-1.62**
PSPTO_2680	*gabD-3*, succinate-semialdehyde dehydrogenase	**-1.8**
PSPTO_2016	*acnA*, aconitate hydratase 1	**-1.89**
PSPTO_3064	aldehyde dehydrogenase family protein	**-1.89**
PSPTO_5072	cytochrome b561	**-1.94**
PSPTO_2405	*xenA*, xenobiotic reductase A	**-2.79**
PSPTO_0062	citrate transporter	**-3.08**
PSPTO_3466	*ssuD*, alkanesulfonate monooxygenase	**-3.1**
PSPTO_0834	alcohol dehydrogenase	**-3.78**
**Carbohydrate transport and metabolism**		
PSPTO_2305	*lsc-2*, levansucrase	**2.7**
PSPTO_1419	*suhB*, inositol-1-monophosphatase	**2.58**
PSPTO_5289	*rpiA*, ribose 5-phosphate isomerase	**2.5**
PSPTO_0566	*rpe*, ribulose-phosphate 3-epimerase	**2.22**
PSPTO_4524	hypothetical protein PSPTO_4524	**1.82**
PSPTO_4494	*tpiA*, triosephosphate isomerase	**1.79**
PSPTO_2688	major facilitator family transporter	**1.69**
PSPTO_4913	hypothetical protein PSPTO_4913	**1.52**
PSPTO_0999	major facilitator family transporter	**1.49**
PSPTO_3278	hypothetical protein PSPTO_3278	**1.42**
PSPTO_1716	alpha-ribazole-5\'-phosphate phosphatase	**1.42**
PSPTO_5168	*fbp*, fructose-1,6-bisphosphatase	**1.41**
PSPTO_3986	hypothetical protein PSPTO_3986	**1.34**
PSPTO_3793	*ppnK*, inorganic polyphosphate/ATP-NAD kinase	**1.28**
PSPTO_1245	polysaccharide deacetylase family protein	**1.18**
PSPTO_1819	major facilitator superfamily transporter	**1.18**
PSPTO_4383	AmpG protein	**1.16**
PSPTO_4947	hypothetical protein PSPTO_4947	**1.11**
PSPTO_3507	*nagZ*, beta-hexosaminidase	**1.07**
PSPTO_1729	membrane protein	**1.05**
PSPTO_5327	*gpmA*, phosphoglycerate mutase	**1.04**
PSPTO_1612	major facilitator family transporter	**1.03**
PSPTO_0346	*thrB*, homoserine kinase	**1.02**
PSPTO_2292	*ppsA*, phosphoenolpyruvate synthase	**1.02**
PSPTO_0385	*tkt*, transketolase	**1.01**
PSPTO_2044	transporter	**1**
PSPTO_0563	polyamine ABC transporter periplasmic polyamine-binding protein	**-1.01**
PSPTO_2926	multidrug transporter	**-1.04**
PSPTO_2401	transketolase	**-1.07**
PSPTO_2761	alpha-amylase family protein	**-1.13**
PSPTO_3534	glycosyl hydrolase, family 5 PslG	**-1.14**
PSPTO_1707	L-sorbosone dehydrogenase	**-1.15**
PSPTO_3121	*zwf-2,* glucose-6-phosphate 1-dehydrogenase	**-1.15**
PSPTO_2369	*rbsC-1*, ribose ABC transporter permease	**-1.17**
PSPTO_3004	*xylG*, xylose transporter ATP-binding subunit	**-1.23**
PSPTO_1051	*dctP,* TRAP dicarboxylate transporter subunit DctP	**-1.23**
PSPTO_2731	periplasmic sugar-binding domain protein	**-1.26**
PSPTO_3737	ABC transporter permease	**-1.27**

PSPTO_1294	glucose ABC transporter permease	**-1.28**
PSPTO_3002	*xylA*, xylose isomerase	**-1.3**
PSPTO_2368	rbsA-1, ribose ABC transporter ATP-binding protein	**-1.31**
PSPTO_2470	senescence marker protein-30 family protein	**-1.34**
PSPTO_3496	*iolI*, iolI protein	**-1.39**
PSPTO_5219	MFS transporter, phthalate permease family	**-1.4**
PSPTO_1295	*gltK*, glucose ABC transporter ATP-binding protein	**-1.4**
PSPTO_5165	*glgP,* glycogen phosphorylase	**-1.41**
PSPTO_2400	ribose ABC transporter permease	**-1.46**
PSPTO_1298	hypothetical protein PSPTO_1298	**-1.47**
PSPTO_3003	*xylF,* D-xylose ABC transporter, periplasmic-D xylose binding protein	**-1.47**
PSPTO_1345	*gnl*, gluconolactonase	**-1.48**
PSPTO_2638	*abP*, L-arabinose ABC transporter periplasmic L-arabinose-binding protein	**-1.48**
PSPTO_2180	MFS transporter, phthalate permease family	**-1.54**
PSPTO_1293	glucose ABC transporter permease	**-1.54**
PSPTO_2762	*glgB*, 1,4-alpha-glucan-branching protein	**-1.54**
PSPTO_2765	hypothetical protein PSPTO_2765	**-1.56**
PSPTO_2667	spermidine/putrescine ABC transporter periplasmic spermidine/putrescine-binding protein	**-1.61**
PSPTO_3560	GDA1/CD39 family protein	**-1.62**
PSPTO_4296	metabolite-proton symporter	**-1.68**
PSPTO_3125	*glgA*, glycogen synthase	**-1.76**
PSPTO_1292	glucose ABC transporter, periplasmic glucose-binding protein	**-1.77**
PSPTO_5340	MFS permease-like protein	**-1.78**
PSPTO_2493	glycosyl hydrolase family protein	**-1.82**
PSPTO_4548	MFS transporter, phthalate permease family	**-1.87**
PSPTO_3735	ABC transporter periplasmic substrate-binding protein	**-1.93**
PSPTO_0838	major facilitator family transporter	**-1.95**
PSPTO_3740	ABC transporter ATP-binding protein	**-2.06**
PSPTO_3739	ABC transporter ATP-binding protein	**-2.08**
PSPTO_3126	alpha-amylase family protein	**-2.11**
PSPTO_3130	*glgX*, glycogen operon protein GlgX	**-2.13**
PSPTO_4306	*pcaT*, dicarboxylic acid transport protein	**-2.18**
PSPTO_2640	*araH*, L-arabinose transporter permease protein	**-2.18**
PSPTO_3128	glycosyl hydrolase family protein	**-2.2**
PSPTO_3018	hypothetical protein PSPTO_3018	**-2.36**
PSPTO_2399	ribose ABC transporter periplasmic ribose-binding protein	**-2.43**
PSPTO_3127	*malQ*, 4-alpha-glucanotransferase	**-2.5**
PSPTO_0202	membrane protein	**-2.61**
PSPTO_3493	iolH protein	**-2.8**
PSPTO_2473	periplasmic substrate-binding protein	**-3.23**
PSPTO_2701	fructokinase	**-3.26**
PSPTO_2703	*uxuB*, D-mannonate oxidoreductase	**-3.49**
PSPTO_2601	membrane protein	**-3.51**
PSPTO_1236	*algL*, alginate lyase	**-3.76**
PSPTO_2702	*xylB*, xylulokinase	**-3.78**
**Amino acid transport and metabolism**		
PSPTO_2206	*brnQ*, branched-chain amino acid transport system II carrier protein	**2.74**
PSPTO_1421	*cysE*, serine O-acetyltransferase	**2.58**
PSPTO_1760	threonine/serine transporter	**2.56**

PSPTO_4938	ATP phosphoribosyltransferase regulatory subunit	**2.53**
PSPTO_1817	aromatic amino acid permease	**2.46**
PSPTO_2055	*speE*, spermidine synthase	**2.28**
PSPTO_0272	*iscS-1*, cysteine desulfurase	**1.98**
PSPTO_3953	*dapA*, dihydrodipicolinate synthase	**1.91**
PSPTO_4503	*dapB*, dihydrodipicolinate reductase	**1.91**
PSPTO_4957	ACT domain protein/phosphoserine phosphatase SerB	**1.91**
PSPTO_1147	LysE family transporter	**1.91**
PSPTO_5294	*serA*, D-3-phosphoglycerate dehydrogenase	**1.91**
PSPTO_4632	*glyA-2*, serine hydroxymethyltransferase	**1.88**
PSPTO_0169	*aroE*, shikimate 5-dehydrogenase	**1.79**
PSPTO_3876	aspartyl aminopeptidase	**1.77**
PSPTO_0316	*gcvT-1*, glycine cleavage system T protein	**1.73**
PSPTO_5069	*metF*, 5,10-methylenetetrahydrofolate reductase	**1.71**
PSPTO_4829	*proA*, gamma-glutamyl phosphate reductase	**1.7**
PSPTO_0793	*selD*, selenide, water dikinase	**1.69**
PSPTO_1828	arginine/ornithine ABC transporter, permease protein	**1.65**
PSPTO_1772	phospho-2-dehydro-3-deoxyheptonate aldolase	**1.63**
PSPTO_1480	*hom*, homoserine dehydrogenase	**1.6**
PSPTO_2043	*aroC*, chorismate synthase	**1.59**
PSPTO_5049	homoserine O-acetyltransferase	**1.56**
PSPTO_0568	*trpE*, anthranilate synthase, component I	**1.52**
PSPTO_5047	*proC*, pyrroline-5-carboxylate reductase	**1.52**
PSPTO_1827	arginine/ornithine ABC transporter, permease protein	**1.51**
PSPTO_1041	*aroA*, 3-phosphoshikimate 1-carboxyvinyltransferase	**1.49**
PSPTO_5074	amine oxidase, flavin-containing protein	**1.43**
PSPTO_0125	*argH*, argininosuccinate lyase	**1.43**
PSPTO_1523	*dapE*, succinyl-diaminopimelate desuccinylase	**1.43**
PSPTO_5058	class I and II aminotransferase	**1.43**
PSPTO_3816	*trpF*, N-(5'-phosphoribosyl)anthranilate isomerase	**1.43**
PSPTO_1481	*thrC*, threonine synthase	**1.43**
PSPTO_3954	hypothetical protein PSPTO_3954	**1.42**
PSPTO_1779	class I and II aminotransferase	**1.41**
PSPTO_0225	*lsyA-2*, diaminopimelate decarboxylase	**1.4**
PSPTO_0598	*speD*, S-adenosylmethionine decarboxylase	**1.4**
PSPTO_0140	homoserine/homoserine lactone efflux protein	**1.39**
PSPTO_0774	beta-alanine--pyruvate aminotransferase	**1.36**
PSPTO_1639	bifunctional tRNA-methyltransferase/FAD-dependent oxidoreductase	**1.34**
PSPTO_1692	*cysM*, cysteine synthase B	**1.34**
PSPTO_4164	*argF*, ornithine carbamoyltransferase	**1.31**
PSPTO_3810	*metZ*, O-succinylhomoserine sulfhydrylase	**1.31**
PSPTO_0186	D,D-heptose 1,7-bisphosphate phosphatase	**1.28**
PSPTO_5126	*aroB*, 3-dehydroquinate synthase	**1.26**
PSPTO_1528	tetrahydrodipicolinate succinylase	**1.25**
PSPTO_5500	sodium/alanine transporter	**1.19**
PSPTO_0280	methionine aminopeptidase	**1.18**
PSPTO_5335	*hisA*, phosphoribosylformimino-5-aminoimidazole carboxamide ribotide isomerase	**1.17**
PSPTO_4155	*argG*, argininosuccinate synthase	**1.1**
PSPTO_5501	*ansA*, L-asparaginase I	**1.09**

PSPTO_4572	pyridoxal-dependent decarboxylase, pyridoxal binding domain protein	**1.06**
PSPTO_0970	class I and II aminotransferase	**1.06**
PSPTO_1729	membrane protein	**1.05**
PSPTO_5179	D-cysteine desulfhydrase	**1.04**
PSPTO_0317	*gcvH-1*, glycine cleavage system H protein	**1.03**
PSPTO_5334	*hisF*, Imidazole glycerol phosphate synthase cyclase subunit	**1.03**
PSPTO_4165	ABC transporter ATP-binding protein	**1.02**
PSPTO_1332	*ilvE*, branched-chain amino acid aminotransferase	**1.01**
PSPTO_2163	*aspC*, aspartate aminotransferase	**1**
PSPTO_2781	hypothetical protein PSPTO_2781	**-1.01**
PSPTO_2522	ABC transporter permease	**-1.05**
PSPTO_5307	putrescine ABC transporter periplasmic putrescine-binding protein	**-1.06**
PSPTO_3060	glycine betaine/L-proline ABC transporter ATP-binding protein	**-1.06**
PSPTO_3059	glycine betaine/L-proline ABC transporter permease	**-1.07**
PSPTO_2960	pyridoxal-phosphate dependent enzyme family/ornithine cyclodeaminase family protein	**-1.08**
PSPTO_4561	*dppA*, dipeptide ABC transporter substrate-binding protein	**-1.09**
PSPTO_2988	branched-chain amino acid ABC transporter permease	**-1.1**
PSPTO_2155	class V aminotransferase	**-1.11**
PSPTO_3717	ABC transporter substrate-binding protein	**-1.12**
PSPTO_3780	glutathionylspermidine synthase	**-1.13**
PSPTO_4112	high-affinity amino acid ABC transporter, ATP-binding protein	**-1.13**
PSPTO_5182	putative amino-acid ABC transporter ATP-binding protein YecC	**-1.13**
PSPTO_2427	serine hydroxymethyltransferase	**-1.14**
PSPTO_2451	*soxD-2*, sarcosine oxidase subunit delta	**-1.15**
PSPTO_3881	polyamine ABC transporter permease	**-1.16**
PSPTO_4915	high affinity branched-chain amino acid ABC transporter ATP-binding protein	**-1.17**
PSPTO_2915	glutamine ABC transporter, permease protein	**-1.17**
PSPTO_4577	glycine betaine/choline OpuC ABC transporter, periplasmic substrate-binding protein	**-1.19**
PSPTO_3058	glycine betaine transporter periplasmic subunit	**-1.19**
PSPTO_3084	glutamine/glutamate ABC transporter, permease protein	**-1.21**
PSPTO_1631	hypothetical protein PSPTO_1631	**-1.22**
PSPTO_5306	putrescine ABC transporter periplasmic putrescine-binding protein	**-1.26**
PSPTO_2812	peptide ABC transporter ATP-binding protein	**-1.26**
PSPTO_2913	glutamine ABC transporter ATP-binding protein	**-1.28**
PSPTO_4557	peptide ABC transporter periplasmic peptide-binding protein	**-1.33**
PSPTO_2665	spermidine/putrescine ABC transporter permease	**-1.34**
PSPTO_4916	high affinity branched-chain amino acid ABC transporter ATP-binding protein	**-1.36**
PSPTO_0457	*soxG-1*, sarcosine oxidase, gamma subunit	**-1.36**
PSPTO_0459	*soxD-1*, sarcosine oxidase subunit delta	**-1.37**
PSPTO_4917	*braE*, high-affinity branched-chain amino acid ABC transporter, permease protein BraE	**-1.42**
PSPTO_3252	dipeptide ABC transporter ATP-binding protein	**-1.43**
PSPTO_0524	peptidase, M20/M25/M40 family	**-1.43**
PSPTO_1622	glycine betaine-binding protein	**-1.44**
PSPTO_4919	high affinity branched-chain amino acid ABC transporter periplasmic amino acid-binding protein	**-1.45**
PSPTO_4918	*braD*, high-affinity branched-chain amino acid ABC transporter, permease protein BraD	**-1.45**
PSPTO_4559	dipeptide ABC transporter substrate-binding protein	**-1.48**
PSPTO_3251	dipeptide ABC transporter ATP-binding protein	**-1.49**

PSPTO_2504	efflux protein, LysE family	**-1.51**
PSPTO_0460	*soxB-1*, sarcosine oxidase, beta subunit	**-1.55**
PSPTO_1343	hypothetical protein PSPTO_1343	**-1.58**
PSPTO_0394	renal dipeptidase family protein	**-1.59**
PSPTO_1633	*asnB*, asparagine synthetase	**-1.59**
PSPTO_3085	glutamine/glutamate ABC transporter, permease protein	**-1.63**
PSPTO_1221	LysE family transporter	**-1.65**
PSPTO_4111	leucine/isoleucine/valine transporter ATP-binding subunit	**-1.69**
PSPTO_4136	amino acid ABC transporter substrate-binding protein	**-1.71**
PSPTO_3495	iolD protein	**-1.73**
PSPTO_0817	oxidoreductase, FAD-binding protein	**-1.75**
PSPTO_0203	cysteine synthase	**-1.78**
PSPTO_3063	dihydrodipicolinate synthase	**-1.85**
PSPTO_4110	leucine/isoleucine/valine transporter permease subunit	**-1.96**
PSPTO_2777	amino acid ABC transporter permease	**-2.04**
PSPTO_2775	amino acid ABC transporter substrate-binding protein	**-2.05**
PSPTO_0461	*glyA-1*, serine hydroxymethyltransferase	**-2.22**
PSPTO_2776	amino acid ABC transporter permease	**-2.23**
PSPTO_0458	*soxA-1*, sarcosine oxidase, alpha subunit	**-2.36**
PSPTO_1258	amino acid ABC transporter ATP-binding protein	**-2.48**
PSPTO_1255	amino acid ABC transporter substrate-binding protein	**-2.54**
PSPTO_1257	amino acid ABC transporter permease	**-2.56**
PSPTO_4558	dipeptide ABC transporter, periplasmic dipeptide-binding protein	**-2.61**
PSPTO_4109	high-affinity branched-chain amino acid ABC transporter permease	**-2.72**
PSPTO_1256	amino acid ABC transporter permease	**-2.77**
PSPTO_2430	pyridoxal-phosphate dependent enzyme family protein	**-3.28**
PSPTO_0873	amidinotransferase family protein	**-4.85**
**Nucleotide transport and metabolism**		
PSPTO_1700	*purM*,phosphoribosylformylglycinamidine cyclo-ligase	**2.81**
PSPTO_2028	*pyrF,* orotidine 5-phosphate decarboxylase	**2.7**
PSPTO_3750	hypothetical protein PSPTO_3750	**2.63**
PSPTO_4937	*purA*, adenylosuccinate synthetase	**2.31**
PSPTO_1509	*adk*, adenylate kinase	**2.19**
PSPTO_1699	*purN,* phosphoribosylglycinamide formyltransferase	**2.17**
PSPTO_2309	*pyrD*, dihydroorotate dehydrogenase	**1.96**
PSPTO_3360	*purB*, adenylosuccinate lyase	**1.85**
PSPTO_1468	*purT*, phosphoribosylglycinamide formyltransferase 2	**1.74**
PSPTO_1459	*purL*,phosphoribosylformylglycinamidine synthase	**1.65**
PSPTO_5282	*thyA*, thymidylate synthase	**1.58**
PSPTO_4314	*purU-3*, formyltetrahydrofolate deformylase	**1.58**
PSPTO_0080	*pyrE*, orotate phosphoribosyltransferase	**1.54**
PSPTO_1130	*upp*, uracil phosphoribosyltransferase	**1.52**
PSPTO_0075	*gmk*, guanylate kinase	**1.37**
PSPTO_1449	*guaB*, inosine 5\'-monophosphate dehydrogenase	**1.35**
PSPTO_3986	hypothetical protein PSPTO_3986	**1.34**
PSPTO_4502	*carA*, carbamoyl-phosphate synthase small subunit	**1.23**
PSPTO_5051	Ham1 protein	**1.21**
PSPTO_0112	*xpt*, xanthine phosphoribosyltransferase	**1.2**
PSPTO_4795	AMP nucleosidase	**1.19**

PSPTO_5249	*ppx*, exopolyphosphatase	**1.18**
PSPTO_4144	deoxycytidine triphosphate deaminase	**1.1**
PSPTO_1552	*pyrG*, CTP synthase	**1.05**
PSPTO_1990	*apt*, adenine phosphoribosyltransferase	**1.03**
PSPTO_3663	*guaD*, guanine aminohydrolase	**-1.01**
PSPTO_3043	5\'-nucleotidase	**-1.05**
PSPTO_4975	cytosine/purines uracil thiamine allantoin permease	**-1.15**
PSPTO_0043	cytidine/deoxycytidylate deaminase family protein	**-1.71**
PSPTO_2862	oxidoreductase, molybdopterin-binding subunit	**-1.82**
PSPTO_1671	*nrdA*, ribonucleotide-diphosphate reductase subunit alpha	**-2.03**
PSPTO_1694	*relA*, GTP pyrophosphokinase	**-4.02**
**Coenzyme transport and metabolism**		
PSPTO_3959	*nadA*, quinolinate synthetase	**3.44**
PSPTO_0796	*ispB*, octylprenyl diphosphate synthase	**2.81**
PSPTO_1708	*cobO*, cob(I)alamin adenosyltransferasehrpA	**2.69**
PSPTO_1709	cobyrinic acid a,c-diamide synthase	**2.62**
PSPTO_3733	*folD-2,* methylenetetrahydrofolate dehydrogenase/ methenyltetrahydrofolate cyclohydrolase	**2.49**
PSPTO_0691	*ribE*, riboflavin synthase subunit alpha	**2.34**
PSPTO_0170	*hemF,* coproporphyrinogen III oxidase	**2.3**
PSPTO_0961	*panB*, 3-methyl-2-oxobutanoate hydroxymethyltransferase	**2.19**
PSPTO_5227	5-formyltetrahydrofolate cyclo-ligase	**2.18**
PSPTO_5476	*ubiA*, 4-hydroxybenzoate octaprenyltransferase	**2.1**
PSPTO_4798	bifunctional hydroxy-methylpyrimidine kinase/ hydroxy-phosphomethylpyrimidine kinase	**2.09**
PSPTO_1710	nitroreductase family protein	**2.06**
PSPTO_0690	*ribD*, riboflavin biosynthesis protein RibD	**2.02**
PSPTO_1711	*cobD*, cobalamin biosynthesis protein CobD	**1.95**
PSPTO_2578	hypothetical protein PSPTO_2578	**1.93**
PSPTO_1525	ThiF family protein	**1.88**
PSPTO_4799	*thiE*, thiamine-phosphate pyrophosphorylase	**1.87**
PSPTO_3142	nicotinamide mononucleotide transporter PnuC	**1.87**
PSPTO_0692	*ribBA-1*, bifunctional 3,4-dihydroxy-2-butanone 4-phosphate synthase/GTP cyclohydrolase II-like protein	**1.86**
PSPTO_0960	*panC*, pantoate--beta-alanine ligase	**1.84**
PSPTO_3969	radical SAM domain-containing protein	**1.83**
PSPTO_4800	*hemL*, glutamate-1-semialdehyde-2,1-aminomutase	**1.82**
PSPTO_1713	*cobQ*, cobyric acid synthase	**1.82**
PSPTO_4878	*cobL*, precorrin-6Y C5,15-methyltransferase	**1.79**
PSPTO_5118	*hemE*, uroporphyrinogen decarboxylase	**1.72**
PSPTO_2019	*pdxB*, erythronate-4-phosphate dehydrogenase	**1.72**
PSPTO_0599	hypothetical protein PSPTO_0599	**1.71**
PSPTO_5068	*ahcY*, adenosylhomocysteinase	**1.71**
PSPTO_4876	*cobH*, precorrin-8X methylmutase	**1.66**
PSPTO_1715	*cobT*, nicotinate-nucleotide--dimethylbenzimidazole phosphoribosyltransferase	**1.66**
PSPTO_0434	*thiG*, thiazole biosynthesis protein ThiG	**1.64**
PSPTO_2035	*folE-2*, GTP cyclohydrolase I	**1.62**
PSPTO_0699	*ispA*, geranyltranstransferase	**1.61**
PSPTO_4879	*cbiD*, cobalamin biosynthesis protein CbiD	**1.6**
PSPTO_0360	*thiI*, thiamin biosynthesis protein ThiI	**1.57**
PSPTO_1714	*cobP*, cobinamide kinase/cobinamide phosphate guanylyltransferase	**1.56**

PSPTO_5475	chorismate-pyruvate lyase	**1.54**
PSPTO_5150	*ubiE*, ubiquinone/menaquinone biosynthesis methyltransferase UbiE	**1.52**
PSPTO_0437	*folA*, dihydrofolate reductase	**1.51**
PSPTO_0417	pantetheine-phosphate adenylyltransferase	**1.49**
PSPTO_0433	*thiS*, thiamine biosynthesis protein ThiS	**1.49**
PSPTO_4877	cobalamin biosynthesis protein CobG	**1.44**
PSPTO_5075	*bioA*, adenosylmethionine--8-amino-7-oxononanoate transaminase	**1.4**
PSPTO_0696	*ribA*, GTP cyclohydrolase II	**1.37**
PSPTO_4880	cobalt-precorrin-6x reductase	**1.35**
PSPTO_0805	*ribF*, riboflavin biosynthesis protein RibF	**1.34**
PSPTO_0962	*folK-2*, 2-amino-4-hydroxy-6-hydroxymethyldihydropteridine pyrophosphokinase	**1.27**
PSPTO_4875	*cobI*, precorrin-2 C(20)-methyltransferase	**1.26**
PSPTO_0948	*nadC*, nicotinate-nucleotide pyrophosphorylase	**1.25**
PSPTO_4922	*nadE*, NH(3)-dependent NAD(+) synthetase	**1.23**
PSPTO_0698	*dxs*, deoxyxylulose-5-phosphate synthase	**1.2**
PSPTO_0085	*coaBC*, phosphopantothenoylcysteine decarboxylase/phosphopantothenate--cysteine ligase	**1.2**
PSPTO_4818	*lipA*, lipoic acid synthetase	**1.18**
PSPTO_4828	*nadD*, nicotinate (nicotinamide) nucleotide adenylyltransferase	**1.11**
PSPTO_5035	*gshB*, glutathione synthetase	**1.1**
PSPTO_0128	*hemC*, porphobilinogen deaminase	**1.08**
PSPTO_0542	*folB-1*, dihydroneopterin aldolase	**1.06**
PSPTO_4225	*nadB*, L-aspartate oxidase	**1.05**
PSPTO_4921	*pncB*, nicotinate phosphoribosyltransferase	**1.05**
PSPTO_1108	*hemA*, glutamyl-tRNA reductase	**1.03**
PSPTO_2352	*moaB*, molybdenum cofactor biosynthesis protein B	**1.02**
PSPTO_1717	*cobS*, cobalamin (5\'-phosphate) synthase	**1.02**
PSPTO_3147	hypothetical protein PSPTO_3147	**-1**
PSPTO_1738	hypothetical protein PSPTO_1738	**-1.04**
PSPTO_3149	cobaltochelatase subunit CobN	**-1.07**
PSPTO_3148	magnesium chelatase, subunit ChlI	**-1.1**
PSPTO_0511	*pqqC*, coenzyme PQQ synthesis protein C	**-1.13**
PSPTO_3553	*hppD*, 4-hydroxyphenylpyruvate dioxygenase	**-1.29**
PSPTO_5240	CDP-6-deoxy-delta-3,4-glucoseen reductase	**-1.46**
PSPTO_5313	molybdenum-pterin binding domain-containing protein	**-2.12**
PSPTO_5086	*mdcB*, 2-(5'-triphosphoribosyl)-3'-dephosphocoenzyme-A synthase	**-2.41**
PSPTO_2595	isochorismate synthase	**-2.9**
PSPTO_4976	*thiC*, thiamin biosynthesis protein ThiC	**-3.11**
PSPTO_2105	thiamine biosynthesis lipoprotein	**-3.5**
PSPTO_0835	*ribD,* C-terminal domain protein, partial	**-3.5**
**Lipid transport and metabolism**		
PSPTO_1105	*ispE,* 4-diphosphocytidyl-2-C-methyl-D-erythritol kinase	**2.47**
PSPTO_1825	*acs*, acetyl-CoA synthetase	**2.46**
PSPTO_0305	fatty acid desaturase	**2.33**
PSPTO_1560	*ispF*, 2-C-methyl-D-erythritol 2,4-cyclodiphosphate synthase	**2.12**
PSPTO_0187	hdtS protein	**2.01**
PSPTO_4831	PAP2 superfamily protein/DedA family protein	**1.86**
PSPTO_0893	outer membrane protein P1	**1.75**
PSPTO_5092	acyltransferase family protein	**1.62**
PSPTO_1766	lipase	**1.52**

PSPTO_5094	acyl carrier protein	**1.49**
PSPTO_0984	*pssA-1*, CDP-diacylglycerol--serine O-phosphatidyltransferase	**1.44**
PSPTO_5093	acyl carrier protein	**1.43**
PSPTO_4660	*tesB*, acyl-CoA thioesterase II	**1.42**
PSPTO_3455	3-oxoacid CoA-transferase, subunit A family	**1.31**
PSPTO_3815	*accD*, acetyl-CoA carboxylase subunit beta	**1.29**
PSPTO_3136	azoreductase	**1.28**
PSPTO_2210	*fabB*, 3-oxoacyl-(acyl carrier protein) synthase I	**1.25**
PSPTO_3899	acyltransferase family protein	**1.19**
PSPTO_4097	*fadD-1*, long-chain-fatty-acid--CoA ligase	**1.19**
PSPTO_3831	*acpP*, acyl carrier protein	**1.17**
PSPTO_4513	hypothetical protein PSPTO_4513	**1.16**
PSPTO_3022	*pgsA*, CDP-diacylglycerol--glycerol-3-phosphate 3-phosphatidyltransferase	**1.14**
PSPTO_3463	*pssA-2*, CDP-diacylglycerol--serine O-phosphatidyltransferase	**1.1**
PSPTO_2823	hypothetical protein PSPTO_2823	**1.09**
PSPTO_2211	*fabA*, 3-hydroxydecanoyl-(acyl carrier protein) dehydratase	**1.05**
PSPTO_4094	3-oxoacyl-(acyl carrier protein) synthase III	**1.01**
PSPTO_3833	*fabD*, malonyl CoA-acyl carrier protein transacylase	**1.01**
PSPTO_4683	coronafacic acid beta-ketoacyl synthetase component	**-1.01**
PSPTO_4277	esterase/lipase/thioesterase family protein	**-1.02**
PSPTO_3163	enoyl-CoA hydratase/isomerase family protein	**-1.03**
PSPTO_3503	cardiolipin synthetase 2	**-1.06**
PSPTO_2940	thiolase family protein	**-1.08**
PSPTO_5185	acyl-CoA dehydrogenase family protein	**-1.12**
PSPTO_1790	acyl-CoA dehydrogenase family protein	**-1.13**
PSPTO_4781	hypothetical protein PSPTO_4781	**-1.2**
PSPTO_0743	3-ketoacyl-(acyl-carrier-protein) reductase	**-1.3**
PSPTO_0569	autotransporting lipase, GDSL family	**-1.43**
PSPTO_5081	malonyl CoA-acyl carrier protein transacylase	**-1.83**
PSPTO_5184	acyl-CoA dehydrogenase family protein	**-2.49**
PSPTO_5084	malonate decarboxylase subunit beta	**-2.75**
PSPTO_0200	hypothetical protein PSPTO_0200	**-3.1**
**Inorganic ion transport and metabolism**		
PSPTO_4160	*bfr,* bacterioferritin	**3.96**
PSPTO_0752	copZ protein	**3.41**
PSPTO_0750	copper-translocating P-type ATPase	**2.75**
PSPTO_0753	Bcr/CflA family multidrug resistance transporter	**2.45**
PSPTO_0998	alkylphosphonate utilization operon protein PhnA	**2.41**
PSPTO_5262	*metN-2*, DL-methionine transporter ATP-binding subunit	**2.32**
PSPTO_5261	*metI-2*, D-methionine ABC transporter permease	**2.19**
PSPTO_0791	phosphonate ABC transporter periplasmic phosphonate-binding protein	**2.18**
PSPTO_5268	*znuA*, zinc ABC transporter periplasmic zinc-binding protein	**2.12**
PSPTO_5266	*znuC*, zinc ABC transporter ATP-binding protein	**1.95**
PSPTO_1801	*trkH*, potassium uptake protein TrkH	**1.92**
PSPTO_3234	sulfate permease family protein	**1.91**
PSPTO_2018	sulfur transfer protein SirA	**1.91**
PSPTO_4979	multidrug resistance protein	**1.87**
PSPTO_5326	rhodanese-like domain-containing protein	**1.78**
PSPTO_0653	bacterioferritin	**1.7**

PSPTO_0763	*fecB*, iron(III) dicitrate transport system, periplasmic iron-binding protein FecB	**1.69**
PSPTO_0790	phosphonate ABC transporter, ATP-binding protein	**1.58**
PSPTO_4569	iron-uptake factor	**1.56**
PSPTO_2355	glutathione-regulated potassium-efflux system protein	**1.52**
PSPTO_5479	transporter	**1.5**
PSPTO_3593	sulfate permease family protein	**1.48**
PSPTO_0314	iron ABC transporter periplasmic iron-binding protein	**1.41**
PSPTO_0762	*fecC*, iron-dicitrate transporter permease subunit	**1.39**
PSPTO_0227	*cyaY,* cyaY protein	**1.36**
PSPTO_5023	sodium/hydrogen exchanger family protein	**1.33**
PSPTO_0760	iron(III) dicitrate transport system, ATP-binding protein FecE	**1.3**
PSPTO_5343	hypothetical protein PSPTO_5343	**1.27**
PSPTO_1846	*mgtE*, magnesium transporter	**1.22**
PSPTO_2463	TonB-dependent siderophore receptor	**1.15**
PSPTO_2634	cation ABC transporter permease	**1.14**
PSPTO_1529	arsC family protein	**1.13**
PSPTO_0761	*fecD*, iron(III) dicitrate transport system, permease protein FecD	**1.09**
PSPTO_5265	*znuB*, zinc ABC transporter permease	**1.08**
PSPTO_3256	iron ABC transporter, periplasmic iron-binding protein	**1.06**
PSPTO_5260	*metQ-2*, D-methionine-binding lipoprotein MetQ	**1.06**
PSPTO_1687	*arsC*, arsenate reductase	**1.05**
PSPTO_3993	*kup*, potassium uptake protein	**1.04**
PSPTO_2635	cation ABC transporter substrate-binding protein	**1.04**
PSPTO_4312	phosphate transporter family protein	**1.03**
PSPTO_5256	sulfate transporter family protein	**-1.01**
PSPTO_2141	cation ABC transporter substrate-binding protein	**-1.01**
PSPTO_4171	amino acid ABC transporter substrate-binding protein	**-1.01**
PSPTO_5255	*cynT*, carbonic anhydrase	**-1.02**
PSPTO_2846	TonB-dependent siderophore receptor	**-1.02**
PSPTO_0311	sulfate ABC transporter, ATP-binding protein CysA	**-1.03**
PSPTO_0564	polyamine ABC transporter permease	**-1.03**
PSPTO_1855	TonB-dependent receptor	**-1.04**
PSPTO_3599	hypothetical protein PSPTO_3599	**-1.05**
PSPTO_3031	peptide ABC transporter permease	**-1.05**
PSPTO_1204	regulatory protein	**-1.05**
PSPTO_0887	sugar ABC transporter permease	**-1.06**
PSPTO_0310	sulfate ABC transporter permease CysW	**-1.08**
PSPTO_3250	dipeptide ABC transporter, permease protein DppC	**-1.08**
PSPTO_5187	*metQ-1*, D-methionine-binding lipoprotein MetQ	**-1.11**
PSPTO_2605	TonB-dependent siderophore receptor	**-1.13**
PSPTO_2556	*phnD*, phosphonates ABC transporter periplasmic phosphonates-binding protein	**-1.16**
PSPTO_1206	TonB-dependent siderophore receptor	**-1.18**
PSPTO_0309	*cysT*, sulfate ABC transporter permease CysT	**-1.2**
PSPTO_2523	ABC transporter permease	**-1.25**
PSPTO_2812	peptide ABC transporter ATP-binding protein	**-1.26**
PSPTO_2140	cation ABC transporter ATP-binding protein	**-1.33**
PSPTO_3090	nickel ABC transporter, permease protein	**-1.36**
PSPTO_4363	*sodB*, superoxide dismutase, Fe	**-1.38**
PSPTO_1792	rhodanese-like domain-containing protein	**-1.41**

PSPTO_2517	sodium/hydrogen exchanger family protein	**-1.45**
PSPTO_3719	ABC transporter permease	**-1.49**
PSPTO_5216	lipoprotein, NLPA family	**-1.49**
PSPTO_2562	*phnJ*, phosphonate metabolism protein PhnJ	**-1.52**
PSPTO_2518	extracellular solute-binding domain protein	**-1.54**
PSPTO_2564	phosphonate ABC transporter ATPase	**-1.58**
PSPTO_5079	*madM*, malonate transporter subunit MadM	**-1.58**
PSPTO_5196	ABC transporter permease	**-1.58**
PSPTO_2563	phosphonate C-P lyase system protein PhnK	**-1.59**
PSPTO_2666	spermidine/putrescine ABC transporter permease	**-1.62**
PSPTO_1930	*cynS*, cyanate lyase	**-1.65**
PSPTO_0110	metallo-beta-lactamase superfamily protein	**-1.7**
PSPTO_2138	ABC transporter periplasmic substrate-binding protein	**-1.72**
PSPTO_2152	TonB-dependent siderophore receptor	**-1.82**
PSPTO_0308	*sbp*, sulfate-binding protein	**-1.83**
PSPTO_3878	ABC transporter substrate-binding protein	**-1.89**
PSPTO_5197	ABC transporter ATP-binding protein	**-1.9**
PSPTO_5195	ABC transporter substrate-binding protein	**-1.92**
PSPTO_4259	glutathione-regulated potassium-efflux system protein	**-1.93**
PSPTO_2706	mannitol ABC transporter permease	**-2.06**
PSPTO_5080	*madL*, malonate transporter subunit MadL	**-2.2**
PSPTO_5316	sulfonate ABC transporter periplasmic sulfonate-binding protein	**-2.23**
PSPTO_2398	ribose ABC transporter ATP-binding protein	**-2.26**
PSPTO_2639	L-arabinose transporter ATP-binding protein	**-2.36**
PSPTO_5314	aliphatic sulfonates transport ATP-binding subunit	**-2.45**
PSPTO_5315	*ssuC*, aliphatic sulfonates ABC transporter permease	**-2.46**
PSPTO_2705	mannitol ABC transporter permease	**-2.67**
PSPTO_2691	membrane protein, TerC family	**-2.88**
**Secondary metabolites biosynthesis, transport and catabolism**		
PSPTO_1735	hypothetical protein PSPTO_1735	**1.89**
PSPTO_3389	lysozyme	**1.6**
PSPTO_1456	*cumA*, multicopper oxidase	**1.58**
PSPTO_5190	hypothetical protein PSPTO_5190	**1.51**
PSPTO_5094	acyl carrier protein	**1.49**
PSPTO_2039	isochorismatase family protein	**1.49**
PSPTO_5093	acyl carrier protein	**1.43**
PSPTO_4177	2-hydroxychromene-2-carboxylate isomerase	**1.29**
PSPTO_4444	toluene tolerance protein	**1.26**
PSPTO_4212	methyltransferase	**1.23**
PSPTO_3138	3-oxoadipate enol-lactone hydrolase	**1.19**
PSPTO_2338	*pcaH*, protocatechuate 3,4-dioxygenase subunit beta	**1.17**
PSPTO_5292	fumarylacetoacetate hydrolase family protein	**1.14**
PSPTO_3332	alkaline metalloendoprotease	**1.08**
PSPTO_3713	hypothetical protein PSPTO_3713	**1.02**
PSPTO_4445	mce-like protein	**1.01**
PSPTO_1859	isothiocyanate resistance protein SaxB; isochorismatase family	**-1.01**
PSPTO_4683	coronafacic acid beta-ketoacyl synthetase component	**-1.01**
PSPTO_4257	4-oxalocrotonate tautomerase	**-1.02**
PSPTO_4709	coronamic acid synthetase CmaA	**-1.04**

PSPTO_2135	*pvsA*, pyoverdine chromophore precursor synthetase	**-1.15**
PSPTO_2941	*fcs*, feruloyl-CoA synthase	**-1.2**
PSPTO_3554	maleylacetoacetate isomerase	**-1.21**
PSPTO_2148	pyoverdine sidechain peptide synthetase II, D-Asp-L-Thr component	**-1.22**
PSPTO_2134	pyoverdine synthetase, thioesterase component	**-1.25**
PSPTO_4520	methyltransferase domain protein	**-1.25**
PSPTO_3051	2,4\'-dihydroxyacetophenone dioxygenase	**-1.27**
PSPTO_4261	isomerase	**-1.5**
PSPTO_2209	dienelactone hydrolase family protein	**-1.55**
PSPTO_5198	dioxygenase, TauD/TfdA family	**-1.59**
PSPTO_4712	coronamic acid synthetase, thioesterase component	**-1.76**
PSPTO_4699	non-ribosomal peptide synthetase, terminal component	**-1.77**
PSPTO_5082	phosphoribosyl-dephospho-CoA transferase	**-1.98**
PSPTO_4710	*cmaB*, coronamic acid synthetase CmaB	**-2.1**
PSPTO_5087	*mdcA*, malonate decarboxylase subunit alpha	**-2.21**
PSPTO_2597	yersiniabactin synthetase, salycilate ligase component	**-3.22**
PSPTO_2602	yersiniabactin non-ribosomal peptide synthetase	**-3.32**
PSPTO_2596	isochorismate pyruvate-lyase	**-3.4**
PSPTO_2429	capK domain protein	**-3.6**
PSPTO_2600	yersiniabactin polyketide/non-ribosomal peptide synthetase	**-3.63**
PSPTO_2598	yersiniabactin synthetase, thioesterase component	**-3.82**
PSPTO_2599	yersiniabactin synthetase, thiazolinyl reductase component	**-4.11**
**Funcation unknow**		
PSPTO_5228	hypothetical protein PSPTO_5228	**3.23**
PSPTO_1774	hypothetical protein PSPTO_1774	**2.98**
PSPTO_5015	Lyase	**2.79**
PSPTO_0513	pqqA, coenzyme PQQ synthesis protein A	**2.74**
PSPTO_0803	hypothetical protein PSPTO_0803	**2.74**
PSPTO_3361	hypothetical protein PSPTO_3361	**2.71**
PSPTO_3908	hypothetical protein PSPTO_3908	**2.67**
PSPTO_2687	hypothetical protein PSPTO_2687	**2.65**
PSPTO_5466	hypothetical protein PSPTO_5466	**2.64**
PSPTO_1104	*prsA*, ribose-phosphate pyrophosphokinase	**2.57**
PSPTO_0326	hypothetical protein PSPTO_0326	**2.5**
PSPTO_0724	DedA family protein	**2.46**
PSPTO_5264	lipoprotein	**2.44**
PSPTO_3175	hypothetical protein PSPTO_3175	**2.44**
PSPTO_1623	hypothetical protein PSPTO_1623	**2.4**
PSPTO_1683	peptidase, SprT family	**2.4**
PSPTO_3896	hypothetical protein PSPTO_3896	**2.4**
PSPTO_4030	hypothetical protein PSPTO_4030	**2.38**
PSPTO_3751	hypothetical protein PSPTO_3751	**2.38**
PSPTO_3123	hypothetical protein PSPTO_3123	**2.35**
PSPTO_3909	hypothetical protein PSPTO_3909	**2.35**
PSPTO_1734	putative rhodanese-related sulfurtransferase	**2.34**
PSPTO_4029	hypothetical protein PSPTO_4029	**2.34**
PSPTO_3067	hypothetical protein PSPTO_3067	**2.34**
PSPTO_0367	hypothetical protein PSPTO_0367	**2.33**
PSPTO_3173	hypothetical protein PSPTO_3173	**2.33**

PSPTO_1690	hypothetical protein PSPTO_1690	**2.32**
PSPTO_1816	hypothetical protein PSPTO_1816	**2.32**
PSPTO_0799	GTP-binding protein, GTP1/Obg family	**2.24**
PSPTO_4211	lipoprotein	**2.23**
PSPTO_1595	hypothetical protein PSPTO_1595	**2.2**
PSPTO_4227	hypothetical protein PSPTO_4227	**2.19**
PSPTO_2017	hypothetical protein PSPTO_2017	**2.16**
PSPTO_0412	hypothetical protein PSPTO_0412	**2.15**
PSPTO_1138	lipoprotein	**2.13**
PSPTO_4816	hypothetical protein PSPTO_4816	**2.13**
PSPTO_4481	hypothetical protein PSPTO_4481	**2.11**
PSPTO_2315	hypothetical protein PSPTO_2315	**2.05**
PSPTO_4484	hypothetical protein PSPTO_4484	**2.05**
PSPTO_0071	lipoprotein	**2.04**
PSPTO_4113	lipoprotein	**2.03**
PSPTO_3858	hypothetical protein PSPTO_3858	**2.03**
PSPTO_3910	hypothetical protein PSPTO_3910	**2**
PSPTO_4114	hypothetical protein PSPTO_4114	**1.98**
PSPTO_4007	hypothetical protein PSPTO_4007	**1.98**
PSPTO_3362	GNAT family acetyltransferase	**1.97**
PSPTO_4009	regulatory protein Cro	**1.97**
PSPTO_1642	hypothetical protein PSPTO_1642	**1.95**
PSPTO_2356	hypothetical protein PSPTO_2356	**1.95**
PSPTO_0792	hypothetical protein PSPTO_0792	**1.94**
PSPTO_1594	hypothetical protein PSPTO_1594	**1.93**
PSPTO_1272	membrane protein	**1.93**
PSPTO_4008	hypothetical protein PSPTO_4008	**1.93**
PSPTO_1624	sodium:solute symporter family protein	**1.92**
PSPTO_5059	hypothetical protein PSPTO_5059	**1.92**
PSPTO_2188	lipoprotein	**1.91**
PSPTO_4226	hypothetical protein PSPTO_4226	**1.88**
PSPTO_0404	hypothetical protein PSPTO_0404	**1.87**
PSPTO_3836	hypothetical protein PSPTO_3836	**1.85**
PSPTO_4668	membrane protein	**1.85**
PSPTO_1593	hypothetical protein PSPTO_1593	**1.85**
PSPTO_2264	hypothetical protein PSPTO_2264	**1.82**
PSPTO_4107	hypothetical protein PSPTO_4107	**1.82**
PSPTO_0138	ABC transporter ATP-binding protein	**1.82**
PSPTO_0567	*gph-1*, phosphoglycolate phosphatase	**1.81**
PSPTO_0078	hypothetical protein PSPTO_0078	**1.8**
PSPTO_0241	hypothetical protein PSPTO_0241	**1.78**
PSPTO_0798	*rpmA*, 50S ribosomal protein L27	**1.78**
PSPTO_5527	hypothetical protein PSPTO_5527	**1.76**
PSPTO_3521	ABC transporter ATP-binding protein	**1.74**
PSPTO_3333	membrane protein	**1.74**
PSPTO_4931	membrane protein	**1.72**
PSPTO_0991	hypothetical protein PSPTO_0991	**1.71**
PSPTO_0683	hypothetical protein PSPTO_0683	**1.71**
PSPTO_3921	hypothetical protein PSPTO_3921	**1.71**

PSPTO_3812	cvpA family protein	**1.7**
PSPTO_3907	hypothetical protein PSPTO_3907	**1.7**
PSPTO_3769	hypothetical protein PSPTO_3769	**1.7**
PSPTO_2527	hypothetical protein PSPTO_2527	**1.68**
PSPTO_1698	hypothetical protein PSPTO_1698	**1.67**
PSPTO_3419	C4-type zinc finger protein, DksA/TraR family	**1.67**
PSPTO_5149	hypothetical protein PSPTO_5149	**1.66**
PSPTO_0226	*lppL*, lipoprotein LppL	**1.66**
PSPTO_0076	hypothetical protein PSPTO_0076	**1.66**
PSPTO_1724	hypothetical protein PSPTO_1724	**1.66**
PSPTO_1614	hypothetical protein PSPTO_1614	**1.66**
PSPTO_4653	xanthine/uracil permease family protein	**1.65**
PSPTO_1728	hypothetical protein PSPTO_1728	**1.65**
PSPTO_4017	hypothetical protein PSPTO_4017	**1.65**
PSPTO_1526	*sufE,* sufE protein	**1.64**
PSPTO_5604	*atpE,* F0F1 ATP synthase subunit C	**1.64**
PSPTO_0968	ISPsy6, transposase	**1.64**
PSPTO_1244	hypothetical protein PSPTO_1244	**1.63**
PSPTO_1487	hypothetical protein PSPTO_1487	**1.63**
PSPTO_4779	hypothetical protein PSPTO_4779	**1.62**
PSPTO_5526	cobalamin synthesis protein/P47K family protein	**1.62**
PSPTO_3974	tolR protein	**1.62**
PSPTO_4121	hypothetical protein PSPTO_4121	**1.61**
PSPTO_1820	hypothetical protein PSPTO_1820	**1.6**
PSPTO_4637	hypothetical protein PSPTO_4637	**1.59**
PSPTO_5013	hypothetical protein PSPTO_5013	**1.58**
PSPTO_4472	*mreB*, rod shape-determining protein MreB	**1.57**
PSPTO_1430	*ndk*, nucleoside diphosphate kinase	**1.56**
PSPTO_0570	transcriptional regulator PrtN	**1.56**
PSPTO_2800	polysaccharide deacetylase family protein	**1.56**
PSPTO_4573	hypothetical protein PSPTO_4573	**1.56**
PSPTO_4022	hypothetical protein PSPTO_4022	**1.55**
PSPTO_0240	prevent-host-death family protein	**1.55**
PSPTO_0097	hypothetical protein PSPTO_0097	**1.53**
PSPTO_3985	hypothetical protein PSPTO_3985	**1.53**
PSPTO_4951	hypothetical protein PSPTO_4951	**1.52**
PSPTO_3420	hypothetical protein PSPTO_3420	**1.52**
PSPTO_4228	hypothetical protein PSPTO_4228	**1.51**
PSPTO_4401	hypothetical protein PSPTO_4401	**1.51**
PSPTO_5046	hypothetical protein PSPTO_5046	**1.5**
PSPTO_1467	hypothetical protein PSPTO_1467	**1.5**
PSPTO_0795	membrane protein	**1.5**
PSPTO_2049	hypothetical protein PSPTO_2049	**1.49**
PSPTO_0990	hypothetical protein PSPTO_0990	**1.48**
PSPTO_1732	hypothetical protein PSPTO_1732	**1.48**
PSPTO_0896	sensor histidine kinase/response regulator	**1.48**
PSPTO_3968	*exsB*, exsB protein	**1.47**
PSPTO_0828	hypothetical protein PSPTO_0828	**1.47**
PSPTO_0368	hypothetical protein PSPTO_0368	**1.46**

PSPTO_2608	Sco1/SenC family protein	**1.46**
PSPTO_0342	hypothetical protein PSPTO_0342	**1.46**
PSPTO_0121	membrane protein	**1.45**
PSPTO_2365	hypothetical protein PSPTO_2365	**1.45**
PSPTO_1140	hypothetical protein PSPTO_1140	**1.44**
PSPTO_1814	hypothetical protein PSPTO_1814	**1.44**
PSPTO_4901	membrane protein	**1.44**
PSPTO_5467	hypothetical protein PSPTO_5467	**1.43**
PSPTO_0228	hypothetical protein PSPTO_0228	**1.43**
PSPTO_0469	hypothetical protein PSPTO_0469	**1.43**
PSPTO_4028	hypothetical protein PSPTO_4028	**1.43**
PSPTO_3569	hypothetical protein PSPTO_3569	**1.43**
PSPTO_2998	hypothetical protein PSPTO_2998	**1.42**
PSPTO_0436	hypothetical protein PSPTO_0436	**1.41**
PSPTO_4464	hypothetical protein PSPTO_4464	**1.41**
PSPTO_1319	hypothetical protein PSPTO_1319	**1.4**
PSPTO_5503	YeeE/YedE family protein	**1.4**
PSPTO_4803	hypothetical protein PSPTO_4803	**1.39**
PSPTO_1721	hypothetical protein PSPTO_1721	**1.39**
PSPTO_5634	hypothetical protein PSPTO_5634	**1.39**
PSPTO_4016	hypothetical protein PSPTO_4016	**1.39**
PSPTO_1414	*yajC,* preprotein translocase subunit YajC	**1.38**
PSPTO_0343	*engB*, GTP-binding protein EngB	**1.38**
PSPTO_1331	acetyltransferase	**1.38**
PSPTO_2766	ABC transporter ATP-binding protein	**1.37**
PSPTO_1643	hypothetical protein PSPTO_1643	**1.37**
PSPTO_0723	hypothetical protein PSPTO_0723	**1.37**
PSPTO_0435	tRNA (guanine-N(7)-)-methyltransferase	**1.37**
PSPTO_2648	hypothetical protein PSPTO_2648	**1.37**
PSPTO_5403	HAD-superfamily hydrolase	**1.36**
PSPTO_2999	hypothetical protein PSPTO_2999	**1.34**
PSPTO_4423	*sspB*, stringent starvation protein B	**1.33**
PSPTO_4863	hypothetical protein PSPTO_4863	**1.31**
PSPTO_4949	membrane protein	**1.31**
PSPTO_5011	GNAT family acetyltransferase	**1.31**
PSPTO_1590	hypothetical protein PSPTO_1590	**1.31**
PSPTO_1829	hypothetical protein PSPTO_1829	**1.3**
PSPTO_0989	hypothetical protein PSPTO_0989	**1.3**
PSPTO_3480	methyl-accepting chemotaxis protein	**1.3**
PSPTO_4792	ISPsy6, transposase	**1.3**
PSPTO_2037	Smr domain protein	**1.29**
PSPTO_0206	hypothetical protein PSPTO_0206	**1.29**
PSPTO_3359	hypothetical protein PSPTO_3359	**1.28**
PSPTO_3303	hypothetical protein PSPTO_3303	**1.28**
PSPTO_4958	HAMP domain protein	**1.27**
PSPTO_3637	FlhB domain-containing protein	**1.26**
PSPTO_4994	ISPsy5, transposase	**1.26**
PSPTO_0521	DNA-binding protein	**1.26**
PSPTO_4545	hypothetical protein PSPTO_4545	**1.25**

PSPTO_0978	hypothetical protein PSPTO_0978	**1.25**
PSPTO_4251	ISPsy5, transposase	**1.25**
PSPTO_3710	GNAT family acetyltransferase	**1.24**
PSPTO_5091	hypothetical protein PSPTO_5091	**1.24**
PSPTO_3654	GNAT family acetyltransferase	**1.24**
PSPTO_2270	ErfK/YbiS/YcfS/YnhG family protein	**1.24**
PSPTO_3723	*hupB*, DNA-binding protein HU-beta	**1.24**
PSPTO_3996	ISPsy5, transposase	**1.24**
PSPTO_0215	hypothetical protein PSPTO_0215	**1.23**
PSPTO_1273	membrane protein	**1.23**
PSPTO_0804	membrane protein, MviN family	**1.23**
PSPTO_2284	membrane protein	**1.23**
PSPTO_4827	iojap-like protein	**1.23**
PSPTO_5095	membrane protein	**1.23**
PSPTO_4393	hypothetical protein PSPTO_4393	**1.22**
PSPTO_0081	hypothetical protein PSPTO_0081	**1.22**
PSPTO_2187	hypothetical protein PSPTO_2187	**1.22**
PSPTO_0697	hypothetical protein PSPTO_0697	**1.22**
PSPTO_5297	hypothetical protein PSPTO_5297	**1.22**
PSPTO_5148	polyhydroxyalkanoate granule-associated protein PhaI	**1.22**
PSPTO_4737	ISPsy5, transposase	**1.22**
PSPTO_4693	ISPsy5, transposase	**1.22**
PSPTO_4663	hypothetical protein PSPTO_4663	**1.21**
PSPTO_0035	ISPsy5, transposase	**1.21**
PSPTO_1189	ISPsy5, transposase	**1.21**
PSPTO_0688	lipoprotein	**1.2**
PSPTO_1686	*wrbA*, trp repressor binding protein	**1.2**
PSPTO_2214	hypothetical protein PSPTO_2214	**1.2**
PSPTO_1127	hypothetical protein PSPTO_1127	**1.2**
PSPTO_4418	tetrapyrrole methylase family protein	**1.2**
PSPTO_4118	hypothetical protein PSPTO_4118	**1.2**
PSPTO_0196	ISPsy5, transposase	**1.2**
PSPTO_1175	membrane protein	**1.2**
PSPTO_2221	lipoprotein	**1.19**
PSPTO_2038	hypothetical protein PSPTO_2038	**1.19**
PSPTO_0039	ISPsy5, transposase	**1.19**
PSPTO_2460	ISPsy5, transposase	**1.19**
PSPTO_5543	ISPsy5, transposase	**1.19**
PSPTO_4764	ISPsy5, transposase	**1.19**
PSPTO_5411	ISPsy5, transposase	**1.19**
PSPTO_4389	ISPsy5, transposase	**1.19**
PSPTO_4766	hypothetical protein PSPTO_4766	**1.19**
PSPTO_4431	hypothetical protein PSPTO_4431	**1.18**
PSPTO_3651	ISPsy5, transposase	**1.18**
PSPTO_3415	hypothetical protein PSPTO_3415	**1.18**
PSPTO_5212	ISPsy5, transposase	**1.18**
PSPTO_5175	hypothetical protein PSPTO_5175	**1.17**
PSPTO_0735	LrgA family protein	**1.17**
PSPTO_3390	tail protein D	**1.17**

PSPTO_4865	*fis*, global DNA-binding transcriptional dual regulator Fis	**1.16**
PSPTO_1145	hypothetical protein PSPTO_1145	**1.16**
PSPTO_3581	GNAT family acetyltransferase	**1.16**
PSPTO_4145	*capB*, cold shock protein CapB	**1.16**
PSPTO_5451	hypothetical protein PSPTO_5451	**1.16**
PSPTO_4060	ISPsy6, transposase	**1.16**
PSPTO_0603	hypothetical protein PSPTO_0603	**1.15**
PSPTO_1938	hypothetical protein PSPTO_1938	**1.15**
PSPTO_2204	ISPsy6, transposase	**1.15**
PSPTO_1180	hypothetical protein PSPTO_1180	**1.14**
PSPTO_0345	hypothetical protein PSPTO_0345	**1.14**
PSPTO_1098	ISPsy5, transposase	**1.14**
PSPTO_1477	ISPsy6, transposase	**1.14**
PSPTO_2360	hypothetical protein PSPTO_2360	**1.13**
PSPTO_1179	HopJ1 protein	**1.13**
PSPTO_5012	hypothetical protein PSPTO_5012	**1.13**
PSPTO_1722	hypothetical protein PSPTO_1722	**1.13**
PSPTO_3416	holin	**1.13**
PSPTO_3414	hypothetical protein PSPTO_3414	**1.13**
PSPTO_4032	*recX*, recX protein	**1.12**
PSPTO_3236	hypothetical protein PSPTO_3236	**1.12**
PSPTO_2002	*ccoQ*, cytochrome c oxidase, cbb3-type, CcoQ subunit	**1.11**
PSPTO_3802	1-aminocyclopropane-1-carboxylate deaminase	**1.11**
PSPTO_0651	*rpoA*, DNA-directed RNA polymerase subunit alpha	**1.11**
PSPTO_3804	hydrolase	**1.11**
PSPTO_5048	hypothetical protein PSPTO_5048	**1.11**
PSPTO_5226	hypothetical protein PSPTO_5226	**1.11**
PSPTO_5151	hypothetical protein PSPTO_5151	**1.11**
PSPTO_2580	membrane protein	**1.1**
PSPTO_4195	hypothetical protein PSPTO_4195	**1.1**
PSPTO_2468	hypothetical protein PSPTO_2468	**1.1**
PSPTO_4802	hypothetical protein PSPTO_4802	**1.1**
PSPTO_4449	phosphatase, YrbI family	**1.1**
PSPTO_0825	hypothetical protein PSPTO_0825	**1.1**
PSPTO_0747	transporter	**1.1**
PSPTO_1126	amine oxidase, flavin-containing protein	**1.09**
PSPTO_4443	STAS domain-containing protein	**1.09**
PSPTO_0415	hypothetical protein PSPTO_0415	**1.09**
PSPTO_2052	hypothetical protein PSPTO_2052	**1.09**
PSPTO_0971	*sfsA*, sugar fermentation stimulation protein	**1.09**
PSPTO_4159	bacterioferritin-associated ferredoxin	**1.09**
PSPTO_0403	hypothetical protein PSPTO_0403	**1.09**
PSPTO_3808	ISPsy6, transposase	**1.09**
PSPTO_1810	PHP domain-containing protein	**1.08**
PSPTO_4428	ATPase	**1.08**
PSPTO_0988	hypothetical protein PSPTO_0988	**1.08**
PSPTO_1433	hypothetical protein PSPTO_1433	**1.08**
PSPTO_1854	hypothetical protein PSPTO_1854	**1.08**
PSPTO_0992	*rimI*, ribosomal-protein-alanine acetyltransferase	**1.07**

PSPTO_0670	ISPsy5, transposase	**1.07**
PSPTO_4583	hypothetical protein PSPTO_4583	**1.06**
PSPTO_1589	lipoprotein	**1.06**
PSPTO_2254	methyl-accepting chemotaxis protein	**1.05**
PSPTO_0282	membrane protein	**1.05**
PSPTO_5591	ISPsy5, transposase	**1.05**
PSPTO_2469	hypothetical protein PSPTO_2469	**1.04**
PSPTO_4218	hypothetical protein PSPTO_4218	**1.04**
PSPTO_1227	ISPsy5, transposase	**1.04**
PSPTO_2072	auxin-binding protein	**1.03**
PSPTO_5008	hypothetical protein PSPTO_5008	**1.03**
PSPTO_5252	DedA family protein	**1.03**
PSPTO_4845	lipoprotein	**1.03**
PSPTO_2971	ISPsy5, transposase	**1.03**
PSPTO_4826	hypothetical protein PSPTO_4826	**1.02**
PSPTO_5338	*hisB,* imidazoleglycerol-phosphate dehydratase	**1.02**
PSPTO_4185	membrane protein	**1.02**
PSPTO_4133	penicillin amidase family protein	**1.02**
PSPTO_3999	ISPsy5, transposase	**1.02**
PSPTO_2067	hypothetical protein PSPTO_2067	**1.02**
PSPTO_2721	membrane protein	**1.01**
PSPTO_2609	hypothetical protein PSPTO_2609	**1.01**
PSPTO_2000	prevent-host-death family protein	**1.01**
PSPTO_3407	hypothetical protein PSPTO_3407	**1.01**
PSPTO_4567	ISPsy5, transposase	**1.01**
PSPTO_5445	ISPsy5, transposase	**1.01**
PSPTO_2375	lipoprotein	**1**
PSPTO_1066	methyl-accepting chemotaxis protein	**-1**
PSPTO_0702	hypothetical protein PSPTO_0702	**-1**
PSPTO_0832	ISPsy4, transposition helper protein	**-1**
PSPTO_5624	Tat (twin-arginine translocation) pathway signal sequence domain-containing protein	**-1.01**
PSPTO_3586	LysR family transcriptional regulator	**-1.01**
PSPTO_5378	LamB/YcsF family protein	**-1.01**
PSPTO_0023	hypothetical protein PSPTO_0023	**-1.01**
PSPTO_1651	hypothetical protein PSPTO_1651	**-1.02**
PSPTO_2501	hypothetical protein PSPTO_2501	**-1.02**
PSPTO_4657	zinc metallopeptidase	**-1.02**
PSPTO_2075	hypothetical protein PSPTO_2075	**-1.02**
PSPTO_0193	ISPsy4, transposition helper protein	**-1.02**
PSPTO_0049	hypothetical protein PSPTO_0049	**-1.02**
PSPTO_4532	hypothetical protein PSPTO_4532	**-1.02**
PSPTO_2675	hypothetical protein PSPTO_2675	**-1.03**
PSPTO_3092	hypothetical protein PSPTO_3092	**-1.03**
PSPTO_5432	hypothetical protein PSPTO_5432	**-1.04**
PSPTO_2144	hypothetical protein PSPTO_2144	**-1.04**
PSPTO_2142	hypothetical protein PSPTO_2142	**-1.04**
PSPTO_2623	phenazine biosynthesis protein, PhzF family	**-1.04**
PSPTO_5232	pyocin/colicin protein	**-1.04**
PSPTO_0333	hypothetical protein PSPTO_0333	**-1.05**

PSPTO_2741	ISPsy4, transposition helper protein	**-1.05**
PSPTO_2238	lipoprotein	**-1.06**
PSPTO_4317	hypothetical protein PSPTO_4317	**-1.06**
PSPTO_0091	hypothetical protein PSPTO_0091	**-1.06**
PSPTO_5620	hypothetical protein PSPTO_5620	**-1.06**
PSPTO_2147	pyoverdine sidechain peptide synthetase I, epsilon-Lys module	**-1.07**
PSPTO_2924	hypothetical protein PSPTO_2924	**-1.07**
PSPTO_0172	hypothetical protein PSPTO_0172	**-1.07**
PSPTO_2086	hypothetical protein PSPTO_2086	**-1.07**
PSPTO_5211	hypothetical protein PSPTO_5211	**-1.07**
PSPTO_0013	hypothetical protein PSPTO_0013	**-1.07**
PSPTO_4750	hypothetical protein PSPTO_4750	**-1.07**
PSPTO_1609	hypothetical protein PSPTO_1609	**-1.08**
PSPTO_0012	hypothetical protein PSPTO_0012	**-1.08**
PSPTO_5208	hypothetical protein PSPTO_5208	**-1.08**
PSPTO_4327	hypothetical protein PSPTO_4327	**-1.09**
PSPTO_2536	hypothetical protein PSPTO_2536	**-1.09**
PSPTO_4351	hypothetical protein PSPTO_4351	**-1.1**
PSPTO_2071	hypothetical protein PSPTO_2071	**-1.11**
PSPTO_0758	2OG-Fe(II) oxygenase family oxidoreductase	**-1.11**
PSPTO_0374	hypothetical protein PSPTO_0374	**-1.11**
PSPTO_3602	hypothetical protein PSPTO_3602	**-1.11**
PSPTO_3995	abortive infection protein, internal deletion	**-1.11**
PSPTO_2888	hypothetical protein PSPTO_2888	**-1.11**
PSPTO_4761	hypothetical protein PSPTO_4761	**-1.11**
PSPTO_4574	hypothetical protein PSPTO_4574	**-1.12**
PSPTO_3880	polyamine ABC transporter permease	**-1.12**
PSPTO_3779	hypothetical protein PSPTO_3779	**-1.12**
PSPTO_2773	hypothetical protein PSPTO_2773	**-1.12**
PSPTO_2091	hypothetical protein PSPTO_2091	**-1.12**
PSPTO_1657	hypothetical protein PSPTO_1657	**-1.12**
PSPTO_2947	major facilitator family transporter	**-1.13**
PSPTO_3055	hypothetical protein PSPTO_3055	**-1.13**
PSPTO_5630	hypothetical protein PSPTO_5630	**-1.13**
PSPTO_4723	hypothetical protein PSPTO_4723	**-1.13**
PSPTO_3378	membrane protein	**-1.14**
PSPTO_1603	hypothetical protein PSPTO_1603	**-1.14**
PSPTO_2953	LysR family transcriptional regulator	**-1.15**
PSPTO_5330	hypothetical protein PSPTO_5330	**-1.15**
PSPTO_3889	hypothetical protein PSPTO_3889	**-1.15**
PSPTO_3065	hypothetical protein PSPTO_3065	**-1.15**
PSPTO_1368	lipoprotein	**-1.16**
PSPTO_2145	iron-regulated membrane protein	**-1.17**
PSPTO_2520	pyridine nucleotide-disulfide oxidoreductase family protein	**-1.17**
PSPTO_0866	hypothetical protein PSPTO_0866	**-1.17**
PSPTO_0916	methyl-accepting chemotaxis protein	**-1.17**
PSPTO_0109	hypothetical protein PSPTO_0109	**-1.17**
PSPTO_3947	hypothetical protein PSPTO_3947	**-1.17**
PSPTO_2318	hypothetical protein PSPTO_2318	**-1.17**

PSPTO_0117	methyl-accepting chemotaxis protein	**-1.18**
PSPTO_3605	lyase	**-1.18**
PSPTO_3041	membrane protein	**-1.18**
PSPTO_4348	hypothetical protein PSPTO_4348	**-1.18**
PSPTO_0867	hypothetical protein PSPTO_0867	**-1.19**
PSPTO_2143	hypothetical protein PSPTO_2143	**-1.19**
PSPTO_0525	lipoprotein	**-1.19**
PSPTO_0027	hypothetical protein PSPTO_0027	**-1.19**
PSPTO_2779	endoribonuclease L-PSP family protein	**-1.2**
PSPTO_2868	hypothetical protein PSPTO_2868	**-1.2**
PSPTO_4609	hypothetical protein PSPTO_4609	**-1.2**
PSPTO_2480	methyl-accepting chemotaxis protein	**-1.21**
PSPTO_1117	hypothetical protein PSPTO_1117	**-1.21**
PSPTO_4040	hypothetical protein PSPTO_4040	**-1.21**
PSPTO_4797	hypothetical protein PSPTO_4797	**-1.21**
PSPTO_2631	hypothetical protein PSPTO_2631	**-1.21**
PSPTO_2535	hypothetical protein PSPTO_2535	**-1.22**
PSPTO_2677	short chain dehydrogenase/reductase family oxidoreductase	**-1.23**
PSPTO_3538	transferase, hexapeptide repeat protein	**-1.23**
PSPTO_4714	cmaU protein	**-1.23**
PSPTO_0711	hypothetical protein PSPTO_0711	**-1.24**
PSPTO_3253	hypothetical protein PSPTO_3253	**-1.25**
PSPTO_0297	hypothetical protein PSPTO_0297	**-1.25**
PSPTO_2949	3-alpha-hydroxysteroid dehydrogenase	**-1.26**
PSPTO_1094	hypothetical protein PSPTO_1094	**-1.26**
PSPTO_1364	hypothetical protein PSPTO_1364	**-1.26**
PSPTO_3054	short chain dehydrogenase/reductase family oxidoreductase	**-1.27**
PSPTO_0477	hypothetical protein PSPTO_0477	**-1.27**
PSPTO_3091	nickel ABC transporter, ATP-binding protein	**-1.28**
PSPTO_4370	hypothetical protein PSPTO_4370	**-1.28**
PSPTO_4318	integral membrane protein	**-1.29**
PSPTO_3700	oxidoreductase, aldo/keto reductase family	**-1.29**
PSPTO_1851	hypothetical protein PSPTO_1851	**-1.3**
PSPTO_1367	GNAT family acetyltransferase	**-1.3**
PSPTO_1793	hypothetical protein PSPTO_1793	**-1.31**
PSPTO_5054	hypothetical protein PSPTO_5054	**-1.31**
PSPTO_4289	hypothetical protein PSPTO_4289	**-1.31**
PSPTO_3174	hypothetical protein PSPTO_3174	**-1.31**
PSPTO_2506	lipoprotein	**-1.31**
PSPTO_2620	hypothetical protein PSPTO_2620	**-1.32**
PSPTO_5312	hypothetical protein PSPTO_5312	**-1.32**
PSPTO_2231	YD repeat protein, partial	**-1.32**
PSPTO_3203	hypothetical protein PSPTO_3203	**-1.32**
PSPTO_1082	hypothetical protein PSPTO_1082	**-1.32**
PSPTO_2515	lipoprotein	**-1.33**
PSPTO_3061	LysR family transcriptional regulator	**-1.33**
PSPTO_0507	hypothetical protein PSPTO_0507	**-1.34**
PSPTO_3337	hypothetical protein PSPTO_3337	**-1.34**
PSPTO_3426	hypothetical protein PSPTO_3426	**-1.35**

PSPTO_0454	hypothetical protein PSPTO_0454	**-1.36**
PSPTO_2165	group II intron, maturase	**-1.36**
PSPTO_3057	MmgE/PrpD family protein	**-1.36**
PSPTO_4546	hypothetical protein PSPTO_4546	**-1.37**
PSPTO_1060	hypothetical protein PSPTO_1060	**-1.37**
PSPTO_5435	secreted protein Hcp	**-1.37**
PSPTO_1069	membrane protein	**-1.37**
PSPTO_3129	hypothetical protein PSPTO_3129	**-1.38**
PSPTO_4103	hypothetical protein PSPTO_4103	**-1.38**
PSPTO_4772	hypothetical protein PSPTO_4772	**-1.38**
PSPTO_5633	hypothetical protein PSPTO_5633	**-1.38**
PSPTO_0063	hypothetical protein PSPTO_0063	**-1.39**
PSPTO_2977	hypothetical protein PSPTO_2977	**-1.39**
PSPTO_0030	hypothetical protein PSPTO_0030	**-1.39**
PSPTO_1070	lipopolysaccharide biosynthesis protein	**-1.39**
PSPTO_0007	hypothetical protein PSPTO_0007	**-1.4**
PSPTO_4967	hypothetical protein PSPTO_4967	**-1.4**
PSPTO_4067	short-chain dehydrogenase/reductase family oxidoreductase	**-1.4**
PSPTO_1571	hypothetical protein PSPTO_1571	**-1.4**
PSPTO_0015	hypothetical protein PSPTO_0015	**-1.4**
PSPTO_1073	membrane protein	**-1.41**
PSPTO_2778	amino acid ABC transporter ATP-binding protein	**-1.42**
PSPTO_1035	colicin/pyosin nuclease family protein	**-1.42**
PSPTO_3539	membrane protein PslK	**-1.43**
PSPTO_0395	hypothetical protein PSPTO_0395	**-1.43**
PSPTO_0031	Ser/Thr protein phosphatase family protein	**-1.43**
PSPTO_4014	ISPsy4, transposition helper protein	**-1.43**
PSPTO_4716	hypothetical protein PSPTO_4716	**-1.43**
PSPTO_4602	putative ABC transporter ATP-binding protein	**-1.44**
PSPTO_2912	glutamine ABC transporter, periplasmic amino acid-binding protein	**-1.44**
PSPTO_1092	mobilization protein MobB	**-1.44**
PSPTO_3558	glcG protein	**-1.45**
PSPTO_2322	ISPsy4, transposition helper protein	**-1.45**
PSPTO_2539	secreted protein Hcp	**-1.45**
PSPTO_5631	hypothetical protein PSPTO_5631	**-1.45**
PSPTO_1900	hypothetical protein PSPTO_1900	**-1.45**
PSPTO_2859	hypothetical protein PSPTO_2859	**-1.45**
PSPTO_2085	hypothetical protein PSPTO_2085	**-1.45**
PSPTO_5576	ISPsy4, transposition helper protein	**-1.45**
PSPTO_2116	hypothetical protein PSPTO_2116	**-1.46**
PSPTO_4335	hypothetical protein PSPTO_4335	**-1.46**
PSPTO_1167	hypothetical protein PSPTO_1167	**-1.46**
PSPTO_2553	hypothetical protein PSPTO_2553	**-1.46**
PSPTO_1303	hypothetical protein PSPTO_1303	**-1.47**
PSPTO_2441	methyl-accepting chemotaxis protein	**-1.47**
PSPTO_4673	hypothetical protein PSPTO_4673	**-1.48**
PSPTO_1096	ISPsy4, transposition helper protein	**-1.48**
PSPTO_4342	insecticidal toxin protein	**-1.49**
PSPTO_2942	hypothetical protein PSPTO_2942	**-1.5**

PSPTO_5203	hypothetical protein PSPTO_5203	**-1.5**
PSPTO_3225	ISPsy4, transposition helper protein	**-1.5**
PSPTO_3014	ISPsy4, transposition helper protein	**-1.5**
PSPTO_0142	hypothetical protein PSPTO_0142	**-1.51**
PSPTO_2537	hypothetical protein PSPTO_2537	**-1.51**
PSPTO_5053	hypothetical protein PSPTO_5053	**-1.52**
PSPTO_5370	ISPsy4, transposition helper protein	**-1.52**
PSPTO_3946	hypothetical protein PSPTO_3946	**-1.52**
PSPTO_3133	methyltransferase	**-1.53**
PSPTO_4623	hypothetical protein PSPTO_4623	**-1.53**
PSPTO_1259	hypothetical protein PSPTO_1259	**-1.53**
PSPTO_0016	hypothetical protein PSPTO_0016	**-1.53**
PSPTO_4626	ISPsy4, transposition helper protein	**-1.53**
PSPTO_2514	hypothetical protein PSPTO_2514	**-1.54**
PSPTO_0155	hypothetical protein PSPTO_0155	**-1.54**
PSPTO_3770	hypothetical protein PSPTO_3770	**-1.54**
PSPTO_1869	hypothetical protein PSPTO_1869	**-1.54**
PSPTO_4258	NAD(P)H dehydrogenase, quinone family	**-1.54**
PSPTO_0528	hypothetical protein PSPTO_0528	**-1.54**
PSPTO_4206	globin family protein	**-1.55**
PSPTO_0056	ISPsy4, transposition helper protein	**-1.55**
PSPTO_3050	AraC family transcriptional regulator	**-1.55**
PSPTO_5352	methyl-accepting chemotaxis protein	**-1.56**
PSPTO_4207	hypothetical protein PSPTO_4207	**-1.56**
PSPTO_2020	hypothetical protein PSPTO_2020	**-1.57**
PSPTO_2695	hypothetical protein PSPTO_2695	**-1.58**
PSPTO_4270	ISPsy4, transposition helper protein	**-1.58**
PSPTO_5616	hypothetical protein PSPTO_5616	**-1.58**
PSPTO_4039	hypothetical protein PSPTO_4039	**-1.58**
PSPTO_1655	hypothetical protein PSPTO_1655	**-1.59**
PSPTO_2320	hypothetical protein PSPTO_2320	**-1.6**
PSPTO_4711	coronamic acid synthetase CmaC	**-1.6**
PSPTO_5210	EF hand domain-containing protein	**-1.61**
PSPTO_5644	hypothetical protein PSPTO_5644	**-1.62**
PSPTO_2239	YD repeat protein	**-1.62**
PSPTO_3485	lipase family protein	**-1.62**
PSPTO_3331	protease inhibitor Inh	**-1.63**
PSPTO_0295	hypothetical protein PSPTO_0295	**-1.63**
PSPTO_5373	hypothetical protein PSPTO_5373	**-1.63**
PSPTO_2391	hypothetical protein PSPTO_2391	**-1.64**
PSPTO_1596	hypothetical protein PSPTO_1596	**-1.64**
PSPTO_2013	hypothetical protein PSPTO_2013	**-1.64**
PSPTO_2873	hypothetical protein PSPTO_2873	**-1.65**
PSPTO_1451	hypothetical protein PSPTO_1451	**-1.65**
PSPTO_2879	lipoprotein	**-1.66**
PSPTO_3736	hypothetical protein PSPTO_3736	**-1.66**
PSPTO_2084	hypothetical protein PSPTO_2084	**-1.66**
PSPTO_2137	MbtH-like protein	**-1.66**
PSPTO_5625	binary cytotoxin component	**-1.69**

PSPTO_0675	arylesterase	**-1.71**
PSPTO_3938	hypothetical protein PSPTO_3938	**-1.71**
PSPTO_4717	hypothetical protein PSPTO_4717	**-1.71**
PSPTO_4676	hypothetical protein PSPTO_4676	**-1.71**
PSPTO_3132	hypothetical protein PSPTO_3132	**-1.73**
PSPTO_2492	short-chain dehydrogenase/reductase family oxidoreductase	**-1.73**
PSPTO_5456	hypothetical protein PSPTO_5456	**-1.74**
PSPTO_2442	chemotaxis protein CheW	**-1.74**
PSPTO_2796	hypothetical protein PSPTO_2796	**-1.74**
PSPTO_5206	EF hand domain-containing protein	**-1.74**
PSPTO_4741	hypothetical protein PSPTO_4741	**-1.74**
PSPTO_1931	hypothetical protein PSPTO_1931	**-1.75**
PSPTO_3547	LysR family transcriptional regulator	**-1.77**
PSPTO_4326	hypothetical protein PSPTO_4326	**-1.77**
PSPTO_3120	Cof-like hydrolase family protein	**-1.78**
PSPTO_0914	STAS domain-containing protein	**-1.78**
PSPTO_3548	hypothetical protein PSPTO_3548	**-1.78**
PSPTO_3728	hypothetical protein PSPTO_3728	**-1.78**
PSPTO_2396	short chain dehydrogenase/reductase family oxidoreductase	**-1.79**
PSPTO_5073	hypothetical protein PSPTO_5073	**-1.79**
PSPTO_1485	hypothetical protein PSPTO_1485	**-1.81**
PSPTO_4743	hypothetical protein PSPTO_4743	**-1.81**
PSPTO_1507	hypothetical protein PSPTO_1507	**-1.82**
PSPTO_4747	hypothetical protein PSPTO_4747	**-1.82**
PSPTO_5204	EF hand domain-containing protein	**-1.82**
PSPTO_5455	hypothetical protein PSPTO_5455	**-1.83**
PSPTO_2692	hypothetical protein PSPTO_2692	**-1.83**
PSPTO_2471	membrane protein	**-1.83**
PSPTO_5430	hypothetical protein PSPTO_5430	**-1.83**
PSPTO_3546	hypothetical protein PSPTO_3546	**-1.84**
PSPTO_1165	hypothetical protein PSPTO_1165	**-1.84**
PSPTO_4324	hypothetical protein PSPTO_4324	**-1.84**
PSPTO_4611	hypothetical protein PSPTO_4611	**-1.85**
PSPTO_3291	methyl-accepting chemotaxis protein	**-1.85**
PSPTO_3299	3-hydroxyacyl-CoA-acyl carrier protein transferase	**-1.86**
PSPTO_5233	colicin/pyocin immunity family protein	**-1.87**
PSPTO_4713	alanyl tRNA synthetase-related protein	**-1.88**
PSPTO_3218	hypothetical protein PSPTO_3218	**-1.88**
PSPTO_3600	oxidoreductase, molybdopterin-binding protein	**-1.89**
PSPTO_3131	hypothetical protein PSPTO_3131	**-1.91**
PSPTO_0156	hypothetical protein PSPTO_0156	**-1.91**
PSPTO_5207	hypothetical protein PSPTO_5207	**-1.92**
PSPTO_5071	hypothetical protein PSPTO_5071	**-1.94**
PSPTO_2550	hypothetical protein PSPTO_2550	**-1.96**
PSPTO_1093	relaxase/mobilization nuclease domain protein	**-1.97**
PSPTO_5458	hypothetical protein PSPTO_5458	**-1.99**
PSPTO_2397	short chain dehydrogenase	**-1.99**
PSPTO_1850	hypothetical protein PSPTO_1850	**-1.99**
PSPTO_0154	hypothetical protein PSPTO_0154	**-2**

PSPTO_1344	hypothetical protein PSPTO_1344	**-2**
PSPTO_3019	oxidoreductase, Gfo/Idh/MocA family	**-2**
PSPTO_3228	hypothetical protein PSPTO_3228	**-2.01**
PSPTO_2083	hypothetical protein PSPTO_2083	**-2.03**
PSPTO_3879	hypothetical protein PSPTO_3879	**-2.04**
PSPTO_3937	hypothetical protein PSPTO_3937	**-2.04**
PSPTO_5426	hypothetical protein PSPTO_5426	**-2.04**
PSPTO_1967	hypothetical protein PSPTO_1967	**-2.05**
PSPTO_1334	methyl-accepting chemotaxis protein	**-2.06**
PSPTO_2874	ppkA-related protein	**-2.07**
PSPTO_4754	hypothetical protein PSPTO_4754	**-2.07**
PSPTO_0038	hypothetical protein PSPTO_0038	**-2.08**
PSPTO_2877	hypothetical protein PSPTO_2877	**-2.09**
PSPTO_5423	hypothetical protein PSPTO_5423	**-2.09**
PSPTO_2641	lipoprotein	**-2.09**
PSPTO_5414	lipoprotein	**-2.1**
PSPTO_1097	membrane protein	**-2.1**
PSPTO_4586	hypothetical protein PSPTO_4586	**-2.11**
PSPTO_4325	hypothetical protein PSPTO_4325	**-2.13**
PSPTO_0307	hypothetical protein PSPTO_0307	**-2.16**
PSPTO_4746	site-specific recombinase, phage integrase family domain protein	**-2.16**
PSPTO_2952	glycosidase	**-2.17**
PSPTO_2390	hypothetical protein PSPTO_2390	**-2.17**
PSPTO_4634	N-acetylmuramoyl-L-alanine amidase	**-2.17**
PSPTO_0020	hypothetical protein PSPTO_0020	**-2.17**
PSPTO_2472	methyl-accepting chemotaxis protein	**-2.18**
PSPTO_5234	hypothetical protein PSPTO_5234	**-2.19**
PSPTO_4516	hypothetical protein PSPTO_4516	**-2.2**
PSPTO_1919	hypothetical protein PSPTO_1919	**-2.21**
PSPTO_3494	myo-inositol 2-dehydrogenase	**-2.21**
PSPTO_0029	transposition helper protein	**-2.21**
PSPTO_2872	HopL1 protein	**-2.22**
PSPTO_5427	hypothetical protein PSPTO_5427	**-2.24**
PSPTO_4742	site-specific recombinase, phage integrase family	**-2.24**
PSPTO_4321	hypothetical protein PSPTO_4321	**-2.28**
PSPTO_4733	hypothetical protein PSPTO_4733	**-2.28**
PSPTO_4719	hypothetical protein PSPTO_4719	**-2.29**
PSPTO_5205	hypothetical protein PSPTO_5205	**-2.3**
PSPTO_2895	hypothetical protein PSPTO_2895	**-2.31**
PSPTO_0021	hypothetical protein PSPTO_0021	**-2.31**
PSPTO_4587	hypothetical protein PSPTO_4587	**-2.32**
PSPTO_5365	hypothetical protein PSPTO_5365	**-2.36**
PSPTO_5364	hypothetical protein PSPTO_5364	**-2.37**
PSPTO_2431	hypothetical protein PSPTO_2431	**-2.38**
PSPTO_2878	lipoprotein	**-2.42**
PSPTO_4607	hypothetical protein PSPTO_4607	**-2.43**
PSPTO_1297	hypothetical protein PSPTO_1297	**-2.44**
PSPTO_4627	hypothetical protein PSPTO_4627	**-2.45**
PSPTO_2458	hypothetical protein PSPTO_2458	**-2.48**

PSPTO_3691	ea59 protein	**-2.5**
PSPTO_3492	oxidoreductase, Gfo/Idh/MocA family	**-2.5**
PSPTO_0871	macrolide efflux protein	**-2.51**
PSPTO_1090	hypothetical protein PSPTO_1090	**-2.54**
PSPTO_4610	hypothetical protein PSPTO_4610	**-2.56**
PSPTO_5420	hypothetical protein PSPTO_5420	**-2.56**
PSPTO_4605	hypothetical protein PSPTO_4605	**-2.58**
PSPTO_4334	hypothetical protein PSPTO_4334	**-2.59**
PSPTO_1410	exchangeable effector locus protein	**-2.59**
PSPTO_3690	hypothetical protein PSPTO_3690	**-2.61**
PSPTO_5619	hypothetical protein PSPTO_5619	**-2.67**
PSPTO_5355	hypothetical protein PSPTO_5355	**-2.68**
PSPTO_3217	hypothetical protein PSPTO_3217	**-2.69**
PSPTO_4606	hypothetical protein PSPTO_4606	**-2.7**
PSPTO_3616	hypothetical protein PSPTO_3616	**-2.71**
PSPTO_2696	mutT/nudix family protein	**-2.72**
PSPTO_4333	moxR protein	**-2.87**
PSPTO_4372	hypothetical protein PSPTO_4372	**-2.89**
PSPTO_4323	hypothetical protein PSPTO_4323	**-2.93**
PSPTO_5419	hypothetical protein PSPTO_5419	**-2.93**
PSPTO_5421	lipoprotein	**-2.93**
PSPTO_5209	hypothetical protein PSPTO_5209	**-2.94**
PSPTO_3929	cold shock domain family protein	**-2.95**
PSPTO_4322	hypothetical protein PSPTO_4322	**-2.96**
PSPTO_5437	hypothetical protein PSPTO_5437	**-3**
PSPTO_5413	EF hand domain-containing protein	**-3.08**
PSPTO_0856	hypothetical protein PSPTO_0856	**-3.17**
PSPTO_5645	hypothetical protein PSPTO_5645	**-3.28**
PSPTO_2422	hypothetical protein PSPTO_2422	**-3.43**
PSPTO_0837	hypothetical protein PSPTO_0837	**-3.53**
PSPTO_0201	hypothetical protein PSPTO_0201	**-3.57**
PSPTO_4272	hypothetical protein PSPTO_4272	**-3.59**
PSPTO_4763	hypothetical protein PSPTO_4763	**-3.63**
PSPTO_0371	indoleacetate-lysine ligase	**-3.64**
PSPTO_4386	hypothetical protein PSPTO_4386	**-3.73**
PSPTO_5646	hypothetical protein PSPTO_5646	**-3.79**
PSPTO_1371	effector locus protein	**-3.84**
PSPTO_4762	von Willebrand factor type A domain-containing protein	**-3.84**
PSPTO_4387	hypothetical protein PSPTO_4387	**-4.1**
PSPTO_0545	SpoVR like family protein	**-4.11**
PSPTO_0546	hypothetical protein PSPTO_0546	**-4.14**
PSPTO_1409	hypothetical protein PSPTO_1409	**-4.15**
PSPTO_2679	hypothetical protein PSPTO_2679	**-4.39**
PSPTO_1408	hypothetical protein PSPTO_1408	**-4.52**
PSPTO_5622	hypothetical protein PSPTO_5622	**-4.79**
PSPTO_4332	hypothetical protein PSPTO_4332	**-5.1**
PSPTO_0875	hypothetical protein PSPTO_0875	**-5.18**
PSPTO_0874	nikkomycin biosynthesis domain protein	**-5.23**
PSPTO_2457	hypothetical protein PSPTO_2457	**-5.34**

Table S2. List of differentially expressed genes of (p)ppGpp^0$_{PssB728a}$ versus *Pss*B728a with |log$_2$FC| value ≥1 and an adjusted p-value <0.05.

Locus tag	Gene description	(p)ppGpp^0$_{PssB728a}$ /*Pss*B728a
**Type III secretion system**		
PSYR_4659	*hopAB2*, type III effector HopAB2	**-1.28**
PSYR_1890	type III effector HopAP1	**-1.64**
PSYR_4269	type III effector HopAE1	**-1.65**
PSYR_1186	*hopM1*, type III effector HopM1	**-1.75**
PSYR_3813	*hopAF1*, type III effector HopAF1	**-1.91**
PSYR_0778	*hopAG1*, type III effector HopAG1	**-1.99**
PSYR_4326	*hopI1*, type III effector HopI1	**-2.05**
PSYR_0738	type III effector protein AvrRpm1	**-2.08**
PSYR_3839	*hopAK1*, type III helper protein HopAK1	**-2.26**
PSYR_1189	*hrpH*, membrane-bound lytic murein transglycosylase D	**-2.5**
PSYR_0779	*hopAH1*, type III effector HopAH1	**-2.54**
PSYR_1205	*hrcU*, type III secretion protein HrcU	**-2.74**
PSYR_1188	*avrE1*, type III effector protein AvrE1	**-2.77**
PSYR_1200	*hrcC*, outer-membrane type III secretion protein HrcC	**-2.95**
PSYR_1220	type III effector HopX1	**-3.04**
PSYR_1185	*shcM*, type III chaperone ShcM	**-3.35**
PSYR_1215	*hrcV*, type III secretion protein HrcV	**-3.37**
PSYR_1197	*hrpE*, type III secretion protein HrpE	**-3.46**
PSYR_1216	*hrpJ*, type III secretion protein HrpJ	**-3.46**
PSYR_1206	*hrcT*, type III secretion protein HrcT	**-3.58**
PSYR_1214	*hrpQ*, type III secretion protein HrpQ	**-3.59**
PSYR_1199	*hrpG*, type III secretion protein HrpG	**-3.63**
PSYR_1217	*hrpL*, RNA polymerase sigma factor HrpL	**-3.78**
PSYR_1202	*hrpV*, negative regulator of hrp expression HrpV	**-3.91**
PSYR_1213	*hrcN*, type III secretion cytoplasmic ATPase HrcN	**-3.94**
PSYR_1198	type III secretion protein HrpF	**-4.04**
PSYR_1201	*hrpT*, type III secretion protein	**-4.07**
PSYR_1195	*hrcJ*, type III secretion protein HrcJ	**-4.24**
PSYR_1889	*hopH1*, type III effector HopH1	**-4.24**
PSYR_1196	*hrpD*, type III secretion protein HrpD	**-4.24**
PSYR_1184	*hrpW1*, type III helper protein HrpW1	**-4.31**
PSYR_1207	*hrcS*, type III secretion protein HrcS	**-4.32**
PSYR_1219	type III effector protein AvrB3	**-4.56**
PSYR_1218	*hrpK1*, type III helper protein HrpK1	**-4.65**
PSYR_1208	*hrcR*, type III secretion protein HrcR	**-4.69**
PSYR_1210	*hrcQa*, type III secretion protein HrcQa	**-4.91**
PSYR_1194	*hrpB*, type III secretion protein HrpB	**-5.17**
PSYR_1212	*hrpO*, type III secretion protein HrpO	**-5.2**
PSYR_1183	*hopAA1-1*, type III effector HopAA1-1	**-5.28**
PSYR_1193	*hrpZ1*, type III restriction system endonuclease	**-5.36**
PSYR_1209	*hrcQb*, type III secretion protein HrcQb	**-5.76**
PSYR_1192	type III helper protein HrpA2	**-5.96**
PSYR_4919	*avrPto1*, type III effector protein AvrPto1	**-6.01**
PSYR_1211	*hrpP*, type III secretion protein HrpP	**-6.34**
**Translation, ribosomal structure and biogenesis**		

PSYR_0208	endoribonuclease L-PSP	**3.22**
PSYR_1639	*rluC*, ribosomal large subunit pseudouridine synthase C	**2.46**
PSYR_3182	*infA*, translation initiation factor IF-1	**2.33**
PSYR_1728	hypothetical protein PSYR_1728	**2.26**
PSYR_0018	*fmt*, methionyl-tRNA formyltransferase	**2.24**
PSYR_0399	*rpmE-2*, 50S ribosomal protein L31	**2.17**
PSYR_0225	*rpmG,* 50S ribosomal protein L33	**2.14**
PSYR_1343	*rpsB*, 30S ribosomal protein S2	**2.07**
PSYR_0019	*def-1*, polypeptide deformylase	**2.03**
PSYR_0213	*rph*, ribonuclease PH	**2.02**
PSYR_0224	*rpmB*, 50S ribosomal protein L28	**2**
PSYR_1390	S4 domain-containing protein	**1.9**
PSYR_0726	*rluD*, ribosomal large subunit pseudouridine synthase D	**1.7**
PSYR_0986	*rsmC*, ribosomal RNA small subunit methyltransferase C	**1.66**
PSYR_0701	*rplU*, 50S ribosomal protein L21	**1.66**
PSYR_2165	50S ribosomal protein L20	**1.66**
PSYR_2163	translation initiation factor IF-3	**1.66**
PSYR_2108	23S rRNA m(2)G2445 methyltransferase	**1.63**
PSYR_2164	50S ribosomal protein L35	**1.63**
PSYR_1344	*tsf,* translation elongation factor Ts	**1.62**
PSYR_1227	*queA*, S-adenosylmethionine--tRNA ribosyltransferase-isomerase	**1.59**
PSYR_0397	*argS*, arginyl-tRNA synthetase	**1.53**
PSYR_1977	*gltX*, glutamyl-tRNA synthetase	**1.53**
PSYR_4279	methyltransferase	**1.52**
PSYR_0012	*glyQ*, glycyl-tRNA synthetase subunit alpha	**1.52**
PSYR_4119	*rpsI*, 30S ribosomal protein S9	**1.45**
PSYR_1228	*tgt*, queuine tRNA-ribosyltransferase	**1.45**
PSYR_4558	*rplA*, 50S ribosomal protein L1	**1.4**
PSYR_0707	*rpsT*, 30S ribosomal protein S20	**1.38**
PSYR_1563	*rnd*, ribonuclease D	**1.37**
PSYR_2162	*thrS*, threonyl-tRNA synthetase	**1.37**
PSYR_3648	initiation factor 2 subunit family	**1.36**
PSYR_1234	RNA methyltransferase, TrmH family, group 1	**1.3**
PSYR_4120	*rplM*, 50S ribosomal protein L13	**1.3**
PSYR_1285	*rplS*, 50S ribosomal protein L19	**1.3**
PSYR_0830	*pcnB*, poly(A) polymerase	**1.29**
PSYR_0017	sun protein	**1.28**
PSYR_3579	hypothetical protein PSYR_3579	**1.26**
PSYR_1406	*aspS*, aspartyl-tRNA synthetase	**1.26**
PSYR_4559	*rplK*, 50S ribosomal protein L11	**1.25**
PSYR_4549	*rpsJ*, 30S ribosomal protein S10	**1.21**
PSYR_3585	Sua5/YciO/YrdC/YwlC family protein	**1.18**
PSYR_3639	hypothetical protein PSYR_3639	**1.18**
PSYR_2133	RNA pseudouridine synthase family protein	**1.17**
PSYR_1644	*rpmF*, 50S ribosomal protein L32	**1.16**
PSYR_4345	*miaB*, tRNA-i(6)A37 modification enzyme MiaB	**1.15**
PSYR_1370	hypothetical protein PSYR_1370	**1.13**
PSYR_0943	ribosomal 5S rRNA E-loop binding protein Ctc/L25/TL5	**1.13**
PSYR_4881	TrmH family RNA methyltransferase	**1.12**

PSYR_4548	*rplC*, 50S ribosomal protein L3	**1.12**
PSYR_4634	*cca*, tRNA nucleotidyltransferase	**1.1**
PSYR_5137	*rpmH*, 50S ribosomal protein L34	**1.08**
PSYR_0011	*glyS*, glycyl-tRNA synthetase subunit beta	**1.07**
PSYR_4639	*rpsU*, 30S ribosomal protein S21	**1.04**
PSYR_3895	*rnt*, ribonuclease T	**1.03**
PSYR_0584	*rplI*, 50S ribosomal protein L9	**1.02**
PSYR_1796	N-acetyltransferase GCN5	**-1.15**
PSYR_5100	hypothetical protein PSYR_5100	**-1.3**
PSYR_2887	deaminase AmnE	**-1.36**
PSYR_5059	GNAT family acetyltransferase	**-1.43**
PSYR_4159	*cafA*, ribonuclease G	**-1.48**
PSYR_1462	N-acetyltransferase GCN5	**-1.62**
PSYR_4179	*rbfA*, ribosome-binding factor A	**-1.7**
PSYR_2691	bifunctional UDP-glucuronic acid decarboxylase/UDP-4-amino-4-deoxy-L-arabinose formyltransferase	**-1.71**
PSYR_4627	*ksgA*, dimethyladenosine transferase	**-3.5**
PSYR_2107	*rmf*, ribosome modulation factor-related protein	**-4**
**RNA processing and modification**		
PSYR_0564	*orn*, oligoribonuclease	**1.83**
**Transcription**		
PSYR_0230	GntR family transcriptional regulator/aminotransferase, class I	**2.53**
PSYR_1044	LysR family transcriptional regulator	**2.5**
PSYR_0653	heavy metal-dependent transcriptional regulator	**2.41**
PSYR_3283	*lexA-2*, LexA repressor	**2.2**
PSYR_3028	hypothetical protein PSYR_3028	**2.14**
PSYR_2141	hypothetical protein PSYR_2141	**1.96**
PSYR_2065	*greB-1*, transcription elongation factor GreB	**1.91**
PSYR_0257	S1 RNA binding domain-containing protein	**1.71**
PSYR_2189	ArsR family transcriptional regulator	**1.67**
PSYR_1916	RulA protein	**1.58**
PSYR_0660	transcriptional regulator	**1.43**
PSYR_3659	*bolA*, bolA protein	**1.39**
PSYR_4299	helix-turn-helix, Fis-type	**1.35**
PSYR_0623	transcriptional regulator	**1.33**
PSYR_0301	rho, transcription termination factor Rho	**1.31**
PSYR_4641	*rpoD*, RNA polymerase sigma-70 factor	**1.29**
PSYR_4560	*nusG*, transcription antitermination protein NusG	**1.28**
PSYR_3027	protein kinase	**1.27**
PSYR_0210	*rpoZ*, DNA-directed RNA polymerase subunit omega	**1.25**
PSYR_4603	repressor protein c2	**1.24**
PSYR_4748	*rpoH*, RNA polymerase sigma-32 factor	**1.23**
PSYR_2933	TetR family transcriptional regulator	**1.23**
PSYR_1858	LuxR family transcriptional regulator	**1.23**
PSYR_3917	*metR*, transcriptional activator MetR	**1.17**
PSYR_4463	*nrdR*, hypothetical protein PSYR_4463	**1.17**
PSYR_2077	Cys regulon transcriptional activator	**1.17**
PSYR_2405	GntR family transcriptional regulator	**1.12**
PSYR_2812	peptidase, S24 family	**1.09**
PSYR_1152	ArsR family transcriptional regulator	**1.08**

PSYR_0056	*algQ*, transcriptional regulator AlgQ	**1.08**
PSYR_0276	zinc uptake regulation protein	**1.04**
PSYR_0820	DNA-binding transcriptional regulator FruR	**-1.02**
PSYR_2882	xylose operon regluatory protein	**-1.05**
PSYR_4731	RNA polymerase sigma-70 family protein	**-1.07**
PSYR_5057	transcriptional regulator	**-1.18**
PSYR_2671	regulatory protein LysR	**-1.18**
PSYR_2154	*rbsR*, ribose operon repressor	**-1.22**
PSYR_3062	LysR family transcriptional regulator	**-1.29**
PSYR_3087	transcriptional regulator GntR	**-1.29**
PSYR_1622	LuxR transcriptional regulator	**-1.31**
PSYR_4811	AraC family transcriptional regulator	**-1.31**
PSYR_4708	AraC family transcriptional regulator	**-1.43**
PSYR_3185	cold shock domain family protein	**-1.43**
PSYR_1777	TetR family transcriptional regulator	**-1.55**
PSYR_0327	sigma-54-binding protein	**-1.64**
PSYR_0526	hypothetical protein PSYR_0526	**-1.91**
PSYR_2564	transcriptional regulator GntR	**-1.92**
PSYR_1312	TetR family transcriptional regulator	**-1.95**
PSYR_2575	*syrR*, transcriptional regulator SyrR	**-2.11**
PSYR_2607	regulatory protein LuxR	**-2.29**
PSYR_1094	cold shock domain family protein	**-2.45**
PSYR_4555	*rpoB*, DNA-directed RNA polymerase subunit beta	**-2.57**
PSYR_4554	*rpoC*, DNA-directed RNA polymerase subunit beta\'	**-3.52**
PSYR_3248	sigma-54 dependent transcriptional regulator	**-3.96**
**Replication, recombination and repair**		
PSYR_4703	exonuclease	**3.82**
PSYR_4297	ATP-dependent RNA helicase rhlE	**2**
PSYR_0458	ATP-dependent RNA helicase rhlE	**1.99**
PSYR_1378	*recA*, recA protein	**1.95**
PSYR_3619	ATP-dependent RNA helicase, DEAD box family	**1.92**
PSYR_3791	*srmB*, ATP-dependent RNA helicase SrmB	**1.84**
PSYR_0516	*dbpA*, ATP-independent RNA helicase DbpA	**1.83**
PSYR_1222	ISPsy5, transposase	**1.82**
PSYR_0400	staphylococcal nuclease-like protein	**1.8**
PSYR_2148	*endA*, endonuclease I	**1.75**
PSYR_3856	DinG family ATP-dependent helicase	**1.66**
PSYR_1764	*dnaQ*, DNA polymerase III subunit epsilon	**1.65**
PSYR_3918	mutT/nudix family protein	**1.62**
PSYR_2244	*topB*, DNA topoisomerase III	**1.55**
PSYR_4370	3-methyladenine DNA glycosylase	**1.45**
PSYR_1763	*rnhA*, ribonuclease HI	**1.42**
PSYR_0215	exodeoxyribonuclease III	**1.41**
PSYR_1226	site-specific recombinase, phage integrase family	**1.4**
PSYR_1588	recombination associated protein rdgC	**1.36**
PSYR_3027	protein kinase	**1.27**
PSYR_5065	*uvrD*, DNA helicase II	**1.25**
PSYR_3704	hypothetical protein PSYR_3704	**1.22**
PSYR_4761	*mutM*, formamidopyrimidine-DNA glycosylase	**1.15**

PSYR_3647	*gyrA*, DNA gyrase subunit A	**1.14**
PSYR_0075	*rep*, ATP-dependent DNA helicase Rep	**1.13**
PSYR_0484	hypothetical protein PSYR_0484	**1.13**
PSYR_4518	*ssB*, single-stranded DNA-binding protein	**1.07**
PSYR_0013	*tag*, DNA-3-methyladenine glycosidase I	**1.06**
PSYR_3970	*ung*, uracil-DNA glycosylase	**1.03**
PSYR_2060	TatD family hydrolase	**1.01**
PSYR_3804	ATP-dependent DNA helicase RecQ	**-1.06**
PSYR_1433	C-5 cytosine-specific DNA methylase	**-1.09**
PSYR_0736	umuC protein, partial	**-1.14**
PSYR_3805	hypothetical protein PSYR_3805	**-1.15**
PSYR_0681	exodeoxyribonuclease V subunit beta	**-1.22**
PSYR_2914	ATP-dependent DNA helicase RecQ	**-1.23**
PSYR_1715	exonuclease SbcD	**-1.26**
PSYR_3984	DNA helicase-like protein	**-1.32**
PSYR_1888	integrase/recombinase XerC	**-1.47**
PSYR_4754	methyltransferase	**-1.52**
PSYR_4354	*holA*, DNA polymerase III subunit delta	**-1.59**
PSYR_1522	single-stranded DNA-binding protein	**-1.61**
PSYR_2896	excinuclease ABC subunit C	**-1.63**
PSYR_0682	*recD*, exodeoxyribonuclease V subunit alpha	**-1.87**
PSYR_0526	hypothetical protein PSYR_0526	**-1.91**
PSYR_3888	endonuclease III	**-1.96**
PSYR_1357	ribonuclease HII	**-2.07**
PSYR_1714	exonuclease SbcC	**-2.19**
PSYR_2828	C-5 cytosine-specific DNA methylase	**-2.39**
PSYR_1090	*holC*, DNA polymerase III subunit chi	**-2.47**
PSYR_4091	mutT/nudix family protein	**-2.49**
**Cell cycle control, cell division, chromosome partitioning**		
PSYR_3284	*sulA*, cell division inhibitor	**1.44**
PSYR_1818	cell division protein ZipA	**1.29**
PSYR_1642	*maf-1*, maf protein	**1.17**
PSYR_3885	ParA family protein	**1.11**
PSYR_1612	*minD*, septum site-determining protein MinD	**1.08**
PSYR_3587	intracellular septation protein A	**1.03**
PSYR_3705	hypothetical protein PSYR_3705	**1**
PSYR_1360	cell cycle protein mesJ	**-1.01**
PSYR_1364	hypothetical protein PSYR_1364	**-1.26**
PSYR_4160	maf-2, maf protein	**-1.31**
PSYR_4103	cell division protein FtsW	**-1.56**
PSYR_3430	*ParA*, family protein	**-1.58**
PSYR_4098	cell division protein FtsA	**-1.97**
PSYR_4097	cell division protein FtsZ	**-3.01**
PSYR_1555	cobyrinic acid a,c-diamide synthase	**-3.51**
**Defense mechanisms**		
PSYR_0536	*msbA*, lipid A ABC transporter, ATP-binding/permease protein	**2.05**
PSYR_3037	membrane protein	**1.45**
PSYR_1905	*lolD*, lipoprotein releasing system, ATP-binding protein LolD	**1.33**
PSYR_2617	*syfC*, syringafactin efflux protein SyfC	**-1.03**

PSYR_2541	oligopeptide/dipeptide ABC transporter ATP-binding protein-like protein	**-1.23**
PSYR_2618	*syfD*, syringafactin efflux protein SyfD	**-1.49**
PSYR_2622	acriflavin resistance protein	**-1.6**
PSYR_4008	*saxB*, isothiocyanate resistance protein SaxB; isochorismatase family	**-1.77**
PSYR_3725	hypothetical protein PSYR_3725	**-1.8**
PSYR_2483	acriflavin resistance protein	**-1.89**
PSYR_2613	cyclic peptide transporter	**-2.16**
PSYR_4924	hypothetical protein PSYR_4924	**-2.17**
PSYR_3076	Type I secretion system ATPase, HlyB	**-2.72**
PSYR_3724	hypothetical protein PSYR_3724	**-2.88**
PSYR_2484	AcrB/AcrD/AcrF family protein	**-3.09**
PSYR_2628	ABC transporter ATP-binding protein	**-4.67**
**Signal transduction mechanisms**		
PSYR_4702	nucleotidyltransferase	**5.71**
PSYR_4273	*cstA*, carbon starvation protein CstA	**2.91**
PSYR_4821	*ntrB*, nitrogen regulation protein NtrB	**2.47**
PSYR_3497	hypothetical protein PSYR_3497	**2.31**
PSYR_3299	LuxR family DNA-binding response regulator	**2.21**
PSYR_1585	sensory box histidine kinase/response regulator	**2.01**
PSYR_4642	sensory box/GGDEF domain/EAL domain-containing protein	**2**
PSYR_4800	DNA-binding heavy metal response regulator	**1.95**
PSYR_4799	heavy metal sensor histidine kinase	**1.66**
PSYR_2113	extracellular solute-binding protein/sensory box protein, partial	**1.64**
PSYR_4822	*ntrC*, nitrogen regulation protein NR(I)	**1.61**
PSYR_0086	sensory box/GGDEF domain/EAL domain-containing protein	**1.35**
PSYR_4346	PhoH-like protein	**1.29**
PSYR_3027	protein kinase	**1.27**
PSYR_4070	*colR*, DNA-binding response regulator ColR	**1.23**
PSYR_3262	dnaK suppressor protein	**1.23**
PSYR_3293	universal stress protein family	**1.21**
PSYR_2024	*sixA*, phosphohistidine phosphatase SixA	**1.21**
PSYR_0263	alginate biosynthesis transcriptional regulator AlgB	**1.18**
PSYR_0063	alginate biosynthesis regulatory protein AlgR	**1.04**
PSYR_4937	sensor histidine kinase	**1**
PSYR_3329	PAS:GGDEF	**-1.01**
PSYR_3434	chemotaxis sensor histidine kinase CheA	**-1.04**
PSYR_3436	chemotaxis protein CheY	**-1.05**
PSYR_3655	sensory box/GGDEF domain/EAL domain-containing protein	**-1.06**
PSYR_1111	DNA-binding response regulator	**-1.07**
PSYR_2446	chemotaxis protein methyltransferase CheR	**-1.11**
PSYR_2939	diguanylate cyclase	**-1.15**
PSYR_4060	GGDEF domain/EAL domain protein	**-1.17**
PSYR_0868	histidine kinase, HAMP region: chemotaxis sensory transducer	**-1.17**
PSYR_2966	methyl-accepting chemotaxis protein	**-1.21**
PSYR_2076	universal stress protein family	**-1.21**
PSYR_2188	histidine kinase	**-1.21**
PSYR_1497	heavy metal response regulator	**-1.22**
PSYR_1304	chemotaxis protein CheW	**-1.24**

PSYR_0788	*cheY-1*, chemotaxis protein CheY	**-1.28**
PSYR_3435	chemotaxis protein CheZ	**-1.29**
PSYR_1159	sensory box/GGDEF domain/EAL domain-containing protein	**-1.29**
PSYR_3554	*csrA-2*, carbon storage regulator	**-1.29**
PSYR_1308	protein-glutamate methylesterase CheB	**-1.32**
PSYR_3996	sensory box DNA/response regulator	**-1.32**
PSYR_0492	sensor histidine kinase/response regulator	**-1.34**
PSYR_4770	sensory box/GGDEF domain/EAL domain-containing protein	**-1.35**
PSYR_0524	lipopolysaccharide core biosynthesis protein	**-1.35**
PSYR_0783	*cheR-1*, chemotaxis protein methyltransferase CheR	**-1.38**
PSYR_2597	diguanylate cyclase	**-1.38**
PSYR_2281	diguanylate cyclase	**-1.4**
PSYR_3451	response regulator	**-1.42**
PSYR_3452	STAS domain-containing protein	**-1.46**
PSYR_0525	lipopolysaccharide biosynthesis protein	**-1.51**
PSYR_1100	hypothetical protein PSYR_1100	**-1.55**
PSYR_1309	response regulator/GGDEF domain-containing protein	**-1.56**
PSYR_3532	sensory box histidine kinase/response regulator	**-1.6**
PSYR_2220	methyl-accepting chemotaxis protein	**-1.62**
PSYR_1307	sensor histidine kinase/response regulator	**-1.7**
PSYR_1305	chemotaxis protein methyltransferase CheR	**-1.76**
PSYR_1066	PhoH-like protein	**-1.77**
PSYR_3994	sensor histidine kinase	**-1.78**
PSYR_1776	methyl-accepting chemotaxis protein	**-1.81**
PSYR_0786	*cheA-1*, chemotaxis sensor histidine kinase CheA	**-1.81**
PSYR_3433	protein-glutamate methylesterase CheB	**-1.82**
PSYR_3093	hypothetical protein PSYR_3093	**-1.87**
PSYR_0526	hypothetical protein PSYR_0526	**-1.91**
PSYR_1306	chemotaxis protein CheW	**-1.96**
PSYR_0266	diguanylate cyclase	**-2.01**
PSYR_4377	response regulator	**-2.06**
PSYR_0994	HDIG domain protein	**-2.13**
PSYR_1112	sensor histidine kinase	**-2.17**
PSYR_0870	diguanylate cyclase	**-2.18**
PSYR_4221	GAF domain/GGDEF domain/EAL domain protein	**-2.19**
PSYR_0509	response regulator/sensory box/GGDEF domain/EAL domain protein	**-2.22**
PSYR_1099	response regulator receiver	**-2.27**
PSYR_3995	sigma-54 dependent transcriptional regulator/response regulator	**-2.35**
PSYR_2245	hypothetical protein PSYR_2245	**-2.38**
PSYR_4373	sensory box histidine kinase	**-2.41**
PSYR_4631	hypothetical protein PSYR_4631	**-2.46**
PSYR_0784	*cheW-1*, chemotaxis protein CheW	**-2.49**
PSYR_4408	sensor histidine kinase/response regulator RetS	**-2.64**
PSYR_4376	DNA binding response regulator, LuxR family	**-2.75**
PSYR_1098	sensory box protein/response regulator	**-2.77**
PSYR_4206	diguanylate cyclase	**-2.79**
PSYR_0785	methyl-accepting chemotaxis protein	**-3.17**
**Cell wall/membrane/envelope biogenesis**		
PSYR_4807	outer membrane porin, OprD family	**4.17**

PSYR_2496	lipopolysaccharide core biosynthesis domain protein	**2.81**
PSYR_1904	*lolC*, lipoprotein releasing system transmembrane protein LolC	**2.37**
PSYR_1232	hypothetical protein PSYR_1232	**2.37**
PSYR_5117	glucosamine--fructose-6-phosphate aminotransferase	**2.24**
PSYR_4841	*lgt*, prolipoprotein diacylglyceryl transferase	**2.22**
PSYR_2980	*galU*, UTP-glucose-1-phosphate uridylyltransferase	**2.14**
PSYR_0368	lipoprotein Blc	**2.06**
PSYR_1999	ompA family protein	**1.93**
PSYR_4743	*mtgA*, monofunctional biosynthetic peptidoglycan transglycosylase	**1.68**
PSYR_1614	*htrB,* lipid A biosynthesis lauroyl acyltransferase	**1.65**
PSYR_0014	lipid A biosynthesis lauroyl acyltransferase	**1.61**
PSYR_0542	*kdtA*, 3-deoxy-D-manno-octulosonic-acid transferase	**1.61**
PSYR_0842	penicillin-binding protein	**1.54**
PSYR_0725	competence lipoprotein ComL	**1.53**
PSYR_3919	D-alanine--D-alanine ligase	**1.5**
PSYR_4801	metal ion efflux outer membrane protein	**1.48**
PSYR_4086	mechanosensitive ion channel family protein	**1.4**
PSYR_0535	toluene tolerance protein	**1.37**
PSYR_4142	sugar isomerase	**1.35**
PSYR_1268	periplasmic binding domain/transglycosylase SLT domain fusion protein	**1.31**
PSYR_4365	penicillin-binding protein	**1.28**
PSYR_0937	mannose-1-phosphate guanylyltransferase/mannose-6-phosphate isomerase	**1.28**
PSYR_0051	hypothetical protein PSYR_0051	**1.25**
PSYR_0880	transglycosylase	**1.2**
PSYR_1906	*lolE*, lipoprotein releasing system transmembrane protein LolE	**1.2**
PSYR_1822	mechanosensitive ion channel family protein	**1.19**
PSYR_4900	hypothetical protein PSYR_4900	**1.18**
PSYR_0520	*rfaF*, ADP-heptose--LPS heptosyltransferase II	**1.16**
PSYR_0237	alanine racemase	**1.16**
PSYR_2478	hypothetical protein PSYR_2478	**1.15**
PSYR_0630	UDP-N-acetylmuramate:L-alanyl-gamma-D-glutamyl-meso-diaminopimelate ligase	**1.11**
PSYR_0637	membrane protein	**1.1**
PSYR_2313	penicillin-binding protein	**1.07**
PSYR_5035	peptidase, M23/M37 family	**1.06**
PSYR_0569	N-acetylmuramoyl-L-alanine amidase family protein	**1.04**
PSYR_2095	membrane protein	**1.03**
PSYR_3083	RND efflux system, outer membrane lipoprotein, NodT	**1.03**
PSYR_4135	UDP-N-acetylglucosamine 1-carboxyvinyltransferase	**1.01**
PSYR_1599	*prc*, tail-specific protease	**-1.02**
PSYR_1362	*kdsA*, 2-dehydro-3-deoxyphosphooctonate aldolase	**-1.03**
PSYR_4805	Rhs family protein	**-1.08**
PSYR_4104	UDP-N-acetylmuramoylalanine--D-glutamate ligase	**-1.15**
PSYR_4997	YD repeat-containing protein, partial	**-1.16**
PSYR_2703	hypothetical protein PSYR_2703	**-1.17**
PSYR_4033	insecticidal toxin protein	**-1.19**
PSYR_4036	insecticidal toxin protein	**-1.21**
PSYR_2690	glycosyl transferase family protein	**-1.26**
PSYR_2287	*tonB-2*, tonB protein	**-1.3**

PSYR_4566	peptidase, M23/M37 family	**-1.31**
PSYR_2620	RND efflux system, outer membrane lipoprotein, NodT	**-1.35**
PSYR_3490	hypothetical protein PSYR_3490	**-1.44**
PSYR_4102	UDP-N-acetylglucosamine-N-acetylmuramyl- (pentapeptide) pyrophosphoryl-undecaprenol N- acetylglucosamine transferase	**-1.48**
PSYR_3302	mannose-1-phosphate guanylyltransferase/mannose-6-phosphate isomerase PslB	**-1.49**
PSYR_0929	glycosyl transferase family protein	**-1.54**
PSYR_1060	*algK*, alginate biosynthesis protein AlgK	**-1.56**
PSYR_4101	UDP-N-acetylmuramate--L-alanine ligase	**-1.59**
PSYR_1059	*algE*, alginate biosynthesis protein AlgE	**-1.59**
PSYR_1516	hypothetical protein PSYR_1516	**-1.6**
PSYR_3760	hypothetical protein PSYR_3760	**-1.63**
PSYR_4100	D-alanine--D-alanine ligase	**-1.65**
PSYR_4105	*mraY*, phospho-N-acetylmuramoyl-pentapeptide-transferase	**-1.72**
PSYR_4994	rhs-like protein	**-1.72**
PSYR_4107	*murE*, UDP-N-acetylmuramoylalanyl-D-glutamate--2,6-diaminopimelate ligase	**-1.75**
PSYR_1352	outer membrane protein OmpH	**-1.82**
PSYR_4099	cell division protein FtsQ	**-1.83**
PSYR_3303	lipoprotein PslD	**-1.83**
PSYR_0920	group 1 glycosyl transferase	**-1.91**
PSYR_2693	4-amino-4-deoxy-L-arabinose transferase	**-1.97**
PSYR_2606	RND efflux system, outer membrane lipoprotein, NodT	**-2.02**
PSYR_1055	*algI,* alginate biosynthesis protein AlgI	**-2.07**
PSYR_1355	*lpxA*, UDP-N-acetylglucosamine acyltransferase	**-2.14**
PSYR_3309	membrane protein PslJ	**-2.15**
PSYR_1058	*algG*, alginate biosynthesis protein AlgG	**-2.15**
PSYR_2696	UDP-glucose 6-dehydrogenase	**-2.16**
PSYR_0956	cyclopropane-fatty-acyl-phospholipid synthase	**-2.19**
PSYR_1054	*algJ*, alginate biosynthesis protein AlgJ	**-2.24**
PSYR_4237	outer membrane porin, OprD family	**-2.26**
PSYR_2321	YD repeat-containing protein	**-2.48**
PSYR_1356	lipid A disaccharide synthase	**-2.51**
PSYR_1117	*oprB*, porin B	**-2.58**
PSYR_3304	exopolysaccharide biosynthesis protein PslE	**-2.62**
PSYR_2322	YD repeat-containing protein	**-2.62**
PSYR_1353	UDP-3-O	**-2.67**
PSYR_3468	glycosyl transferase family protein	**-2.88**
PSYR_4964	OmpA/MotB protein	**-2.95**
PSYR_2485	outer membrane efflux protein	**-3.15**
PSYR_1052	*algA*, alginate biosynthesis protein AlgA	**-3.25**
PSYR_1053	*algF*, alginate biosynthesis protein AlgF	**-3.29**
PSYR_4988	Rhs family protein	**-3.3**
PSYR_3469	glycosyl transferase family protein	**-3.36**
PSYR_4986	YD repeat-containing protein	**-3.4**
PSYR_3307	glycosyl transferase, group 1 family protein PslH	**-3.46**
PSYR_3308	glycosyl transferase, group 1 family protein PslI	**-3.5**
PSYR_3305	glycosyl transferase, group 1 family protein PslF	**-4.04**
PSYR_3088	group 1 glycosyl transferase	**-4.56**
PSYR_3131	RND family efflux transporter MFP subunit	**-4.87**

**Cell motility**		
PSYR_3449	flagellar hook-length control protein FliK	**-1**
PSYR_2446	chemotaxis protein methyltransferase CheR	**-1.11**
PSYR_0868	histidine kinase, HAMP region: chemotaxis sensory transducer	**-1.17**
PSYR_2188	histidine kinase	**-1.21**
PSYR_1304	chemotaxis protein CheW	**-1.24**
PSYR_3475	*flgG*, flagellar basal-body rod protein FlgG	**-1.27**
PSYR_3455	flagellar assembly protein H	**-1.33**
PSYR_3443	*fliQ*, flagellar biosynthetic protein FliQ	**-1.36**
PSYR_3474	*flgH*, flagellar L-ring protein FlgH	**-1.36**
PSYR_0783	*cheR-1*, chemotaxis protein methyltransferase CheR	**-1.38**
PSYR_3473	*flgI*, flagellar P-ring protein FlgI	**-1.43**
PSYR_3442	flagellar biosynthetic protein FliR	**-1.57**
PSYR_3472	peptidoglycan hydrolase FlgJ	**-1.61**
PSYR_3441	flagellar biosynthetic protein FlhB	**-1.74**
PSYR_3454	flagellum-specific ATP synthase FliI	**-1.76**
PSYR_1305	chemotaxis protein methyltransferase CheR	**-1.76**
PSYR_1511	type II secretion system protein E	**-1.93**
PSYR_1306	chemotaxis protein CheW	**-1.96**
PSYR_3453	flagellar biosynthesis chaperone	**-2.04**
PSYR_3481	flagellar basal-body rod protein FlgB	**-2.09**
PSYR_3466	*fliC*, flagellin	**-2.14**
PSYR_3480	flagellar basal body rod protein FlgC	**-2.18**
PSYR_3432	flagellar motor protein	**-2.25**
PSYR_3479	*flgD*, basal-body rod modification protein FlgD	**-2.42**
PSYR_0784	*cheW-1*, chemotaxis protein CheW	**-2.49**
PSYR_0796	*pilD*, type IV pilus prepilin peptidase PilD	**-2.69**
PSYR_3464	*fliD*, flagellar hook-associated protein FliD	**-2.84**
PSYR_3431	motB protein	**-2.91**
PSYR_3465	flagellin FlaG	**-2.98**
PSYR_0785	methyl-accepting chemotaxis protein	**-3.17**
PSYR_3470	*flgL*, flagellar hook-associated protein FlgL	**-3.26**
PSYR_3478	*flgE-1*, flagellar hook protein FlgE	**-3.39**
**Intracellular trafficking, secretion, and vesicular transport**		
PSYR_4183	*secG*, preprotein translocase subunit SecG	**1.85**
PSYR_1641	signal peptide peptidase SppA, 36K type	**1.74**
PSYR_3149	*gspG*, general secretion pathway protein G	**1.69**
PSYR_4643	conjugal transfer protein	**1.69**
PSYR_3390	*ccmD*, heme exporter protein CcmD	**1.61**
PSYR_3148	*gspH*, general secretion pathway protein H	**1.4**
PSYR_3151	*gspE*, general secretion pathway protein E	**1.36**
PSYR_3150	*gspF*,general secretion pathway protein F	**1.34**
PSYR_0410	hypothetical protein PSYR_0410	**1.18**
PSYR_4882	*secB*, protein-export protein SecB	**1.1**
PSYR_3147	general secretion pathway protein I	**1.09**
PSYR_3805	hypothetical protein PSYR_3805	**-1.15**
PSYR_1517	type II and III secretion system protein	**-1.41**
PSYR_0382	*tatC*, sec-independent protein translocase TatC	**-1.67**
PSYR_3077	Type I secretion outer membrane protein, TolC	**-1.67**

PSYR_1513	type II secretion system protein	**-1.71**
PSYR_1514	type II secretion system protein E	**-1.71**
PSYR_1512	prepilin	**-1.73**
PSYR_3454	flagellum-specific ATP synthase FliI	**-1.76**
PSYR_0798	type IV pilus biogenesis protein PilB	**-1.9**
PSYR_1511	type II secretion system protein E	**-1.93**
PSYR_0799	type IV pilus biogenesis protein	**-2.38**
PSYR_0796	*pilD*, type IV pilus prepilin peptidase PilD	**-2.69**
PSYR_4374	hypothetical protein PSYR_4374	**-2.71**
PSYR_3075	Type I secretion membrane fusion protein, HlyD	**-3.06**
PSYR_0797	*pilC*, type IV pilus biogenesis protein PilC	**-3.2**
PSYR_4960	hypothetical protein PSYR_4960	**-3.77**
**Posttranslational modification, protein turnover, chaperones**		
PSYR_4452	urease accessory protein UreF	**4.78**
PSYR_4451	urease accessory protein UreE	**4**
PSYR_1980	heat shock protein, Hsp20 family	**3.86**
PSYR_4454	urease accessory protein	**2.78**
PSYR_1594	FKBP-type peptidyl-prolyl cis-trans isomerase	**2.53**
PSYR_2083	hypothetical protein PSYR_2083	**2.41**
PSYR_0268	*dsbA*, thiol:disulfide interchange protein DsbA	**2.26**
PSYR_3700	lipoprotein	**1.85**
PSYR_3616	*htpX*, heat shock protein HtpX	**1.84**
PSYR_4203	*smpB*, SsrA-binding protein	**1.74**
PSYR_1641	signal peptide peptidase SppA, 36K type	**1.74**
PSYR_1016	*trxB*, thioredoxin reductase	**1.67**
PSYR_1289	*dsbC*, thiol:disulfide interchange protein DsbC	**1.63**
PSYR_3393	*ccmA*, heme exporter protein CcmA	**1.6**
PSYR_4073	chaperonin, 10 kDa	**1.56**
PSYR_3389	*ccmE*, cytochrome c-type biogenesis protein CcmE	**1.53**
PSYR_4883	glutaredoxin	**1.49**
PSYR_3392	*ccmB*, heme exporter protein CcmB	**1.41**
PSYR_2901	*ppiC-1*, peptidyl-prolyl cis-trans isomerase C	**1.32**
PSYR_2907	*ppiC-2*, peptidyl-prolyl cis-trans isomerase C	**1.29**
PSYR_4072	chaperonin, 60 kDa	**1.26**
PSYR_3391	*ccmC*, heme exporter protein CcmC	**1.25**
PSYR_4752	peptidase, M16 family	**1.25**
PSYR_2466	*yhgI*, yhgI protein	**1.24**
PSYR_1746	*tig*, trigger factor	**1.23**
PSYR_4130	trypsin domain-containing protein	**1.2**
PSYR_4398	*dsbD*, thiol:disulfide interchange protein DsbD	**1.19**
PSYR_1140	*dsbB*, disulfide oxidoreductase	**1.17**
PSYR_4118	*sspA*, stringent starvation protein A	**1.16**
PSYR_0300	*trx-2*, thioredoxin	**1.13**
PSYR_1844	hypothetical protein PSYR_1844	**1.12**
PSYR_4194	*dnaJ,* dnaJ protein	**1.07**
PSYR_1629	glutathione S-transferase family protein	**1.03**
PSYR_0984	lipoprotein	**1.02**
PSYR_3733	autotransporter	**-1.13**
PSYR_2195	urease accessory protein UreD	**-1.13**

PSYR_4977	ADP-ribosylglycohydrolase	**-1.22**
PSYR_4625	peptidyl-prolyl cis-trans isomerase SurA	**-1.37**
PSYR_3061	glutathione S-transferase	**-1.47**
PSYR_4567	hypothetical protein PSYR_4567	**-2.02**
PSYR_4092	glutathione S-transferase family protein	**-2.53**
PSYR_0796	*pilD*, type IV pilus prepilin peptidase PilD	**-2.69**
PSYR_4877	antioxidant, AhpC/Tsa family	**-3.13**
PSYR_4958	clpB protein	**-3.45**
**Energy production and conversion**		
PSYR_0629	aldehyde dehydrogenase family protein	**2.7**
PSYR_3196	*aceA*, isocitrate lyase	**2.34**
PSYR_5127	*atpB*, F0F1 ATP synthase subunit A	**1.98**
PSYR_5013	CAIB/BAIF family protein	**1.84**
PSYR_1011	oxidoreductase FAD/FMN-binding protein	**1.77**
PSYR_1375	*fdxA*, ferredoxin	**1.6**
PSYR_0091	succinate-semialdehyde dehydrogenase	**1.59**
PSYR_4851	oxidoreductase, FAD-binding protein	**1.58**
PSYR_2022	*gpsA*, glycerol-3-phosphate dehydrogenase (NAD(P)+)	**1.57**
PSYR_0168	*pckA*, phosphoenolpyruvate carboxykinase	**1.55**
PSYR_0539	oxidoreductase, aldo/keto reductase family	**1.55**
PSYR_0976	*mqo*, malate:quinone oxidoreductase	**1.52**
PSYR_0198	citrate transporter	**1.46**
PSYR_2006	succinate dehydrogenase, hydrophobic membrane anchor protein	**1.38**
PSYR_2005	*sdhC*, succinate dehydrogenase, cytochrome b556 subunit	**1.37**
PSYR_0456	cytochrome b561	**1.35**
PSYR_0232	hypothetical protein PSYR_0232	**1.32**
PSYR_0227	aldehyde dehydrogenase family protein	**1.27**
PSYR_1902	glycerophosphoryl diester phosphodiesterase	**1.26**
PSYR_0624	*ppa-1*, inorganic pyrophosphatase	**1.25**
PSYR_4733	betaine aldehyde dehydrogenase BADH	**1.24**
PSYR_1141	*cyoA*, cytochrome o ubiquinol oxidase subunit II	**1.13**
PSYR_1367	alcohol dehydrogenase, class III	**1.13**
PSYR_2973	lactoylglutathione lyase	**1.12**
PSYR_1153	oxidoreductase zinc-binding protein	**1.11**
PSYR_1992	oxidoreductase zinc-binding protein	**1.1**
PSYR_2007	succinate dehydrogenase, flavoprotein subunit	**1.1**
PSYR_2004	*gltA*, citrate synthase I	**1.06**
PSYR_4031	fumarate hydratase, class I	**1.06**
PSYR_2387	malate:quinone oxidoreductase	**1.04**
PSYR_4759	ferredoxin	**1.03**
PSYR_5125	*atpF*, F0F1 ATP synthase subunit B	**1.02**
PSYR_2533	oxidoreductase, FAD-binding	**1.02**
PSYR_2394	aldehyde dehydrogenase family protein	**1.01**
PSYR_0313	oxidoreductase zinc-binding protein	**1**
PSYR_2011	dihydrolipoamide dehydrogenase	**-1.02**
PSYR_2377	hypothetical protein PSYR_2377	**-1.03**
PSYR_2010	dihydrolipoamide succinyltransferase	**-1.03**
PSYR_2302	FAD linked oxidase domain-containing protein	**-1.03**
PSYR_3200	*nuoE*, NADH dehydrogenase subunit E	**-1.06**

PSYR_2330	oxidoreductase, FAD-binding protein	**-1.08**
PSYR_2190	*xenA*, xenobiotic reductase A	**-1.13**
PSYR_1481	*ppa-2,* inorganic pyrophosphatase	**-1.2**
PSYR_2672	citrate transporter	**-1.21**
PSYR_3201	NADH dehydrogenase I subunit F	**-1.27**
PSYR_1625	pyruvate dehydrogenase, E1 component	**-1.36**
PSYR_3905	glycerol kinase	**-1.38**
PSYR_3372	hypothetical protein PSYR_3372	**-1.47**
PSYR_3887	iron-sulfur cluster-binding protein	**-1.47**
PSYR_4718	glutathione-independent formaldehyde dehydrogenase	**-1.53**
PSYR_0428	hypothetical protein PSYR_0428	**-1.54**
PSYR_4782	NADH:flavin oxidoreductase/NADH oxidase family protein	**-1.67**
PSYR_0352	DszA family monooxygenase	**-1.76**
PSYR_2462	sulfite reductase	**-1.88**
PSYR_2278	luciferase family protein	**-1.95**
PSYR_3202	NADH dehydrogenase subunit G	**-2.1**
PSYR_2013	*sucD,* succinyl-CoA synthetase subunit alpha	**-2.31**
PSYR_2012	*sucC*, succinyl-CoA synthetase subunit beta	**-2.35**
PSYR_3203	NADH:ubiquinone oxidoreductase subunit H	**-2.36**
PSYR_0517	*aceF*, pyruvate dehydrogenase complex, E2 component, dihydrolipoamide acetyltransferase	**-2.41**
PSYR_3209	NADH:ubiquinone oxidoreductase subunit N	**-2.44**
PSYR_3205	NADH:ubiquinone oxidoreductase subunit J	**-2.56**
PSYR_3204	*nuoI*, NADH dehydrogenase subunit I	**-2.56**
PSYR_3206	NADH:ubiquinone oxidoreductase subunit K	**-2.71**
PSYR_3208	NADH:ubiquinone oxidoreductase subunit M	**-2.72**
PSYR_0333	hypothetical protein PSYR_0333	**-2.82**
PSYR_3207	NADH:ubiquinone oxidoreductase subunit L	**-2.88**
PSYR_3247	*ssuD*, alkanesulfonate monooxygenase	**-3.33**
**Carbohydrate transport and metabolism**		
PSYR_3578	major facilitator superfamily transporter	**2.13**
PSYR_0370	*fbp*, fructose-1,6-bisphosphatase	**1.81**
PSYR_2856	chitin-binding protein	**1.63**
PSYR_4487	carbohydrate kinase PfkB	**1.61**
PSYR_4847	*rpiA*, ribose 5-phosphate isomerase	**1.41**
PSYR_2908	phosphoglucomutase, alpha-D-glucose phosphate-specific	**1.39**
PSYR_1233	*suhB*, inositol-1-monophosphatase	**1.38**
PSYR_4611	*rpe,* ribulose-phosphate 3-epimerase	**1.35**
PSYR_0567	hypothetical protein PSYR_0567	**1.35**
PSYR_1712	hypothetical protein PSYR_1712	**1.29**
PSYR_1914	transaldolase	**1.17**
PSYR_2090	*ppsA*, phosphoenolpyruvate synthase	**1.13**
PSYR_2235	senescence marker protein-30 family protein	**1.12**
PSYR_4885	*gpmA*, phosphoglycerate mutase	**1.08**
PSYR_4184	*tpiA*, triosephosphate isomerase	**1.06**
PSYR_4792	*tkt*, transketolase	**1.05**
PSYR_1113	glucose ABC transporter, periplasmic glucose-binding protein	**1.04**
PSYR_1115	glucose ABC transporter permease	**-1.03**
PSYR_2298	glycosyl hydrolase family protein	**-1.03**
PSYR_3267	iolH protein	**-1.04**

PSYR_2513	putrescine ABC transporter periplasmic putrescine-binding protein	**-1.05**
PSYR_3263	sugar ABC transporter permease	**-1.05**
PSYR_4226	MFS transporter, phthalate permease family	**-1.05**
PSYR_1740	ABC transporter permease	**-1.06**
PSYR_1056	*algL*, alginate lyase	**-1.06**
PSYR_5042	major facilitator family transporter	**-1.07**
PSYR_2490	alpha-amylase family protein	**-1.08**
PSYR_0900	*dctP,* TRAP dicarboxylate transporter subunit DctP	**-1.08**
PSYR_2993	alpha-amylase family protein	**-1.08**
PSYR_2401	spermidine/putrescine ABC transporter periplasmic spermidine/putrescine-binding protein	**-1.09**
PSYR_0826	*pgi,* glucose-6-phosphate isomerase	**-1.09**
PSYR_2238	periplasmic substrate-binding protein	**-1.12**
PSYR_2491	*glgB,* 1,4-alpha-glucan-branching protein	**-1.2**
PSYR_3270	*iolI*, iolI protein	**-1.23**
PSYR_4899	MFS permease-like protein	**-1.23**
PSYR_1742	ABC transporter periplasmic substrate-binding protein	**-1.23**
PSYR_2884	*xylF*, D-xylose ABC transporter, periplasmic-D xylose binding protein	**-1.26**
PSYR_2997	*glgX*, glycogen operon protein GlgX	**-1.27**
PSYR_1116	*gltK*, glucose ABC transporter ATP-binding protein	**-1.28**
PSYR_2153	*rbsC-1*, ribose ABC transporter permease	**-1.33**
PSYR_2994	*malQ*, 4-alpha-glucanotransferase	**-1.34**
PSYR_2563	HAD family hydrolase	**-1.37**
PSYR_3673	alpha-ribazole-5\'-phosphate phosphatase	**-1.39**
PSYR_1898	membrane protein	**-1.42**
PSYR_2883	*xylA*, xylose isomerase	**-1.43**
PSYR_2885	*xylG*, xylose transporter ATP-binding subunit	**-1.44**
PSYR_1121	6-phosphogluconolactonase	**-1.45**
PSYR_2152	*rbsA-1*, ribose ABC transporter ATP-binding protein	**-1.45**
PSYR_2995	glycosyl hydrolase family protein	**-1.45**
PSYR_2291	aldolase II superfamily protein	**-1.47**
PSYR_2886	D-xylose ABC transporter permease	**-1.51**
PSYR_2186	senescence marker protein-30	**-1.55**
PSYR_2373	*araH*, L-arabinose transporter permease protein	**-1.61**
PSYR_1122	keto-hydroxyglutarate-aldolase/keto-deoxy-phosphogluconate aldolase	**-1.65**
PSYR_2588	HpcH/HpaI aldolase	**-1.69**
PSYR_1118	hypothetical protein PSYR_1118	**-1.71**
PSYR_2440	mannitol ABC transporter, periplasmic mannitol-binding protein	**-1.72**
PSYR_2923	carbohydrate kinase PfkB	**-1.74**
PSYR_2566	Glycerone kinase	**-1.75**
PSYR_4010	*pcaT,* dicarboxylic acid transport protein	**-1.78**
PSYR_1363	enolase	**-1.83**
PSYR_2569	periplasmic binding protein/LacI transcriptional regulator	**-1.83**
PSYR_2692	polysaccharide deacetylase	**-1.9**
PSYR_1989	galactonate dehydratase	**-1.94**
PSYR_1990	MFS transporter, phthalate permease family	**-2.05**
PSYR_2565	hypothetical protein PSYR_2565	**-2.27**
PSYR_4812	sugar ABC transporter periplasmic sugar-binding protein	**-2.97**
PSYR_3306	glycosyl hydrolase, family 5 PslG	**-2.99**

PSYR_2434	fructokinase	**-3.93**
PSYR_2437	ABC transporter	**-4.45**
PSYR_2436	*uxuB*, D-mannonate oxidoreductase	**-5.27**
PSYR_2435	*xylB*, xylulokinase	**-5.64**
**Amino acid transport and metabolism**		
PSYR_0678	beta-alanine--pyruvate aminotransferase	**2.93**
PSYR_4709	glycine betaine/L-proline ABC transporter, periplasmic substrate-binding protein	**2.36**
PSYR_2900	phospho-2-dehydro-3-deoxyheptonate aldolase	**2.33**
PSYR_4426	branched-chain amino acid ABC transporter substrate-binding protein	**2.13**
PSYR_0295	amino acid ABC transporter ATP-binding protein	**1.89**
PSYR_0557	ACT domain protein/phosphoserine phosphatase SerB	**1.75**
PSYR_0246	*gcvT-1*, glycine cleavage system T protein	**1.7**
PSYR_1235	*cysE*, serine O-acetyltransferase	**1.65**
PSYR_3580	aromatic amino acid permease	**1.49**
PSYR_0474	homoserine O-acetyltransferase	**1.38**
PSYR_1609	aspartyl aminopeptidase	**1.37**
PSYR_4852	*serA*, D-3-phosphoglycerate dehydrogenase	**1.36**
PSYR_4581	anthranilate synthase component II	**1.36**
PSYR_4609	*trpE*, anthranilate synthase, component I	**1.35**
PSYR_0476	*proC*, pyrroline-5-carboxylate reductase	**1.34**
PSYR_0576	ATP phosphoribosyltransferase regulatory subunit	**1.29**
PSYR_4834	proline-specific permease proY	**1.29**
PSYR_4580	anthranilate phosphoribosyltransferase	**1.28**
PSYR_4369	*proA*, gamma-glutamyl phosphate reductase	**1.25**
PSYR_1853	*aroC*, chorismate synthase	**1.21**
PSYR_2015	*brnQ*, branched-chain amino acid transport system II carrier protein	**1.17**
PSYR_0182	*lsyA-2*, diaminopimelate decarboxylase	**1.17**
PSYR_3747	*asnB*, asparagine synthetase	**1.17**
PSYR_3570	arginine/ornithine ABC transporter, permease protein	**1.16**
PSYR_4894	*hisA*, phosphoribosylformimino-5-aminoimidazole carboxamide ribotide isomerase	**1.14**
PSYR_1336	tetrahydrodipicolinate succinylase	**1.09**
PSYR_0090	*gabT-2*, 4-aminobutyrate aminotransferase	**1.08**
PSYR_3024	peptidase M14, carboxypeptidase A	**1.08**
PSYR_3742	Bifunctional,tRNA-methyltransferase/FAD-dependent oxidoreductase	**1.04**
PSYR_1973	*aspC*, aspartate aminotransferase	**1.03**
PSYR_3931	hypothetical protein PSYR_3931	**1.01**
PSYR_4436	urease subunit alpha	**-1**
PSYR_4713	*soxB-1*, sarcosine oxidase, beta subunit	**-1.01**
PSYR_3269	iolD protein	**-1.01**
PSYR_2941	glutamine/glutamate ABC transporter periplasmic glutamine/glutamate-binding protein	**-1.03**
PSYR_2888	amine oxidase, flavin-containing protein	**-1.03**
PSYR_4211	peptide ABC transporter substrate-binding protein	**-1.03**
PSYR_2855	5-methyltetrahydropteroyltriglutamate/homocysteine S-methyltransferase	**-1.03**
PSYR_4715	*soxA-1*, sarcosine oxidase, alpha subunit	**-1.04**
PSYR_4712	*glyA-1*, serine hydroxymethyltransferase	**-1.05**
PSYR_1672	*ilvA-1*, threonine dehydratase	**-1.05**

PSYR_4321	pyridoxal-5\'-phosphate-dependent enzyme, beta subunit	**-1.05**
PSYR_2538	peptide ABC transporter permease	**-1.06**
PSYR_0846	acetolactate synthase large subunit	**-1.08**
PSYR_4132	histidinol-phosphate aminotransferase	**-1.08**
PSYR_4716	*soxG-1*, sarcosine oxidase, gamma subunit	**-1.08**
PSYR_4612	polyamine ABC transporter permease	**-1.1**
PSYR_0599	*braE*, high-affinity branched-chain amino acid ABC transporter, permease protein BraE	**-1.12**
PSYR_3645	chorismate mutase/prephenate dehydratase	**-1.13**
PSYR_4675	peptidase S9, prolyl oligopeptidase active site region	**-1.14**
PSYR_0974	amino acid ABC transporter substrate-binding protein	**-1.15**
PSYR_2223	*soxD-2*, sarcosine oxidase subunit delta	**-1.17**
PSYR_0117	class I and II aminotransferase	**-1.21**
PSYR_4784	renal dipeptidase family protein	**-1.22**
PSYR_2585	Orn/DAP/Arg decarboxylase 2:Orn/DAP/Arg decarboxylase 2	**-1.22**
PSYR_2619	diaminobutyrate-2-oxoglutarate transaminase	**-1.24**
PSYR_3158	sarcosine oxidase	**-1.25**
PSYR_4238	*dppA*, dipeptide ABC transporter substrate-binding protein	**-1.28**
PSYR_2055	transglutaminase-like domain protein	**-1.29**
PSYR_2604	hypothetical protein PSYR_2604	**-1.29**
PSYR_1698	glutathionylspermidine synthase	**-1.31**
PSYR_1156	hypothetical protein PSYR_1156	**-1.31**
PSYR_1257	*leuA*, 2-isopropylmalate synthase	**-1.31**
PSYR_2542	peptide ABC transporter periplasmic peptide-binding protein	**-1.32**
PSYR_2539	peptide ABC transporter permease	**-1.34**
PSYR_4236	dipeptide ABC transporter substrate-binding protein	**-1.35**
PSYR_0691	gamma-glutamyltranspeptidase	**-1.41**
PSYR_0600	high affinity branched-chain amino acid ABC transporter ATP-binding protein	**-1.42**
PSYR_0973	amino acid ABC transporter permease	**-1.44**
PSYR_4863	*potG*, putrescine ABC transporter ATP-binding protein	**-1.5**
PSYR_0601	high affinity branched-chain amino acid ABC transporter ATP-binding protein	**-1.52**
PSYR_0847	acetolactate synthase small subunit	**-1.54**
PSYR_3555	aspartate kinase	**-1.56**
PSYR_2540	peptide ABC transporter ATP-binding protein	**-1.57**
PSYR_0412	glutamate synthase, small subunit	**-1.61**
PSYR_2745	pyridoxal-phosphate dependent enzyme family/ornithine cyclodeaminase family protein	**-1.62**
PSYR_1075	amino acid ABC transporter ATP-binding protein	**-1.71**
PSYR_3220	cysteine desulfurase	**-1.73**
PSYR_4235	dipeptide ABC transporter, periplasmic dipeptide-binding protein	**-1.75**
PSYR_0356	putative amino-acid ABC transporter ATP-binding protein YecC	**-1.79**
PSYR_4234	peptide ABC transporter periplasmic peptide-binding protein	**-1.85**
PSYR_0848	ketol-acid reductoisomerase	**-1.97**
PSYR_2398	spermidine/putrescine ABC transporter ATP-binding protein	**-1.98**
PSYR_4093	glutamate N-acetyltransferase/amino-acid acetyltransferase	**-2.06**
PSYR_1074	amino acid ABC transporter permease	**-2.15**
PSYR_2567	zinc-containing alcohol dehydrogenase superfamily protein	**-2.17**
PSYR_1758	ABC transporter substrate-binding protein	**-2.26**
PSYR_1073	amino acid ABC transporter permease	**-2.3**

PSYR_3846	high-affinity branched-chain amino acid ABC transporter permease	**-2.33**
PSYR_2964	amino acid ABC transporter permease	**-2.33**
PSYR_2399	spermidine/putrescine ABC transporter permease	**-2.34**
PSYR_1340	class I and II aminotransferase	**-2.63**
PSYR_3847	leucine/isoleucine/valine transporter permease subunit	**-2.73**
PSYR_3849	high-affinity amino acid ABC transporter, ATP-binding protein	**-2.99**
PSYR_3848	leucine/isoleucine/valine transporter ATP-binding subunit	**-3.08**
PSYR_2962	extracellular solute-binding protein	**-3.13**
PSYR_2965	amino acid ABC transporter ATP-binding protein	**-3.52**
**Nucleotide transport and metabolism**		
PSYR_4453	urease accessory protein UreG	**4.33**
PSYR_1728	tRNA--hydroxylase	**2.26**
PSYR_0661	adenosine deaminase	**1.94**
PSYR_1836	*pyrF*, orotidine 5-phosphate decarboxylase	**1.7**
PSYR_1319	*adk*, adenylate kinase	**1.6**
PSYR_4840	*thyA*, thymidylate synthase	**1.55**
PSYR_0483	pyrimidine operon regulatory protein PyrR	**1.48**
PSYR_3689	*purM*,phosphoribosylformylglycinamidine cyclo-ligase	**1.43**
PSYR_4192	*carA*, carbamoyl-phosphate synthase small subunit	**1.16**
PSYR_1277	*purT*, phosphoribosylglycinamide formyltransferase 2	**1.14**
PSYR_0216	*pyrE*, orotate phosphoribosyltransferase	**1.08**
PSYR_4018	*purU-3*, formyltetrahydrofolate deformylase	**1.01**
PSYR_4629	bis(5\'-nucleosyl)-tetraphosphatase, symmetrical	**-1.13**
PSYR_4407	phosphoribosylamine--glycine ligase	**-1.6**
PSYR_3531	cytosine/purines uracil thiamine allantoin permease	**-1.62**
PSYR_3717	*nrdA*, ribonucleotide-diphosphate reductase subunit alpha	**-2.02**
PSYR_3695	*relA*, GTP pyrophosphokinase	**-5.22**
**Coenzyme transport and metabolism**		
PSYR_0389	*ubiE*, ubiquinone/menaquinone biosynthesis methyltransferase UbiE	**1.96**
PSYR_1542	*nadA*, quinolinate synthetase	**1.74**
PSYR_4341	*thiE*, thiamine-phosphate pyrophosphorylase	**1.74**
PSYR_4342	*hemL*, glutamate-1-semialdehyde-2,1-aminomutase	**1.7**
PSYR_1333	ThiF family protein	**1.62**
PSYR_1743	*folD-2,*methylenetetrahydrofolate,dehydrogenase/ methenyltetrahydrofolate cyclohydrolase	**1.53**
PSYR_0317	5-formyltetrahydrofolate cyclo-ligase	**1.52**
PSYR_0604	*dxs*, deoxyxylulose-5-phosphate synthase	**1.51**
PSYR_4574	hypothetical protein PSYR_4574	**1.49**
PSYR_0967	*hemH*, ferrochelatase	**1.49**
PSYR_3558	6,7-dimethyl-8-ribityllumazine synthase	**1.45**
PSYR_5031	*ubiA*, 4-hydroxybenzoate octaprenyltransferase	**1.37**
PSYR_4581	anthranilate synthase component II	**1.36**
PSYR_1845	*folE-2*, GTP cyclohydrolase I	**1.34**
PSYR_1418	radical SAM domain-containing protein	**1.32**
PSYR_4816	*thiI*, thiamin biosynthesis protein ThiI	**1.32**
PSYR_4340	bifunctional hydroxy-methylpyrimidine kinase/ hydroxy-phosphomethylpyrimidine kinase	**1.28**
PSYR_0460	*ahcY*, adenosylhomocysteinase	**1.27**
PSYR_0709	*ribF*, riboflavin biosynthesis protein RibF	**1.26**
PSYR_0815	*nadC*, nicotinate-nucleotide pyrophosphorylase	**1.25**

PSYR_0595	*pncB*, nicotinate phosphoribosyltransferase	**1.25**
PSYR_4461	*ribE*, riboflavin synthase subunit alpha	**1.24**
PSYR_0024	*hemF*, coproporphyrinogen III oxidase	**1.22**
PSYR_2098	uroporphyrin-III C-methyltransferase	**1.22**
PSYR_0292	delta-aminolevulinic acid dehydratase	**1.21**
PSYR_0487	*gshB*, glutathione synthetase	**1.17**
PSYR_2135	molybdopterin-guanine dinucleotide biosynthesis protein MobA	**1.13**
PSYR_1828	*pdxB*, erythronate-4-phosphate dehydrogenase	**1.12**
PSYR_4737	*folA*, dihydrofolate reductase	**1.12**
PSYR_3681	*cobO*, cob(I)alamin adenosyltransferasehrpA	**1.11**
PSYR_5030	chorismate-pyruvate lyase	**1.11**
PSYR_1021	dihydromonapterin reductase	**1.1**
PSYR_4758	pantetheine-phosphate adenylyltransferase	**1.09**
PSYR_4368	*nadD*, nicotinate (nicotinamide) nucleotide adenylyltransferase	**1.08**
PSYR_0700	*ispB*, octylprenyl diphosphate synthase	**1.05**
PSYR_0948	*hemA*, glutamyl-tRNA reductase	**1.04**
PSYR_0828	*panB*, 3-methyl-2-oxobutanoate hydroxymethyltransferase	**1.03**
PSYR_4418	*cobL*, precorrin-6Y C5,15-methyltransferase	**1**
PSYR_0537	lipopolysaccharide biosynthesis protein RfaE	**1**
PSYR_3654	hypothetical protein PSYR_3654	**-1.01**
PSYR_2377	hypothetical protein PSYR_2377	**-1.03**
PSYR_3672	*cobS*, cobalamin (5\'-phosphate) synthase	**-1.04**
PSYR_3674	*cobT*,nicotinate-nucleotide--dimethylbenzimidazole phosphoribosyltransferase	**-1.08**
PSYR_3015	cobaltochelatase subunit CobN	**-1.18**
PSYR_3013	magnesium chelatase subunit ChlD	**-1.19**
PSYR_2514	ABC transporter ATP-binding protein	**-1.2**
PSYR_4686	8-amino-7-oxononanoate synthase	**-1.24**
PSYR_3014	magnesium chelatase, subunit ChlI	**-1.28**
PSYR_3531	cytosine/purines uracil thiamine allantoin permease	**-1.62**
PSYR_4684	biotin synthesis protein BioC	**-2.2**
PSYR_4683	dethiobiotin synthetase	**-2.39**
PSYR_0544	*thiC*, thiamin biosynthesis protein ThiC	**-2.44**
PSYR_4626	pyridoxal phosphate biosynthetic protein PdxA	**-2.56**
PSYR_0334	L-ectoine synthase	**-3.37**
PSYR_4873	molybdenum-pterin binding domain-containing protein	**-4.74**
**Lipid transport and metabolism**		
PSYR_3572	*acs*, acetyl-CoA synthetase	**5.01**
PSYR_3237	3-oxoacid CoA-transferase, subunit A family	**4.41**
PSYR_3238	3-oxoacid CoA-transferase, subunit B family	**3.96**
PSYR_3239	short-chain fatty acid transporter	**2.7**
PSYR_0706	acyltransferase 3	**2.13**
PSYR_0087	fatty acid desaturase	**2.06**
PSYR_0436	acyl carrier protein	**1.83**
PSYR_2465	acetyltransferase family protein	**1.7**
PSYR_0229	phospholipase D	**1.62**
PSYR_0437	acyl carrier protein	**1.61**
PSYR_1369	*ispF*, 2-C-methyl-D-erythritol 2,4-cyclodiphosphate synthase	**1.52**
PSYR_2019	*fabB*, 3-oxoacyl-(acyl carrier protein) synthase I	**1.49**
PSYR_3300	lipid kinase	**1.49**

PSYR_2467	biotin carboxylase	**1.38**
PSYR_2020	*fabA*, 3-hydroxydecanoyl-(acyl carrier protein) dehydratase	**1.36**
PSYR_0645	acetyl-CoA acetyltransferase	**1.34**
PSYR_3003	azoreductase	**1.31**
PSYR_4724	putative acyltransferase	**1.2**
PSYR_1892	3-ketoacyl-(acyl-carrier-protein) reductase	**1.1**
PSYR_0945	*ispE*, 4-diphosphocytidyl-2-C-methyl-D-erythritol kinase	**1.06**
PSYR_0438	acyltransferase family protein	**1.03**
PSYR_4293	*tesB*, acyl-CoA thioesterase II	**1**
PSYR_1703	Fatty acid desaturase	**-1.03**
PSYR_3289	3-ketoacyl-CoA thiolase	**-1.11**
PSYR_2725	thiolase family protein	**-1.11**
PSYR_3669	outer membrane protein	**-1.2**
PSYR_4606	autotransporting lipase, GDSL family	**-1.23**
PSYR_0424	hypothetical protein PSYR_0424	**-1.4**
PSYR_1538	propionyl-CoA carboxylase	**-1.44**
PSYR_1349	1-deoxy-D-xylulose 5-phosphate reductoisomerase	**-1.46**
PSYR_4685	bioH, bioH protein	**-1.5**
PSYR_0425	3-oxoacyl-(acyl carrier protein) synthase I	**-1.56**
PSYR_0353	acyl-CoA dehydrogenase family protein	**-1.65**
PSYR_4311	malonyl CoA-ACP transacylase	**-1.87**
PSYR_3604	acyl-CoA dehydrogenase family protein	**-2.13**
PSYR_0331	AMP-dependent synthetase and ligase	**-2.51**
PSYR_0354	acyl-CoA dehydrogenase family protein	**-3.02**
**Inorganic ion transport and metabolism**		
PSYR_0656	copZ protein	**4.38**
PSYR_0654	copper-translocating P-type ATPase	**3.45**
PSYR_3897	*bfr*, bacterioferritin	**2.81**
PSYR_0863	alkylphosphonate utilization operon protein PhnA	**2.79**
PSYR_0189	ammonium transporter	**2.7**
PSYR_0275	*znuA*, zinc ABC transporter periplasmic zinc-binding protein	**1.94**
PSYR_5034	transporter	**1.84**
PSYR_4884	rhodanese-like domain-containing protein	**1.79**
PSYR_0244	iron ABC transporter periplasmic iron-binding protein	**1.79**
PSYR_0281	metN-2, DL-methionine transporter ATP-binding subunit	**1.62**
PSYR_2756	*modA*, molybdate ABC transporter periplasmic molybdate-binding protein	**1.49**
PSYR_4827	choline transporter	**1.37**
PSYR_0282	*metI-2*, D-methionine ABC transporter permease	**1.35**
PSYR_4210	*nhaA-1*, sodium-proton antiporter NhaA	**1.33**
PSYR_0657	Bcr/CflA family multidrug resistance transporter	**1.32**
PSYR_3094	iron ABC transporter, periplasmic iron-binding protein	**1.3**
PSYR_4016	phosphate transporter family protein	**1.3**
PSYR_0283	metQ-2, D-methionine-binding lipoprotein MetQ	**1.26**
PSYR_0667	*fecB*, iron(III) dicitrate transport system, periplasmic iron-binding protein FecB	**1.23**
PSYR_2078	phosphoadenosine phosphosulfate reductase	**1.23**
PSYR_4243	iron-uptake factor	**1.15**
PSYR_1337	arsC family protein	**1.14**
PSYR_3702	*arsC*, arsenate reductase	**1.12**

PSYR_0245	iron ABC transporter permease	**1.06**
PSYR_3171	DsrC-like protein	**1.06**
PSYR_0280	*katE*, catalase	**1.06**
PSYR_3170	DsrH family protein	**1.02**
PSYR_0695	phosphonate ABC transporter periplasmic phosphonate-binding protein	**1.01**
PSYR_2139	glutathione-regulated potassium-efflux system protein	**1**
PSYR_2546	DMT superfamily multiple drug efflux pump	**-1.02**
PSYR_2603	secretion protein HlyD	**-1.02**
PSYR_0156	peptide ABC transporter permease	**-1.05**
PSYR_2538	peptide ABC transporter permease	**-1.06**
PSYR_1962	TonB-dependent siderophore receptor	**-1.06**
PSYR_2525	transporter, partial	**-1.08**
PSYR_4613	polyamine ABC transporter permease	**-1.08**
PSYR_1606	ABC transporter substrate-binding protein	**-1.09**
PSYR_2537	hypothetical protein PSYR_2537	**-1.15**
PSYR_0328	lipoprotein, NLPA family	**-1.15**
PSYR_4628	apaG protein	**-1.18**
PSYR_2904	peptide ABC transporter permease	**-1.22**
PSYR_2586	EmrB/QacA family drug resistance transporter	**-1.23**
PSYR_1951	cation ABC transporter substrate-binding protein	**-1.24**
PSYR_2329	extracellular solute-binding domain protein	**-1.26**
PSYR_3106	*pstS*, phosphate ABC transporter substrate-binding protein	**-1.27**
PSYR_5039	phosphate ABC transporter permease	**-1.3**
PSYR_2372	L-arabinose transporter ATP-binding protein	**-1.3**
PSYR_0346	AcrB/AcrD/AcrF family protein	**-1.31**
PSYR_4126	*cysN/C*, bifunctional sulfate adenylyltransferase subunit 1/adenylylsulfate kinase protein	**-1.31**
PSYR_2539	peptide ABC transporter permease	**-1.34**
PSYR_2960	nickel ABC transporter, permease protein	**-1.37**
PSYR_0082	sulfate ABC transporter permease CysW	**-1.41**
PSYR_0081	sulfate ABC transporter, ATP-binding protein CysA	**-1.43**
PSYR_1949	cation ABC transporter permease	**-1.47**
PSYR_1948	ABC transporter periplasmic substrate-binding protein	**-1.49**
PSYR_1950	cation ABC transporter ATP-binding protein	**-1.5**
PSYR_0083	*cysT*, sulfate ABC transporter permease CysT	**-1.52**
PSYR_0349	D-methionine ABC transporter permease	**-1.55**
PSYR_4208	catalase/peroxidase HPI	**-1.57**
PSYR_2540	peptide ABC transporter ATP-binding protein	**-1.57**
PSYR_3603	ABC transporter, periplasmic substrate-binding protein, aliphatic sulfonates family	**-1.66**
PSYR_0350	D-methionine ABC transporter ATP-binding protein	**-1.9**
PSYR_4826	TonB-dependent receptor:TonB box, N-terminal, partial	**-1.97**
PSYR_0339	ABC transporter permease	**-1.99**
PSYR_0084	*sbp*, sulfate-binding protein	**-2.15**
PSYR_3602	rhodanese-like domain-containing protein	**-2.29**
PSYR_4861	*potI*, putrescine ABC transporter permease	**-2.29**
PSYR_0692	phosphonate ABC transporter, permease protein	**-2.3**
PSYR_4862	*potH*, putrescine ABC transporter permease	**-2.32**
PSYR_2400	spermidine/putrescine ABC transporter permease	**-2.35**
PSYR_1757	ABC transporter permease	**-2.7**

PSYR_0338	ABC transporter ATP-binding protein	**-2.86**
PSYR_0340	ABC transporter substrate-binding protein	**-2.9**
PSYR_4876	sulfonate ABC transporter periplasmic sulfonate-binding protein	**-3.55**
PSYR_1756	ABC transporter permease	**-3.58**
PSYR_3132	AcrB/AcrD/AcrF family protein	**-3.58**
PSYR_2439	mannitol ABC transporter permease	**-3.81**
PSYR_4875	*ssuC*, aliphatic sulfonates ABC transporter permease	**-3.95**
PSYR_4874	aliphatic sulfonates transport ATP-binding subunit	**-4.04**
PSYR_2438	mannitol ABC transporter permease	**-4.23**
**Secondary metabolites biosynthesis, transport and catabolism**		
PSYR_3785	DSBA oxidoreductase	**2**
PSYR_0436	acyl carrier protein	**1.83**
PSYR_1849	isochorismatase family protein	**1.73**
PSYR_4583	lysozyme	**1.65**
PSYR_1493	twin-arginine translocation pathway signal:copper-resistance protein CopA	**1.62**
PSYR_0437	acyl carrier protein	**1.61**
PSYR_0290	enhancing lycopene biosynthesis protein 2	**1.53**
PSYR_4139	mce-like protein	**1.44**
PSYR_2066	permease	**1.4**
PSYR_4138	toluene tolerance protein	**1.31**
PSYR_3988	hypothetical protein PSYR_3988	**1.31**
PSYR_2067	ABC transporter ATP-binding protein	**1.25**
PSYR_4141	toluene tolerance ABC transporter, ATP-binding protein	**1.12**
PSYR_4140	membrane protein	**1.12**
PSYR_1892	3-ketoacyl-(acyl-carrier-protein) reductase	**1.1**
PSYR_3326	*hmgA*, homogentisate 1,2-dioxygenase	**1.07**
PSYR_1762	hypothetical protein PSYR_1762	**1.03**
PSYR_3657	DSBA oxidoreductase	**1**
PSYR_2726	*fcs*, feruloyl-CoA synthase	**-1.07**
PSYR_2584	IucA/IucC protein	**-1.1**
PSYR_4314	beta-ketoacyl synthase	**-1.21**
PSYR_3983	serralysin	**-1.21**
PSYR_4312	erythronolide synthase	**-1.26**
PSYR_2534	dienelactone hydrolase family protein	**-1.27**
PSYR_2615	amino acid adenylation	**-1.3**
PSYR_2587	IucA/IucC protein	**-1.33**
PSYR_2611	amino acid adenylation	**-1.35**
PSYR_2616	amino acid adenylation	**-1.39**
PSYR_2614	amino acid adenylation	**-1.39**
PSYR_4313	beta-ketoacyl synthase	**-1.39**
PSYR_4310	hypothetical protein PSYR_4310	**-1.52**
PSYR_2746	L-lysine 6-monooxygenase	**-1.58**
PSYR_1795	taurine dioxygenase	**-1.58**
PSYR_1792	amino acid adenylation	**-1.63**
PSYR_4311	malonyl CoA-ACP transacylase	**-1.87**
PSYR_2608	amino acid adenylation	**-1.93**
PSYR_1793	amino acid adenylation	**-2.15**
PSYR_3074	hemolysin-type calcium-binding protein	**-2.3**
PSYR_1794	amino acid adenylation	**-2.37**

PSYR_0337	dioxygenase, TauD/TfdA family	**-2.82**
PSYR_3089	hemolysin-type calcium-binding protein	**-3.53**
**Funcation unknow**		
PSYR_3757	hypothetical protein PSYR_3757	**6.43**
PSYR_3756	sodium:solute symporter family protein	**5.86**
PSYR_3668	hypothetical protein PSYR_3668	**3.98**
PSYR_4175	transport-associated protein	**3.62**
PSYR_4645	prevent-host-death family protein	**3.43**
PSYR_0744	hypothetical protein	**3.14**
PSYR_0508	Lyase	**3.1**
PSYR_0260	hypothetical protein PSYR_0260	**3.05**
PSYR_4644	plasmid stabilization system protein	**3.04**
PSYR_0745	hypothetical protein	**2.93**
PSYR_3971	lipoprotein	**2.92**
PSYR_0207	lipoprotein	**2.91**
PSYR_3966	hypothetical protein PSYR_3966	**2.87**
PSYR_4745	hypothetical protein PSYR_4745	**2.79**
PSYR_5020	hypothetical protein PSYR_5020	**2.73**
PSYR_4272	hypothetical protein PSYR_4272	**2.65**
PSYR_2497	lipoprotein	**2.65**
PSYR_3261	chemotaxis sensory transducer protein	**2.49**
PSYR_2324	hypothetical protein PSYR_2324	**2.49**
PSYR_1859	hypothetical protein PSYR_1859	**2.44**
PSYR_3034	hypothetical protein PSYR_3034	**2.38**
PSYR_2348	hypothetical protein PSYR_2348	**2.38**
PSYR_3786	RNA binding S1	**2.36**
PSYR_4605	transcriptional regulator PrtN	**2.35**
PSYR_2687	PepSY-associated TM helix family protein	**2.34**
PSYR_4604	hypothetical protein PSYR_4604	**2.26**
PSYR_1381	hypothetical protein PSYR_1381	**2.22**
PSYR_0743	hypothetical protein	**2.16**
PSYR_4744	hypothetical protein PSYR_4744	**2.13**
PSYR_4328	hypothetical protein PSYR_4328	**2.1**
PSYR_2140	hypothetical protein PSYR_2140	**2.09**
PSYR_3699	hypothetical protein PSYR_3699	**2.08**
PSYR_0369	lipoprotein	**2.08**
PSYR_3967	membrane protein TctB	**2.08**
PSYR_4704	hypothetical protein PSYR_4704	**2.07**
PSYR_3755	hypothetical protein PSYR_3755	**2.07**
PSYR_0467	hypothetical protein PSYR_0467	**2.07**
PSYR_2149	hypothetical protein PSYR_2149	**2.06**
PSYR_4722	hypothetical protein PSYR_4722	**2.04**
PSYR_4597	hypothetical protein PSYR_4597	**1.98**
PSYR_3617	thiopurine S-methyltransferase	**1.96**
PSYR_0214	hypothetical protein PSYR_0214	**1.95**
PSYR_4772	hypothetical protein PSYR_4772	**1.94**
PSYR_0269	ribosome biosis GTP-binding protein YsxC	**1.91**
PSYR_1847	Smr protein/MutS2 C-terminal, partial	**1.85**
PSYR_4076	hypothetical protein PSYR_4076	**1.85**

PSYR_3884	cold-shock protein, DNA-binding	**1.84**
PSYR_1998	lipoprotein	**1.82**
PSYR_0318	hypothetical protein PSYR_0318	**1.82**
PSYR_5066	import inner membrane translocase, subunit Tim44	**1.81**
PSYR_0702	50S ribosomal protein L27	**1.79**
PSYR_3968	hypothetical protein PSYR_3968	**1.79**
PSYR_1640	HAD family hydrolase	**1.78**
PSYR_2495	ABC transporter	**1.76**
PSYR_0191	hypothetical protein PSYR_0191	**1.76**
PSYR_1848	hypothetical protein PSYR_1848	**1.76**
PSYR_1766	hypothetical protein PSYR_1766	**1.75**
PSYR_1092	permease YjgP/YjgQ	**1.74**
PSYR_3929	hypothetical protein PSYR_3929	**1.72**
PSYR_0546	lipoprotein	**1.71**
PSYR_3038	hypothetical protein PSYR_3038	**1.68**
PSYR_0052	ABC transporter	**1.68**
PSYR_0980	phospholipid/glycerol acyltransferase	**1.68**
PSYR_1577	hypothetical protein PSYR_1577	**1.67**
PSYR_3658	hypothetical protein PSYR_3658	**1.67**
PSYR_1382	hypothetical protein PSYR_1382	**1.65**
PSYR_1821	N-acetyltransferase GCN5	**1.65**
PSYR_3620	hypothetical protein PSYR_3620	**1.64**
PSYR_3851	hypothetical protein PSYR_3851	**1.64**
PSYR_1626	Pirin, N-terminal:Pirin, C-terminal, partial	**1.63**
PSYR_4705	hypothetical protein PSYR_4705	**1.63**
PSYR_4942	HAD family hydrolase	**1.61**
PSYR_0435	hypothetical protein PSYR_0435	**1.61**
PSYR_3922	hypothetical protein PSYR_3922	**1.61**
PSYR_3210	hypothetical protein PSYR_3210	**1.6**
PSYR_4087	nucleotide-binding protein	**1.57**
PSYR_0966	hypothetical protein PSYR_0966	**1.57**
PSYR_2393	flavin reductase-like protein	**1.57**
PSYR_1589	hypothetical protein PSYR_1589	**1.56**
PSYR_4906	lipoprotein	**1.55**
PSYR_3294	ABC transporter	**1.53**
PSYR_4367	Iojap-related protein	**1.53**
PSYR_0187	hypothetical protein PSYR_0187	**1.53**
PSYR_4797	hypothetical protein PSYR_4797	**1.53**
PSYR_5055	YeeE/YedE	**1.52**
PSYR_1765	N-acetyltransferase GCN5	**1.52**
PSYR_3921	hypothetical protein PSYR_3921	**1.52**
PSYR_3783	hypothetical protein PSYR_3783	**1.5**
PSYR_4257	hypothetical protein PSYR_4257	**1.49**
PSYR_0248	hypothetical protein PSYR_0248	**1.49**
PSYR_3784	hypothetical protein PSYR_3784	**1.48**
PSYR_2082	hypothetical protein PSYR_2082	**1.47**
PSYR_2023	hypothetical protein PSYR_2023	**1.47**
PSYR_2518	hypothetical protein PSYR_2518	**1.47**
PSYR_4298	hypothetical protein PSYR_4298	**1.47**

PSYR_1578	hypothetical protein PSYR_1578	**1.46**
PSYR_3618	catalytic LigB subunit of aromatic ring-opening dioxygenase	**1.46**
PSYR_4598	lipoprotein	**1.46**
PSYR_1576	glutamine amidotransferase, class-II	**1.45**
PSYR_3766	hypothetical protein PSYR_3766	**1.45**
PSYR_2159	lipoprotein	**1.45**
PSYR_0626	hypothetical protein PSYR_0626	**1.44**
PSYR_4356	hypothetical protein PSYR_4356	**1.44**
PSYR_3577	hypothetical protein PSYR_3577	**1.44**
PSYR_0563	ribosome-associated GTPase	**1.44**
PSYR_0882	hypothetical protein PSYR_0882	**1.44**
PSYR_1852	hypothetical protein PSYR_1852	**1.43**
PSYR_3740	hypothetical protein PSYR_3740	**1.42**
PSYR_4074	FxsA protein	**1.42**
PSYR_0279	lipoprotein	**1.41**
PSYR_0703	GTPase ObgE	**1.41**
PSYR_1860	ankyrin	**1.41**
PSYR_4582	hypothetical protein PSYR_4582	**1.41**
PSYR_2420	hypothetical protein PSYR_2420	**1.4**
PSYR_3703	flavodoxin/nitric oxide synthase	**1.4**
PSYR_1124	hypothetical protein PSYR_1124	**1.4**
PSYR_1575	hypothetical protein PSYR_1575	**1.39**
PSYR_3928	hypothetical protein PSYR_3928	**1.37**
PSYR_2064	hypothetical protein PSYR_2064	**1.36**
PSYR_0727	hypothetical protein PSYR_0727	**1.36**
PSYR_2070	ErfK/YbiS/YcfS/YnhG	**1.36**
PSYR_4366	rRNA large subunit methyltransferase	**1.36**
PSYR_0319	hypothetical protein PSYR_0319	**1.36**
PSYR_2932	hypothetical protein PSYR_2932	**1.36**
PSYR_2381	Allergen V5/Tpx-1 related	**1.36**
PSYR_4596	hypothetical protein PSYR_4596	**1.36**
PSYR_4344	hypothetical protein PSYR_4344	**1.35**
PSYR_0192	hypothetical protein PSYR_0192	**1.35**
PSYR_1785	deoxyribodipyrimidine photolyase-like protein	**1.35**
PSYR_4839	hypothetical protein PSYR_4839	**1.34**
PSYR_5092	histidine kinase, HAMP region: chemotaxis sensory transducer	**1.34**
PSYR_0457	hypothetical protein PSYR_0457	**1.34**
PSYR_2386	hypothetical protein PSYR_2386	**1.34**
PSYR_4422	hypothetical protein PSYR_4422	**1.33**
PSYR_1564	hypothetical protein PSYR_1564	**1.32**
PSYR_0944	ribose-phosphate pyrophosphokinase	**1.31**
PSYR_2337	hypothetical protein PSYR_2337	**1.31**
PSYR_2347	hypothetical protein PSYR_2347	**1.3**
PSYR_1866	hypothetical protein PSYR_1866	**1.3**
PSYR_1297	YaeQ protein	**1.29**
PSYR_0291	hypothetical protein PSYR_0291	**1.29**
PSYR_0963	hypothetical protein PSYR_0963	**1.29**
PSYR_3753	short chain dehydrogenase	**1.29**
PSYR_4586	tail protein	**1.29**

PSYR_4455	hypothetical protein PSYR_4455	**1.28**
PSYR_4764	hypothetical protein PSYR_4764	**1.27**
PSYR_4610	phosphoglycolate phosphatase	**1.27**
PSYR_3660	hypothetical protein PSYR_3660	**1.27**
PSYR_4296	hypothetical protein PSYR_4296	**1.27**
PSYR_3844	hypothetical protein PSYR_3844	**1.26**
PSYR_4112	hypothetical protein PSYR_4112	**1.26**
PSYR_3164	hypothetical protein PSYR_3164	**1.25**
PSYR_0231	hypothetical protein PSYR_0231	**1.25**
PSYR_3870	SecC motif-containing protein	**1.25**
PSYR_0965	amine oxidase, flavin-containing	**1.24**
PSYR_0252	adenylate cyclase	**1.24**
PSYR_2502	hypothetical protein PSYR_2502	**1.24**
PSYR_3666	hypothetical protein PSYR_3666	**1.23**
PSYR_3586	phosphoesterase PHP, N-terminal:PHP, C-terminal, partial	**1.22**
PSYR_4599	hypothetical protein PSYR_4599	**1.22**
PSYR_4723	hypothetical protein PSYR_4723	**1.22**
PSYR_0181	lipoprotein LppL	**1.21**
PSYR_1803	hypothetical protein PSYR_1803	**1.21**
PSYR_4329	hypothetical protein PSYR_4329	**1.21**
PSYR_1869	hypothetical protein PSYR_1869	**1.21**
PSYR_2641	hypothetical protein PSYR_2641	**1.21**
PSYR_3403	RNA 2\'-O-ribose methyltransferase	**1.2**
PSYR_0212	hypothetical protein PSYR_0212	**1.2**
PSYR_1017	type III effector HopJ1	**1.2**
PSYR_4137	sulfate transporter/antisigma-factor antagonist STAS	**1.2**
PSYR_2234	hypothetical protein PSYR_2234	**1.2**
PSYR_2977	cointegrate resolution protein T	**1.2**
PSYR_0477	hypothetical protein PSYR_0477	**1.19**
PSYR_2879	VirK	**1.19**
PSYR_0857	ribosomal-protein-alanine acetyltransferase	**1.19**
PSYR_3573	hypothetical protein PSYR_3573	**1.19**
PSYR_2233	hypothetical protein PSYR_2233	**1.18**
PSYR_3358	transmembrane pair	**1.18**
PSYR_4947	hypothetical protein PSYR_4947	**1.18**
PSYR_2285	twin-arginine translocation pathway signal	**1.17**
PSYR_1837	zinc-containing alcohol dehydrogenase superfamily protein	**1.17**
PSYR_3664	hypothetical protein PSYR_3664	**1.16**
PSYR_0267	endonuclease/exonuclease/phosphatase	**1.16**
PSYR_2112	hypothetical protein PSYR_2112	**1.15**
PSYR_4736	hypothetical protein PSYR_4736	**1.15**
PSYR_1033	protein YebG	**1.14**
PSYR_0190	nitrogen regulatory protein P-II	**1.14**
PSYR_0565	hypothetical protein PSYR_0565	**1.13**
PSYR_0132	hypothetical protein PSYR_0132	**1.13**
PSYR_3058	amidase	**1.13**
PSYR_1475	Phage integrase:Phage integrase, N-terminal SAM-like	**1.13**
PSYR_0390	hypothetical protein PSYR_0390	**1.12**
PSYR_1064	hypothetical protein PSYR_1064	**1.12**

PSYR_1402	hypothetical protein PSYR_1402	**1.12**
PSYR_2952	NADP oxidoreductase, coenzyme F420-dependent	**1.12**
PSYR_1022	hypothetical protein PSYR_1022	**1.11**
PSYR_0512	N-acetyltransferase GCN5	**1.1**
PSYR_0568	hypothetical protein PSYR_0568	**1.1**
PSYR_2052	lipoprotein	**1.1**
PSYR_3260	hypothetical protein PSYR_3260	**1.1**
PSYR_1389	hypothetical protein PSYR_1389	**1.09**
PSYR_4593	sigma factor domain-containing protein	**1.09**
PSYR_0320	hypothetical protein PSYR_0320	**1.08**
PSYR_4303	hypothetical protein PSYR_4303	**1.07**
PSYR_4594	hypothetical protein PSYR_4594	**1.07**
PSYR_2813	hypothetical protein PSYR_2813	**1.07**
PSYR_0636	LrgA protein	**1.06**
PSYR_0985	hypothetical protein PSYR_0985	**1.06**
PSYR_0226	hypothetical protein PSYR_0226	**1.06**
PSYR_0194	short-chain dehydrogenase	**1.06**
PSYR_4587	baseplate J-like protein	**1.06**
PSYR_1643	hypothetical protein PSYR_1643	**1.05**
PSYR_3352	N-acetyltransferase GCN5	**1.05**
PSYR_0188	hypothetical protein PSYR_0188	**1.05**
PSYR_4172	hypothetical protein PSYR_4172	**1.04**
PSYR_4095	hypothetical protein PSYR_4095	**1.04**
PSYR_1334	Fe-S metabolism associated SufE	**1.03**
PSYR_3962	glycine cleavage T protein (aminomethyl transferase)	**1.03**
PSYR_0555	hypothetical protein PSYR_0555	**1.03**
PSYR_1834	hypothetical protein PSYR_1834	**1.03**
PSYR_0315	hypothetical protein PSYR_0315	**1.03**
PSYR_2477	hypothetical protein PSYR_2477	**1.03**
PSYR_3216	hypothetical protein PSYR_3216	**1.03**
PSYR_4806	hypothetical protein PSYR_4806	**1.02**
PSYR_0856	hypothetical protein PSYR_0856	**1.01**
PSYR_3857	hypothetical protein PSYR_3857	**1.01**
PSYR_1229	preprotein translocase subunit YajC	**1.01**
PSYR_0217	hypothetical protein PSYR_0217	**1.01**
PSYR_4760	hypothetical protein PSYR_4760	**1.01**
PSYR_2134	hypothetical protein PSYR_2134	**1.01**
PSYR_0983	hypothetical protein PSYR_0983	**1.01**
PSYR_3982	3-hydroxyacyl-CoA-ACP transferase	**1.01**
PSYR_4117	ClpXP protease specificity-enhancing factor	**1**
PSYR_1093	permease YjgP/YjgQ	**1**
PSYR_0152	pyridine nucleotide-disulfide oxidoreductase family protein	**-1**
PSYR_0308	hypothetical protein PSYR_0308	**-1**
PSYR_2861	hypothetical protein PSYR_2861	**-1**
PSYR_4915	hypothetical protein PSYR_4915	**-1.01**
PSYR_2088	hypothetical protein PSYR_2088	**-1.01**
PSYR_2307	hypothetical protein PSYR_2307	**-1.01**
PSYR_4655	hypothetical protein PSYR_4655	**-1.02**
PSYR_1911	hypothetical protein PSYR_1911	**-1.03**

PSYR_3268	myo-inositol 2-dehydrogenase	**-1.03**
PSYR_4127	hypothetical protein PSYR_4127	**-1.03**
PSYR_0773	aryldialkylphosphatase	**-1.03**
PSYR_3746	hypothetical protein PSYR_3746	**-1.03**
PSYR_4661	phosphopantetheinyl transferase	**-1.03**
PSYR_4000	hypothetical protein PSYR_4000	**-1.04**
PSYR_0101	hypothetical protein PSYR_0101	**-1.04**
PSYR_0118	hypothetical protein PSYR_0118	**-1.04**
PSYR_1934	hypothetical protein PSYR_1934	**-1.04**
PSYR_3286	hypothetical protein PSYR_3286	**-1.05**
PSYR_1954	hypothetical protein PSYR_1954	**-1.05**
PSYR_1605	hypothetical protein PSYR_1605	**-1.05**
PSYR_2355	GAF domain-containing protein	**-1.05**
PSYR_4068	hypothetical protein PSYR_4068	**-1.06**
PSYR_0648	transporter	**-1.06**
PSYR_1314	lipoprotein	**-1.07**
PSYR_1957	pyoverdine sidechain peptide synthetase I, epsilon-Lys module	**-1.07**
PSYR_2561	hypothetical protein PSYR_2561	**-1.07**
PSYR_2661	hypothetical protein PSYR_2661	**-1.07**
PSYR_2559	hypothetical protein PSYR_2559	**-1.07**
PSYR_1442	hypothetical protein PSYR_1442	**-1.08**
PSYR_3321	helix-turn-helix, Fis-type	**-1.08**
PSYR_3320	hypothetical protein PSYR_3320	**-1.08**
PSYR_2346	hypothetical protein PSYR_2346	**-1.08**
PSYR_0621	hypothetical protein PSYR_0621	**-1.08**
PSYR_0992	hypothetical protein PSYR_0992	**-1.09**
PSYR_0080	hypothetical protein PSYR_0080	**-1.09**
PSYR_0161	hypothetical protein PSYR_0161	**-1.09**
PSYR_2792	Phage head morphosis protein, SPP1 gp7	**-1.09**
PSYR_0341	hypothetical protein PSYR_0341	**-1.09**
PSYR_5114	hypothetical protein PSYR_5114	**-1.1**
PSYR_2797	hypothetical protein PSYR_2797	**-1.1**
PSYR_4309	hypothetical protein PSYR_4309	**-1.1**
PSYR_0530	hypothetical protein PSYR_0530	**-1.11**
PSYR_3494	hypothetical protein PSYR_3494	**-1.11**
PSYR_1935	hypothetical protein PSYR_1935	**-1.11**
PSYR_3759	hypothetical protein PSYR_3759	**-1.12**
PSYR_2996	hypothetical protein PSYR_2996	**-1.12**
PSYR_0619	WavE lipopolysaccharide synthesis	**-1.13**
PSYR_0143	chemotaxis sensory transducer protein	**-1.13**
PSYR_4783	hypothetical protein PSYR_4783	**-1.13**
PSYR_2214	methyl-accepting chemotaxis protein	**-1.13**
PSYR_3341	hypothetical protein PSYR_3341	**-1.13**
PSYR_2905	peptide ABC transporter ATP-binding protein	**-1.15**
PSYR_4084	hypothetical protein PSYR_4084	**-1.15**
PSYR_1741	hypothetical protein PSYR_1741	**-1.15**
PSYR_0906	histidine kinase, HAMP region:Cache: chemotaxis sensory transducer	**-1.16**
PSYR_3046	hypothetical protein PSYR_3046	**-1.16**
PSYR_1527	hypothetical protein PSYR_1527	**-1.16**

PSYR_2299	hypothetical protein PSYR_2299	**-1.16**
PSYR_2663	hypothetical protein PSYR_2663	**-1.16**
PSYR_1088	hypothetical protein PSYR_1088	**-1.17**
PSYR_2961	ABC transporter	**-1.18**
PSYR_4026	hypothetical protein PSYR_4026	**-1.18**
PSYR_1554	hypothetical protein PSYR_1554	**-1.18**
PSYR_0940	hypothetical protein	**-1.18**
PSYR_2660	hypothetical protein PSYR_2660	**-1.19**
PSYR_0426	lipoprotein	**-1.2**
PSYR_1701	membrane protein	**-1.2**
PSYR_1530	hypothetical protein PSYR_1530	**-1.2**
PSYR_0923	hypothetical protein PSYR_0923	**-1.21**
PSYR_0112	hypothetical protein PSYR_0112	**-1.22**
PSYR_1927	hypothetical protein PSYR_1927	**-1.22**
PSYR_3730	hypothetical protein PSYR_3730	**-1.22**
PSYR_0739	hypothetical protein	**-1.23**
PSYR_2317	hypothetical protein	**-1.23**
PSYR_3092	PAP2 superfamily protein	**-1.24**
PSYR_1579	hypothetical protein PSYR_1579	**-1.24**
PSYR_1717	TPR repeat-containing von Willebrand factor, type A	**-1.24**
PSYR_4486	hypothetical protein PSYR_4486	**-1.24**
PSYR_1446	hypothetical protein PSYR_1446	**-1.26**
PSYR_0578	histidine kinase, HAMP region: chemotaxis sensory transducer	**-1.26**
PSYR_1028	hypothetical protein PSYR_1028	**-1.27**
PSYR_2308	hypothetical protein PSYR_2308	**-1.27**
PSYR_1952	hypothetical protein PSYR_1952	**-1.27**
PSYR_1524	hypothetical protein PSYR_1524	**-1.27**
PSYR_1931	hypothetical protein PSYR_1931	**-1.27**
PSYR_0413	hypothetical protein PSYR_0413	**-1.28**
PSYR_1150	chemotaxis sensory transducer protein	**-1.28**
PSYR_0070	hypothetical protein PSYR_0070	**-1.28**
PSYR_1953	hypothetical protein PSYR_1953	**-1.3**
PSYR_0789	chemotaxis sensory transducer protein	**-1.31**
PSYR_2699	hypothetical protein PSYR_2699	**-1.31**
PSYR_0531	LmbE-like protein	**-1.31**
PSYR_4437	hypothetical protein PSYR_4437	**-1.31**
PSYR_2738	regulatory protein LysR	**-1.32**
PSYR_2724	hypothetical protein PSYR_2724	**-1.32**
PSYR_3728	hypothetical protein PSYR_3728	**-1.32**
PSYR_3729	hypothetical protein PSYR_3729	**-1.32**
PSYR_0939	hypothetical protein	**-1.32**
PSYR_3761	hypothetical protein PSYR_3761	**-1.33**
PSYR_4926	N-acetyltransferase GCN5	**-1.33**
PSYR_5093	histidine kinase, HAMP region: chemotaxis sensory transducer	**-1.33**
PSYR_4907	hypothetical protein PSYR_4907	**-1.33**
PSYR_3344	hypothetical protein PSYR_3344	**-1.34**
PSYR_1716	hypothetical protein PSYR_1716	**-1.34**
PSYR_1255	hypothetical protein PSYR_1255	**-1.34**
PSYR_3940	hypothetical protein PSYR_3940	**-1.35**

PSYR_3731	hypothetical protein PSYR_3731	**-1.35**
PSYR_2560	hypothetical protein PSYR_2560	**-1.35**
PSYR_4719	hypothetical protein PSYR_4719	**-1.37**
PSYR_1924	hypothetical protein PSYR_1924	**-1.37**
PSYR_4989	hypothetical protein PSYR_4989	**-1.37**
PSYR_0887	sensory box protein	**-1.38**
PSYR_4925	hypothetical protein PSYR_4925	**-1.38**
PSYR_2841	hypothetical protein PSYR_2841	**-1.38**
PSYR_2673	hypothetical protein PSYR_2673	**-1.38**
PSYR_3815	hypothetical protein PSYR_3815	**-1.38**
PSYR_0153	hypothetical protein PSYR_0153	**-1.39**
PSYR_2826	hypothetical protein PSYR_2826	**-1.39**
PSYR_3067	hypothetical protein PSYR_3067	**-1.4**
PSYR_2747	extracellular ligand-binding receptor	**-1.4**
PSYR_1881	hypothetical protein PSYR_1881	**-1.4**
PSYR_4085	hypothetical protein PSYR_4085	**-1.4**
PSYR_3941	hypothetical protein PSYR_3941	**-1.41**
PSYR_0380	histidine kinase, HAMP region: chemotaxis sensory transducer	**-1.41**
PSYR_5115	helicase	**-1.41**
PSYR_2316	ral secretion pathway protein I	**-1.41**
PSYR_1523	hypothetical protein PSYR_1523	**-1.42**
PSYR_2739	ThiJ/PfpI	**-1.43**
PSYR_3371	oxidoreductase, molybdopterin binding	**-1.43**
PSYR_1926	hypothetical protein PSYR_1926	**-1.43**
PSYR_0907	hypothetical protein PSYR_0907	**-1.45**
PSYR_3788	hypothetical protein PSYR_3788	**-1.45**
PSYR_2799	hypothetical protein PSYR_2799	**-1.45**
PSYR_2674	hypothetical protein PSYR_2674	**-1.45**
PSYR_2609	Alpha/beta hydrolase fold	**-1.45**
PSYR_4888	hypothetical protein PSYR_4888	**-1.46**
PSYR_2297	Short-chain dehydrogenase/reductase SDR	**-1.46**
PSYR_1529	hypothetical protein PSYR_1529	**-1.47**
PSYR_2987	Cof protein/HAD-superfamily hydrolase	**-1.48**
PSYR_3816	hypothetical protein PSYR_3816	**-1.48**
PSYR_0787	sulfate transporter/antisigma-factor antagonist STAS	**-1.49**
PSYR_2800	hypothetical protein PSYR_2800	**-1.49**
PSYR_3133	amidohydrolase 2	**-1.52**
PSYR_0871	hypothetical protein PSYR_0871	**-1.53**
PSYR_0825	hypothetical protein PSYR_0825	**-1.53**
PSYR_0427	hypothetical protein PSYR_0427	**-1.54**
PSYR_2744	hypothetical protein PSYR_2744	**-1.54**
PSYR_4025	ATPase	**-1.55**
PSYR_2185	hypothetical protein PSYR_2185	**-1.55**
PSYR_2543	hypothetical protein PSYR_2543	**-1.56**
PSYR_0958	amine oxidase, flavin-containing	**-1.57**
PSYR_1528	Arc-like DNA binding	**-1.58**
PSYR_3534	histidine kinase, HAMP region:Cache: chemotaxis sensory transducer	**-1.58**
PSYR_0104	hypothetical protein PSYR_0104	**-1.58**
PSYR_0772	hypothetical protein PSYR_0772	**-1.58**

PSYR_1460	hypothetical protein PSYR_1460	**-1.58**
PSYR_4258	hypothetical protein PSYR_4258	**-1.61**
PSYR_1030	bacteriophage N4 adsorption protein B	**-1.62**
PSYR_4878	outer membrane porin	**-1.62**
PSYR_0840	pentapeptide repeat-containing protein	**-1.62**
PSYR_1928	regulatory protein LysR	**-1.62**
PSYR_0893	hypothetical protein PSYR_0893	**-1.62**
PSYR_4035	insecticidal toxin protein	**-1.63**
PSYR_3900	hypothetical protein PSYR_3900	**-1.63**
PSYR_1470	hypothetical protein PSYR_1470	**-1.63**
PSYR_0532	hypothetical protein PSYR_0532	**-1.64**
PSYR_0913	histidine kinase, HAMP region: chemotaxis sensory transducer	**-1.65**
PSYR_3311	virulence factor MVIN-like	**-1.65**
PSYR_4955	hypothetical protein PSYR_4955	**-1.66**
PSYR_4514	hypothetical protein PSYR_4514	**-1.67**
PSYR_1490	hypothetical protein PSYR_1490	**-1.69**
PSYR_0330	hypothetical protein PSYR_0330	**-1.69**
PSYR_1519	SecC motif-containing protein	**-1.7**
PSYR_1471	hypothetical protein PSYR_1471	**-1.7**
PSYR_1713	hypothetical protein PSYR_1713	**-1.72**
PSYR_4207	N-acetyltransferase GCN5	**-1.72**
PSYR_2840	hypothetical protein PSYR_2840	**-1.73**
PSYR_2999	LmbE-like protein	**-1.77**
PSYR_2512	hypothetical protein PSYR_2512	**-1.77**
PSYR_1263	ATPase	**-1.77**
PSYR_0957	hypothetical protein PSYR_0957	**-1.78**
PSYR_3342	hypothetical protein PSYR_3342	**-1.78**
PSYR_4949	hypothetical protein PSYR_4949	**-1.81**
PSYR_0894	PilT protein, N-terminal, partial	**-1.82**
PSYR_1699	hypothetical protein PSYR_1699	**-1.82**
PSYR_4676	hypothetical protein PSYR_4676	**-1.84**
PSYR_1539	histidine kinase, HAMP region: chemotaxis sensory transducer	**-1.84**
PSYR_2998	hypothetical protein PSYR_2998	**-1.85**
PSYR_3000	methyltransferase	**-1.86**
PSYR_4682	hypothetical protein PSYR_4682	**-1.86**
PSYR_2791	hypothetical protein PSYR_2791	**-1.88**
PSYR_2633	hypothetical protein PSYR_2633	**-1.89**
PSYR_3090	hypothetical protein PSYR_3090	**-1.89**
PSYR_3373	hypothetical protein PSYR_3373	**-1.93**
PSYR_3998	heat shock protein DnaJ, N-terminal, partial	**-1.93**
PSYR_1089	hypothetical protein PSYR_1089	**-1.94**
PSYR_3533	hypothetical protein PSYR_3533	**-1.95**
PSYR_0100	hypothetical protein PSYR_0100	**-1.95**
PSYR_1029	peptidase aspartic, active site	**-1.96**
PSYR_2279	hypothetical protein PSYR_2279	**-1.96**
PSYR_5012	hypothetical protein PSYR_5012	**-1.96**
PSYR_1930	hypothetical protein PSYR_1930	**-1.97**
PSYR_0167	hypothetical protein PSYR_0167	**-1.98**
PSYR_0493	CheW-like protein	**-1.99**

PSYR_1085	SAM-dependent methyltransferase	**-2.01**
PSYR_4974	Rhs element Vgr protein	**-2.01**
PSYR_4973	hypothetical protein PSYR_4973	**-2.01**
PSYR_1204	hypothetical protein PSYR_1204	**-2.08**
PSYR_4385	Alpha-2-macroglobulin, N-terminal:Alpha-2-macroglobulin, N-terminal, partial	**-2.09**
PSYR_0381	16S ribosomal RNA methyltransferase RsmE	**-2.1**
PSYR_2461	hypothetical protein PSYR_2461	**-2.11**
PSYR_4985	hypothetical protein PSYR_4985	**-2.12**
PSYR_2650	hypothetical protein PSYR_2650	**-2.13**
PSYR_3310	hexapaptide repeat-containing transferase	**-2.14**
PSYR_4956	hypothetical protein PSYR_4956	**-2.14**
PSYR_2646	radical SAM family protein	**-2.16**
PSYR_1472	hypothetical protein PSYR_1472	**-2.16**
PSYR_4984	hypothetical protein PSYR_4984	**-2.16**
PSYR_4082	hypothetical protein PSYR_4082	**-2.18**
PSYR_2963	amino acid ABC transporter permease	**-2.21**
PSYR_2568	short chain dehydrogenase	**-2.23**
PSYR_4509	hypothetical protein PSYR_4509	**-2.23**
PSYR_4355	hypothetical protein PSYR_4355	**-2.26**
PSYR_1477	hypothetical protein PSYR_1477	**-2.27**
PSYR_2824	hypothetical protein PSYR_2824	**-2.31**
PSYR_1515	hypothetical protein PSYR_1515	**-2.33**
PSYR_2651	hypothetical protein PSYR_2651	**-2.33**
PSYR_2433	hypothetical protein PSYR_2433	**-2.4**
PSYR_2842	hypothetical protein PSYR_2842	**-2.4**
PSYR_0332	hypothetical protein PSYR_0332	**-2.4**
PSYR_2652	hypothetical protein PSYR_2652	**-2.42**
PSYR_1476	hypothetical protein PSYR_1476	**-2.46**
PSYR_2837	hypothetical protein PSYR_2837	**-2.48**
PSYR_4083	hypothetical protein PSYR_4083	**-2.5**
PSYR_2345	ea59 protein	**-2.51**
PSYR_0750	hypothetical protein PSYR_0750	**-2.51**
PSYR_4872	hypothetical protein PSYR_4872	**-2.53**
PSYR_0085	hypothetical protein PSYR_0085	**-2.54**
PSYR_0099	hypothetical protein PSYR_0099	**-2.57**
PSYR_3601	hypothetical protein PSYR_3601	**-2.6**
PSYR_5010	hypothetical protein PSYR_5010	**-2.61**
PSYR_0335	hypothetical protein PSYR_0335	**-2.62**
PSYR_5009	hypothetical protein PSYR_5009	**-2.62**
PSYR_4968	hypothetical protein PSYR_4968	**-2.63**
PSYR_4983	Rhs element Vgr protein	**-2.63**
PSYR_1929	hypothetical protein PSYR_1929	**-2.7**
PSYR_0326	hypothetical protein PSYR_0326	**-2.73**
PSYR_2836	hypothetical protein PSYR_2836	**-2.73**
PSYR_0097	hypothetical protein PSYR_0097	**-2.75**
PSYR_4957	hypothetical protein PSYR_4957	**-2.75**
PSYR_2623	hypothetical protein PSYR_2623	**-2.77**
PSYR_3719	hypothetical protein PSYR_3719	**-2.79**
PSYR_1474	hypothetical protein PSYR_1474	**-2.87**

PSYR_2644	hypothetical protein PSYR_2644	**-2.88**
PSYR_3718	hypothetical protein PSYR_3718	**-2.88**
PSYR_2215	CheW-like protein	**-2.94**
PSYR_4967	hypothetical protein PSYR_4967	**-2.96**
PSYR_4972	Sel1 repeat-containing protein	**-3**
PSYR_1203	hypothetical protein PSYR_1203	**-3.06**
PSYR_2648	hypothetical protein PSYR_2648	**-3.06**
PSYR_2647	hypothetical protein PSYR_2647	**-3.11**
PSYR_2612	syrP protein	**-3.19**
PSYR_4963	hypothetical protein PSYR_4963	**-3.23**
PSYR_2649	hypothetical protein PSYR_2649	**-3.28**
PSYR_4959	hypothetical protein PSYR_4959	**-3.38**
PSYR_4982	hypothetical protein PSYR_4982	**-3.39**
PSYR_4388	response regulator receiver	**-3.41**
PSYR_2625	lipoprotein	**-3.51**
PSYR_4813	sorbitol dehydrogenase	**-3.54**
PSYR_2632	virulence protein SrfB	**-3.58**
PSYR_3807	hypothetical protein PSYR_3807	**-3.58**
PSYR_0928	hypothetical protein PSYR_0928	**-3.71**
PSYR_4966	ImpA-like protein	**-3.85**
PSYR_4510	hypothetical protein PSYR_4510	**-3.85**
PSYR_2624	lipoprotein	**-3.87**
PSYR_3809	hypothetical protein PSYR_3809	**-3.87**
PSYR_0737	transmembrane protein	**-3.87**
PSYR_4633	SpoVR family protein	**-3.89**
PSYR_2323	hypothetical protein PSYR_2323	**-4.05**
PSYR_4962	hypothetical protein PSYR_4962	**-4.07**
PSYR_2626	hypothetical protein PSYR_2626	**-4.1**
PSYR_3129	3-hydroxyacyl-CoA-ACP transferase	**-4.12**
PSYR_2631	hypothetical protein PSYR_2631	**-4.18**
PSYR_4971	Sel1 repeat-containing protein	**-4.24**
PSYR_0098	hypothetical protein PSYR_0098	**-4.31**
PSYR_0927	hypothetical protein PSYR_0927	**-4.32**
PSYR_4511	hypothetical protein PSYR_4511	**-4.38**
PSYR_4961	hypothetical protein PSYR_4961	**-4.38**
PSYR_4632	hypothetical protein PSYR_4632	**-4.41**
PSYR_4965	hypothetical protein PSYR_4965	**-4.42**
PSYR_2630	hypothetical protein PSYR_2630	**-4.73**
PSYR_2629	von Willebrand factor, type A	**-5.03**
PSYR_3808	hypothetical protein PSYR_3808	**-5.06**
PSYR_4987	hypothetical protein PSYR_4987	**-5.37**
