# Supplementary material for: Comparative transcriptomic analysis of global gene expression mediated by (p) ppGpp reveals common regulatory networks in Pseudomonas syringae
Source: BMC Genomics. 2020 Apr 10;21:296. doi: 10.1186/s12864-020-6701-2 (PMC7146990; doi:10.1186/s12864-020-6701-2)
Supplement: Supplementary file 3 — Additional file 3: Table S3. List of homologues and unique genes via comparative analysis of PstDC3000 and PssB728a genomes. Table S4. List of homologues genes in both PstDC3000 and PssB728a regulated by (p) ppGpp in a similar way. Table S5. List of homologues genes in both PstDC3000 and PssB728a inversely regulated by (p)ppGpp. [file 12864_2020_6701_MOESM3_ESM.zip › Additional file 3 Table S4.pdf]

Table S4. List of homologues genes in both *Pst*DC3000 and *Pss*B728a regulated by (p)ppGpp in a similar way with p-value <0.05

Locus tag		Gene description	(p)ppGpp^0$_{Pst\mathrm{DC3000}}$ /*Pst*DC3000	(p)ppGpp^0$_{Pss\mathrm{B728a}}$ /*Pss*B728a
**DC3000**	**B728a**			
**Type III secretion system**				
PSPTO_1373	*PSYR_1184*	*hrpW1*, type III helper protein HrpW1	**-6.12**	**-4.31**
PSPTO_1382	*PSYR_1193*	*hrpZ1*, type III restriction system endonuclease	**-5.22**	**-5.36**
PSPTO_1383	*PSYR_1194*	*hrpB*, type III secretion protein HrpB	**-5.17**	**-5.17**
PSPTO_1384	*PSYR_1195*	*hrcJ*, type III secretion protein HrcJ	**-5.11**	**-4.24**
PSPTO_0588	*PSYR_1889*	*hopH1*, type III effector HopH1	**-4.94**	**-4.24**
PSPTO_1385	*PSYR_1196*	*hrpD*, type III secretion protein HrpD	**-4.76**	**-4.24**
PSPTO_1399	*PSYR_1212*	*hrpO*, type III secretion protein HrpO	**-4.72**	**-5.20**
PSPTO_1375	*PSYR_1186*	*hopM1*, type III effector HopM1	**-4.64**	**-1.75**
PSPTO_4101	*PSYR_3839*	*hopAK1*, type III helper protein HopAK1	**-4.59**	**-2.26**
PSPTO_1400	*PSYR_1213*	*hrcN*, type III secretion cytoplasmic ATPase HrcN	**-4.51**	**-3.94**
PSPTO_1374	*PSYR_1185*	*shcM,* type III chaperone ShcM	**-4.49**	**-3.35**
PSPTO_1390	*PSYR_1201*	*hrpT*, type III secretion protein	**-4.47**	**-4.07**
PSPTO_1386	*PSYR_1197*	*hrpE*, type III secretion protein HrpE	**-4.43**	**-3.46**
PSPTO_1405	*PSYR_1218*	*hrpK1*, type III helper protein HrpK1	**-4.36**	**-4.65**
PSPTO_4776	*PSYR_4326*	*hopI1*, type III effector HopI1	**-4.36**	**-2.05**
PSPTO_1389	*PSYR_1200*	*hrcC,* outer-membrane type III secretion protein HrcC	**-4.22**	**-2.95**
PSPTO_1398	*PSYR_1211*	*hrpP*, type III secretion protein HrpP	**-4.17**	**-6.34**
PSPTO_1401	*PSYR_1214*	*hrpQ*, type III secretion protein HrpQ	**-4.15**	**-3.59**
PSPTO_3087	*PSYR_4659*	*hopAB2*, type III effector HopAB2	**-4.14**	**-1.28**
PSPTO_1377	*PSYR_1188*	*avrE1*, type III effector protein AvrE1	**-4.13**	**-2.77**
PSPTO_1378	*PSYR_1189*	*hrpH*, membrane-bound lytic murein transglycosylase D	**-4.11**	**-2.50**
PSPTO_1395	*PSYR_1208*	*hrcR*, type III secretion protein HrcR	**-4.05**	**-4.69**
PSPTO_1391	*PSYR_1202*	*hrpV*, negative regulator of hrp expression HrpV	**-4.04**	**-3.91**
PSPTO_1394	*PSYR_1207*	*hrcS*, type III secretion protein HrcS	**-3.98**	**-4.32**
PSPTO_1396	*PSYR_1209*	*hrcQb*, type III secretion protein HrcQb	**-3.82**	**-5.76**
PSPTO_1392	*PSYR_1205*	*hrcU*, type III secretion protein HrcU	**-3.73**	**-2.74**
PSPTO_1393	*PSYR_1206*	*hrcT*, type III secretion protein HrcT	**-3.58**	**-3.58**
PSPTO_1388	*PSYR_1199*	*hrpG*, type III secretion protein HrpG	**-3.55**	**-3.63**
PSPTO_1397	*PSYR_1210*	*hrcQa*, type III secretion protein HrcQa	**-3.54**	**-4.91**
PSPTO_1403	*PSYR_1216*	*hrpJ*, type III secretion protein HrpJ	**-3.46**	**-3.46**
PSPTO_1402	*PSYR_1215*	*hrcV*, type III secretion protein HrcV	**-3.45**	**-3.37**
PSPTO_1372	*PSYR_1183*	*hopAA1-1*, type III effector HopAA1-1	**-3.37**	**-5.28**
PSPTO_1376	*PSYR_1187*	*shcE*, type III chaperone ShcE	**-3.20**	**-0.95**

PSPTO_1404	*PSYR_1217*	*hrpL*, RNA polymerase sigma factor HrpL	**-2.82**	**-3.78**
PSPTO_0901	*PSYR_0778*	*hopAG1*, type III effector HopAG1	**-2.53**	**-1.99**
PSPTO_4001	*PSYR_4919*	*avrPto1*, type III effector protein AvrPto1	**-2.36**	**-6.01**
PSPTO_1568	*PSYR_3813*	*hopAF1*, type III effector HopAF1	**-2.25**	**-1.91**
PSPTO_0905	*PSYR_0779*	*hopAH1*, type III effector HopAH1	**-1.50**	**-2.54**
**Translation, ribosomal structure and biogenesis**				
PSPTO_3352	*PSYR_3182*	*infA*, translation initiation factor IF-1	**4.20**	**2.33**
PSPTO_5136	*PSYR_0399*	*rpmE-2*, 50S ribosomal protein L31	**3.27**	**2.17**
PSPTO_0090	*PSYR_0225*	*rpmG*, 50S ribosomal protein L33	**2.71**	**2.14**
PSPTO_3750	*PSYR_1728*	hypothetical protein PSPTO_3750	**2.63**	**2.26**
PSPTO_5615	*PSYR_5137*	*rpmH*, 50S ribosomal protein L34	**2.56**	**1.08**
PSPTO_0802	*PSYR_0707*	*rpsT*, 30S ribosomal protein S20	**2.47**	**1.38**
PSPTO_0089	*PSYR_0224*	*rpmB*, 50S ribosomal protein L28	**2.44**	**2.00**
PSPTO_5138	*PSYR_0397*	*argS*, arginyl-tRNA synthetase	**2.43**	**1.53**
PSPTO_1420	*PSYR_1234*	RNA methyltransferase, TrmH family, group 1	**2.37**	**1.30**
PSPTO_4425	*PSYR_4119*	*rpsI*, 30S ribosomal protein S9	**2.31**	**1.45**
PSPTO_1146	*PSYR_0986*	*rsmC*, ribosomal RNA small subunit methyltransferase C	**2.30**	**1.66**
PSPTO_4019	*PSYR_1392*	ribosomal protein S12 methylthiotransferase	**2.25**	**0.86**
PSPTO_0072	*PSYR_0208*	endoribonuclease L-PSP	**2.19**	**3.22**
PSPTO_4645	*PSYR_4279*	methyltransferase	**2.19**	**1.52**
PSPTO_3835	*PSYR_1644*	*rpmF*, 50S ribosomal protein L32	**2.14**	**1.16**
PSPTO_4862	*PSYR_4402*	*prmA*, ribosomal protein L11 methyltransferase	**2.14**	**0.51**
PSPTO_4426	*PSYR_4120*	*rplM*, 50S ribosomal protein L13	**2.14**	**1.30**
PSPTO_0184	*PSYR_0012*	*glyQ*, glycyl-tRNA synthetase subunit alpha	**2.14**	**1.52**
PSPTO_0177	*PSYR_0019*	*def-1*, polypeptide deformylase	**2.12**	**2.03**
PSPTO_1476	*PSYR_1285*	*rplS*, 50S ribosomal protein L19	**1.97**	**1.30**
PSPTO_4930	*PSYR_0584*	*rplI*, 50S ribosomal protein L9	**1.94**	**1.02**
PSPTO_1744	*PSYR_3648*	initiation factor 2 subunit family	**1.94**	**1.36**
PSPTO_1501	*PSYR_1311*	*lysS*, lysyl-tRNA synthetase	**1.93**	**0.69**
PSPTO_0963	*PSYR_0830*	*pcnB*, poly(A) polymerase	**1.92**	**1.29**
PSPTO_0827	*PSYR_0726*	*rluD*, ribosomal large subunit pseudouridine synthase D	**1.91**	**1.70**
PSPTO_0539	*PSYR_4639*	*rpsU*, 30S ribosomal protein S21	**1.87**	**1.04**
PSPTO_1818	*PSYR_3579*	hypothetical protein PSPTO_1818	**1.87**	**1.26**
PSPTO_0618	*PSYR_4556*	*rplL*, 50S ribosomal protein L7/L12	**1.84**	**0.59**
PSPTO_1412	*PSYR_1227*	*queA*, S-adenosylmethionine--tRNA ribosyltransferase-isomerase	**1.83**	**1.59**
PSPTO_1561	*PSYR_1370*	hypothetical protein PSPTO_1561	**1.82**	**1.13**
PSPTO_3922	*PSYR_1563*	*rnd*, ribonuclease D	**1.79**	**1.37**

PSPTO_4021	*PSYR_1390*	S4 domain-containing protein	**1.78**	**1.90**
PSPTO_1473	*PSYR_1282*	*rpsP*, 30S ribosomal protein S16	**1.76**	**0.72**
PSPTO_0077	*PSYR_0213*	*rph*, ribonuclease PH	**1.76**	**2.02**
PSPTO_1413	*PSYR_1228*	*tgt*, queuine tRNA-ribosyltransferase	**1.73**	**1.45**
PSPTO_0625	*PSYR_4549*	*rpsJ*, 30S ribosomal protein S10	**1.73**	**1.21**
PSPTO_3358	*PSYR_3190*	*trmU*, tRNA (5-methyl aminomethyl-2-thiouridylate)-methyltransferase	**1.72**	**0.93**
PSPTO_1103	*PSYR_0943*	ribosomal 5S rRNA E-loop binding protein Ctc/L25/TL5	**1.70**	**1.13**
PSPTO_1534	*PSYR_1343*	*rpsB*, 30S ribosomal protein S2	**1.68**	**2.07**
PSPTO_0616	*PSYR_4558*	*rplA*, 50S ribosomal protein L1	**1.66**	**1.40**
PSPTO_3817	*PSYR_1662*	*truA*, tRNA pseudouridine synthase A	**1.65**	**0.87**
PSPTO_0640	*PSYR_4534*	*rpsH*, 30S ribosomal protein S8	**1.62**	**0.71**
PSPTO_4932	*PSYR_0582*	*rpsR*, 30S ribosomal protein S18	**1.61**	**0.95**
PSPTO_0185	*PSYR_0011*	*glyS*, glycyl-tRNA synthetase subunit beta	**1.59**	**1.07**
PSPTO_4812	*PSYR_4352*	*leuS*, leucyl-tRNA synthetase	**1.59**	**0.50**
PSPTO_5323	*PSYR_4881*	TrmH family RNA methyltransferase	**1.57**	**1.12**
PSPTO_3840	*PSYR_1639*	*rluC*, ribosomal large subunit pseudouridine synthase C	**1.51**	**2.46**
PSPTO_1475	*PSYR_1284*	*trmD*, tRNA (guanine-N1)-methyltransferase	**1.49**	**0.78**
PSPTO_0617	*PSYR_4557*	*rplJ*, 50S ribosomal protein L10	**1.49**	**0.53**
PSPTO_0615	*PSYR_4559*	*rplK*, 50S ribosomal protein L11	**1.49**	**1.25**
PSPTO_1474	*PSYR_1283*	*rimM*, 16S rRNA-processing protein RimM	**1.47**	**0.68**
PSPTO_1268	*PSYR_1086*	*valS*, valyl-tRNA synthetase	**1.43**	**0.36**
PSPTO_1533	*PSYR_1342*	*map-1*, methionine aminopeptidase	**1.43**	**0.84**
PSPTO_0797	*PSYR_0701*	*rplU*, 50S ribosomal protein L21	**1.40**	**1.66**
PSPTO_2378	*PSYR_2162*	*thrS*, threonyl-tRNA synthetase	**1.38**	**1.37**
PSPTO_1500	*PSYR_1310*	*prfB*, hypothetical protein PSPTO_1500	**1.37**	**0.83**
PSPTO_0641	*PSYR_4533*	*rplF*, 50S ribosomal protein L6	**1.36**	**0.48**
PSPTO_5614	*PSYR_5136*	*rnpA*, ribonuclease P protein component	**1.36**	**0.48**
PSPTO_0806	*PSYR_0710*	*ileS*, isoleucyl-tRNA synthetase	**1.36**	**0.57**
PSPTO_1535	*PSYR_1344*	*tsf*, translation elongation factor Ts	**1.35**	**1.62**
PSPTO_4499	*PSYR_4189*	hypothetical protein PSPTO_4499	**1.34**	**0.61**
PSPTO_4804	*PSYR_4345*	*miaB*, tRNA-i(6)A37 modification enzyme MiaB	**1.34**	**1.15**
PSPTO_4933	*PSYR_0581*	*rpsF*, 30S ribosomal protein S6	**1.33**	**0.97**
PSPTO_0544	*PSYR_4634*	*cca,* tRNA nucleotidyltransferase	**1.32**	**1.10**
PSPTO_0643	*PSYR_4531*	*rpsE,* 30S ribosomal protein S5	**1.32**	**0.19**
PSPTO_4158	*PSYR_3895*	*rnt*, ribonuclease T	**1.31**	**1.03**
PSPTO_1811	*PSYR_3585*	Sua5/YciO/YrdC/YwlC family protein	**1.29**	**1.18**
PSPTO_0626	*PSYR_4548*	*rplC*, 50S ribosomal protein L3	**1.28**	**1.12**
PSPTO_0652	*PSYR_4523*	*rplQ*, 50S ribosomal protein L17	**1.24**	**0.32**

PSPTO_3981	*PSYR_1406*	*aspS*, aspartyl-tRNA synthetase	**1.20**	**1.26**
PSPTO_2311	*PSYR_2108*	23S rRNA m(2)G2445 methyltransferase	**1.19**	**1.63**
PSPTO_0642	*PSYR_4532*	*rplR*, 50S ribosomal protein L18	**1.19**	**0.40**
PSPTO_0650	*PSYR_4525*	*rpsD*, 30S ribosomal protein S4	**1.18**	**0.18**
PSPTO_4654	*PSYR_4287*	*trmA*, tRNA (uracil-5-)-methyltransferase	**1.17**	**0.61**
PSPTO_1815	*PSYR_3581*	*rluB*, ribosomal large subunit pseudouridine synthase B	**1.15**	**0.88**
PSPTO_4247	*PSYR_3981*	*rsuA*, ribosomal small subunit pseudouridine synthase A	**1.12**	**0.39**
PSPTO_1765	*PSYR_3628*	*efp*, translation elongation factor P	**1.12**	**0.79**
PSPTO_0629	*PSYR_4545*	*rplB*, 50S ribosomal protein L2	**1.11**	**0.67**
PSPTO_1753	*PSYR_3639*	hypothetical protein PSPTO_1753	**1.11**	**1.18**
PSPTO_0102	*PSYR_0236*	endoribonuclease L-PSP family protein	**1.10**	**0.86**
PSPTO_3988	*PSYR_1399*	*proS*, prolyl-tRNA synthetase	**1.10**	**0.56**
PSPTO_2166	*PSYR_1977*	*gltX*, glutamyl-tRNA synthetase	**1.09**	**1.53**
PSPTO_0644	*PSYR_4530*	*rpmD*, 50S ribosomal protein L30	**1.09**	**0.13**
PSPTO_0627	*PSYR_4547*	*rplD*, 50S ribosomal protein L4	**1.08**	**0.93**
PSPTO_1750	*PSYR_3642*	*rpsA*, 30S ribosomal protein S1	**1.06**	**0.78**
PSPTO_4841	*PSYR_4380*	translation initiation factor Sui1	**1.05**	**0.64**
PSPTO_0967	*PSYR_0834*	glutamyl-Q tRNA(Asp) synthetase	**1.04**	**0.64**
PSPTO_0178	*PSYR_0018*	*fmt*, methionyl-tRNA formyltransferase	**1.00**	**2.24**
PSPTO_2349	*PSYR_2133*	RNA pseudouridine synthase family protein	**0.90**	**1.17**
PSPTO_2381	*PSYR_2165*	50S ribosomal protein L20	**0.72**	**1.66**
PSPTO_2379	*PSYR_2163*	translation initiation factor IF-3	**0.54**	**1.66**
PSPTO_0179	*PSYR_0017*	sun protein	**0.53**	**1.28**
PSPTO_2380	*PSYR_2164*	50S ribosomal protein L35	**0.24**	**1.63**
PSPTO_5507	*PSYR_5059*	GNAT family acetyltransferase	**-0.34**	**-1.43**
PSPTO_3006	*PSYR_2887*	deaminase AmnE	**-0.94**	**-1.36**
PSPTO_2964	*PSYR_2749*	hypothetical protein PSPTO_2964	**-1.03**	**-0.31**
PSPTO_4490	*PSYR_4180*	*infB*, translation initiation factor IF-2	**-1.09**	**-0.92**
PSPTO_0551	*PSYR_4627*	*ksgA*, dimethyladenosine transferase	**-1.31**	**-3.50**
PSPTO_4489	*PSYR_4179*	*rbfA*, ribosome-binding factor A	**-1.72**	**-1.70**
PSPTO_2421	*PSYR_2208*	*amdA*, amidase	**-2.06**	**-0.68**
PSPTO_2310	*PSYR_2107*	*rmf*, ribosome modulation factor-related protein	**-2.74**	**-4.00**
**RNA processing and modification**				
PSPTO_4950	*PSYR_0564*	*orn*, oligoribonuclease	**1.30**	**1.83**
**Transcription**				
PSPTO_1768	*PSYR_3626*	MarR family transcriptional regulator	**3.27**	**0.91**
PSPTO_0327	*PSYR_0257*	S1 RNA binding domain-containing protein	**2.79**	**1.71**
PSPTO_4180	*PSYR_3917*	*metR*, transcriptional activator MetR	**2.51**	**1.17**
PSPTO_0689	*PSYR_4463*	*nrdR*, hypothetical protein PSPTO_0689	**2.44**	**1.17**

PSPTO_3510	*PSYR_3283*	*lexA-2*, LexA repressor	**2.34**	**2.20**
PSPTO_5267	*PSYR_0276*	zinc uptake regulation protein	**2.32**	**1.04**
PSPTO_0537	*PSYR_4641*	*rpoD*, RNA polymerase sigma-70 factor	**2.17**	**1.29**
PSPTO_1216	*PSYR_1044*	LysR family transcriptional regulator	**2.14**	**2.50**
PSPTO_5638	*PSYR_2141*	hypothetical protein PSPTO_5638	**1.80**	**1.96**
PSPTO_3822	*PSYR_1657*	TetR family transcriptional regulator	**1.72**	**0.61**
PSPTO_2808	*PSYR_2536*	LysR family transcriptional regulator	**1.71**	**0.56**
PSPTO_1336	*PSYR_1152*	ArsR family transcriptional regulator	**1.70**	**1.08**
PSPTO_0074	*PSYR_0210*	*rpoZ*, DNA-directed RNA polymerase subunit omega	**1.69**	**1.25**
PSPTO_1733	*PSYR_3659*	*bolA*, bolA protein	**1.64**	**1.39**
PSPTO_1158	*PSYR_1000*	TetR family transcriptional regulator	**1.62**	**0.79**
PSPTO_0749	*PSYR_0653*	heavy metal-dependent transcriptional regulator	**1.60**	**2.41**
PSPTO_2404	*PSYR_2189*	ArsR family transcriptional regulator	**1.49**	**1.67**
PSPTO_4132	*PSYR_3869*	repressor protein c2	**1.46**	**0.93**
PSPTO_4104	*PSYR_3842*	ATP-dependent helicase HepA	**1.45**	**0.64**
PSPTO_0362	*PSYR_4814*	DeoR family transcriptional regulator	**1.44**	**0.71**
PSPTO_0430	*PSYR_4748*	*rpoH*, RNA polymerase sigma-32 factor	**1.43**	**1.23**
PSPTO_1299	*PSYR_1119*	*hexR*, Hex regulon repressor	**1.42**	**0.26**
PSPTO_0096	*PSYR_0230*	GntR family transcriptional regulator/aminotransferase, class I	**1.41**	**2.53**
PSPTO_3162	*PSYR_3028*	hypothetical protein PSPTO_3162	**1.36**	**2.14**
PSPTO_4920	*PSYR_0596*	LysR family transcriptional regulator	**1.34**	**0.72**
PSPTO_4550	*PSYR_4228*	GntR family transcriptional regulator	**1.34**	**0.24**
PSPTO_2533	*PSYR_2343*	GNAT family acetyltransferase	**1.33**	**0.69**
PSPTO_3040	*PSYR_2913*	LysR family transcriptional regulator	**1.27**	**0.25**
PSPTO_3161	*PSYR_3027*	protein kinase	**1.23**	**1.27**
PSPTO_3773	*PSYR_1707*	GntR family transcriptional regulator	**1.22**	**0.68**
PSPTO_2265	*PSYR_2065*	*greB-1*, transcription elongation factor GreB	**1.20**	**1.91**
PSPTO_0614	*PSYR_4560*	*nusG,* transcription antitermination protein NusG	**1.20**	**1.28**
PSPTO_1422	*PSYR_1236*	rrf2 family protein	**1.19**	**0.54**
PSPTO_3861	*PSYR_1624*	MarR family transcriptional regulator	**1.18**	**0.60**
PSPTO_0134	*PSYR_0056*	*algQ,* transcriptional regulator AlgQ	**1.13**	**1.08**
PSPTO_3319	*PSYR_3154*	TetR family transcriptional regulator	**1.12**	**0.80**
PSPTO_2172	*PSYR_1982*	LysR family transcriptional regulator	**1.11**	**0.54**
PSPTO_5242	*PSYR_0301*	*rho*, transcription termination factor Rho	**1.08**	**1.31**
PSPTO_1813	*PSYR_3583*	hypothetical protein PSPTO_1813	**1.08**	**0.27**
PSPTO_5311	*PSYR_4869*	TetR family transcriptional regulator	**1.06**	**0.29**
PSPTO_3076	*PSYR_2933*	TetR family transcriptional regulator	**1.06**	**1.23**
PSPTO_2945	*PSYR_2729*	MarR family transcriptional regulator	**1.04**	**0.14**
PSPTO_3980	*PSYR_1407*	hypothetical protein PSPTO_3980	**1.04**	**0.91**

PSPTO_1704	*PSYR_3685*	NAD-dependent deacetylase	**1.03**	**0.41**
PSPTO_2671	*PSYR_2405*	GntR family transcriptional regulator	**1.03**	**1.12**
PSPTO_0756	*PSYR_0660*	transcriptional regulator	**1.01**	**1.43**
PSPTO_0571	*PSYR_4603*	repressor protein c2	**0.94**	**1.24**
PSPTO_0721	*PSYR_0623*	transcriptional regulator	**0.83**	**1.33**
PSPTO_2121	*PSYR_1916*	RulA protein	**0.58**	**1.58**
PSPTO_2279	*PSYR_2077*	Cys regulon transcriptional activator	**0.45**	**1.17**
PSPTO_5505	*PSYR_5057*	transcriptional regulator	**-0.57**	**-1.18**
PSPTO_3863	*PSYR_1622*	LuxR transcriptional regulator	**-0.58**	**-1.31**
PSPTO_0444	*PSYR_4731*	RNA polymerase sigma-70 family protein	**-0.63**	**-1.07**
PSPTO_3196	*PSYR_3062*	LysR family transcriptional regulator	**-0.78**	**-1.29**
PSPTO_5217	*PSYR_0327*	sigma-54-binding protein	**-0.81**	**-1.64**
PSPTO_1274	*PSYR_1094*	cold shock domain family protein	**-0.95**	**-2.45**
PSPTO_3001	*PSYR_2882*	xylose operon regluatory protein	**-0.96**	**-1.05**
PSPTO_1502	*PSYR_1312*	TetR family transcriptional regulator	**-1.05**	**-1.95**
PSPTO_1645	*PSYR_3737*	MarR family transcriptional regulator	**-1.12**	**-0.11**
PSPTO_2370	*PSYR_2154*	*rbsR*, ribose operon repressor	**-1.16**	**-1.22**
PSPTO_2780	*PSYR_2508*	IclR family transcriptional regulator	**-1.18**	**-0.84**
PSPTO_3741	*PSYR_1736*	sigma-54 dependent transcriptional regulator	**-1.19**	**-0.67**
PSPTO_0465	*PSYR_4708*	AraC family transcriptional regulator	**-1.23**	**-1.43**
PSPTO_0619	*PSYR_4555*	rpoB, DNA-directed RNA polymerase subunit beta	**-1.25**	**-2.57**
PSPTO_3355	*PSYR_3185*	cold shock domain family protein	**-1.26**	**-1.43**
PSPTO_2828	*PSYR_2575*	*syrR*, transcriptional regulator SyrR	**-1.35**	**-2.11**
PSPTO_4302	*PSYR_4006*	TetR family transcriptional regulator	**-1.36**	**-0.35**
PSPTO_2751	*PSYR_2480*	LysR family transcriptional regulator	**-1.38**	**-0.61**
PSPTO_1286	*PSYR_1107*	RNA polymerase sigma-70 family protein	**-1.40**	**-0.85**
PSPTO_2833	*PSYR_2578*	LuxR family transcriptional regulator	**-1.44**	**-0.26**
PSPTO_0620	*PSYR_4554*	*rpoC*, DNA-directed RNA polymerase subunit beta\'	**-1.52**	**-3.52**
PSPTO_3025	*PSYR_2898*	DNA-binding protein	**-1.55**	**-0.73**
PSPTO_3540	*PSYR_3312*	LysR family transcriptional regulator	**-1.69**	**-0.24**
PSPTO_4775	*PSYR_4325*	GntR family transcriptional regulator/aminotransferase, class I	**-2.79**	**-0.95**
PSPTO_3467	*PSYR_3248*	sigma-54 dependent transcriptional regulator	**-2.86**	**-3.96**
**Replication, recombination and repair**				
PSPTO_1775	*PSYR_3619*	ATP-dependent RNA helicase, DEAD box family	**2.98**	**1.92**
PSPTO_3638	*PSYR_3396*	*recR*, recombination protein RecR	**2.48**	**0.72**
PSPTO_0470	*PSYR_4703*	exonuclease	**2.27**	**3.82**
PSPTO_4120	*PSYR_3856*	DinG family ATP-dependent helicase	**2.16**	**1.66**
PSPTO_5007	*PSYR_0516*	*dbpA*, ATP-independent RNA helicase	**2.15**	**1.83**

		DbpA		
PSPTO_4058	*PSYR_1376*	*mutS*, DNA mismatch repair protein MutS	**2.04**	**0.81**
PSPTO_4664	*PSYR_4297*	ATP-dependent RNA helicase rhlE	**2.01**	**2.00**
PSPTO_5070	*PSYR_0458*	ATP-dependent RNA helicase rhlE	**1.95**	**1.99**
PSPTO_2101	*PSYR_1896*	*mfd*, transcription-repair coupling factor	**1.94**	**0.59**
PSPTO_4181	*PSYR_3918*	mutT/nudix family protein	**1.91**	**1.62**
PSPTO_0700	*PSYR_0606*	*xseB*, exodeoxyribonuclease VII small subunit	**1.90**	**0.57**
PSPTO_4830	*PSYR_4370*	3-methyladenine DNA glycosylase	**1.86**	**1.45**
PSPTO_1587	*PSYR_3791*	*srmB*, ATP-dependent RNA helicase SrmB	**1.86**	**1.84**
PSPTO_1745	*PSYR_3647*	*gyrA*, DNA gyrase subunit A	**1.85**	**1.14**
PSPTO_2255	*PSYR_2060*	TatD family hydrolase	**1.83**	**1.01**
PSPTO_4033	*PSYR_1378*	*recA*, recA protein	**1.69**	**1.95**
PSPTO_3897	*PSYR_1588*	recombination associated protein rdgC	**1.68**	**1.36**
PSPTO_3711	*PSYR_1764*	*dnaQ*, DNA polymerase III subunit epsilon	**1.66**	**1.65**
PSPTO_5137	*PSYR_0398*	*priA*, primosomal protein N`	**1.65**	**0.79**
PSPTO_2478	*PSYR_2244*	*topB*, DNA topoisomerase III	**1.62**	**1.55**
PSPTO_3712	*PSYR_1763*	*rnhA*, ribonuclease HI	**1.62**	**1.42**
PSPTO_1703	*PSYR_3686*	DNA replication initiation factor	**1.60**	**0.90**
PSPTO_0113	*PSYR_0075*	*rep*, ATP-dependent DNA helicase Rep	**1.59**	**1.13**
PSPTO_4773	*PSYR_4320*	ATP-dependent helicase, N-terminal domain protein, partial	**1.52**	**0.98**
PSPTO_1446	*PSYR_1259*	*xseA*, exodeoxyribonuclease VII large subunit	**1.48**	**0.68**
PSPTO_2747	*PSYR_2476*	*xthA*, exodeoxyribonuclease III	**1.47**	**0.32**
PSPTO_0079	*PSYR_0215*	exodeoxyribonuclease III	**1.45**	**1.41**
PSPTO_4507	*PSYR_4197*	*recN*, DNA repair protein RecN	**1.39**	**0.78**
PSPTO_5516	*PSYR_5065*	*uvrD*, DNA helicase II	**1.26**	**1.25**
PSPTO_4665	*PSYR_4300*	exonuclease	**1.26**	**0.90**
PSPTO_4236	*PSYR_3970*	*ung*, uracil-DNA glycosylase	**1.24**	**1.03**
PSPTO_3161	*PSYR_3027*	protein kinase	**1.23**	**1.27**
PSPTO_5304	*PSYR_1222*	ISPsy5, transposase	**1.17**	**1.82**
PSPTO_1488	*PSYR_1298*	*recJ*, single-stranded-DNA-specific exonuclease RecJ	**1.13**	**0.97**
PSPTO_4419	*PSYR_4113*	lipoprotein	**1.13**	**0.68**
PSPTO_0344	*PSYR_0270*	*polI*, DNA polymerase I	**1.07**	**0.55**
PSPTO_0183	*PSYR_0013*	*tag*, DNA-3-methyladenine glycosidase I	**1.02**	**1.06**
PSPTO_0414	*PSYR_4761*	*mutM*, formamidopyrimidine-DNA glycosylase	**1.00**	**1.15**
PSPTO_5135	*PSYR_0400*	staphylococcal nuclease-like protein	**0.99**	**1.80**
PSPTO_2364	*PSYR_2148*	*endA*, endonuclease I	**0.97**	**1.75**
PSPTO_0656	*PSYR_4518*	*ssB*, single-stranded DNA-binding protein	**0.94**	**1.07**

PSPTO_1685	*PSYR_3704*	hypothetical protein PSPTO_1685	**0.90**	**1.22**
PSPTO_5038	*PSYR_0484*	hypothetical protein PSPTO_5038	**0.83**	**1.13**
PSPTO_3765	*PSYR_1715*	exonuclease SbcD	**-0.33**	**-1.26**
PSPTO_4149	*PSYR_3888*	endonuclease III	**-0.34**	**-1.96**
PSPTO_1548	*PSYR_1357*	ribonuclease HII	**-0.43**	**-2.07**
PSPTO_0424	*PSYR_4754*	methyltransferase	**-0.44**	**-1.52**
PSPTO_3766	*PSYR_1714*	exonuclease SbcC	**-0.47**	**-2.19**
PSPTO_3023	*PSYR_2896*	excinuclease ABC subunit C	**-0.59**	**-1.63**
PSPTO_0777	*PSYR_0681*	exodeoxyribonuclease V subunit beta	**-0.92**	**-1.22**
PSPTO_5472	*PSYR_5027*	HU family DNA-binding protein	**-1.07**	**-0.33**
PSPTO_0778	*PSYR_0682*	*recD*, exodeoxyribonuclease V subunit alpha	**-1.09**	**-1.87**
PSPTO_3357	*PSYR_3189*	mutT/nudix family protein	**-1.11**	**-0.31**
PSPTO_2624	*PSYR_2364*	hypothetical protein PSPTO_2624	**-1.15**	**-0.85**
PSPTO_3656	*PSYR_1819*	*ligA*, NAD-dependent DNA ligase LigA	**-1.16**	**-0.27**
PSPTO_3465	*PSYR_3246*	KU domain protein	**-1.17**	**-0.48**
PSPTO_4397	*PSYR_4091*	mutT/nudix family protein	**-1.22**	**-2.49**
PSPTO_3427	*PSYR_2843*	C-5 cytosine-specific DNA methylase family protein	**-1.29**	**-0.32**
PSPTO_1270	*PSYR_1090*	*holC*, DNA polymerase III subunit chi	**-1.45**	**-2.47**
PSPTO_2976	*PSYR_2759*	DNA topoisomerase, type I	**-1.62**	**-0.79**
**Cell cycle control, cell division, chromosome partitioning**				
PSPTO_3837	*PSYR_1642*	*maf-1*, maf protein	**2.17**	**1.17**
PSPTO_3511	*PSYR_3284*	*sulA*, cell division inhibitor	**1.75**	**1.44**
PSPTO_5139	*PSYR_0396*	hypothetical protein PSPTO_5139	**1.72**	**0.37**
PSPTO_1684	*PSYR_3705*	hypothetical protein PSPTO_1684	**1.37**	**1.00**
PSPTO_3873	*PSYR_1612*	*minD*, septum site-determining protein MinD	**1.18**	**1.08**
PSPTO_3874	*PSYR_1611*	*minE*, cell division topological specificity factor MinE	**1.06**	**0.79**
PSPTO_3872	*PSYR_1613*	*minC*, septum site-determining protein MinC	**1.03**	**0.66**
PSPTO_3657	*PSYR_1818*	cell division protein ZipA	**0.92**	**1.29**
PSPTO_1809	*PSYR_3587*	intracellular septation protein A	**0.74**	**1.03**
PSPTO_4146	*PSYR_3885*	ParA family protein	**0.35**	**1.11**
PSPTO_1551	*PSYR_1360*	cell cycle protein mesJ	**-0.22**	**-1.01**
PSPTO_4409	*PSYR_4103*	cell division protein FtsW	**-0.40**	**-1.56**
PSPTO_4404	*PSYR_4098*	cell division protein FtsA	**-0.98**	**-1.97**
PSPTO_1555	*PSYR_1364*	hypothetical protein PSPTO_1555	**-1.12**	**-1.26**
PSPTO_4403	*PSYR_4097*	cell division protein FtsZ	**-2.33**	**-3.01**
**Defense mechanisms**				
PSPTO_2110	*PSYR_1905*	*lolD*, lipoprotein releasing system, ATP-binding protein LolD	**1.94**	**1.33**
PSPTO_4984	*PSYR_0536*	*msbA*, lipid A ABC transporter, ATP-	**1.82**	**2.05**

		binding/permease protein		
PSPTO_4119	*PSYR_3855*	*estC*, carboxylesterase	**1.64**	**0.63**
PSPTO_3172	*PSYR_3037*	membrane protein	**1.52**	**1.45**
PSPTO_0951	*PSYR_0818*	AmpE protein	**1.35**	**0.97**
PSPTO_3855	*PSYR_1630*	ABC transporter ATP-binding protein	**1.21**	**0.97**
PSPTO_3141	*PSYR_3008*	undecaprenyl pyrophosphate phosphatase	**1.15**	**0.73**
PSPTO_0950	*PSYR_0817*	*ampD*, N-acetyl-anhydromuranmyl-L-alanine amidase	**1.06**	**0.86**
PSPTO_2832	*PSYR_2618*	*syfD*, syringafactin efflux protein SyfD	**-1.16**	**-1.49**
PSPTO_1653	*PSYR_3725*	hypothetical protein PSPTO_1653	**-1.42**	**-1.80**
PSPTO_4303	*PSYR_4007*	RND family efflux transporter MFP subunit	**-1.45**	**-0.77**
PSPTO_2831	*PSYR_2617*	*syfC*, syringafactin efflux protein SyfC	**-1.46**	**-1.03**
PSPTO_2755	*PSYR_2484*	AcrB/AcrD/AcrF family protein	**-1.57**	**-3.09**
PSPTO_2875	*PSYR_2628*	ABC transporter ATP-binding protein	**-1.73**	**-4.67**
PSPTO_4304	*PSYR_4008*	*saxB*, isothiocyanate resistance protein SaxB; isochorismatase family	**-2.16**	**-1.77**
PSPTO_1654	*PSYR_3724*	hypothetical protein PSPTO_1654	**-2.79**	**-2.88**
**Signal transduction mechanisms**				
PSPTO_0471	*PSYR_4702*	nucleotidyltransferase	**3.55**	**5.71**
PSPTO_0969	*PSYR_0835*	*dksA*, dnaK suppressor protein	**3.52**	**0.93**
PSPTO_4638	*PSYR_4273*	*cstA*, carbon starvation protein CstA	**2.07**	**2.91**
PSPTO_0306	*PSYR_0086*	sensory box/GGDEF domain/EAL domain-containing protein	**2.04**	**1.35**
PSPTO_3900	*PSYR_1585*	sensory box histidine kinase/response regulator	**2.04**	**2.01**
PSPTO_0114	*PSYR_0074*	GGDEF domain/EAL domain protein	**1.70**	**0.84**
PSPTO_2118	*PSYR_1913*	anti-anti-sigma factor	**1.70**	**0.72**
PSPTO_5398	*PSYR_4937*	sensor histidine kinase	**1.65**	**1.00**
PSPTO_2117	*PSYR_1912*	response regulator	**1.61**	**0.53**
PSPTO_4027	*PSYR_1384*	LuxR family DNA-binding response regulator	**1.57**	**0.64**
PSPTO_0536	*PSYR_4642*	sensory box/GGDEF domain/EAL domain-containing protein	**1.54**	**2.00**
PSPTO_4896	*PSYR_4439*	sensor histidine kinase	**1.42**	**0.78**
PSPTO_4374	*PSYR_4070*	*colR*, DNA-binding response regulator ColR	**1.35**	**1.23**
PSPTO_2245	*PSYR_2050*	*kdpD*, sensor protein KdpD	**1.34**	**0.90**
PSPTO_2358	*PSYR_2142*	hypothetical protein PSPTO_2358	**1.34**	**0.90**
PSPTO_3161	*PSYR_3027*	protein kinase	**1.23**	**1.27**
PSPTO_4554	*PSYR_4231*	sensor histidine kinase	**1.20**	**0.94**
PSPTO_1629	*PSYR_3751*	*csrA-1*, carbon storage regulator	**1.15**	**0.21**
PSPTO_3520	*PSYR_3293*	universal stress protein family	**1.11**	**1.21**
PSPTO_4373	*PSYR_4069*	*colS*, sensor histidine kinase ColS	**1.10**	**0.79**

PSPTO_0965	*PSYR_0832*	sensor histidine kinase	**1.10**	**0.13**
PSPTO_2215	*PSYR_2024*	*sixA*, phosphohistidine phosphatase SixA	**1.09**	**1.21**
PSPTO_1803	*PSYR_3591*	sensor histidine kinase	**1.01**	**0.47**
PSPTO_3603	*PSYR_3374*	DNA-binding heavy metal response regulator	**1.01**	**0.57**
PSPTO_0361	*PSYR_4815*	*typA*, GTP-binding protein TypA	**1.00**	**0.74**
PSPTO_0334	*PSYR_0263*	alginate biosynthesis transcriptional regulator AlgB	**0.90**	**1.18**
PSPTO_4805	*PSYR_4346*	PhoH-like protein	**0.67**	**1.29**
PSPTO_3487	*PSYR_3262*	dnaK suppressor protein	**0.59**	**1.23**
PSPTO_3526	*PSYR_3299*	LuxR family DNA-binding response regulator	**0.42**	**2.21**
PSPTO_0127	*PSYR_0063*	alginate biosynthesis regulatory protein AlgR	**0.08**	**1.04**
PSPTO_4833	*PSYR_4373*	sensory box histidine kinase	**-0.13**	**-2.41**
PSPTO_3098	*PSYR_2966*	methyl-accepting chemotaxis protein	**-0.26**	**-1.21**
PSPTO_1152	*PSYR_0994*	HDIG domain protein	**-0.37**	**-2.13**
PSPTO_1494	*PSYR_1304*	chemotaxis protein CheW	**-0.39**	**-1.24**
PSPTO_1499	*PSYR_1309*	response regulator/GGDEF domain-containing protein	**-0.42**	**-1.56**
PSPTO_1737	*PSYR_3655*	sensory box/GGDEF domain/EAL domain-containing protein	**-0.46**	**-1.06**
PSPTO_1291	*PSYR_1112*	sensor histidine kinase	**-0.47**	**-2.17**
PSPTO_1290	*PSYR_1111*	DNA-binding response regulator	**-0.48**	**-1.07**
PSPTO_5030	*PSYR_0492*	sensor histidine kinase/response regulator	**-0.52**	**-1.34**
PSPTO_2713	*PSYR_2446*	chemotaxis protein methyltransferase CheR	**-0.53**	**-1.11**
PSPTO_1004	*PSYR_0870*	diguanylate cyclase	**-0.53**	**-2.18**
PSPTO_1980	*PSYR_3436*	chemotaxis protein CheY	**-0.57**	**-1.05**
PSPTO_1498	*PSYR_1308*	protein-glutamate methylesterase CheB	**-0.58**	**-1.32**
PSPTO_4292	*PSYR_3995*	sigma-54 dependent transcriptional regulator/response regulator	**-0.60**	**-2.35**
PSPTO_4868	*PSYR_4408*	sensor histidine kinase/response regulator RetS	**-0.62**	**-2.64**
PSPTO_1963	*PSYR_3452*	STAS domain-containing protein	**-0.64**	**-1.46**
PSPTO_2278	*PSYR_2076*	universal stress protein family	**-0.67**	**-1.21**
PSPTO_1981	*PSYR_3435*	chemotaxis protein CheZ	**-0.69**	**-1.29**
PSPTO_1982	*PSYR_3434*	chemotaxis sensor histidine kinase CheA	**-0.71**	**-1.04**
PSPTO_4543	*PSYR_4221*	GAF domain/GGDEF domain/EAL domain protein	**-0.73**	**-2.19**
PSPTO_1495	*PSYR_1305*	chemotaxis protein methyltransferase CheR	**-0.80**	**-1.76**
PSPTO_1496	*PSYR_1306*	chemotaxis protein CheW	**-0.84**	**-1.96**
PSPTO_4837	*PSYR_4377*	response regulator	**-0.86**	**-2.06**

PSPTO_1964	*PSYR_3451*	response regulator	**-0.88**	**-1.42**
PSPTO_5014	*PSYR_0509*	response regulator/sensory box/GGDEF domain/EAL domain protein	**-0.88**	**-2.22**
PSPTO_4291	*PSYR_3994*	sensor histidine kinase	**-0.94**	**-1.78**
PSPTO_1983	*PSYR_3433*	protein-glutamate methylesterase CheB	**-0.95**	**-1.82**
PSPTO_1497	*PSYR_1307*	sensor histidine kinase/response regulator	**-0.97**	**-1.70**
PSPTO_2479	*PSYR_2245*	hypothetical protein PSPTO_2479	**-0.99**	**-2.38**
PSPTO_3246	*PSYR_3093*	hypothetical protein PSPTO_3246	**-1.03**	**-1.87**
PSPTO_4293	*PSYR_3996*	sensory box DNA/response regulator	**-1.11**	**-1.32**
PSPTO_2642	*PSYR_2374*	sensor histidine kinase	**-1.14**	**-0.52**
PSPTO_2896	*PSYR_2700*	sensory box histidine kinase/response regulator	**-1.18**	**-0.58**
PSPTO_0915	*PSYR_0788*	cheY-1, chemotaxis protein CheY	**-1.21**	**-1.28**
PSPTO_3699	*PSYR_1776*	methyl-accepting chemotaxis protein	**-1.21**	**-1.81**
PSPTO_0339	*PSYR_0266*	diguanylate cyclase	**-1.22**	**-2.01**
PSPTO_2591	*PSYR_2281*	diguanylate cyclase	**-1.27**	**-1.40**
PSPTO_1348	*PSYR_1159*	sensory box/GGDEF domain/EAL domain-containing protein	**-1.27**	**-1.29**
PSPTO_4365	*PSYR_4060*	GGDEF domain/EAL domain protein	**-1.34**	**-1.17**
PSPTO_1278	*PSYR_1098*	sensory box protein/response regulator	**-1.35**	**-2.77**
PSPTO_2128	*PSYR_1938*	response regulator	**-1.40**	**-0.44**
PSPTO_0406	*PSYR_4770*	sensory box/GGDEF domain/EAL domain-containing protein	**-1.42**	**-1.35**
PSPTO_2448	*PSYR_2220*	methyl-accepting chemotaxis protein	**-1.52**	**-1.62**
PSPTO_0913	*PSYR_0786*	*cheA-1*, chemotaxis sensor histidine kinase CheA	**-1.56**	**-1.81**
PSPTO_0910	*PSYR_0783*	*cheR-1*, chemotaxis protein methyltransferase CheR	**-1.65**	**-1.38**
PSPTO_1844	*PSYR_3554*	*csrA-2*, carbon storage regulator	**-1.76**	**-1.29**
PSPTO_1870	*PSYR_3532*	sensory box histidine kinase/response regulator	**-1.93**	**-1.60**
PSPTO_0911	*PSYR_0784*	*cheW-1*, chemotaxis protein CheW	**-1.97**	**-2.49**
PSPTO_4371	*PSYR_4067*	*inaA*, inaA protein	**-2.10**	**-0.72**
PSPTO_0912	*PSYR_0785*	methyl-accepting chemotaxis protein	**-2.11**	**-3.17**
PSPTO_1246	*PSYR_1066*	PhoH-like protein	**-2.47**	**-1.77**
PSPTO_0547	*PSYR_4631*	hypothetical protein PSPTO_0547	**-3.02**	**-2.46**
**Cell wall/membrane/envelope biogenesis**				
PSPTO_0977	*PSYR_0842*	penicillin-binding protein	**2.53**	**1.54**
PSPTO_2109	*PSYR_1904*	*lolC*, lipoprotein releasing system transmembrane protein LolC	**2.38**	**2.37**
PSPTO_0431	*PSYR_4743*	*mtgA*, monofunctional biosynthetic peptidoglycan transglycosylase	**2.20**	**1.68**
PSPTO_2189	*PSYR_1999*	ompA family protein	**2.02**	**1.93**
PSPTO_4448	*PSYR_4142*	sugar isomerase	**1.99**	**1.35**

PSPTO_4220	*PSYR_3954*	*lepA*, GTP-binding protein LepA	**1.94**	**0.64**
PSPTO_5283	*PSYR_4841*	lgt, prolipoprotein diacylglyceryl transferase	**1.92**	**2.22**
PSPTO_4509	*PSYR_4199*	*omlA*, outer membrane lipoprotein OmlA	**1.70**	**0.46**
PSPTO_4182	*PSYR_3919*	D-alanine--D-alanine ligase	**1.69**	**1.50**
PSPTO_4945	*PSYR_0569*	N-acetylmuramoyl-L-alanine amidase family protein	**1.61**	**1.04**
PSPTO_4115	*PSYR_3852*	lipoprotein SlyB	**1.61**	**0.35**
PSPTO_5003	*PSYR_0520*	*rfaF*, ADP-heptose--LPS heptosyltransferase II	**1.60**	**1.16**
PSPTO_2056	*PSYR_1865*	membrane protein	**1.59**	**0.61**
PSPTO_3871	*PSYR_1614*	*htrB*, lipid A biosynthesis lauroyl acyltransferase	**1.59**	**1.65**
PSPTO_1330	*PSYR_1146*	glycosyl transferase family protein	**1.52**	**0.79**
PSPTO_0338	*PSYR_0265*	N-acetylmuramoyl-L-alanine amidase family protein	**1.51**	**0.86**
PSPTO_4392	*PSYR_4086*	mechanosensitive ion channel family protein	**1.49**	**1.40**
PSPTO_4471	*PSYR_4162*	*mreC*, rod shape-determining protein MreC	**1.49**	**0.54**
PSPTO_4985	*PSYR_0535*	toluene tolerance protein	**1.48**	**1.37**
PSPTO_0139	*PSYR_0051*	hypothetical protein PSPTO_0139	**1.45**	**1.25**
PSPTO_3113	*PSYR_2980*	*galU*, UTP-glucose-1-phosphate uridylyltransferase	**1.36**	**2.14**
PSPTO_3952	*PSYR_1549*	lipoprotein	**1.33**	**0.31**
PSPTO_1025	*PSYR_0880*	transglycosylase	**1.32**	**1.20**
PSPTO_0826	*PSYR_0725*	competence lipoprotein ComL	**1.31**	**1.53**
PSPTO_0182	*PSYR_0014*	lipid A biosynthesis lauroyl acyltransferase	**1.30**	**1.61**
PSPTO_5133	*PSYR_0402*	penicillin-binding protein	**1.29**	**0.86**
PSPTO_2111	*PSYR_1906*	*lolE*, lipoprotein releasing system transmembrane protein LolE	**1.23**	**1.20**
PSPTO_4978	*PSYR_0542*	*kdtA*, 3-deoxy-D-manno-octulosonic-acid transferase	**1.20**	**1.61**
PSPTO_2509	*PSYR_2313*	penicillin-binding protein	**1.20**	**1.07**
PSPTO_4026	*PSYR_1385*	*dgkA*, diacylglycerol kinase	**1.18**	**0.74**
PSPTO_1574	*PSYR_3803*	mechanosensitive ion channel family protein	**1.18**	**0.24**
PSPTO_5341	*PSYR_4900*	hypothetical protein PSPTO_5341	**1.15**	**1.18**
PSPTO_3845	*PSYR_1635*	*lpxK*, tetraacyldisaccharide 4\'-kinase	**1.14**	**0.51**
PSPTO_5050	*PSYR_0473*	metW, metW protein	**1.13**	**0.72**
PSPTO_5193	*PSYR_0344*	RND family efflux transporter MFP subunit	**1.12**	**0.30**
PSPTO_1458	*PSYR_1268*	periplasmic binding	**1.11**	**1.31**

		domain/transglycosylase SLT domain fusion protein		
PSPTO_2749	*PSYR_2478*	hypothetical protein PSPTO_2749	**1.10**	**1.15**
PSPTO_5002	*PSYR_0521*	*waaC*, lipopolysaccharide heptosyltransferase	**1.10**	**0.67**
PSPTO_4825	*PSYR_4365*	penicillin-binding protein	**1.05**	**1.28**
PSPTO_2681	*PSYR_2414*	*pbpG*, penicillin-binding protein 7	**1.03**	**0.90**
PSPTO_1445	*PSYR_1258*	peptidase, M23/M37 family	**1.02**	**0.66**
PSPTO_0377	*PSYR_4801*	metal ion efflux outer membrane protein	**0.97**	**1.48**
PSPTO_2297	*PSYR_2095*	membrane protein	**0.85**	**1.03**
PSPTO_0729	*PSYR_0630*	UDP-N-acetylmuramate:L-alanyl-gamma-D-glutamyl-meso-diaminopimelate ligase	**0.77**	**1.11**
PSPTO_2767	*PSYR_2496*	lipopolysaccharide core biosynthesis domain protein	**0.70**	**2.81**
PSPTO_5480	*PSYR_5035*	peptidase, M23/M37 family	**0.69**	**1.06**
PSPTO_0369	*PSYR_4807*	outer membrane porin, OprD family	**0.65**	**4.17**
PSPTO_0736	*PSYR_0637*	membrane protein	**0.64**	**1.10**
PSPTO_4441	*PSYR_4135*	UDP-N-acetylglucosamine 1-carboxyvinyltransferase	**0.60**	**1.01**
PSPTO_0103	*PSYR_0237*	alanine racemase	**0.59**	**1.16**
PSPTO_5595	*PSYR_5117*	glucosamine--fructose-6-phosphate aminotransferase	**0.57**	**2.24**
PSPTO_5170	*PSYR_0368*	lipoprotein Blc	**0.30**	**2.06**
PSPTO_4410	*PSYR_4104*	UDP-N-acetylmuramoylalanine--D-glutamate ligase	**-0.09**	**-1.15**
PSPTO_1547	*PSYR_1356*	lipid A disaccharide synthase	**-0.16**	**-2.51**
PSPTO_1923	*PSYR_3490*	hypothetical protein PSPTO_1923	**-0.23**	**-1.44**
PSPTO_4408	*PSYR_4102*	UDP-N-acetylglucosamine-N-acetylmuramyl- (pentapeptide) pyrophosphoryl-undecaprenol N-acetylglucosamine transferase	**-0.29**	**-1.48**
PSPTO_3301	*PSYR_3131*	RND family efflux transporter MFP subunit	**-0.37**	**-4.87**
PSPTO_4407	*PSYR_4101*	UDP-N-acetylmuramate--L-alanine ligase	**-0.48**	**-1.59**
PSPTO_0607	*PSYR_4566*	peptidase, M23/M37 family	**-0.50**	**-1.31**
PSPTO_2899	*PSYR_2703*	hypothetical protein PSPTO_2899	**-0.59**	**-1.17**
PSPTO_1544	*PSYR_1353*	UDP-3-O	**-0.59**	**-2.67**
PSPTO_2891	*PSYR_2696*	UDP-glucose 6-dehydrogenase	**-0.61**	**-2.16**
PSPTO_3537	*PSYR_3309*	membrane protein PslJ	**-0.66**	**-2.15**
PSPTO_4406	*PSYR_4100*	D-alanine--D-alanine ligase	**-0.72**	**-1.65**
PSPTO_4081	*PSYR_4988*	Rhs family protein	**-0.74**	**-3.30**
PSPTO_1620	*PSYR_3760*	hypothetical protein PSPTO_1620	**-0.81**	**-1.63**
PSPTO_1116	*PSYR_0956*	cyclopropane-fatty-acyl-phospholipid synthase	**-0.88**	**-2.19**

PSPTO_3530	*PSYR_3302*	mannose-1-phosphate guanylyltransferase/mannose-6-phosphate isomerase PslB	**-0.97**	**-1.49**
PSPTO_1231	*PSYR_1051*	insecticidal toxin protein	**-1.02**	**-0.69**
PSPTO_3328	*PSYR_3159*	*tliF*, ABC transporter permease	**-1.08**	**-0.89**
PSPTO_4405	*PSYR_4099*	cell division protein FtsQ	**-1.08**	**-1.83**
PSPTO_2343	*PSYR_2127*	outer membrane porin, OprD family	**-1.10**	**-0.84**
PSPTO_0373	*PSYR_4805*	Rhs family protein	**-1.22**	**-1.08**
PSPTO_3885	*PSYR_1599*	*prc*, tail-specific protease	**-1.24**	**-1.02**
PSPTO_3536	*PSYR_3308*	glycosyl transferase, group 1 family protein PslI	**-1.32**	**-3.50**
PSPTO_2481	*PSYR_2287*	*tonB-2*, tonB protein	**-1.36**	**-1.30**
PSPTO_4305	*PSYR_4009*	outer membrane efflux protein	**-1.37**	**-0.39**
PSPTO_4344	*PSYR_4037*	insecticidal toxin protein	**-1.44**	**-0.72**
PSPTO_4343	*PSYR_4036*	insecticidal toxin protein	**-1.45**	**-1.21**
PSPTO_3531	*PSYR_3303*	lipoprotein PslD	**-1.49**	**-1.83**
PSPTO_1947	*PSYR_3468*	glycosyl transferase family protein	**-1.58**	**-2.88**
PSPTO_2011	*PSYR_3408*	autotransporter	**-1.62**	**-0.89**
PSPTO_3535	*PSYR_3307*	glycosyl transferase, group 1 family protein PslH	**-1.69**	**-3.46**
PSPTO_3533	*PSYR_3305*	glycosyl transferase, group 1 family protein PslF	**-1.73**	**-4.04**
PSPTO_4340	*PSYR_4033*	insecticidal toxin protein	**-1.79**	**-1.19**
PSPTO_1632	*PSYR_3748*	GNAT family acetyltransferase	**-1.87**	**-0.36**
PSPTO_4560	*PSYR_4237*	outer membrane porin, OprD family	**-1.96**	**-2.26**
PSPTO_3532	*PSYR_3304*	exopolysaccharide biosynthesis protein PslE	**-1.97**	**-2.62**
PSPTO_1242	*PSYR_1062*	alginate biosynthesis protein Alg8	**-1.99**	**-0.40**
PSPTO_2756	*PSYR_2485*	outer membrane efflux protein	**-2.00**	**-3.15**
PSPTO_1296	*PSYR_1117*	*oprB*, porin B	**-2.29**	**-2.58**
PSPTO_3987	*PSYR_1400*	*oprD*, porin D	**-2.45**	**-0.41**
PSPTO_1946	*PSYR_3469*	glycosyl transferase family protein	**-2.60**	**-3.36**
PSPTO_5436	*PSYR_4382*	Rhs element Vgr protein	**-2.67**	**-0.33**
PSPTO_1241	*PSYR_1061*	alginate biosynthesis protein Alg44	**-2.72**	**-0.89**
PSPTO_1240	*PSYR_1060*	*algK*, alginate biosynthesis protein AlgK	**-3.36**	**-1.56**
PSPTO_1239	*PSYR_1059*	*algE*, alginate biosynthesis protein AlgE	**-3.49**	**-1.59**
PSPTO_1238	*PSYR_1058*	*algG*, alginate biosynthesis protein AlgG	**-3.61**	**-2.15**
PSPTO_1237	*PSYR_1057*	*algX*, alginate biosynthesis protein AlgX	**-3.65**	**-0.99**
PSPTO_1235	*PSYR_1055*	*algI*, alginate biosynthesis protein AlgI	**-4.10**	**-2.07**
PSPTO_1234	*PSYR_1054*	*algJ*, alginate biosynthesis protein AlgJ	**-4.56**	**-2.24**
PSPTO_1233	*PSYR_1053*	*algF*, alginate biosynthesis protein AlgF	**-4.74**	**-3.29**
PSPTO_1232	*PSYR_1052*	*algA*, alginate biosynthesis protein AlgA	**-5.04**	**-3.25**
**Cell motility**				
PSPTO_1966	*PSYR_3449*	flagellar hook-length control protein FliK	**-0.15**	**-1.00**

PSPTO_1961	*PSYR_3454*	flagellum-specific ATP synthase FliI	**-0.17**	**-1.76**
PSPTO_1960	*PSYR_3455*	flagellar assembly protein H	**-0.18**	**-1.33**
PSPTO_1974	*PSYR_3442*	flagellar biosynthetic protein FliR	**-0.31**	**-1.57**
PSPTO_1943	*PSYR_3472*	peptidoglycan hydrolase FlgJ	**-0.34**	**-1.61**
PSPTO_1494	*PSYR_1304*	chemotaxis protein CheW	**-0.39**	**-1.24**
PSPTO_2713	*PSYR_2446*	chemotaxis protein methyltransferase CheR	**-0.53**	**-1.11**
PSPTO_1933	*PSYR_3481*	flagellar basal-body rod protein FlgB	**-0.54**	**-2.09**
PSPTO_1962	*PSYR_3453*	flagellar biosynthesis chaperone	**-0.55**	**-2.04**
PSPTO_1934	*PSYR_3480*	flagellar basal body rod protein FlgC	**-0.71**	**-2.18**
PSPTO_1495	*PSYR_1305*	chemotaxis protein methyltransferase CheR	**-0.80**	**-1.76**
PSPTO_1496	*PSYR_1306*	chemotaxis protein CheW	**-0.84**	**-1.96**
PSPTO_1975	*PSYR_3441*	flagellar biosynthetic protein FlhB	**-0.89**	**-1.74**
PSPTO_1984	*PSYR_3432*	flagellar motor protein	**-0.94**	**-2.25**
PSPTO_1944	*PSYR_3471*	*flgK*, flagellar hook-associated protein FlgK	**-1.11**	**-0.88**
PSPTO_1935	*PSYR_3479*	*flgD*, basal-body rod modification protein FlgD	**-1.17**	**-2.42**
PSPTO_1985	*PSYR_3431*	motB protein	**-1.28**	**-2.91**
PSPTO_1951	*PSYR_3464*	*fliD*, flagellar hook-associated protein FliD	**-1.40**	**-2.84**
PSPTO_0910	*PSYR_0783*	*cheR-1*, chemotaxis protein methyltransferase CheR	**-1.65**	**-1.38**
PSPTO_0815	*PSYR_0719*	type IV pilus-associated protein	**-1.70**	**-0.46**
PSPTO_0911	*PSYR_0784*	*cheW-1*, chemotaxis protein CheW	**-1.97**	**-2.49**
PSPTO_1945	*PSYR_3470*	*flgL*, flagellar hook-associated protein FlgL	**-2.07**	**-3.26**
PSPTO_0912	*PSYR_0785*	methyl-accepting chemotaxis protein	**-2.11**	**-3.17**
PSPTO_0924	*PSYR_0796*	*pilD*, type IV pilus prepilin peptidase PilD	**-2.12**	**-2.69**
PSPTO_1949	*PSYR_3466*	*fliC*, flagellin	**-2.32**	**-2.14**
PSPTO_1936	*PSYR_3478*	*flgE-1*, flagellar hook protein FlgE	**-2.36**	**-3.39**
PSPTO_1950	*PSYR_3465*	flagellin FlaG	**-2.52**	**-2.98**
**Intracellular trafficking, secretion, and vesicular transport**				
PSPTO_3632	*PSYR_3390*	*ccmD*, heme exporter protein CcmD	**1.66**	**1.61**
PSPTO_4493	*PSYR_4183*	*secG*, preprotein translocase subunit SecG	**1.54**	**1.85**
PSPTO_4852	*PSYR_4392*	hypothetical protein PSPTO_4852	**1.30**	**0.49**
PSPTO_5612	*PSYR_5134*	inner membrane protein, 60 kDa	**1.18**	**0.48**
PSPTO_1415	*PSYR_1230*	*secD*, protein-export membrane protein SecD	**1.18**	**0.03**
PSPTO_5324	*PSYR_4882*	*secB*, protein-export protein SecB	**1.14**	**1.10**
PSPTO_5125	*PSYR_0410*	hypothetical protein PSPTO_5125	**1.12**	**1.18**
PSPTO_4853	*PSYR_4393*	type II/IV secretion system protein	**1.09**	**0.38**
PSPTO_4400	*PSYR_4094*	*secA*, preprotein translocase subunit SecA	**1.02**	**0.21**

PSPTO_3838	*PSYR_1641*	signal peptide peptidase SppA, 36K type	**0.71**	**1.74**
PSPTO_1961	*PSYR_3454*	flagellum-specific ATP synthase FliI	**-0.17**	**-1.76**
PSPTO_4834	*PSYR_4374*	hypothetical protein PSPTO_4834	**-0.44**	**-2.71**
PSPTO_0926	*PSYR_0798*	type IV pilus biogenesis protein PilB	**-0.49**	**-1.90**
PSPTO_2011	*PSYR_3408*	autotransporter	**-1.62**	**-0.89**
PSPTO_0816	*PSYR_0720*	type IV pilus biogenesis protein	**-2.00**	**-0.17**
PSPTO_0924	*PSYR_0796*	*pilD*, type IV pilus prepilin peptidase PilD	**-2.12**	**-2.69**
PSPTO_0925	*PSYR_0797*	*pilC*, type IV pilus biogenesis protein PilC	**-2.39**	**-3.20**
**Posttranslational modification, protein turnover, chaperones**				
PSPTO_4515	*PSYR_4203*	*smpB*, SsrA-binding protein	**2.97**	**1.74**
PSPTO_3028	*PSYR_2901*	*ppiC-1*, peptidyl-prolyl cis-trans isomerase C	**2.59**	**1.32**
PSPTO_1479	*PSYR_1289*	*dsbC*, thiol:disulfide interchange protein DsbC	**2.13**	**1.63**
PSPTO_4424	*PSYR_4118*	*sspA*, stringent starvation protein A	**2.07**	**1.16**
PSPTO_2285	*PSYR_2083*	hypothetical protein PSPTO_2285	**1.99**	**2.41**
PSPTO_3631	*PSYR_3389*	*ccmE*, cytochrome c-type biogenesis protein CcmE	**1.96**	**1.53**
PSPTO_1471	*PSYR_1280*	membrane protein	**1.95**	**0.87**
PSPTO_3107	*PSYR_2974*	*ahpF*, alkyl hydroperoxide reductase subunit F	**1.86**	**0.41**
PSPTO_2529	*PSYR_2340*	FKBP-type peptidyl-prolyl cis-trans isomerase	**1.82**	**0.62**
PSPTO_3856	*PSYR_1629*	glutathione S-transferase family protein	**1.81**	**1.03**
PSPTO_3727	*PSYR_1746*	*tig*, trigger factor	**1.81**	**1.23**
PSPTO_4435	*PSYR_4130*	trypsin domain-containing protein	**1.80**	**1.20**
PSPTO_3259	*PSYR_3097*	peptidase, U32 family	**1.80**	**0.83**
PSPTO_0540	*PSYR_4638*	*gcp*, O-sialoglycoprotein endopeptidase	**1.78**	**0.86**
PSPTO_3634	*PSYR_3392*	*ccmB*, heme exporter protein CcmB	**1.73**	**1.41**
PSPTO_4504	*PSYR_4194*	*dnaJ*, dnaJ protein	**1.62**	**1.07**
PSPTO_4581	*PSYR_4255*	FKBP-type peptidyl-prolyl cis-trans isomerase	**1.51**	**0.76**
PSPTO_4640	*PSYR_4275*	*radA*, DNA repair protein RadA	**1.47**	**0.74**
PSPTO_3633	*PSYR_3391*	*ccmC*, heme exporter protein CcmC	**1.46**	**1.25**
PSPTO_4992	*PSYR_0528*	carbamoyltransferase family protein	**1.45**	**0.93**
PSPTO_2034	*PSYR_1844*	hypothetical protein PSPTO_2034	**1.44**	**1.12**
PSPTO_4161	*PSYR_3898*	glutaredoxin-like protein	**1.40**	**0.51**
PSPTO_3890	*PSYR_1594*	FKBP-type peptidyl-prolyl cis-trans isomerase	**1.39**	**2.53**
PSPTO_5243	*PSYR_0300*	*trx-2*, thioredoxin	**1.38**	**1.13**
PSPTO_0341	*PSYR_0268*	*dsbA*, thiol:disulfide interchange protein DsbA	**1.38**	**2.26**
PSPTO_1178	*PSYR_1016*	*trxB*, thioredoxin reductase	**1.30**	**1.67**
PSPTO_4858	*PSYR_4398*	*dsbD*, thiol:disulfide interchange protein	**1.30**	**1.19**

		DsbD		
PSPTO_3034	*PSYR_2907*	*ppiC-2*, peptidyl-prolyl cis-trans isomerase C	**1.29**	**1.29**
PSPTO_3635	*PSYR_3393*	*ccmA*, heme exporter protein CcmA	**1.29**	**1.60**
PSPTO_1778	*PSYR_3616*	*htpX*, heat shock protein HtpX	**1.29**	**1.84**
PSPTO_4898	*PSYR_4441*	*yegD*, heat shock protein YegD	**1.28**	**0.98**
PSPTO_1689	*PSYR_3700*	lipoprotein	**1.23**	**1.85**
PSPTO_5325	*PSYR_4883*	glutaredoxin	**1.21**	**1.49**
PSPTO_3955	*PSYR_1546*	AhpC/Tsa family protein	**1.21**	**0.83**
PSPTO_3630	*PSYR_3388*	*ccmF*, cytochrome c-type biogenesis protein CcmF	**1.17**	**0.99**
PSPTO_5140	*PSYR_0395*	*hslV*, heat shock protein HslV	**1.13**	**0.23**
PSPTO_0135	*PSYR_0055*	FKBP-type peptidyl-prolyl cis-trans isomerase	**1.09**	**0.65**
PSPTO_3901	*PSYR_1584*	hypothetical protein PSPTO_3901	**1.01**	**0.96**
PSPTO_0238	*PSYR_0169*	*hslO*, chaperonin, 33 kDa	**1.01**	**0.74**
PSPTO_4912	*PSYR_4454*	urease accessory protein	**0.99**	**2.78**
PSPTO_2735	*PSYR_2466*	*yhgI*, yhgI protein	**0.96**	**1.24**
PSPTO_4910	*PSYR_4452*	urease accessory protein UreF	**0.91**	**4.78**
PSPTO_0426	*PSYR_4752*	peptidase, M16 family	**0.72**	**1.25**
PSPTO_3838	*PSYR_1641*	signal peptide peptidase SppA, 36K type	**0.71**	**1.74**
PSPTO_1144	*PSYR_0984*	lipoprotein	**0.27**	**1.02**
PSPTO_4909	*PSYR_4451*	urease accessory protein UreE	**0.27**	**4.00**
PSPTO_4377	*PSYR_4073*	chaperonin, 10 kDa	**0.25**	**1.56**
PSPTO_2170	*PSYR_1980*	heat shock protein, Hsp20 family	**0.16**	**3.86**
PSPTO_4376	*PSYR_4072*	chaperonin, 60 kDa	**0.07**	**1.26**
PSPTO_0553	*PSYR_4625*	peptidyl-prolyl cis-trans isomerase SurA	**-0.37**	**-1.37**
PSPTO_1650	*PSYR_3733*	autotransporter	**-0.65**	**-1.13**
PSPTO_2408	*PSYR_2195*	urease accessory protein UreD	**-0.68**	**-1.13**
PSPTO_1365	*PSYR_1175*	glutathione S-transferase	**-1.02**	**-0.80**
PSPTO_0606	*PSYR_4567*	hypothetical protein PSPTO_0606	**-1.20**	**-2.02**
PSPTO_5317	*PSYR_4877*	antioxidant, AhpC/Tsa family	**-1.33**	**-3.13**
PSPTO_4398	*PSYR_4092*	glutathione S-transferase family protein	**-1.34**	**-2.53**
PSPTO_5425	*PSYR_4958*	clpB protein	**-1.89**	**-3.45**
PSPTO_0924	*PSYR_0796*	*pilD*, type IV pilus prepilin peptidase PilD	**-2.12**	**-2.69**
**Energy production and conversion**				
PSPTO_4059	*PSYR_1375*	*fdxA*, ferredoxin	**2.81**	**1.60**
PSPTO_0722	*PSYR_0624*	*ppa-1*, inorganic pyrophosphatase	**2.29**	**1.25**
PSPTO_1136	*PSYR_0976*	*mqo*, malate:quinone oxidoreductase	**2.28**	**1.52**
PSPTO_5605	*PSYR_5127*	*atpB*, F0F1 ATP synthase subunit A	**2.07**	**1.98**
PSPTO_3920	*PSYR_1565*	D-isomer specific 2-hydroxyacid dehydrogenase family protein	**1.99**	**0.70**
PSPTO_0239	*PSYR_0168*	*pckA*, phosphoenolpyruvate carboxykinase	**1.96**	**1.55**

PSPTO_4981	*PSYR_0539*	oxidoreductase, aldo/keto reductase family	**1.86**	**1.55**
PSPTO_2194	*PSYR_2004*	*gltA*, citrate synthase I	**1.75**	**1.06**
PSPTO_5606	*PSYR_5128*	*atpI*, F0F1 ATP synthase subunit I	**1.62**	**0.84**
PSPTO_1325	*PSYR_1141*	*cyoA*, cytochrome o ubiquinol oxidase subunit II	**1.51**	**1.13**
PSPTO_5293	*PSYR_4851*	oxidoreductase, FAD-binding protein	**1.48**	**1.58**
PSPTO_1802	*PSYR_3592*	nitroreductase family protein	**1.34**	**0.15**
PSPTO_2107	*PSYR_1902*	glycerophosphoryl diester phosphodiesterase	**1.29**	**1.26**
PSPTO_5598	*PSYR_5120*	*atpC*, F0F1 ATP synthase subunit epsilon	**1.28**	**0.08**
PSPTO_0416	*PSYR_4759*	ferredoxin	**1.28**	**1.03**
PSPTO_5603	*PSYR_5125*	*atpF*, F0F1 ATP synthase subunit B	**1.26**	**1.02**
PSPTO_3039	*PSYR_2912*	alcohol dehydrogenase	**1.22**	**0.65**
PSPTO_4928	*PSYR_0587*	hypothetical protein PSPTO_4928	**1.22**	**0.16**
PSPTO_1173	*PSYR_1011*	oxidoreductase FAD/FMN-binding protein	**1.19**	**1.77**
PSPTO_5602	*PSYR_5124*	*atpH*, F0F1 ATP synthase subunit delta	**1.17**	**0.91**
PSPTO_2182	*PSYR_1992*	oxidoreductase zinc-binding protein	**1.13**	**1.10**
PSPTO_5601	*PSYR_5123*	*atpA*, F0F1 ATP synthase subunit alpha	**1.07**	**0.80**
PSPTO_0754	*PSYR_0658*	alcohol dehydrogenase	**1.06**	**0.88**
PSPTO_2195	*PSYR_2005*	*sdhC*, succinate dehydrogenase, cytochrome b556 subunit	**1.03**	**1.37**
PSPTO_2070	*PSYR_1880*	cytochrome c2	**1.01**	**0.68**
PSPTO_3924	*PSYR_1561*	*sfcA*, malate dehydrogenase	**1.01**	**0.60**
PSPTO_2213	*PSYR_2022*	*gpsA*, glycerol-3-phosphate dehydrogenase (NAD(P)+)	**0.95**	**1.57**
PSPTO_4339	*PSYR_4031*	fumarate hydratase, class I	**0.86**	**1.06**
PSPTO_1558	*PSYR_1367*	alcohol dehydrogenase, class III	**0.78**	**1.13**
PSPTO_0098	*PSYR_0232*	hypothetical protein PSPTO_0098	**0.71**	**1.32**
PSPTO_2196	*PSYR_2006*	succinate dehydrogenase, hydrophobic membrane anchor protein	**0.70**	**1.38**
PSPTO_1337	*PSYR_1153*	oxidoreductase zinc-binding protein	**0.66**	**1.11**
PSPTO_0728	*PSYR_0629*	aldehyde dehydrogenase family protein	**0.61**	**2.70**
PSPTO_2197	*PSYR_2007*	succinate dehydrogenase, flavoprotein subunit	**0.59**	**1.10**
PSPTO_2660	*PSYR_2394*	aldehyde dehydrogenase family protein	**0.59**	**1.01**
PSPTO_0441	*PSYR_4733*	betaine aldehyde dehydrogenase BADH	**0.33**	**1.24**
PSPTO_0300	*PSYR_0091*	succinate-semialdehyde dehydrogenase	**0.10**	**1.59**
PSPTO_3106	*PSYR_2973*	lactoylglutathione lyase	**0.10**	**1.12**
PSPTO_3369	*PSYR_3201*	NADH dehydrogenase I subunit F	**-0.10**	**-1.27**
PSPTO_3860	*PSYR_1625*	pyruvate dehydrogenase, E1 component	**-0.28**	**-1.36**
PSPTO_5104	*PSYR_0428*	hypothetical protein PSPTO_5104	**-0.39**	**-1.54**
PSPTO_4168	*PSYR_3905*	glycerol kinase	**-0.47**	**-1.38**

PSPTO_2645	*PSYR_2377*	hypothetical protein PSPTO_2645	**-0.59**	**-1.03**
PSPTO_3370	*PSYR_3202*	NADH dehydrogenase subunit G	**-0.60**	**-2.10**
PSPTO_2200	*PSYR_2010*	dihydrolipoamide succinyltransferase	**-0.60**	**-1.03**
PSPTO_3376	*PSYR_3208*	NADH:ubiquinone oxidoreductase subunit M	**-0.66**	**-2.72**
PSPTO_3375	*PSYR_3207*	NADH:ubiquinone oxidoreductase subunit L	**-0.67**	**-2.88**
PSPTO_3374	*PSYR_3206*	NADH:ubiquinone oxidoreductase subunit K	**-0.70**	**-2.71**
PSPTO_3371	*PSYR_3203*	NADH:ubiquinone oxidoreductase subunit H	**-0.75**	**-2.36**
PSPTO_2730	*PSYR_2462*	sulfite reductase	**-0.77**	**-1.88**
PSPTO_3377	*PSYR_3209*	NADH:ubiquinone oxidoreductase subunit N	**-0.79**	**-2.44**
PSPTO_3373	*PSYR_3205*	NADH:ubiquinone oxidoreductase subunit J	**-0.85**	**-2.56**
PSPTO_2588	*PSYR_2278*	luciferase family protein	**-0.88**	**-1.95**
PSPTO_2201	*PSYR_2011*	dihydrolipoamide dehydrogenase	**-0.94**	**-1.02**
PSPTO_0455	*PSYR_4718*	glutathione-independent formaldehyde dehydrogenase	**-0.98**	**-1.53**
PSPTO_2957	*PSYR_2742*	methanol dehydrogenase, NAD-dependent	**-1.00**	**-0.77**
PSPTO_2519	*PSYR_2330*	oxidoreductase, FAD-binding protein	**-1.09**	**-1.08**
PSPTO_4615	*PSYR_1481*	*ppa-2*, inorganic pyrophosphatase	**-1.11**	**-1.20**
PSPTO_0986	*PSYR_0851*	membrane protein	**-1.16**	**-0.61**
PSPTO_4358	*PSYR_4053*	oxidoreductase FAD/FMN-binding protein	**-1.16**	**-0.48**
PSPTO_5006	*PSYR_0517*	*aceF*, pyruvate dehydrogenase complex, E2 component, dihydrolipoamide acetyltransferase	**-1.23**	**-2.41**
PSPTO_5186	*PSYR_0352*	DszA family monooxygenase	**-1.29**	**-1.76**
PSPTO_0396	*PSYR_4782*	NADH:flavin oxidoreductase/NADH oxidase family protein	**-1.38**	**-1.67**
PSPTO_3372	*PSYR_3204*	*nuoI*, NADH dehydrogenase subunit I	**-1.41**	**-2.56**
PSPTO_2289	*PSYR_2087*	aconitate hydratase	**-1.42**	**-0.82**
PSPTO_2203	*PSYR_2013*	*sucD*, succinyl-CoA synthetase subunit alpha	**-1.43**	**-2.31**
PSPTO_5240	*PSYR_0303*	CDP-6-deoxy-delta-3,4-glucoseen reductase	**-1.46**	**-0.81**
PSPTO_2676	*PSYR_2410*	*fdhA-2*, glutathione-independent formaldehyde dehydrogenase	**-1.47**	**-0.27**
PSPTO_3601	*PSYR_3372*	hypothetical protein PSPTO_3601	**-1.49**	**-1.47**
PSPTO_2202	*PSYR_2012*	*sucC*, succinyl-CoA synthetase subunit beta	**-1.50**	**-2.35**
PSPTO_2943	*PSYR_2727*	vdh, vanillin dehydrogenase	**-1.58**	**-0.66**

PSPTO_2680	*PSYR_2413*	*gabD-3*, succinate-semialdehyde dehydrogenase	**-1.80**	**-0.33**
PSPTO_2016	*PSYR_3404*	*acnA*, aconitate hydratase 1	**-1.89**	**-0.85**
PSPTO_2405	*PSYR_2190*	*xenA*, xenobiotic reductase A	**-2.79**	**-1.13**
PSPTO_3466	*PSYR_3247*	*ssuD,* alkanesulfonate monooxygenase	**-3.10**	**-3.33**
**Carbohydrate transport and metabolism**				
PSPTO_2305	*PSYR_2103*	*lsc-2*, levansucrase	**2.70**	**0.36**
PSPTO_1419	*PSYR_1233*	*suhB*, inositol-1-monophosphatase	**2.58**	**1.38**
PSPTO_5289	*PSYR_4847*	*rpiA*, ribose 5-phosphate isomerase	**2.50**	**1.41**
PSPTO_0566	*PSYR_4611*	*rpe*, ribulose-phosphate 3-epimerase	**2.22**	**1.35**
PSPTO_4494	*PSYR_4184*	*tpiA*, triosephosphate isomerase	**1.79**	**1.06**
PSPTO_2688	*PSYR_2421*	major facilitator family transporter	**1.69**	**0.84**
PSPTO_4913	*PSYR_0603*	hypothetical protein PSPTO_4913	**1.52**	**0.25**
PSPTO_0999	*PSYR_0865*	major facilitator family transporter	**1.49**	**0.78**
PSPTO_3278	*PSYR_3113*	hypothetical protein PSPTO_3278	**1.42**	**0.44**
PSPTO_5168	*PSYR_0370*	*fbp*, fructose-1,6-bisphosphatase	**1.41**	**1.81**
PSPTO_3986	*PSYR_1401*	hypothetical protein PSPTO_3986	**1.34**	**0.86**
PSPTO_3793	*PSYR_1685*	*ppnK*, inorganic polyphosphate/ATP-NAD kinase	**1.28**	**0.93**
PSPTO_1245	*PSYR_1065*	polysaccharide deacetylase family protein	**1.18**	**0.51**
PSPTO_1819	*PSYR_3578*	major facilitator superfamily transporter	**1.18**	**2.13**
PSPTO_4383	*PSYR_4078*	AmpG protein	**1.16**	**0.30**
PSPTO_4947	*PSYR_0567*	hypothetical protein PSPTO_4947	**1.11**	**1.35**
PSPTO_3507	*PSYR_3281*	*nagZ*, beta-hexosaminidase	**1.07**	**0.82**
PSPTO_1729	*PSYR_3663*	membrane protein	**1.05**	**0.56**
PSPTO_5327	*PSYR_4885*	*gpmA*, phosphoglycerate mutase	**1.04**	**1.08**
PSPTO_1612	*PSYR_3768*	major facilitator family transporter	**1.03**	**0.78**
PSPTO_0346	*PSYR_0272*	*thrB*, homoserine kinase	**1.02**	**0.70**
PSPTO_2292	*PSYR_2090*	*ppsA*, phosphoenolpyruvate synthase	**1.02**	**1.13**
PSPTO_0385	*PSYR_4792*	*tkt,* transketolase	**1.01**	**1.05**
PSPTO_2044	*PSYR_1854*	transporter	**1.00**	**0.64**
PSPTO_2978	*PSYR_2856*	chitin-binding protein	**0.62**	**1.63**
PSPTO_3035	*PSYR_2908*	phosphoglucomutase, alpha-D-glucose phosphate-specific	**0.52**	**1.39**
PSPTO_2119	*PSYR_1914*	transaldolase	**0.14**	**1.17**
PSPTO_2785	*PSYR_2513*	putrescine ABC transporter periplasmic putrescine-binding protein	**-0.19**	**-1.05**
PSPTO_1554	*PSYR_1363*	enolase	**-0.38**	**-1.83**
PSPTO_5488	*PSYR_5042*	major facilitator family transporter	**-0.41**	**-1.07**
PSPTO_1302	*PSYR_1122*	keto-hydroxyglutarate-aldolase/keto-deoxy-phosphogluconate aldolase	**-0.47**	**-1.65**
PSPTO_3488	*PSYR_3263*	sugar ABC transporter permease	**-0.65**	**-1.05**
PSPTO_0364	*PSYR_4812*	sugar ABC transporter periplasmic sugar-binding protein	**-0.77**	**-2.97**

PSPTO_2826	*PSYR_2565*	hypothetical protein PSPTO_2826	**-0.77**	**-2.27**
PSPTO_1301	*PSYR_1121*	6-phosphogluconolactonase	**-0.84**	**-1.45**
PSPTO_2707	*PSYR_2440*	mannitol ABC transporter, periplasmic mannitol-binding protein	**-0.85**	**-1.72**
PSPTO_2179	*PSYR_1989*	galactonate dehydratase	**-0.91**	**-1.94**
PSPTO_2103	*PSYR_1898*	membrane protein	**-0.91**	**-1.42**
PSPTO_2487	*PSYR_2291*	aldolase II superfamily protein	**-0.94**	**-1.47**
PSPTO_3005	*PSYR_2886*	D-xylose ABC transporter permease	**-0.96**	**-1.51**
PSPTO_0563	*PSYR_4614*	polyamine ABC transporter periplasmic polyamine-binding protein	**-1.01**	**-0.43**
PSPTO_2761	*PSYR_2490*	alpha-amylase family protein	**-1.13**	**-1.08**
PSPTO_3534	*PSYR_3306*	glycosyl hydrolase, family 5 PslG	**-1.14**	**-2.99**
PSPTO_1707	*PSYR_3682*	L-sorbosone dehydrogenase	**-1.15**	**-0.11**
PSPTO_3121	*PSYR_2988*	*zwf-2*, glucose-6-phosphate 1-dehydrogenase	**-1.15**	**-0.04**
PSPTO_2369	*PSYR_2153*	*rbsC-1*, ribose ABC transporter permease	**-1.17**	**-1.33**
PSPTO_3004	*PSYR_2885*	*xylG*, xylose transporter ATP-binding subunit	**-1.23**	**-1.44**
PSPTO_1051	*PSYR_0900*	*dctP*, TRAP dicarboxylate transporter subunit DctP	**-1.23**	**-1.08**
PSPTO_2731	*PSYR_2463*	periplasmic sugar-binding domain protein	**-1.26**	**-0.80**
PSPTO_3737	*PSYR_1740*	ABC transporter permease	**-1.27**	**-1.06**
PSPTO_1294	*PSYR_1115*	glucose ABC transporter permease	**-1.28**	**-1.03**
PSPTO_3002	*PSYR_2883*	*xylA*, xylose isomerase	**-1.30**	**-1.43**
PSPTO_2368	*PSYR_2152*	*rbsA-1*, ribose ABC transporter ATP-binding protein	**-1.31**	**-1.45**
PSPTO_3496	*PSYR_3270*	*iolI,* iolI protein	**-1.39**	**-1.23**
PSPTO_5219	*PSYR_0325*	MFS transporter, phthalate permease family	**-1.40**	**-0.71**
PSPTO_1295	*PSYR_1116*	*gltK*, glucose ABC transporter ATP-binding protein	**-1.40**	**-1.28**
PSPTO_1298	*PSYR_1118*	hypothetical protein PSPTO_1298	**-1.47**	**-1.71**
PSPTO_3003	*PSYR_2884*	*xylF*, D-xylose ABC transporter, periplasmic-D xylose binding protein	**-1.47**	**-1.26**
PSPTO_2180	*PSYR_1990*	MFS transporter, phthalate permease family	**-1.54**	**-2.05**
PSPTO_1293	*PSYR_1114*	glucose ABC transporter permease	**-1.54**	**-0.19**
PSPTO_2762	*PSYR_2491*	*glgB*, 1,4-alpha-glucan-branching protein	**-1.54**	**-1.20**
PSPTO_2765	*PSYR_2494*	hypothetical protein PSPTO_2765	**-1.56**	**-0.80**
PSPTO_2667	*PSYR_2401*	spermidine/putrescine ABC transporter periplasmic spermidine/putrescine-binding protein	**-1.61**	**-1.09**
PSPTO_4296	*PSYR_3999*	metabolite-proton symporter	**-1.68**	**-0.37**
PSPTO_3125	*PSYR_2992*	*glgA*, glycogen synthase	**-1.76**	**-0.85**

PSPTO_5340	*PSYR_4899*	MFS permease-like protein	**-1.78**	**-1.23**
PSPTO_2493	*PSYR_2298*	glycosyl hydrolase family protein	**-1.82**	**-1.03**
PSPTO_4548	*PSYR_4226*	MFS transporter, phthalate permease family	**-1.87**	**-1.05**
PSPTO_3735	*PSYR_1742*	ABC transporter periplasmic substrate-binding protein	**-1.93**	**-1.23**
PSPTO_3740	*PSYR_1737*	ABC transporter ATP-binding protein	**-2.06**	**-0.79**
PSPTO_3739	*PSYR_1738*	ABC transporter ATP-binding protein	**-2.08**	**-0.57**
PSPTO_3126	*PSYR_2993*	alpha-amylase family protein	**-2.11**	**-1.08**
PSPTO_3130	*PSYR_2997*	*glgX*, glycogen operon protein GlgX	**-2.13**	**-1.27**
PSPTO_4306	*PSYR_4010*	pcaT, dicarboxylic acid transport protein	**-2.18**	**-1.78**
PSPTO_2640	*PSYR_2373*	*araH*, L-arabinose transporter permease protein	**-2.18**	**-1.61**
PSPTO_3128	*PSYR_2995*	glycosyl hydrolase family protein	**-2.20**	**-1.45**
PSPTO_3127	*PSYR_2994*	malQ, 4-alpha-glucanotransferase	**-2.50**	**-1.34**
PSPTO_3493	*PSYR_3267*	iolH protein	**-2.80**	**-1.04**
PSPTO_2473	*PSYR_2238*	periplasmic substrate-binding protein	**-3.23**	**-1.12**
PSPTO_2701	*PSYR_2434*	fructokinase	**-3.26**	**-3.93**
PSPTO_2703	*PSYR_2436*	*uxuB*, D-mannonate oxidoreductase	**-3.49**	**-5.27**
PSPTO_1236	*PSYR_1056*	*algL*, alginate lyase	**-3.76**	**-1.06**
PSPTO_2702	*PSYR_2435*	*xylB*, xylulokinase	**-3.78**	**-5.64**
**Amino acid transport and metabolism**				
PSPTO_2206	*PSYR_2015*	*brnQ*, branched-chain amino acid transport system II carrier protein	**2.74**	**1.17**
PSPTO_1421	*PSYR_1235*	*cysE*, serine O-acetyltransferase	**2.58**	**1.65**
PSPTO_1760	*PSYR_3633*	threonine/serine transporter	**2.56**	**0.31**
PSPTO_4938	*PSYR_0576*	ATP phosphoribosyltransferase regulatory subunit	**2.53**	**1.29**
PSPTO_1817	*PSYR_3580*	aromatic amino acid permease	**2.46**	**1.49**
PSPTO_2055	*PSYR_1864*	*speE*, spermidine synthase	**2.28**	**0.84**
PSPTO_0272	*PSYR_0134*	*iscS-1*, cysteine desulfurase	**1.98**	**0.77**
PSPTO_3953	*PSYR_1548*	*dapA*, dihydrodipicolinate synthase	**1.91**	**0.88**
PSPTO_4503	*PSYR_4193*	*dapB*, dihydrodipicolinate reductase	**1.91**	**0.44**
PSPTO_4957	*PSYR_0557*	ACT domain protein/phosphoserine phosphatase SerB	**1.91**	**1.75**
PSPTO_1147	*PSYR_0989*	LysE family transporter	**1.91**	**0.50**
PSPTO_5294	*PSYR_4852*	*serA*, D-3-phosphoglycerate dehydrogenase	**1.91**	**1.36**
PSPTO_4632	*PSYR_4270*	*glyA-2*, serine hydroxymethyltransferase	**1.88**	**0.74**
PSPTO_0169	*PSYR_0025*	*aroE*, shikimate 5-dehydrogenase	**1.79**	**0.86**
PSPTO_3876	*PSYR_1609*	aspartyl aminopeptidase	**1.77**	**1.37**
PSPTO_0316	*PSYR_0246*	*gcvT-1*, glycine cleavage system T protein	**1.73**	**1.70**
PSPTO_5069	*PSYR_0459*	*metF*, 5,10-methylenetetrahydrofolate reductase	**1.71**	**0.74**

PSPTO_4829	*PSYR_4369*	*proA*, gamma-glutamyl phosphate reductase	**1.70**	**1.25**
PSPTO_0793	*PSYR_0696*	*selD*, selenide, water dikinase	**1.69**	**0.56**
PSPTO_1828	*PSYR_3569*	arginine/ornithine ABC transporter, permease protein	**1.65**	**0.69**
PSPTO_1772	*PSYR_3622*	phospho-2-dehydro-3-deoxyheptonate aldolase	**1.63**	**0.80**
PSPTO_1480	*PSYR_1290*	*hom*, homoserine dehydrogenase	**1.60**	**0.36**
PSPTO_2043	*PSYR_1853*	*aroC*, chorismate synthase	**1.59**	**1.21**
PSPTO_5049	*PSYR_0474*	homoserine O-acetyltransferase	**1.56**	**1.38**
PSPTO_0568	*PSYR_4609*	*trpE*, anthranilate synthase, component I	**1.52**	**1.35**
PSPTO_5047	*PSYR_0476*	*proC*, pyrroline-5-carboxylate reductase	**1.52**	**1.34**
PSPTO_1827	*PSYR_3570*	arginine/ornithine ABC transporter, permease protein	**1.51**	**1.16**
PSPTO_1041	*PSYR_0888*	*aroA*, 3-phosphoshikimate 1-carboxyvinyltransferase	**1.49**	**0.27**
PSPTO_5074	*PSYR_0455*	amine oxidase, flavin-containing protein	**1.43**	**0.83**
PSPTO_0125	*PSYR_0065*	*argH*, argininosuccinate lyase	**1.43**	**0.75**
PSPTO_1523	*PSYR_1331*	*dapE*, succinyl-diaminopimelate desuccinylase	**1.43**	**0.83**
PSPTO_5058	*PSYR_0468*	class I and II aminotransferase	**1.43**	**0.87**
PSPTO_3816	*PSYR_1663*	trpF, N-(5'-phosphoribosyl)anthranilate isomerase	**1.43**	**0.62**
PSPTO_3954	*PSYR_1547*	hypothetical protein PSPTO_3954	**1.42**	**0.88**
PSPTO_1779	*PSYR_3615*	class I and II aminotransferase	**1.41**	**0.72**
PSPTO_0225	*PSYR_0182*	*lsyA-2*, diaminopimelate decarboxylase	**1.40**	**1.17**
PSPTO_0598	*PSYR_4575*	*speD*, S-adenosylmethionine decarboxylase	**1.40**	**0.94**
PSPTO_0140	*PSYR_0050*	homoserine/homoserine lactone efflux protein	**1.39**	**0.94**
PSPTO_0774	*PSYR_0678*	beta-alanine--pyruvate aminotransferase	**1.36**	**2.93**
PSPTO_1639	*PSYR_3742*	bifunctional tRNA-methyltransferase/FAD-dependent oxidoreductase	**1.34**	**1.04**
PSPTO_1692	*PSYR_3697*	*cysM*, cysteine synthase B	**1.34**	**0.07**
PSPTO_4164	*PSYR_3901*	*argF*, ornithine carbamoyltransferase	**1.31**	**0.52**
PSPTO_0186	*PSYR_0010*	D,D-heptose 1,7-bisphosphate phosphatase	**1.28**	**0.19**
PSPTO_5126	*PSYR_0409*	*aroB*, 3-dehydroquinate synthase	**1.26**	**0.95**
PSPTO_1528	*PSYR_1336*	tetrahydrodipicolinate succinylase	**1.25**	**1.09**
PSPTO_5335	*PSYR_4894*	hisA, phosphoribosylformimino-5-aminoimidazole carboxamide ribotide isomerase	**1.17**	**1.14**
PSPTO_4572	*PSYR_4247*	pyridoxal-dependent decarboxylase,	**1.06**	**0.48**

		pyridoxal binding domain protein		
PSPTO_0970	*PSYR_0836*	class I and II aminotransferase	**1.06**	**0.35**
PSPTO_1729	*PSYR_3663*	membrane protein	**1.05**	**0.56**
PSPTO_5179	*PSYR_0359*	D-cysteine desulfhydrase	**1.04**	**0.28**
PSPTO_0317	*PSYR_0247*	gcvH-1, glycine cleavage system H protein	**1.03**	**0.61**
PSPTO_5334	*PSYR_4893*	*hisF*, Imidazole glycerol phosphate synthase cyclase subunit	**1.03**	**0.92**
PSPTO_1332	*PSYR_1148*	*ilvE*, branched-chain amino acid aminotransferase	**1.01**	**0.08**
PSPTO_2163	*PSYR_1973*	*aspC*, aspartate aminotransferase	**1.00**	**1.03**
PSPTO_0593	*PSYR_4580*	anthranilate phosphoribosyltransferase	**0.90**	**1.28**
PSPTO_4197	*PSYR_3931*	hypothetical protein PSPTO_4197	**0.71**	**1.01**
PSPTO_5276	*PSYR_4834*	proline-specific permease proY	**0.58**	**1.29**
PSPTO_3027	*PSYR_2900*	phospho-2-dehydro-3-deoxyheptonate aldolase	**0.40**	**2.33**
PSPTO_0592	*PSYR_4581*	anthranilate synthase component II	**0.40**	**1.36**
PSPTO_0787	*PSYR_0691*	gamma-glutamyltranspeptidase	**-0.08**	**-1.41**
PSPTO_3082	*PSYR_2941*	glutamine/glutamate ABC transporter periplasmic glutamine/glutamate-binding protein	**-0.15**	**-1.03**
PSPTO_1531	*PSYR_1340*	class I and II aminotransferase	**-0.22**	**-2.63**
PSPTO_0981	*PSYR_0846*	acetolactate synthase large subunit	**-0.28**	**-1.08**
PSPTO_1747	*PSYR_3645*	chorismate mutase/prephenate dehydratase	**-0.33**	**-1.13**
PSPTO_4437	*PSYR_4132*	histidinol-phosphate aminotransferase	**-0.37**	**-1.08**
PSPTO_0508	*PSYR_4675*	hypothetical protein PSPTO_0508	**-0.39**	**-1.14**
PSPTO_0982	*PSYR_0847*	acetolactate synthase small subunit	**-0.46**	**-1.54**
PSPTO_0983	*PSYR_0848*	ketol-acid reductoisomerase	**-0.49**	**-1.97**
PSPTO_4399	*PSYR_4093*	glutamate N-acetyltransferase/amino-acid acetyltransferase	**-0.55**	**-2.06**
PSPTO_2630	*PSYR_2965*	amino acid ABC transporter ATP-binding protein	**-0.60**	**-3.52**
PSPTO_2810	*PSYR_2538*	peptide ABC transporter permease	**-0.66**	**-1.06**
PSPTO_5121	*PSYR_0412*	glutamate synthase, small subunit	**-0.67**	**-1.61**
PSPTO_2814	*PSYR_2542*	peptide ABC transporter periplasmic peptide-binding protein	**-0.70**	**-1.32**
PSPTO_0565	*PSYR_4612*	polyamine ABC transporter permease	**-0.71**	**-1.10**
PSPTO_2250	*PSYR_2055*	transglutaminase-like domain protein	**-0.71**	**-1.29**
PSPTO_4895	*PSYR_4436*	urease subunit alpha	**-0.73**	**-1.00**
PSPTO_1843	*PSYR_3555*	aspartate kinase	**-0.74**	**-1.56**
PSPTO_3007	*PSYR_2888*	amine oxidase, flavin-containing protein	**-0.77**	**-1.03**
PSPTO_3438	*PSYR_3220*	cysteine desulfurase	**-0.78**	**-1.73**
PSPTO_2664	*PSYR_2398*	spermidine/putrescine ABC transporter	**-0.91**	**-1.98**

		ATP-binding protein		
PSPTO_4534	*PSYR_4211*	peptide ABC transporter substrate-binding protein	**-0.97**	**-1.03**
PSPTO_2811	*PSYR_2539*	peptide ABC transporter permease	**-0.99**	**-1.34**
PSPTO_3327	*PSYR_3158*	sarcosine oxidase	**-0.99**	**-1.25**
PSPTO_2781	*PSYR_2509*	hypothetical protein PSPTO_2781	**-1.01**	**-0.86**
PSPTO_2522	*PSYR_2333*	ABC transporter permease	**-1.05**	**-0.87**
PSPTO_5307	*PSYR_4865*	putrescine ABC transporter periplasmic putrescine-binding protein	**-1.06**	**-0.85**
PSPTO_2960	*PSYR_2745*	pyridoxal-phosphate dependent enzyme family/ornithine cyclodeaminase family protein	**-1.08**	**-1.62**
PSPTO_4561	*PSYR_4238*	*dppA*, dipeptide ABC transporter substrate-binding protein	**-1.09**	**-1.28**
PSPTO_2155	*PSYR_1965*	class V aminotransferase	**-1.11**	**-0.58**
PSPTO_3717	*PSYR_1758*	ABC transporter substrate-binding protein	**-1.12**	**-2.26**
PSPTO_3780	*PSYR_1698*	glutathionylspermidine synthase	**-1.13**	**-1.31**
PSPTO_4112	*PSYR_3849*	high-affinity amino acid ABC transporter, ATP-binding protein	**-1.13**	**-2.99**
PSPTO_5182	*PSYR_0356*	putative amino-acid ABC transporter ATP-binding protein YecC	**-1.13**	**-1.79**
PSPTO_2451	*PSYR_2223*	*soxD-2*, sarcosine oxidase subunit delta	**-1.15**	**-1.17**
PSPTO_3881	*PSYR_1603*	polyamine ABC transporter permease	**-1.16**	**-0.69**
PSPTO_4915	*PSYR_0601*	high affinity branched-chain amino acid ABC transporter ATP-binding protein	**-1.17**	**-1.52**
PSPTO_3084	*PSYR_2943*	glutamine/glutamate ABC transporter, permease protein	**-1.21**	**-0.59**
PSPTO_1631	*PSYR_3749*	hypothetical protein PSPTO_1631	**-1.22**	**-0.21**
PSPTO_5306	*PSYR_4864*	putrescine ABC transporter periplasmic putrescine-binding protein	**-1.26**	**-0.35**
PSPTO_2812	*PSYR_2540*	peptide ABC transporter ATP-binding protein	**-1.26**	**-1.57**
PSPTO_4557	*PSYR_4234*	peptide ABC transporter periplasmic peptide-binding protein	**-1.33**	**-1.85**
PSPTO_2665	*PSYR_2399*	spermidine/putrescine ABC transporter permease	**-1.34**	**-2.34**
PSPTO_4916	*PSYR_0600*	high affinity branched-chain amino acid ABC transporter ATP-binding protein	**-1.36**	**-1.42**
PSPTO_0457	*PSYR_4716*	*soxG-1*, sarcosine oxidase, gamma subunit	**-1.36**	**-1.08**
PSPTO_0459	*PSYR_4714*	*soxD-1*, sarcosine oxidase subunit delta	**-1.37**	**-0.66**
PSPTO_4917	*PSYR_0599*	*braE*, high-affinity branched-chain amino acid ABC transporter, permease protein BraE	**-1.42**	**-1.12**
PSPTO_1622	*PSYR_3758*	glycine betaine-binding protein	**-1.44**	**-0.58**

PSPTO_4919	*PSYR_0597*	high affinity branched-chain amino acid ABC transporter periplasmic amino acid-binding protein	**-1.45**	**-0.38**
PSPTO_4918	*PSYR_0598*	*braD,* high-affinity branched-chain amino acid ABC transporter, permease protein BraD	**-1.45**	**-0.55**
PSPTO_4559	*PSYR_4236*	dipeptide ABC transporter substrate-binding protein	**-1.48**	**-1.35**
PSPTO_0460	*PSYR_4713*	*soxB-1*, sarcosine oxidase, beta subunit	**-1.55**	**-1.01**
PSPTO_1343	*PSYR_1156*	hypothetical protein PSPTO_1343	**-1.58**	**-1.31**
PSPTO_0394	*PSYR_4784*	renal dipeptidase family protein	**-1.59**	**-1.22**
PSPTO_3085	*PSYR_2944*	glutamine/glutamate ABC transporter, permease protein	**-1.63**	**-0.94**
PSPTO_4111	*PSYR_3848*	leucine/isoleucine/valine transporter ATP-binding subunit	**-1.69**	**-3.08**
PSPTO_3495	*PSYR_3269*	iolD protein	**-1.73**	**-1.01**
PSPTO_0817	*PSYR_0721*	oxidoreductase, FAD-binding protein	**-1.75**	**-0.23**
PSPTO_4110	*PSYR_3847*	leucine/isoleucine/valine transporter permease subunit	**-1.96**	**-2.73**
PSPTO_2777	*PSYR_2505*	amino acid ABC transporter permease	**-2.04**	**-0.38**
PSPTO_0461	*PSYR_4712*	*glyA-1*, serine hydroxymethyltransferase	**-2.22**	**-1.05**
PSPTO_2776	*PSYR_2504*	amino acid ABC transporter permease	**-2.23**	**-0.40**
PSPTO_0458	*PSYR_4715*	*soxA-1*, sarcosine oxidase, alpha subunit	**-2.36**	**-1.04**
PSPTO_1258	*PSYR_1075*	amino acid ABC transporter ATP-binding protein	**-2.48**	**-1.71**
PSPTO_1255	*PSYR_1072*	amino acid ABC transporter substrate-binding protein	**-2.54**	**-0.79**
PSPTO_1257	*PSYR_1074*	amino acid ABC transporter permease	**-2.56**	**-2.15**
PSPTO_4558	*PSYR_4235*	dipeptide ABC transporter, periplasmic dipeptide-binding protein	**-2.61**	**-1.75**
PSPTO_4109	*PSYR_3846*	high-affinity branched-chain amino acid ABC transporter permease	**-2.72**	**-2.33**
PSPTO_1256	*PSYR_1073*	amino acid ABC transporter permease	**-2.77**	**-2.30**
**Nucleotide transport and metabolism**				
PSPTO_1700	*PSYR_3689*	*purM*,phosphoribosylformylglycinamidine cyclo-ligase	**2.81**	**1.43**
PSPTO_2028	*PSYR_1836*	*pyrF*, orotidine 5-phosphate decarboxylase	**2.70**	**1.70**
PSPTO_3750	*PSYR_1728*	hypothetical protein PSPTO_3750	**2.63**	**2.26**
PSPTO_4937	*PSYR_0577*	*purA*, adenylosuccinate synthetase	**2.31**	**0.79**
PSPTO_1509	*PSYR_1319*	*adk*, adenylate kinase	**2.19**	**1.60**
PSPTO_1699	*PSYR_3690*	*purN,* phosphoribosylglycinamide formyltransferase	**2.17**	**0.37**
PSPTO_2309	*PSYR_2106*	*pyrD*, dihydroorotate dehydrogenase	**1.96**	**0.15**

PSPTO_3360	*PSYR_3192*	*purB*, adenylosuccinate lyase	**1.85**	**0.29**
PSPTO_1468	*PSYR_1277*	*purT*, phosphoribosylglycinamide formyltransferase 2	**1.74**	**1.14**
PSPTO_1459	*PSYR_1269*	*purL*,phosphoribosylformylglycinamidine synthase	**1.65**	**0.60**
PSPTO_5282	*PSYR_4840*	*thyA*, thymidylate synthase	**1.58**	**1.55**
PSPTO_4314	*PSYR_4018*	purU-3, formyltetrahydrofolate deformylase	**1.58**	**1.01**
PSPTO_0080	*PSYR_0216*	*pyrE*, orotate phosphoribosyltransferase	**1.54**	**1.08**
PSPTO_1130	*PSYR_0969*	*upp*, uracil phosphoribosyltransferase	**1.52**	**0.77**
PSPTO_0075	*PSYR_0211*	*gmk*, guanylate kinase	**1.37**	**0.63**
PSPTO_1449	*PSYR_1261*	*guaB*, inosine 5\'-monophosphate dehydrogenase	**1.35**	**0.44**
PSPTO_3986	*PSYR_1401*	hypothetical protein PSPTO_3986	**1.34**	**0.86**
PSPTO_4502	*PSYR_4192*	*carA*, carbamoyl-phosphate synthase small subunit	**1.23**	**1.16**
PSPTO_5051	*PSYR_0472*	Ham1 protein	**1.21**	**0.15**
PSPTO_0112	*PSYR_0076*	*xpt,* xanthine phosphoribosyltransferase	**1.20**	**0.11**
PSPTO_4795	*PSYR_4337*	AMP nucleosidase	**1.19**	**0.29**
PSPTO_5249	*PSYR_0294*	*ppx*, exopolyphosphatase	**1.18**	**0.72**
PSPTO_4144	*PSYR_3883*	deoxycytidine triphosphate deaminase	**1.10**	**0.17**
PSPTO_1552	*PSYR_1361*	*pyrG*, CTP synthase	**1.05**	**0.16**
PSPTO_1990	*PSYR_3426*	*apt*, adenine phosphoribosyltransferase	**1.03**	**0.81**
PSPTO_4911	*PSYR_4453*	urease accessory protein UreG	**0.80**	**4.33**
PSPTO_0757	*PSYR_0661*	adenosine deaminase	**0.78**	**1.94**
PSPTO_5039	*PSYR_0483*	pyrimidine operon regulatory protein PyrR	**0.40**	**1.48**
PSPTO_0549	*PSYR_4629*	bis(5\'-nucleosyl)-tetraphosphatase, symmetrical	**-0.23**	**-1.13**
PSPTO_1871	*PSYR_3531*	cytosine/purines uracil thiamine allantoin permease	**-0.85**	**-1.62**
PSPTO_4867	*PSYR_4407*	phosphoribosylamine--glycine ligase	**-0.91**	**-1.60**
PSPTO_4975	*PSYR_0545*	cytosine/purines uracil thiamine allantoin permease	**-1.15**	**-0.12**
PSPTO_1671	*PSYR_3717*	nrdA, ribonucleotide-diphosphate reductase subunit alpha	**-2.03**	**-2.02**
PSPTO_1694	*PSYR_3695*	*relA*, GTP pyrophosphokinase	**-4.02**	**-5.22**
**Coenzyme transport and metabolism**				
PSPTO_3959	*PSYR_1542*	*nadA*, quinolinate synthetase	**3.44**	**1.74**
PSPTO_0796	*PSYR_0700*	*ispB*, octylprenyl diphosphate synthase	**2.81**	**1.05**
PSPTO_1708	*PSYR_3681*	*cobO*, cob(I)alamin adenosyltransferasehrpA	**2.69**	**1.11**
PSPTO_1709	*PSYR_3680*	cobyrinic acid a,c-diamide synthase	**2.62**	**0.64**
PSPTO_3733	*PSYR_1743*	*folD-2*, methylenetetrahydrofolate	**2.49**	**1.53**

		dehydrogenase/ methenyltetrahydrofolate cyclohydrolase		
PSPTO_0691	*PSYR_4461*	*ribE,* riboflavin synthase subunit alpha	**2.34**	**1.24**
PSPTO_0170	*PSYR_0024*	*hemF*, coproporphyrinogen III oxidase	**2.30**	**1.22**
PSPTO_0961	*PSYR_0828*	*panB*, 3-methyl-2-oxobutanoate hydroxymethyltransferase	**2.19**	**1.03**
PSPTO_5227	*PSYR_0317*	5-formyltetrahydrofolate cyclo-ligase	**2.18**	**1.52**
PSPTO_5476	*PSYR_5031*	*ubiA*, 4-hydroxybenzoate octaprenyltransferase	**2.10**	**1.37**
PSPTO_4798	*PSYR_4340*	bifunctional hydroxy-methylpyrimidine kinase/ hydroxy-phosphomethylpyrimidine kinase	**2.09**	**1.28**
PSPTO_1710	*PSYR_3679*	nitroreductase family protein	**2.06**	**0.41**
PSPTO_0690	*PSYR_4462*	*ribD*, riboflavin biosynthesis protein RibD	**2.02**	**0.98**
PSPTO_1711	*PSYR_3678*	*cobD*, cobalamin biosynthesis protein CobD	**1.95**	**0.34**
PSPTO_2578	*PSYR_2269*	hypothetical protein PSPTO_2578	**1.93**	**0.50**
PSPTO_1525	*PSYR_1333*	ThiF family protein	**1.88**	**1.62**
PSPTO_4799	*PSYR_4341*	*thiE*, thiamine-phosphate pyrophosphorylase	**1.87**	**1.74**
PSPTO_3142	*PSYR_3009*	nicotinamide mononucleotide transporter PnuC	**1.87**	**0.41**
PSPTO_0692	*PSYR_4460*	*ribBA-1*, bifunctional 3,4-dihydroxy-2-butanone 4-phosphate synthase/GTP cyclohydrolase II-like protein	**1.86**	**0.80**
PSPTO_0960	*PSYR_0827*	*panC*, pantoate--beta-alanine ligase	**1.84**	**0.29**
PSPTO_3969	*PSYR_1418*	radical SAM domain-containing protein	**1.83**	**1.32**
PSPTO_4800	*PSYR_4342*	*hemL*, glutamate-1-semialdehyde-2,1-aminomutase	**1.82**	**1.70**
PSPTO_4878	*PSYR_4418*	*cobL*, precorrin-6Y C5,15-methyltransferase	**1.79**	**1.00**
PSPTO_5118	*PSYR_0414*	*hemE*, uroporphyrinogen decarboxylase	**1.72**	**0.82**
PSPTO_2019	*PSYR_1828*	*pdxB*, erythronate-4-phosphate dehydrogenase	**1.72**	**1.12**
PSPTO_0599	*PSYR_4574*	hypothetical protein PSPTO_0599	**1.71**	**1.49**
PSPTO_5068	*PSYR_0460*	*ahcY*, adenosylhomocysteinase	**1.71**	**1.27**
PSPTO_4876	*PSYR_4416*	*cobH*, precorrin-8X methylmutase	**1.66**	**0.89**
PSPTO_0434	*PSYR_4740*	*thiG*, thiazole biosynthesis protein ThiG	**1.64**	**0.47**
PSPTO_2035	*PSYR_1845*	*folE-2*, GTP cyclohydrolase I	**1.62**	**1.34**
PSPTO_0699	*PSYR_0605*	*ispA*, geranyltranstransferase	**1.61**	**0.70**
PSPTO_4879	*PSYR_4419*	*cbiD*, cobalamin biosynthesis protein CbiD	**1.60**	**0.89**
PSPTO_0360	*PSYR_4816*	*thiI*, thiamin biosynthesis protein ThiI	**1.57**	**1.32**
PSPTO_5475	*PSYR_5030*	chorismate-pyruvate lyase	**1.54**	**1.11**

PSPTO_5150	*PSYR_0389*	*ubiE*, ubiquinone/menaquinone biosynthesis methyltransferase UbiE	**1.52**	**1.96**
PSPTO_0437	*PSYR_4737*	*folA*, dihydrofolate reductase	**1.51**	**1.12**
PSPTO_0417	*PSYR_4758*	pantetheine-phosphate adenylyltransferase	**1.49**	**1.09**
PSPTO_0433	*PSYR_4741*	*thiS*, thiamine biosynthesis protein ThiS	**1.49**	**0.29**
PSPTO_4877	*PSYR_4417*	cobalamin biosynthesis protein CobG	**1.44**	**0.90**
PSPTO_5075	*PSYR_0454*	*bioA*, adenosylmethionine--8-amino-7-oxononanoate transaminase	**1.40**	**0.95**
PSPTO_0696	*PSYR_4456*	*ribA*, GTP cyclohydrolase II	**1.37**	**0.74**
PSPTO_4880	*PSYR_4420*	cobalt-precorrin-6x reductase	**1.35**	**0.28**
PSPTO_0805	*PSYR_0709*	*ribF*, riboflavin biosynthesis protein RibF	**1.34**	**1.26**
PSPTO_0962	*PSYR_0829*	*folK-2*, 2-amino-4-hydroxy-6-hydroxymethyldihydropteridine pyrophosphokinase	**1.27**	**0.92**
PSPTO_4875	*PSYR_4415*	*cobI*, precorrin-2 C(20)-methyltransferase	**1.26**	**0.88**
PSPTO_0948	*PSYR_0815*	*nadC*, nicotinate-nucleotide pyrophosphorylase	**1.25**	**1.25**
PSPTO_4922	*PSYR_0594*	*nadE*, NH(3)-dependent NAD(+) synthetase	**1.23**	**0.88**
PSPTO_0698	*PSYR_0604*	*dxs,* deoxyxylulose-5-phosphate synthase	**1.20**	**1.51**
PSPTO_0085	*PSYR_0221*	*coaBC*, phosphopantothenoylcysteine decarboxylase/phosphopantothenate--cysteine ligase	**1.20**	**0.84**
PSPTO_4818	*PSYR_4358*	*lipA*, lipoic acid synthetase	**1.18**	**0.78**
PSPTO_4828	*PSYR_4368*	*nadD*, nicotinate (nicotinamide) nucleotide adenylyltransferase	**1.11**	**1.08**
PSPTO_5035	*PSYR_0487*	*gshB*, glutathione synthetase	**1.10**	**1.17**
PSPTO_0128	*PSYR_0062*	*hemC*, porphobilinogen deaminase	**1.08**	**0.96**
PSPTO_0542	*PSYR_4636*	*folB-1*, dihydroneopterin aldolase	**1.06**	**0.51**
PSPTO_4225	*PSYR_3959*	*nadB*, L-aspartate oxidase	**1.05**	**0.34**
PSPTO_4921	*PSYR_0595*	*pncB*, nicotinate phosphoribosyltransferase	**1.05**	**1.25**
PSPTO_1108	*PSYR_0948*	*hemA*, glutamyl-tRNA reductase	**1.03**	**1.04**
PSPTO_2352	*PSYR_2136*	*moaB*, molybdenum cofactor biosynthesis protein B	**1.02**	**0.85**
PSPTO_1128	*PSYR_0967*	*hemH,* ferrochelatase	**0.98**	**1.49**
PSPTO_4983	*PSYR_0537*	lipopolysaccharide biosynthesis protein RfaE	**0.96**	**1.00**
PSPTO_2351	*PSYR_2135*	molybdopterin-guanine dinucleotide biosynthesis protein MobA	**0.91**	**1.13**
PSPTO_5251	*PSYR_0292*	delta-aminolevulinic acid dehydratase	**0.76**	**1.21**
PSPTO_1183	*PSYR_1021*	dihydromonapterin reductase	**0.73**	**1.10**
PSPTO_0592	*PSYR_4581*	anthranilate synthase component II	**0.40**	**1.36**
PSPTO_2300	*PSYR_2098*	uroporphyrin-III C-methyltransferase	**0.30**	**1.22**

PSPTO_1839	*PSYR_3558*	6,7-dimethyl-8-ribityllumazine synthase	**0.04**	**1.45**
PSPTO_0497	*PSYR_4684*	biotin synthesis protein BioC	**-0.13**	**-2.20**
PSPTO_0498	*PSYR_4683*	dethiobiotin synthetase	**-0.14**	**-2.39**
PSPTO_2786	*PSYR_2514*	ABC transporter ATP-binding protein	**-0.20**	**-1.20**
PSPTO_0495	*PSYR_4686*	8-amino-7-oxononanoate synthase	**-0.35**	**-1.24**
PSPTO_2645	*PSYR_2377*	hypothetical protein PSPTO_2645	**-0.59**	**-1.03**
PSPTO_0552	*PSYR_4626*	pyridoxal phosphate biosynthetic protein PdxA	**-0.83**	**-2.56**
PSPTO_1871	*PSYR_3531*	cytosine/purines uracil thiamine allantoin permease	**-0.85**	**-1.62**
PSPTO_3147	*PSYR_3013*	hypothetical protein PSPTO_3147	**-1.00**	**-1.19**
PSPTO_1738	*PSYR_3654*	hypothetical protein PSPTO_1738	**-1.04**	**-1.01**
PSPTO_3149	*PSYR_3015*	cobaltochelatase subunit CobN	**-1.07**	**-1.18**
PSPTO_3148	*PSYR_3014*	magnesium chelatase, subunit ChII	**-1.10**	**-1.28**
PSPTO_0511	*PSYR_4672*	*pqqC*, coenzyme PQQ synthesis protein C	**-1.13**	**-0.61**
PSPTO_5240	*PSYR_0303*	CDP-6-deoxy-delta-3,4-glucoseen reductase	**-1.46**	**-0.81**
PSPTO_5313	*PSYR_4873*	molybdenum-pterin binding domain-containing protein	**-2.12**	**-4.74**
PSPTO_5086	*PSYR_0443*	*mdcB*, 2-(5'-triphosphoribosyl)-3'-dephosphocoenzyme-A synthase	**-2.41**	**-0.30**
PSPTO_4976	*PSYR_0544*	*thiC,* thiamin biosynthesis protein ThiC	**-3.11**	**-2.44**
**Lipid transport and metabolism**				
PSPTO_1105	*PSYR_0945*	*ispE*, 4-diphosphocytidyl-2-C-methyl-D-erythritol kinase	**2.47**	**1.06**
PSPTO_1825	*PSYR_3572*	*acs*, acetyl-CoA synthetase	**2.46**	**5.01**
PSPTO_0305	*PSYR_0087*	fatty acid desaturase	**2.33**	**2.06**
PSPTO_1560	*PSYR_1369*	*ispF*, 2-C-methyl-D-erythritol 2,4-cyclodiphosphate synthase	**2.12**	**1.52**
PSPTO_0187	*PSYR_0009*	hdtS protein	**2.01**	**0.67**
PSPTO_4831	*PSYR_4371*	PAP2 superfamily protein/DedA family protein	**1.86**	**0.80**
PSPTO_5092	*PSYR_0438*	acyltransferase family protein	**1.62**	**1.03**
PSPTO_5094	*PSYR_0436*	acyl carrier protein	**1.49**	**1.83**
PSPTO_5093	*PSYR_0437*	acyl carrier protein	**1.43**	**1.61**
PSPTO_4660	*PSYR_4293*	*tesB*, acyl-CoA thioesterase II	**1.42**	**1.00**
PSPTO_3455	*PSYR_3237*	3-oxoacid CoA-transferase, subunit A family	**1.31**	**4.41**
PSPTO_3815	*PSYR_1664*	*accD*, acetyl-CoA carboxylase subunit beta	**1.29**	**0.69**
PSPTO_3136	*PSYR_3003*	azoreductase	**1.28**	**1.31**
PSPTO_2210	*PSYR_2019*	*fabB*, 3-oxoacyl-(acyl carrier protein) synthase I	**1.25**	**1.49**
PSPTO_3899	*PSYR_1586*	acyltransferase family protein	**1.19**	**0.43**

PSPTO_4097	*PSYR_3834*	*fadD-1*, long-chain-fatty-acid--CoA ligase	**1.19**	**0.42**
PSPTO_3831	*PSYR_1648*	*acpP*, acyl carrier protein	**1.17**	**0.49**
PSPTO_4513	*PSYR_4201*	hypothetical protein PSPTO_4513	**1.16**	**0.31**
PSPTO_3022	*PSYR_2895*	*pgsA*, CDP-diacylglycerol--glycerol-3-phosphate 3-phosphatidyltransferase	**1.14**	**0.73**
PSPTO_3463	*PSYR_3244*	*pssA-2*, CDP-diacylglycerol--serine O-phosphatidyltransferase	**1.10**	**0.92**
PSPTO_2211	*PSYR_2020*	*fabA*, 3-hydroxydecanoyl-(acyl carrier protein) dehydratase	**1.05**	**1.36**
PSPTO_4094	*PSYR_3830*	3-oxoacyl-(acyl carrier protein) synthase III	**1.01**	**0.88**
PSPTO_3833	*PSYR_1646*	fabD, malonyl CoA-acyl carrier protein transacylase	**1.01**	**0.11**
PSPTO_0095	*PSYR_0229*	phospholipase D	**0.93**	**1.62**
PSPTO_0452	*PSYR_4724*	putative acyltransferase	**0.93**	**1.20**
PSPTO_3456	*PSYR_3238*	3-oxoacid CoA-transferase, subunit B family	**0.84**	**3.96**
PSPTO_2736	*PSYR_2467*	biotin carboxylase	**0.79**	**1.38**
PSPTO_2097	*PSYR_1892*	3-ketoacyl-(acyl-carrier-protein) reductase	**0.62**	**1.10**
PSPTO_1540	*PSYR_1349*	1-deoxy-D-xylulose 5-phosphate reductoisomerase	**-0.29**	**-1.46**
PSPTO_1720	*PSYR_3669*	outer membrane protein	**-0.74**	**-1.20**
PSPTO_5108	*PSYR_0424*	hypothetical protein PSPTO_5108	**-0.76**	**-1.40**
PSPTO_5107	*PSYR_0425*	3-oxoacyl-(acyl carrier protein) synthase I	**-0.80**	**-1.56**
PSPTO_3516	*PSYR_3289*	3-ketoacyl-CoA thiolase	**-0.87**	**-1.11**
PSPTO_3163	*PSYR_3030*	enoyl-CoA hydratase/isomerase family protein	**-1.03**	**-0.39**
PSPTO_2940	*PSYR_2725*	thiolase family protein	**-1.08**	**-1.11**
PSPTO_5185	*PSYR_0353*	acyl-CoA dehydrogenase family protein	**-1.12**	**-1.65**
PSPTO_1790	*PSYR_3604*	acyl-CoA dehydrogenase family protein	**-1.13**	**-2.13**
PSPTO_0569	*PSYR_4606*	autotransporting lipase, GDSL family	**-1.43**	**-1.23**
PSPTO_5081	*PSYR_0448*	malonyl CoA-acyl carrier protein transacylase	**-1.83**	**-0.57**
PSPTO_5184	*PSYR_0354*	acyl-CoA dehydrogenase family protein	**-2.49**	**-3.02**
PSPTO_5084	*PSYR_0445*	malonate decarboxylase subunit beta	**-2.75**	**-0.18**
**Inorganic ion transport and metabolism**				
PSPTO_0752	*PSYR_0656*	copZ protein	**3.41**	**4.38**
PSPTO_4160	*PSYR_3897*	*bfr,* bacterioferritin	**3.96**	**2.81**
PSPTO_0750	*PSYR_0654*	copper-translocating P-type ATPase	**2.75**	**3.45**
PSPTO_0753	*PSYR_0657*	Bcr/CflA family multidrug resistance transporter	**2.45**	**1.32**
PSPTO_0998	*PSYR_0863*	alkylphosphonate utilization operon protein PhnA	**2.41**	**2.79**
PSPTO_5262	*PSYR_0281*	*metN-2*, DL-methionine transporter ATP-	**2.32**	**1.62**

		binding subunit		
PSPTO_5261	*PSYR_0282*	*metI-2*, D-methionine ABC transporter permease	**2.19**	**1.35**
PSPTO_0791	*PSYR_0695*	phosphonate ABC transporter periplasmic phosphonate-binding protein	**2.18**	**1.01**
PSPTO_5268	*PSYR_0275*	*znuA*, zinc ABC transporter periplasmic zinc-binding protein	**2.12**	**1.94**
PSPTO_5266	*PSYR_0277*	*znuC*, zinc ABC transporter ATP-binding protein	**1.95**	**0.90**
PSPTO_1801	*PSYR_3593*	*trkH*, potassium uptake protein TrkH	**1.92**	**0.62**
PSPTO_3234	*PSYR_3086*	sulfate permease family protein	**1.91**	**0.46**
PSPTO_2018	*PSYR_1827*	sulfur transfer protein SirA	**1.91**	**0.80**
PSPTO_4979	*PSYR_0541*	multidrug resistance protein	**1.87**	**0.96**
PSPTO_5326	*PSYR_4884*	rhodanese-like domain-containing protein	**1.78**	**1.79**
PSPTO_0763	*PSYR_0667*	*fecB*, iron(III) dicitrate transport system, periplasmic iron-binding protein FecB	**1.69**	**1.23**
PSPTO_0790	*PSYR_0694*	phosphonate ABC transporter, ATP-binding protein	**1.58**	**0.33**
PSPTO_4569	*PSYR_4243*	iron-uptake factor	**1.56**	**1.15**
PSPTO_2355	*PSYR_2139*	glutathione-regulated potassium-efflux system protein	**1.52**	**1.00**
PSPTO_5479	*PSYR_5034*	transporter	**1.50**	**1.84**
PSPTO_3593	*PSYR_3363*	sulfate permease family protein	**1.48**	**0.22**
PSPTO_0314	*PSYR_0244*	iron ABC transporter periplasmic iron-binding protein	**1.41**	**1.79**
PSPTO_0762	*PSYR_0666*	*fecC*, iron-dicitrate transporter permease subunit	**1.39**	**0.97**
PSPTO_0227	*PSYR_0180*	*cyaY*, cyaY protein	**1.36**	**0.43**
PSPTO_5023	*PSYR_0502*	sodium/hydrogen exchanger family protein	**1.33**	**0.67**
PSPTO_5343	*PSYR_4902*	hypothetical protein PSPTO_5343	**1.27**	**0.51**
PSPTO_1846	*PSYR_3552*	*mgtE*, magnesium transporter	**1.22**	**0.98**
PSPTO_2634	*PSYR_2367*	cation ABC transporter permease	**1.14**	**0.30**
PSPTO_1529	*PSYR_1337*	arsC family protein	**1.13**	**1.14**
PSPTO_5265	*PSYR_0278*	*znuB*, zinc ABC transporter permease	**1.08**	**0.52**
PSPTO_3256	*PSYR_3094*	iron ABC transporter, periplasmic iron-binding protein	**1.06**	**1.30**
PSPTO_5260	*PSYR_0283*	*metQ-2*, D-methionine-binding lipoprotein MetQ	**1.06**	**1.26**
PSPTO_1687	*PSYR_3702*	*arsC*, arsenate reductase	**1.05**	**1.12**
PSPTO_3993	*PSYR_1394*	*kup*, potassium uptake protein	**1.04**	**0.28**
PSPTO_4312	*PSYR_4016*	phosphate transporter family protein	**1.03**	**1.30**
PSPTO_0315	*PSYR_0245*	iron ABC transporter permease	**0.95**	**1.06**
PSPTO_0218	*PSYR_0189*	ammonium transporter	**0.93**	**2.70**

PSPTO_2280	*PSYR_2078*	phosphoadenosine phosphosulfate reductase	**0.90**	**1.23**
PSPTO_5269	*PSYR_4827*	choline transporter	**0.66**	**1.37**
PSPTO_3340	*PSYR_3170*	DsrH family protein	**0.62**	**1.02**
PSPTO_3341	*PSYR_3171*	hypothetical protein PSPTO_3341	**0.39**	**1.06**
PSPTO_5191	*PSYR_0346*	AcrB/AcrD/AcrF family protein	**-0.12**	**-1.31**
PSPTO_5485	*PSYR_5039*	phosphate ABC transporter permease	**-0.43**	**-1.30**
PSPTO_0550	*PSYR_4628*	apaG protein	**-0.43**	**-1.18**
PSPTO_2798	*PSYR_2525*	transporter, partial	**-0.46**	**-1.08**
PSPTO_5189	*PSYR_0349*	D-methionine ABC transporter permease	**-0.53**	**-1.55**
PSPTO_0250	*PSYR_0156*	peptide ABC transporter permease	**-0.64**	**-1.05**
PSPTO_2810	*PSYR_2538*	peptide ABC transporter permease	**-0.66**	**-1.06**
PSPTO_2809	*PSYR_2537*	hypothetical protein PSPTO_2809	**-0.68**	**-1.15**
PSPTO_0788	*PSYR_0692*	phosphonate ABC transporter, permease protein	**-0.71**	**-2.30**
PSPTO_1791	*PSYR_3603*	ABC transporter, periplasmic substrate-binding protein, aliphatic sulfonates family	**-0.85**	**-1.66**
PSPTO_4530	*PSYR_4208*	catalase/peroxidase HPI	**-0.88**	**-1.57**
PSPTO_5188	*PSYR_0350*	D-methionine ABC transporter ATP-binding protein	**-0.91**	**-1.90**
PSPTO_3718	*PSYR_1757*	ABC transporter permease	**-0.92**	**-2.70**
PSPTO_2139	*PSYR_1949*	cation ABC transporter permease	**-0.94**	**-1.47**
PSPTO_2811	*PSYR_2539*	peptide ABC transporter permease	**-0.99**	**-1.34**
PSPTO_5256	*PSYR_0287*	sulfate transporter family protein	**-1.01**	**-0.73**
PSPTO_2141	*PSYR_1951*	cation ABC transporter substrate-binding protein	**-1.01**	**-1.24**
PSPTO_4171	*PSYR_3908*	amino acid ABC transporter substrate-binding protein	**-1.01**	**-0.19**
PSPTO_0311	*PSYR_0081*	sulfate ABC transporter, ATP-binding protein CysA	**-1.03**	**-1.43**
PSPTO_0564	*PSYR_4613*	polyamine ABC transporter permease	**-1.03**	**-1.08**
PSPTO_3599	*PSYR_3370*	hypothetical protein PSPTO_3599	**-1.05**	**-0.77**
PSPTO_3031	*PSYR_2904*	peptide ABC transporter permease	**-1.05**	**-1.22**
PSPTO_0887	*PSYR_0760*	sugar ABC transporter permease	**-1.06**	**-0.53**
PSPTO_0310	*PSYR_0082*	sulfate ABC transporter permease CysW	**-1.08**	**-1.41**
PSPTO_5187	*PSYR_0351*	*metQ-1*, D-methionine-binding lipoprotein MetQ	**-1.11**	**-0.75**
PSPTO_2556	*PSYR_2248*	*phnD*, phosphonates ABC transporter periplasmic phosphonates-binding protein	**-1.16**	**-0.45**
PSPTO_0309	*PSYR_0083*	*cysT*, sulfate ABC transporter permease CysT	**-1.20**	**-1.52**
PSPTO_2523	*PSYR_2334*	ABC transporter permease	**-1.25**	**-0.98**
PSPTO_2812	*PSYR_2540*	peptide ABC transporter ATP-binding	**-1.26**	**-1.57**

		protein		
PSPTO_2140	*PSYR_1950*	cation ABC transporter ATP-binding protein	**-1.33**	**-1.50**
PSPTO_3090	*PSYR_2960*	nickel ABC transporter, permease protein	**-1.36**	**-1.37**
PSPTO_4363	*PSYR_4059*	*sodB*, superoxide dismutase, Fe	**-1.38**	**-0.09**
PSPTO_1792	*PSYR_3602*	rhodanese-like domain-containing protein	**-1.41**	**-2.29**
PSPTO_2517	*PSYR_2328*	sodium/hydrogen exchanger family protein	**-1.45**	**-0.36**
PSPTO_3719	*PSYR_1756*	ABC transporter permease	**-1.49**	**-3.58**
PSPTO_5216	*PSYR_0328*	lipoprotein, NLPA family	**-1.49**	**-1.15**
PSPTO_2562	*PSYR_2254*	*phnJ*, phosphonate metabolism protein PhnJ	**-1.52**	**-0.52**
PSPTO_2518	*PSYR_2329*	extracellular solute-binding domain protein	**-1.54**	**-1.26**
PSPTO_2564	*PSYR_2256*	phosphonate ABC transporter ATPase	**-1.58**	**-0.71**
PSPTO_5079	*PSYR_0450*	*madM*, malonate transporter subunit MadM	**-1.58**	**-0.46**
PSPTO_5196	*PSYR_0339*	ABC transporter permease	**-1.58**	**-1.99**
PSPTO_2563	*PSYR_2255*	phosphonate C-P lyase system protein PhnK	**-1.59**	**-0.91**
PSPTO_2666	*PSYR_2400*	spermidine/putrescine ABC transporter permease	**-1.62**	**-2.35**
PSPTO_2138	*PSYR_1948*	ABC transporter periplasmic substrate-binding protein	**-1.72**	**-1.49**
PSPTO_2152	*PSYR_1962*	TonB-dependent siderophore receptor	**-1.82**	**-1.06**
PSPTO_0308	*PSYR_0084*	*sbp*, sulfate-binding protein	**-1.83**	**-2.15**
PSPTO_3878	*PSYR_1606*	ABC transporter substrate-binding protein	**-1.89**	**-1.09**
PSPTO_5197	*PSYR_0338*	ABC transporter ATP-binding protein	**-1.90**	**-2.86**
PSPTO_5195	*PSYR_0340*	ABC transporter substrate-binding protein	**-1.92**	**-2.90**
PSPTO_2706	*PSYR_2439*	mannitol ABC transporter permease	**-2.06**	**-3.81**
PSPTO_5080	*PSYR_0449*	*madL*, malonate transporter subunit MadL	**-2.20**	**-0.80**
PSPTO_5316	*PSYR_4876*	sulfonate ABC transporter periplasmic sulfonate-binding protein	**-2.23**	**-3.55**
PSPTO_2639	*PSYR_2372*	L-arabinose transporter ATP-binding protein	**-2.36**	**-1.30**
PSPTO_5314	*PSYR_4874*	aliphatic sulfonates transport ATP-binding subunit	**-2.45**	**-4.04**
PSPTO_5315	*PSYR_4875*	*ssuC*, aliphatic sulfonates ABC transporter permease	**-2.46**	**-3.95**
PSPTO_2705	*PSYR_2438*	mannitol ABC transporter permease	**-2.67**	**-4.23**
PSPTO_2691	*PSYR_2424*	membrane protein, TerC family	**-2.88**	**-0.11**
**Secondary metabolites biosynthesis, transport and catabolism**				
PSPTO_1735	*PSYR_3657*	hypothetical protein PSPTO_1735	**1.89**	**1.00**
PSPTO_3389	*PSYR_4583*	lysozyme	**1.60**	**1.65**

PSPTO_1456	*PSYR_1266*	cumA, multicopper oxidase	**1.58**	**0.40**
PSPTO_5190	*PSYR_0348*	hypothetical protein PSPTO_5190	**1.51**	**0.39**
PSPTO_5094	*PSYR_0436*	acyl carrier protein	**1.49**	**1.83**
PSPTO_2039	*PSYR_1849*	isochorismatase family protein	**1.49**	**1.73**
PSPTO_5093	*PSYR_0437*	acyl carrier protein	**1.43**	**1.61**
PSPTO_4177	*PSYR_3914*	2-hydroxychromene-2-carboxylate isomerase	**1.29**	**0.27**
PSPTO_4444	*PSYR_4138*	toluene tolerance protein	**1.26**	**1.31**
PSPTO_4212	*PSYR_3946*	methyltransferase	**1.23**	**0.66**
PSPTO_3138	*PSYR_3005*	3-oxoadipate enol-lactone hydrolase	**1.19**	**0.72**
PSPTO_2338	*PSYR_2122*	pcaH, protocatechuate 3,4-dioxygenase subunit beta	**1.17**	**0.49**
PSPTO_5292	*PSYR_4850*	fumarylacetoacetate hydrolase family protein	**1.14**	**0.56**
PSPTO_3713	*PSYR_1762*	hypothetical protein PSPTO_3713	**1.02**	**1.03**
PSPTO_4445	*PSYR_4139*	mce-like protein	**1.01**	**1.44**
PSPTO_4286	*PSYR_3988*	hypothetical protein PSPTO_4286	**0.96**	**1.31**
PSPTO_4447	*PSYR_4141*	toluene tolerance ABC transporter, ATP-binding protein	**0.90**	**1.12**
PSPTO_2097	*PSYR_1892*	3-ketoacyl-(acyl-carrier-protein) reductase	**0.62**	**1.10**
PSPTO_2267	*PSYR_2067*	ABC transporter ATP-binding protein	**0.48**	**1.25**
PSPTO_4446	*PSYR_4140*	membrane protein	**0.36**	**1.12**
PSPTO_5253	*PSYR_0290*	enhancing lycopene biosynthesis protein 2	**0.25**	**1.53**
PSPTO_2266	*PSYR_2066*	permease	**0.10**	**1.40**
PSPTO_2806	*PSYR_2534*	dienelactone hydrolase family protein	**-0.43**	**-1.27**
PSPTO_2961	*PSYR_2746*	L-lysine 6-monooxygenase	**-0.84**	**-1.58**
PSPTO_2135	*PSYR_1945*	*pvsA*, pyoverdine chromophore precursor synthetase	**-1.15**	**-0.22**
PSPTO_2941	*PSYR_2726*	*fcs*, feruloyl-CoA synthase	**-1.20**	**-1.07**
PSPTO_2148	*PSYR_1958*	pyoverdine sidechain peptide synthetase II, D-Asp-L-Thr component	**-1.22**	**-0.98**
PSPTO_2134	*PSYR_1944*	pyoverdine synthetase, thioesterase component	**-1.25**	**-0.49**
PSPTO_2209	*PSYR_2018*	dienelactone hydrolase family protein	**-1.55**	**-0.30**
PSPTO_5198	*PSYR_0337*	dioxygenase, TauD/TfdA family	**-1.59**	**-2.82**
PSPTO_4699	*PSYR_3722*	non-ribosomal peptide synthetase, terminal component	**-1.77**	**-0.57**
PSPTO_5082	*PSYR_0447*	phosphoribosyl-dephospho-CoA transferase	**-1.98**	**-0.53**
PSPTO_4710	*PSYR_2610*	*cmaB*, coronamic acid synthetase CmaB	**-2.10**	**-0.82**
**Funcation unknow**				
PSPTO_5228	*PSYR_0316*	hypothetical protein PSPTO_5228	**3.23**	**0.75**
PSPTO_1774	*PSYR_3620*	hypothetical protein PSPTO_1774	**2.98**	**1.64**
PSPTO_5015	*PSYR_0508*	Lyase	**2.79**	**3.10**

PSPTO_0513	*PSYR_4670*	*pqqA*, coenzyme PQQ synthesis protein A	**2.74**	**0.77**
PSPTO_3361	*PSYR_3193*	hypothetical protein PSPTO_3361	**2.71**	**0.80**
PSPTO_3908	*PSYR_1577*	hypothetical protein PSPTO_3908	**2.67**	**1.67**
PSPTO_2687	*PSYR_2420*	hypothetical protein PSPTO_2687	**2.65**	**1.40**
PSPTO_5466	*PSYR_5020*	hypothetical protein PSPTO_5466	**2.64**	**2.73**
PSPTO_1104	*PSYR_0944*	prsA, ribose-phosphate pyrophosphokinase	**2.57**	**1.31**
PSPTO_0326	*PSYR_0256*	hypothetical protein PSPTO_0326	**2.50**	**0.11**
PSPTO_0724	*PSYR_0626*	DedA family protein	**2.46**	**1.44**
PSPTO_5264	*PSYR_0279*	lipoprotein	**2.44**	**1.41**
PSPTO_3175	*PSYR_3040*	hypothetical protein PSPTO_3175	**2.44**	**0.73**
PSPTO_1623	*PSYR_3757*	hypothetical protein PSPTO_1623	**2.40**	**6.43**
PSPTO_1683	*PSYR_3706*	peptidase, SprT family	**2.40**	**0.69**
PSPTO_4030	*PSYR_1381*	hypothetical protein PSPTO_4030	**2.38**	**2.22**
PSPTO_3751	*PSYR_1727*	hypothetical protein PSPTO_3751	**2.38**	**0.89**
PSPTO_3123	*PSYR_2990*	hypothetical protein PSPTO_3123	**2.35**	**0.79**
PSPTO_3909	*PSYR_1576*	hypothetical protein PSPTO_3909	**2.35**	**1.45**
PSPTO_1734	*PSYR_3658*	putative rhodanese-related sulfurtransferase	**2.34**	**1.67**
PSPTO_4029	*PSYR_1382*	hypothetical protein PSPTO_4029	**2.34**	**1.65**
PSPTO_0367	*PSYR_4809*	hypothetical protein PSPTO_0367	**2.33**	**0.61**
PSPTO_3173	*PSYR_3038*	hypothetical protein PSPTO_3173	**2.33**	**1.68**
PSPTO_1690	*PSYR_3699*	hypothetical protein PSPTO_1690	**2.32**	**2.08**
PSPTO_0799	*PSYR_0703*	GTP-binding protein, GTP1/Obg family	**2.24**	**1.41**
PSPTO_4211	*PSYR_3945*	lipoprotein	**2.23**	**0.62**
PSPTO_4227	*PSYR_3961*	hypothetical protein PSPTO_4227	**2.19**	**0.44**
PSPTO_2017	*PSYR_3403*	hypothetical protein PSPTO_2017	**2.16**	**1.20**
PSPTO_0412	*PSYR_4764*	hypothetical protein PSPTO_0412	**2.15**	**1.27**
PSPTO_1138	*PSYR_0978*	lipoprotein	**2.13**	**0.88**
PSPTO_4816	*PSYR_4356*	hypothetical protein PSPTO_4816	**2.13**	**1.44**
PSPTO_4481	*PSYR_4172*	hypothetical protein PSPTO_4481	**2.11**	**1.04**
PSPTO_2315	*PSYR_2112*	hypothetical protein PSPTO_2315	**2.05**	**1.15**
PSPTO_4484	*PSYR_4175*	hypothetical protein PSPTO_4484	**2.05**	**3.62**
PSPTO_0071	*PSYR_0207*	lipoprotein	**2.04**	**2.91**
PSPTO_4113	*PSYR_3850*	lipoprotein	**2.03**	**0.74**
PSPTO_3858	*PSYR_1627*	hypothetical protein PSPTO_3858	**2.03**	**0.93**
PSPTO_3910	*PSYR_1575*	hypothetical protein PSPTO_3910	**2.00**	**1.39**
PSPTO_4114	*PSYR_3851*	hypothetical protein PSPTO_4114	**1.98**	**1.64**
PSPTO_3362	*PSYR_3194*	GNAT family acetyltransferase	**1.97**	**0.43**
PSPTO_1642	*PSYR_3740*	hypothetical protein PSPTO_1642	**1.95**	**1.42**
PSPTO_1594	*PSYR_3784*	hypothetical protein PSPTO_1594	**1.93**	**1.48**
PSPTO_1272	*PSYR_1092*	membrane protein	**1.93**	**1.74**
PSPTO_1624	*PSYR_3756*	sodium:solute symporter family protein	**1.92**	**5.86**
PSPTO_2188	*PSYR_1998*	lipoprotein	**1.91**	**1.82**

PSPTO_4226	*PSYR_3960*	hypothetical protein PSPTO_4226	**1.88**	**0.23**
PSPTO_0404	*PSYR_4772*	hypothetical protein PSPTO_0404	**1.87**	**1.94**
PSPTO_3836	*PSYR_1643*	hypothetical protein PSPTO_3836	**1.85**	**1.05**
PSPTO_4668	*PSYR_4303*	membrane protein	**1.85**	**1.07**
PSPTO_1593	*PSYR_3786*	hypothetical protein PSPTO_1593	**1.85**	**2.36**
PSPTO_2264	*PSYR_2064*	hypothetical protein PSPTO_2264	**1.82**	**1.36**
PSPTO_4107	*PSYR_3844*	hypothetical protein PSPTO_4107	**1.82**	**1.26**
PSPTO_0138	*PSYR_0052*	ABC transporter ATP-binding protein	**1.82**	**1.68**
PSPTO_0567	*PSYR_4610*	*gph-1*, phosphoglycolate phosphatase	**1.81**	**1.27**
PSPTO_0078	*PSYR_0214*	hypothetical protein PSPTO_0078	**1.80**	**1.95**
PSPTO_0241	*PSYR_4644*	hypothetical protein PSPTO_0241	**1.78**	**3.04**
PSPTO_0798	*PSYR_0702*	*rpmA*, 50S ribosomal protein L27	**1.78**	**1.79**
PSPTO_3521	*PSYR_3294*	ABC transporter ATP-binding protein	**1.74**	**1.53**
PSPTO_3333	*PSYR_3164*	membrane protein	**1.74**	**1.25**
PSPTO_4931	*PSYR_0583*	membrane protein	**1.72**	**0.99**
PSPTO_0991	*PSYR_0856*	hypothetical protein PSPTO_0991	**1.71**	**1.01**
PSPTO_0683	*PSYR_4469*	hypothetical protein PSPTO_0683	**1.71**	**0.87**
PSPTO_3921	*PSYR_1564*	hypothetical protein PSPTO_3921	**1.71**	**1.32**
PSPTO_3812	*PSYR_1667*	cvpA family protein	**1.70**	**0.65**
PSPTO_3907	*PSYR_1578*	hypothetical protein PSPTO_3907	**1.70**	**1.46**
PSPTO_3769	*PSYR_1711*	hypothetical protein PSPTO_3769	**1.70**	**0.87**
PSPTO_2527	*PSYR_2337*	hypothetical protein PSPTO_2527	**1.68**	**1.31**
PSPTO_1698	*PSYR_3691*	hypothetical protein PSPTO_1698	**1.67**	**0.80**
PSPTO_5149	*PSYR_0390*	hypothetical protein PSPTO_5149	**1.66**	**1.12**
PSPTO_0226	*PSYR_0181*	*lppL*, lipoprotein LppL	**1.66**	**1.21**
PSPTO_0076	*PSYR_0212*	hypothetical protein PSPTO_0076	**1.66**	**1.20**
PSPTO_1724	*PSYR_3666*	hypothetical protein PSPTO_1724	**1.66**	**1.23**
PSPTO_1614	*PSYR_3766*	hypothetical protein PSPTO_1614	**1.66**	**1.45**
PSPTO_4653	*PSYR_4286*	xanthine/uracil permease family protein	**1.65**	**0.93**
PSPTO_1728	*PSYR_3664*	hypothetical protein PSPTO_1728	**1.65**	**1.16**
PSPTO_1526	*PSYR_1334*	*sufE*, sufE protein	**1.64**	**1.03**
PSPTO_5604	*PSYR_5126*	*atpE*, F0F1 ATP synthase subunit C	**1.64**	**0.99**
PSPTO_1244	*PSYR_1064*	hypothetical protein PSPTO_1244	**1.63**	**1.12**
PSPTO_1487	*PSYR_1297*	hypothetical protein PSPTO_1487	**1.63**	**1.29**
PSPTO_4779	*PSYR_4328*	hypothetical protein PSPTO_4779	**1.62**	**2.10**
PSPTO_4121	*PSYR_3857*	hypothetical protein PSPTO_4121	**1.61**	**1.01**
PSPTO_1820	*PSYR_3577*	hypothetical protein PSPTO_1820	**1.60**	**1.44**
PSPTO_4637	*PSYR_4272*	hypothetical protein PSPTO_4637	**1.59**	**2.65**
PSPTO_5013	*PSYR_0510*	hypothetical protein PSPTO_5013	**1.58**	**0.98**
PSPTO_4472	*PSYR_4163*	*mreB*, rod shape-determining protein MreB	**1.57**	**0.68**
PSPTO_1430	*PSYR_1244*	*ndk*, nucleoside diphosphate kinase	**1.56**	**0.21**
PSPTO_0570	*PSYR_4605*	transcriptional regulator PrtN	**1.56**	**2.35**
PSPTO_4022	*PSYR_1389*	hypothetical protein PSPTO_4022	**1.55**	**1.09**

PSPTO_0240	*PSYR_4645*	prevent-host-death family protein	**1.55**	**3.43**
PSPTO_0097	*PSYR_0231*	hypothetical protein PSPTO_0097	**1.53**	**1.25**
PSPTO_3985	*PSYR_1402*	hypothetical protein PSPTO_3985	**1.53**	**1.12**
PSPTO_4951	*PSYR_0563*	hypothetical protein PSPTO_4951	**1.52**	**1.44**
PSPTO_4228	*PSYR_3962*	hypothetical protein PSPTO_4228	**1.51**	**1.03**
PSPTO_4401	*PSYR_4095*	hypothetical protein PSPTO_4401	**1.51**	**1.04**
PSPTO_5046	*PSYR_0477*	hypothetical protein PSPTO_5046	**1.50**	**1.19**
PSPTO_1467	*PSYR_1276*	hypothetical protein PSPTO_1467	**1.50**	**0.93**
PSPTO_2049	*PSYR_1859*	hypothetical protein PSPTO_2049	**1.49**	**2.44**
PSPTO_0990	*PSYR_0855*	hypothetical protein PSPTO_0990	**1.48**	**0.81**
PSPTO_1732	*PSYR_3660*	hypothetical protein PSPTO_1732	**1.48**	**1.27**
PSPTO_3968	*PSYR_1419*	*exsB*, exsB protein	**1.47**	**0.31**
PSPTO_0828	*PSYR_0727*	hypothetical protein PSPTO_0828	**1.47**	**1.36**
PSPTO_0368	*PSYR_4808*	hypothetical protein PSPTO_0368	**1.46**	**0.17**
PSPTO_2608	*PSYR_2285*	Sco1/SenC family protein	**1.46**	**1.17**
PSPTO_0121	*PSYR_0066*	membrane protein	**1.45**	**0.67**
PSPTO_2365	*PSYR_2149*	hypothetical protein PSPTO_2365	**1.45**	**2.06**
PSPTO_1140	*PSYR_0980*	hypothetical protein PSPTO_1140	**1.44**	**1.68**
PSPTO_1814	*PSYR_3582*	hypothetical protein PSPTO_1814	**1.44**	**0.86**
PSPTO_4901	*PSYR_4444*	membrane protein	**1.44**	**0.95**
PSPTO_5467	*PSYR_5021*	hypothetical protein PSPTO_5467	**1.43**	**0.08**
PSPTO_0228	*PSYR_0179*	hypothetical protein PSPTO_0228	**1.43**	**0.60**
PSPTO_0469	*PSYR_4704*	hypothetical protein PSPTO_0469	**1.43**	**2.07**
PSPTO_4028	*PSYR_1383*	hypothetical protein PSPTO_4028	**1.43**	**0.74**
PSPTO_2998	*PSYR_2879*	hypothetical protein PSPTO_2998	**1.42**	**1.19**
PSPTO_0436	*PSYR_4738*	hypothetical protein PSPTO_0436	**1.41**	**0.34**
PSPTO_4464	*PSYR_4155*	hypothetical protein PSPTO_4464	**1.41**	**0.76**
PSPTO_1319	*PSYR_1136*	hypothetical protein PSPTO_1319	**1.40**	**0.82**
PSPTO_5503	*PSYR_5055*	YeeE/YedE family protein	**1.40**	**1.52**
PSPTO_4803	*PSYR_4344*	hypothetical protein PSPTO_4803	**1.39**	**1.35**
PSPTO_1721	*PSYR_3668*	hypothetical protein PSPTO_1721	**1.39**	**3.98**
PSPTO_5634	*PSYR_0972*	hypothetical protein PSPTO_5634	**1.39**	**0.86**
PSPTO_1414	*PSYR_1229*	*yajC*, preprotein translocase subunit YajC	**1.38**	**1.01**
PSPTO_0343	*PSYR_0269*	*engB*, GTP-binding protein EngB	**1.38**	**1.91**
PSPTO_1331	*PSYR_1147*	acetyltransferase	**1.38**	**0.66**
PSPTO_2766	*PSYR_2495*	ABC transporter ATP-binding protein	**1.37**	**1.76**
PSPTO_1643	*PSYR_3739*	hypothetical protein PSPTO_1643	**1.37**	**0.90**
PSPTO_0723	*PSYR_0625*	hypothetical protein PSPTO_0723	**1.37**	**0.68**
PSPTO_0435	*PSYR_4739*	tRNA (guanine-N(7)-)-methyltransferase	**1.37**	**0.32**
PSPTO_5403	*PSYR_4942*	HAD-superfamily hydrolase	**1.36**	**1.61**
PSPTO_4423	*PSYR_4117*	*sspB*, stringent starvation protein B	**1.33**	**1.00**
PSPTO_4863	*PSYR_4403*	hypothetical protein PSPTO_4863	**1.31**	**0.69**
PSPTO_4949	*PSYR_0565*	membrane protein	**1.31**	**1.13**
PSPTO_5011	*PSYR_0512*	GNAT family acetyltransferase	**1.31**	**1.10**

PSPTO_1829	*PSYR_3568*	hypothetical protein PSPTO_1829	**1.30**	**0.54**
PSPTO_0989	*PSYR_0854*	hypothetical protein PSPTO_0989	**1.30**	**0.32**
PSPTO_3480	*PSYR_3261*	methyl-accepting chemotaxis protein	**1.30**	**2.49**
PSPTO_2037	*PSYR_1847*	Smr domain protein	**1.29**	**1.85**
PSPTO_3359	*PSYR_3191*	hypothetical protein PSPTO_3359	**1.28**	**0.31**
PSPTO_4958	*PSYR_0556*	HAMP domain protein	**1.27**	**0.88**
PSPTO_3637	*PSYR_3395*	FlhB domain-containing protein	**1.26**	**0.81**
PSPTO_3710	*PSYR_1765*	GNAT family acetyltransferase	**1.24**	**1.52**
PSPTO_5091	*PSYR_0439*	hypothetical protein PSPTO_5091	**1.24**	**0.55**
PSPTO_3654	*PSYR_1821*	GNAT family acetyltransferase	**1.24**	**1.65**
PSPTO_2270	*PSYR_2070*	ErfK/YbiS/YcfS/YnhG family protein	**1.24**	**1.36**
PSPTO_3723	*PSYR_1750*	*hupB*, DNA-binding protein HU-beta	**1.24**	**0.11**
PSPTO_0215	*PSYR_0191*	hypothetical protein PSPTO_0215	**1.23**	**1.76**
PSPTO_1273	*PSYR_1093*	membrane protein	**1.23**	**1.00**
PSPTO_0804	*PSYR_0708*	membrane protein, MviN family	**1.23**	**0.60**
PSPTO_2284	*PSYR_2082*	membrane protein	**1.23**	**1.47**
PSPTO_4827	*PSYR_4367*	iojap-like protein	**1.23**	**1.53**
PSPTO_5095	*PSYR_0435*	membrane protein	**1.23**	**1.61**
PSPTO_4393	*PSYR_4087*	hypothetical protein PSPTO_4393	**1.22**	**1.57**
PSPTO_0081	*PSYR_0217*	hypothetical protein PSPTO_0081	**1.22**	**1.01**
PSPTO_2187	*PSYR_1997*	hypothetical protein PSPTO_2187	**1.22**	**0.54**
PSPTO_0697	*PSYR_4455*	hypothetical protein PSPTO_0697	**1.22**	**1.28**
PSPTO_5297	*PSYR_4855*	hypothetical protein PSPTO_5297	**1.22**	**0.68**
PSPTO_4663	*PSYR_4296*	hypothetical protein PSPTO_4663	**1.21**	**1.27**
PSPTO_0035	*PSYR_1221*	ISPsy5, transposase	**1.21**	**0.69**
PSPTO_0688	*PSYR_4464*	lipoprotein	**1.20**	**0.33**
PSPTO_1686	*PSYR_3703*	*wrbA*, trp repressor binding protein	**1.20**	**1.40**
PSPTO_2214	*PSYR_2023*	hypothetical protein PSPTO_2214	**1.20**	**1.47**
PSPTO_1127	*PSYR_0966*	hypothetical protein PSPTO_1127	**1.20**	**1.57**
PSPTO_4418	*PSYR_4112*	tetrapyrrole methylase family protein	**1.20**	**1.26**
PSPTO_2221	*PSYR_2029*	lipoprotein	**1.19**	**0.33**
PSPTO_2038	*PSYR_1848*	hypothetical protein PSPTO_2038	**1.19**	**1.76**
PSPTO_5175	*PSYR_0363*	hypothetical protein PSPTO_5175	**1.17**	**0.71**
PSPTO_0735	*PSYR_0636*	LrgA family protein	**1.17**	**1.06**
PSPTO_4865	*PSYR_4405*	*fis*, global DNA-binding transcriptional dual regulator Fis	**1.16**	**0.64**
PSPTO_1145	*PSYR_0985*	hypothetical protein PSPTO_1145	**1.16**	**1.06**
PSPTO_3581	*PSYR_3352*	GNAT family acetyltransferase	**1.16**	**1.05**
PSPTO_4145	*PSYR_3884*	*capB*, cold shock protein CapB	**1.16**	**1.84**
PSPTO_0603	*PSYR_4570*	hypothetical protein PSPTO_0603	**1.15**	**0.51**
PSPTO_1180	*PSYR_1018*	hypothetical protein PSPTO_1180	**1.14**	**0.96**
PSPTO_0345	*PSYR_0271*	hypothetical protein PSPTO_0345	**1.14**	**0.82**
PSPTO_2360	*PSYR_2144*	hypothetical protein PSPTO_2360	**1.13**	**0.68**
PSPTO_1179	*PSYR_1017*	HopJ1 protein	**1.13**	**1.20**

PSPTO_5012	*PSYR_0511*	hypothetical protein PSPTO_5012	**1.13**	**0.98**
PSPTO_4032	*PSYR_1379*	*recX,* recX protein	**1.12**	**0.84**
PSPTO_2002	*PSYR_3415*	*ccoQ*, cytochrome c oxidase, cbb3-type, CcoQ subunit	**1.11**	**0.82**
PSPTO_3802	*PSYR_1676*	1-aminocyclopropane-1-carboxylate deaminase	**1.11**	**0.51**
PSPTO_0651	*PSYR_4524*	*rpoA*, DNA-directed RNA polymerase subunit alpha	**1.11**	**0.04**
PSPTO_3804	*PSYR_1674*	hydrolase	**1.11**	**0.13**
PSPTO_5048	*PSYR_0475*	hypothetical protein PSPTO_5048	**1.11**	**0.73**
PSPTO_5226	*PSYR_0318*	hypothetical protein PSPTO_5226	**1.11**	**1.82**
PSPTO_5151	*PSYR_0388*	hypothetical protein PSPTO_5151	**1.11**	**0.89**
PSPTO_2580	*PSYR_2271*	membrane protein	**1.10**	**0.35**
PSPTO_4195	*PSYR_3929*	hypothetical protein PSPTO_4195	**1.10**	**1.72**
PSPTO_2468	*PSYR_2233*	hypothetical protein PSPTO_2468	**1.10**	**1.18**
PSPTO_4802	*PSYR_4343*	hypothetical protein PSPTO_4802	**1.10**	**0.88**
PSPTO_4449	*PSYR_4143*	phosphatase, YrbI family	**1.10**	**0.88**
PSPTO_0825	*PSYR_0724*	hypothetical protein PSPTO_0825	**1.10**	**0.20**
PSPTO_1126	*PSYR_0965*	amine oxidase, flavin-containing protein	**1.09**	**1.24**
PSPTO_4443	*PSYR_4137*	STAS domain-containing protein	**1.09**	**1.20**
PSPTO_0415	*PSYR_4760*	hypothetical protein PSPTO_0415	**1.09**	**1.01**
PSPTO_2052	*PSYR_1861*	hypothetical protein PSPTO_2052	**1.09**	**0.77**
PSPTO_0971	*PSYR_0837*	*sfsA*, sugar fermentation stimulation protein	**1.09**	**0.19**
PSPTO_4159	*PSYR_3896*	bacterioferritin-associated ferredoxin	**1.09**	**0.30**
PSPTO_1810	*PSYR_3586*	PHP domain-containing protein	**1.08**	**1.22**
PSPTO_4428	*PSYR_4122*	ATPase	**1.08**	**0.43**
PSPTO_0992	*PSYR_0857*	*rimI*, ribosomal-protein-alanine acetyltransferase	**1.07**	**1.19**
PSPTO_4583	*PSYR_4257*	hypothetical protein PSPTO_4583	**1.06**	**1.49**
PSPTO_2254	*PSYR_2059*	methyl-accepting chemotaxis protein	**1.05**	**0.68**
PSPTO_0282	*PSYR_0115*	membrane protein	**1.05**	**0.23**
PSPTO_2469	*PSYR_2234*	hypothetical protein PSPTO_2469	**1.04**	**1.20**
PSPTO_2072	*PSYR_1882*	auxin-binding protein	**1.03**	**0.41**
PSPTO_5008	*PSYR_0515*	hypothetical protein PSPTO_5008	**1.03**	**0.91**
PSPTO_5252	*PSYR_0291*	DedA family protein	**1.03**	**1.29**
PSPTO_4826	*PSYR_4366*	hypothetical protein PSPTO_4826	**1.02**	**1.36**
PSPTO_5338	*PSYR_4897*	*hisB*, imidazoleglycerol-phosphate dehydratase	**1.02**	**0.89**
PSPTO_4185	*PSYR_3921*	membrane protein	**1.02**	**1.52**
PSPTO_2721	*PSYR_2454*	membrane protein	**1.01**	**0.72**
PSPTO_2609	*PSYR_2286*	hypothetical protein PSPTO_2609	**1.01**	**0.79**
PSPTO_2375	*PSYR_2159*	lipoprotein	**1.00**	**1.45**
PSPTO_4946	*PSYR_0568*	hypothetical protein PSPTO_4946	**0.95**	**1.10**

PSPTO_1197	*PSYR_1033*	hypothetical protein PSPTO_1197	**0.95**	**1.14**
PSPTO_2247	*PSYR_2052*	lipoprotein	**0.95**	**1.10**
PSPTO_5281	*PSYR_4839*	hypothetical protein PSPTO_5281	**0.90**	**1.34**
PSPTO_2350	*PSYR_2134*	lipoprotein	**0.89**	**1.01**
PSPTO_3839	*PSYR_1640*	HAD-superfamily hydrolase	**0.88**	**1.78**
PSPTO_5224	*PSYR_0320*	hypothetical protein PSPTO_5224	**0.86**	**1.08**
PSPTO_3672	*PSYR_1803*	membrane protein	**0.85**	**1.21**
PSPTO_4959	*PSYR_0555*	lipoprotein	**0.84**	**1.03**
PSPTO_4974	*PSYR_0546*	lipoprotein	**0.83**	**1.71**
PSPTO_0217	*PSYR_0190*	nitrogen regulatory protein P-II	**0.80**	**1.14**
PSPTO_0322	*PSYR_0252*	adenylate cyclase	**0.80**	**1.24**
PSPTO_0438	*PSYR_4736*	hypothetical protein PSPTO_0438	**0.79**	**1.15**
PSPTO_5517	*PSYR_5066*	hypothetical protein PSPTO_5517	**0.78**	**1.81**
PSPTO_3859	*PSYR_1626*	hypothetical protein PSPTO_3859	**0.72**	**1.63**
PSPTO_5225	*PSYR_0319*	hypothetical protein PSPTO_5225	**0.71**	**1.36**
PSPTO_1776	*PSYR_3618*	hypothetical protein PSPTO_1776	**0.68**	**1.46**
PSPTO_1184	*PSYR_1022*	hypothetical protein PSPTO_1184	**0.65**	**1.11**
PSPTO_4186	*PSYR_3922*	hypothetical protein PSPTO_4186	**0.65**	**1.61**
PSPTO_0468	*PSYR_4705*	hypothetical protein PSPTO_0468	**0.62**	**1.63**
PSPTO_1123	*PSYR_0963*	hypothetical protein PSPTO_1123	**0.60**	**1.29**
PSPTO_2774	*PSYR_2502*	hypothetical protein PSPTO_2774	**0.58**	**1.24**
PSPTO_4279	*PSYR_2932*	hypothetical protein PSPTO_4279	**0.58**	**1.36**
PSPTO_0220	*PSYR_0187*	hypothetical protein PSPTO_0220	**0.57**	**1.53**
PSPTO_1304	*PSYR_1124*	hypothetical protein PSPTO_1304	**0.57**	**1.40**
PSPTO_2649	*PSYR_2381*	hypothetical protein PSPTO_2649	**0.56**	**1.36**
PSPTO_3169	*PSYR_3034*	hypothetical protein PSPTO_3169	**0.55**	**2.38**
PSPTO_1824	*PSYR_3573*	hypothetical protein PSPTO_1824	**0.55**	**1.19**
PSPTO_4194	*PSYR_3928*	hypothetical protein PSPTO_4194	**0.54**	**1.37**
PSPTO_5553	*PSYR_5092*	methyl-accepting chemotaxis protein	**0.52**	**1.34**
PSPTO_1143	*PSYR_0983*	hypothetical protein PSPTO_1143	**0.50**	**1.01**
PSPTO_2025	*PSYR_1834*	hypothetical protein PSPTO_2025	**0.49**	**1.03**
PSPTO_1627	*PSYR_3753*	short chain dehydrogenase	**0.48**	**1.29**
PSPTO_4379	*PSYR_4074*	hypothetical protein PSPTO_4379	**0.47**	**1.42**
PSPTO_4248	*PSYR_3982*	3-hydroxyacyl-CoA-acyl carrier protein transferase	**0.44**	**1.01**
PSPTO_0381	*PSYR_4797*	hypothetical protein PSPTO_0381	**0.43**	**1.53**
PSPTO_1777	*PSYR_3617*	thiopurine s-methyltransferase	**0.41**	**1.96**
PSPTO_0330	*PSYR_0260*	hypothetical protein PSPTO_0330	**0.40**	**3.05**
PSPTO_3709	*PSYR_1766*	hypothetical protein PSPTO_3709	**0.39**	**1.75**
PSPTO_0340	*PSYR_0267*	endonuclease/exonuclease/phosphatase family protein	**0.35**	**1.16**
PSPTO_2790	*PSYR_2518*	hypothetical protein PSPTO_2790	**0.33**	**1.47**
PSPTO_1625	*PSYR_3755*	hypothetical protein PSPTO_1625	**0.33**	**2.07**
PSPTO_3110	*PSYR_2977*	cointegrate resolution protein T	**0.32**	**1.20**

PSPTO_0208	*PSYR_0192*	hypothetical protein PSPTO_0208	**0.32**	**1.35**
PSPTO_3895	*PSYR_1589*	hypothetical protein PSPTO_3895	**0.31**	**1.56**
PSPTO_5169	*PSYR_0369*	lipoprotein	**0.28**	**2.08**
PSPTO_3587	*PSYR_3358*	hypothetical protein PSPTO_3587	**0.27**	**1.18**
PSPTO_4881	*PSYR_4422*	hypothetical protein PSPTO_4881	**0.18**	**1.33**
PSPTO_2769	*PSYR_2497*	lipoprotein	**0.17**	**2.65**
PSPTO_0219	*PSYR_0188*	hypothetical protein PSPTO_0219	**0.16**	**1.05**
PSPTO_2029	*PSYR_1837*	oxidoreductase zinc-binding protein	**0.10**	**1.17**
PSPTO_0318	*PSYR_0248*	hypothetical protein PSPTO_0318	**0.10**	**1.49**
PSPTO_1008	*PSYR_0871*	methyl-accepting chemotaxis protein	**-0.10**	**-1.53**
PSPTO_1504	*PSYR_1314*	lipoprotein	**-0.12**	**-1.07**
PSPTO_5194	*PSYR_1088*	hypothetical protein PSPTO_5194	**-0.17**	**-1.17**
PSPTO_1619	*PSYR_3761*	hypothetical protein PSPTO_1619	**-0.21**	**-1.33**
PSPTO_1045	*PSYR_0894*	virulence-associated protein	**-0.24**	**-1.82**
PSPTO_0863	*PSYR_1446*	hypothetical protein PSPTO_0863	**-0.25**	**-1.26**
PSPTO_1193	*PSYR_1029*	hypothetical protein PSPTO_1193	**-0.25**	**-1.96**
PSPTO_1194	*PSYR_1030*	bacteriophage N4 adsorption protein B	**-0.26**	**-1.62**
PSPTO_3513	*PSYR_3286*	hypothetical protein PSPTO_3513	**-0.30**	**-1.05**
PSPTO_1040	*PSYR_0887*	sensory box protein	**-0.30**	**-1.38**
PSPTO_1267	*PSYR_1085*	hypothetical protein PSPTO_1267	**-0.32**	**-2.01**
PSPTO_5318	*PSYR_4878*	outer membrane porin, OprD family	**-0.33**	**-1.62**
PSPTO_0713	*PSYR_0619*	hypothetical protein PSPTO_0713	**-0.35**	**-1.13**
PSPTO_0253	*PSYR_0153*	hypothetical protein PSPTO_0253	**-0.38**	**-1.39**
PSPTO_2893	*PSYR_3092*	PAP2 superfamily protein	**-0.39**	**-1.24**
PSPTO_2870	*PSYR_2633*	hypothetical protein PSPTO_2870	**-0.40**	**-1.89**
PSPTO_1192	*PSYR_1028*	hypothetical protein PSPTO_1192	**-0.41**	**-1.27**
PSPTO_3906	*PSYR_1579*	hypothetical protein PSPTO_3906	**-0.42**	**-1.24**
PSPTO_5119	*PSYR_0413*	hypothetical protein PSPTO_5119	**-0.42**	**-1.28**
PSPTO_3201	*PSYR_3067*	hypothetical protein PSPTO_3201	**-0.43**	**-1.40**
PSPTO_0958	*PSYR_0825*	hypothetical protein PSPTO_0958	**-0.45**	**-1.53**
PSPTO_5158	*PSYR_0381*	hypothetical protein PSPTO_5158	**-0.45**	**-2.10**
PSPTO_0975	*PSYR_0840*	pentapeptide repeat-containing protein	**-0.46**	**-1.62**
PSPTO_5106	*PSYR_0426*	lipoprotein	**-0.48**	**-1.20**
PSPTO_0859	*PSYR_1442*	hypothetical protein PSPTO_0859	**-0.53**	**-1.08**
PSPTO_4815	*PSYR_4355*	hypothetical protein PSPTO_4815	**-0.53**	**-2.26**
PSPTO_2589	*PSYR_2279*	hypothetical protein PSPTO_2589	**-0.54**	**-1.96**
PSPTO_2954	*PSYR_2739*	ThiJ/PfpI family protein	**-0.60**	**-1.43**
PSPTO_2815	*PSYR_2543*	hypothetical protein PSPTO_2815	**-0.60**	**-1.56**
PSPTO_1150	*PSYR_0992*	hypothetical protein PSPTO_1150	**-0.61**	**-1.09**
PSPTO_3032	*PSYR_2905*	peptide ABC transporter ATP-binding protein	**-0.62**	**-1.15**
PSPTO_1847	*PSYR_1528*	transcriptional repressor	**-0.63**	**-1.58**
PSPTO_5105	*PSYR_0427*	hypothetical protein PSPTO_5105	**-0.63**	**-1.54**
PSPTO_5361	*PSYR_4915*	hypothetical protein PSPTO_5361	**-0.63**	**-1.01**

PSPTO_0254	*PSYR_0152*	pyridine nucleotide-disulfide oxidoreductase family protein	**-0.65**	**-1.00**
PSPTO_2959	*PSYR_2744*	hypothetical protein PSPTO_2959	**-0.66**	**-1.54**
PSPTO_5385	*PSYR_4925*	hypothetical protein PSPTO_5385	**-0.66**	**-1.38**
PSPTO_2962	*PSYR_2747*	hypothetical protein PSPTO_2962	**-0.69**	**-1.40**
PSPTO_1061	*PSYR_0906*	methyl-accepting chemotaxis protein	**-0.69**	**-1.16**
PSPTO_4936	*PSYR_0578*	methyl-accepting chemotaxis protein	**-0.70**	**-1.26**
PSPTO_1621	*PSYR_3759*	hypothetical protein PSPTO_1621	**-0.71**	**-1.12**
PSPTO_3572	*PSYR_3344*	hypothetical protein PSPTO_3572	**-0.71**	**-1.34**
PSPTO_1118	*PSYR_0958*	amine oxidase, flavin-containing protein	**-0.72**	**-1.57**
PSPTO_2880	*PSYR_2623*	hypothetical protein PSPTO_2880	**-0.72**	**-2.77**
PSPTO_4584	*PSYR_4258*	hypothetical protein PSPTO_4584	**-0.74**	**-1.61**
PSPTO_1339	*PSYR_0335*	membrane protein	**-0.75**	**-2.62**
PSPTO_5029	*PSYR_0493*	hypothetical protein PSPTO_5029	**-0.75**	**-1.99**
PSPTO_0263	*PSYR_0143*	methyl-accepting chemotaxis protein	**-0.77**	**-1.13**
PSPTO_5386	*PSYR_4926*	hypothetical protein PSPTO_5386	**-0.80**	**-1.33**
PSPTO_2502	*PSYR_2308*	hypothetical protein PSPTO_2502	**-0.80**	**-1.27**
PSPTO_1269	*PSYR_1089*	hypothetical protein PSPTO_1269	**-0.81**	**-1.94**
PSPTO_5554	*PSYR_5093*	methyl-accepting chemotaxis protein	**-0.81**	**-1.33**
PSPTO_0108	*PSYR_0080*	membrane protein	**-0.82**	**-1.09**
PSPTO_3777	*PSYR_1701*	membrane protein	**-0.85**	**-1.20**
PSPTO_0244	*PSYR_0161*	hypothetical protein PSPTO_0244	**-0.86**	**-1.09**
PSPTO_2729	*PSYR_2461*	hypothetical protein PSPTO_2729	**-0.89**	**-2.11**
PSPTO_2871	*PSYR_2632*	hypothetical protein PSPTO_2871	**-0.89**	**-3.58**
PSPTO_0363	*PSYR_4813*	sorbitol dehydrogenase	**-0.89**	**-3.54**
PSPTO_2700	*PSYR_2433*	hypothetical protein PSPTO_2700	**-0.92**	**-2.40**
PSPTO_1062	*PSYR_0907*	hypothetical protein PSPTO_1062	**-0.93**	**-1.45**
PSPTO_5218	*PSYR_0326*	hypothetical protein PSPTO_5218	**-0.94**	**-2.73**
PSPTO_2290	*PSYR_2088*	hypothetical protein PSPTO_2290	**-0.97**	**-1.01**
PSPTO_1066	*PSYR_0913*	methyl-accepting chemotaxis protein	**-1.00**	**-1.65**
PSPTO_0702	*PSYR_0608*	hypothetical protein PSPTO_0702	**-1.00**	**-0.62**
PSPTO_5624	*PSYR_1967*	Tat (twin-arginine translocation) pathway signal sequence domain-containing protein	**-1.01**	**-0.85**
PSPTO_1651	*PSYR_3727*	hypothetical protein PSPTO_1651	**-1.02**	**-0.82**
PSPTO_2501	*PSYR_2307*	hypothetical protein PSPTO_2501	**-1.02**	**-1.01**
PSPTO_2675	*PSYR_2409*	hypothetical protein PSPTO_2675	**-1.03**	**-0.67**
PSPTO_5432	*PSYR_4954*	hypothetical protein PSPTO_5432	**-1.04**	**-0.10**
PSPTO_2144	*PSYR_1954*	hypothetical protein PSPTO_2144	**-1.04**	**-1.05**
PSPTO_2142	*PSYR_1952*	hypothetical protein PSPTO_2142	**-1.04**	**-1.27**
PSPTO_2623	*PSYR_2363*	phenazine biosynthesis protein, PhzF family	**-1.04**	**-0.84**
PSPTO_5232	*PSYR_0310*	pyocin/colicin protein	**-1.04**	**-0.46**
PSPTO_2238	*PSYR_2043*	lipoprotein	**-1.06**	**-0.54**

PSPTO_4317	*PSYR_4021*	hypothetical protein PSPTO_4317	**-1.06**	**-0.72**
PSPTO_2147	*PSYR_1957*	pyoverdine sidechain peptide synthetase I, epsilon-Lys module	**-1.07**	**-1.07**
PSPTO_4351	*PSYR_4046*	hypothetical protein PSPTO_4351	**-1.10**	**-0.63**
PSPTO_2071	*PSYR_1881*	hypothetical protein PSPTO_2071	**-1.11**	**-1.40**
PSPTO_0758	*PSYR_0662*	2OG-Fe(II) oxygenase family oxidoreductase	**-1.11**	**-0.38**
PSPTO_0374	*PSYR_4804*	hypothetical protein PSPTO_0374	**-1.11**	**-0.27**
PSPTO_3602	*PSYR_3373*	hypothetical protein PSPTO_3602	**-1.11**	**-1.93**
PSPTO_4574	*PSYR_4248*	hypothetical protein PSPTO_4574	**-1.12**	**-0.41**
PSPTO_3880	*PSYR_1604*	polyamine ABC transporter permease	**-1.12**	**-0.61**
PSPTO_3779	*PSYR_1699*	hypothetical protein PSPTO_3779	**-1.12**	**-1.82**
PSPTO_2953	*PSYR_2738*	LysR family transcriptional regulator	**-1.15**	**-1.32**
PSPTO_5330	*PSYR_4888*	hypothetical protein PSPTO_5330	**-1.15**	**-1.46**
PSPTO_1368	*PSYR_1181*	lipoprotein	**-1.16**	**-0.40**
PSPTO_2145	*PSYR_1955*	iron-regulated membrane protein	**-1.17**	**-0.38**
PSPTO_2520	*PSYR_2331*	pyridine nucleotide-disulfide oxidoreductase family protein	**-1.17**	**-0.97**
PSPTO_0866	*PSYR_1447*	hypothetical protein PSPTO_0866	**-1.17**	**-0.45**
PSPTO_0916	*PSYR_0789*	methyl-accepting chemotaxis protein	**-1.17**	**-1.31**
PSPTO_0117	*PSYR_0071*	methyl-accepting chemotaxis protein	**-1.18**	**-0.31**
PSPTO_0867	*PSYR_1448*	hypothetical protein PSPTO_0867	**-1.19**	**-0.52**
PSPTO_2143	*PSYR_1953*	hypothetical protein PSPTO_2143	**-1.19**	**-1.30**
PSPTO_2779	*PSYR_2507*	endoribonuclease L-PSP family protein	**-1.20**	**-0.65**
PSPTO_2868	*PSYR_2635*	hypothetical protein PSPTO_2868	**-1.20**	**-0.66**
PSPTO_2480	*PSYR_2246*	methyl-accepting chemotaxis protein	**-1.21**	**-0.57**
PSPTO_1117	*PSYR_0957*	hypothetical protein PSPTO_1117	**-1.21**	**-1.78**
PSPTO_2535	*PSYR_0196*	hypothetical protein PSPTO_2535	**-1.22**	**-0.43**
PSPTO_2677	*PSYR_2411*	short chain dehydrogenase/reductase family oxidoreductase	**-1.23**	**-0.82**
PSPTO_3538	*PSYR_3310*	transferase, hexapeptide repeat protein	**-1.23**	**-2.14**
PSPTO_0711	*PSYR_0617*	hypothetical protein PSPTO_0711	**-1.24**	**-0.34**
PSPTO_2949	*PSYR_2733*	3-alpha-hydroxysteroid dehydrogenase	**-1.26**	**-0.62**
PSPTO_3091	*PSYR_2961*	nickel ABC transporter, ATP-binding protein	**-1.28**	**-1.18**
PSPTO_4370	*PSYR_4066*	hypothetical protein PSPTO_4370	**-1.28**	**-0.78**
PSPTO_4318	*PSYR_4022*	integral membrane protein	**-1.29**	**-0.94**
PSPTO_1851	*PSYR_3817*	hypothetical protein PSPTO_1851	**-1.30**	**-0.62**
PSPTO_1793	*PSYR_3601*	hypothetical protein PSPTO_1793	**-1.31**	**-2.60**
PSPTO_5054	*PSYR_4084*	hypothetical protein PSPTO_5054	**-1.31**	**-1.15**
PSPTO_2620	*PSYR_2360*	hypothetical protein PSPTO_2620	**-1.32**	**-0.83**
PSPTO_5312	*PSYR_4871*	hypothetical protein PSPTO_5312	**-1.32**	**-0.80**
PSPTO_2231	*PSYR_2040*	YD repeat protein, partial	**-1.32**	**-0.85**
PSPTO_3203	*PSYR_3069*	hypothetical protein PSPTO_3203	**-1.32**	**-0.92**

PSPTO_2515	*PSYR_2326*	lipoprotein	**-1.33**	**-0.30**
PSPTO_0507	*PSYR_4676*	hypothetical protein PSPTO_0507	**-1.34**	**-1.84**
PSPTO_0454	*PSYR_4719*	hypothetical protein PSPTO_0454	**-1.36**	**-1.37**
PSPTO_3129	*PSYR_2996*	hypothetical protein PSPTO_3129	**-1.38**	**-1.12**
PSPTO_0063	*PSYR_0199*	hypothetical protein PSPTO_0063	**-1.39**	**-0.87**
PSPTO_2778	*PSYR_2506*	amino acid ABC transporter ATP-binding protein	**-1.42**	**-0.20**
PSPTO_3539	*PSYR_3311*	membrane protein PslK	**-1.43**	**-1.65**
PSPTO_0395	*PSYR_4783*	hypothetical protein PSPTO_0395	**-1.43**	**-1.13**
PSPTO_3558	*PSYR_3334*	glcG protein	**-1.45**	**-0.51**
PSPTO_2116	*PSYR_1911*	hypothetical protein PSPTO_2116	**-1.46**	**-1.03**
PSPTO_4335	*PSYR_4027*	hypothetical protein PSPTO_4335	**-1.46**	**-0.16**
PSPTO_1167	*PSYR_0687*	hypothetical protein PSPTO_1167	**-1.46**	**-0.88**
PSPTO_1303	*PSYR_1123*	hypothetical protein PSPTO_1303	**-1.47**	**-0.56**
PSPTO_2441	*PSYR_2214*	methyl-accepting chemotaxis protein	**-1.47**	**-1.13**
PSPTO_4342	*PSYR_4035*	insecticidal toxin protein	**-1.49**	**-1.63**
PSPTO_3133	*PSYR_3000*	methyltransferase	**-1.53**	**-1.86**
PSPTO_4623	*PSYR_1490*	hypothetical protein PSPTO_4623	**-1.53**	**-1.69**
PSPTO_2514	*PSYR_2325*	hypothetical protein PSPTO_2514	**-1.54**	**-0.30**
PSPTO_0155	*PSYR_0037*	hypothetical protein PSPTO_0155	**-1.54**	**-0.19**
PSPTO_3770	*PSYR_1710*	hypothetical protein PSPTO_3770	**-1.54**	**-0.30**
PSPTO_1869	*PSYR_3533*	hypothetical protein PSPTO_1869	**-1.54**	**-1.95**
PSPTO_4206	*PSYR_3940*	globin family protein	**-1.55**	**-1.35**
PSPTO_5352	*PSYR_4907*	methyl-accepting chemotaxis protein	**-1.56**	**-1.33**
PSPTO_4207	*PSYR_3941*	hypothetical protein PSPTO_4207	**-1.56**	**-1.41**
PSPTO_2020	*PSYR_1829*	hypothetical protein PSPTO_2020	**-1.57**	**-0.97**
PSPTO_5644	*PSYR_4015*	hypothetical protein PSPTO_5644	**-1.62**	**-0.52**
PSPTO_2239	*PSYR_2044*	YD repeat protein	**-1.62**	**-0.44**
PSPTO_3331	*PSYR_3162*	protease inhibitor Inh	**-1.63**	**-0.17**
PSPTO_2391	*PSYR_2823*	hypothetical protein PSPTO_2391	**-1.64**	**-0.56**
PSPTO_2873	*PSYR_2630*	hypothetical protein PSPTO_2873	**-1.65**	**-4.73**
PSPTO_2879	*PSYR_2624*	lipoprotein	**-1.66**	**-3.87**
PSPTO_3736	*PSYR_1741*	hypothetical protein PSPTO_3736	**-1.66**	**-1.15**
PSPTO_5625	*PSYR_3989*	binary cytotoxin component	**-1.69**	**-0.66**
PSPTO_0675	*PSYR_4478*	arylesterase	**-1.71**	**-0.95**
PSPTO_3132	*PSYR_2999*	hypothetical protein PSPTO_3132	**-1.73**	**-1.77**
PSPTO_2492	*PSYR_2297*	short-chain dehydrogenase/reductase family oxidoreductase	**-1.73**	**-1.46**
PSPTO_5456	*PSYR_5010*	hypothetical protein PSPTO_5456	**-1.74**	**-2.61**
PSPTO_2442	*PSYR_2215*	chemotaxis protein CheW	**-1.74**	**-2.94**
PSPTO_2796	*PSYR_2524*	hypothetical protein PSPTO_2796	**-1.74**	**-0.92**
PSPTO_3547	*PSYR_3321*	LysR family transcriptional regulator	**-1.77**	**-1.08**
PSPTO_3120	*PSYR_2987*	Cof-like hydrolase family protein	**-1.78**	**-1.48**
PSPTO_0914	*PSYR_0787*	STAS domain-containing protein	**-1.78**	**-1.49**

PSPTO_5455	*PSYR_5009*	hypothetical protein PSPTO_5455	**-1.83**	**-2.62**
PSPTO_2692	*PSYR_2425*	hypothetical protein PSPTO_2692	**-1.83**	**-0.39**
PSPTO_3546	*PSYR_3320*	hypothetical protein PSPTO_3546	**-1.84**	**-1.08**
PSPTO_1165	*PSYR_3731*	hypothetical protein PSPTO_1165	**-1.84**	**-1.35**
PSPTO_4611	*PSYR_1477*	hypothetical protein PSPTO_4611	**-1.85**	**-2.27**
PSPTO_3299	*PSYR_3129*	3-hydroxyacyl-CoA-acyl carrier protein transferase	**-1.86**	**-4.12**
PSPTO_5233	*PSYR_0309*	colicin/pyocin immunity family protein	**-1.87**	**-0.80**
PSPTO_3600	*PSYR_3371*	oxidoreductase, molybdopterin-binding protein	**-1.89**	**-1.43**
PSPTO_3131	*PSYR_2998*	hypothetical protein PSPTO_3131	**-1.91**	**-1.85**
PSPTO_5458	*PSYR_5012*	hypothetical protein PSPTO_5458	**-1.99**	**-1.96**
PSPTO_3879	*PSYR_1605*	hypothetical protein PSPTO_3879	**-2.04**	**-1.05**
PSPTO_1334	*PSYR_1150*	methyl-accepting chemotaxis protein	**-2.06**	**-1.28**
PSPTO_2874	*PSYR_2629*	ppkA-related protein	**-2.07**	**-5.03**
PSPTO_2877	*PSYR_2626*	hypothetical protein PSPTO_2877	**-2.09**	**-4.10**
PSPTO_4586	*PSYR_4260*	hypothetical protein PSPTO_4586	**-2.11**	**-0.49**
PSPTO_0307	*PSYR_0085*	hypothetical protein PSPTO_0307	**-2.16**	**-2.54**
PSPTO_2472	*PSYR_2237*	methyl-accepting chemotaxis protein	**-2.18**	**-0.58**
PSPTO_5234	*PSYR_0308*	hypothetical protein PSPTO_5234	**-2.19**	**-1.00**
PSPTO_4516	*PSYR_4204*	hypothetical protein PSPTO_4516	**-2.20**	**-0.36**
PSPTO_1919	*PSYR_3493*	hypothetical protein PSPTO_1919	**-2.21**	**-0.20**
PSPTO_3494	*PSYR_3268*	myo-inositol 2-dehydrogenase	**-2.21**	**-1.03**
PSPTO_2872	*PSYR_2631*	HopL1 protein	**-2.22**	**-4.18**
PSPTO_4719	*PSYR_1432*	hypothetical protein PSPTO_4719	**-2.29**	**-0.42**
PSPTO_2895	*PSYR_2699*	hypothetical protein PSPTO_2895	**-2.31**	**-1.31**
PSPTO_4587	*PSYR_4261*	hypothetical protein PSPTO_4587	**-2.32**	**-0.14**
PSPTO_2878	*PSYR_2625*	lipoprotein	**-2.42**	**-3.51**
PSPTO_3691	*PSYR_2345*	ea59 protein	**-2.50**	**-2.51**
PSPTO_3492	*PSYR_3266*	oxidoreductase, Gfo/Idh/MocA family	**-2.50**	**-0.25**
PSPTO_4610	*PSYR_1476*	hypothetical protein PSPTO_4610	**-2.56**	**-2.46**
PSPTO_4334	*PSYR_4026*	hypothetical protein PSPTO_4334	**-2.59**	**-1.18**
PSPTO_3690	*PSYR_2346*	hypothetical protein PSPTO_3690	**-2.61**	**-1.08**
PSPTO_2696	*PSYR_2430*	mutT/nudix family protein	**-2.72**	**-0.49**
PSPTO_4333	*PSYR_4025*	moxR protein	**-2.87**	**-1.55**
PSPTO_4372	*PSYR_4068*	hypothetical protein PSPTO_4372	**-2.89**	**-1.06**
PSPTO_0545	*PSYR_4633*	SpoVR like family protein	**-4.11**	**-3.89**
PSPTO_0546	*PSYR_4632*	hypothetical protein PSPTO_0546	**-4.14**	**-4.41**
