# Supplementary material for: Comparative transcriptomic analysis of global gene expression mediated by (p) ppGpp reveals common regulatory networks in Pseudomonas syringae
Source: BMC Genomics. 2020 Apr 10;21:296. doi: 10.1186/s12864-020-6701-2 (PMC7146990; doi:10.1186/s12864-020-6701-2)
Supplement: Supplementary file 3 — Additional file 3: Table S3. List of homologues and unique genes via comparative analysis of PstDC3000 and PssB728a genomes. Table S4. List of homologues genes in both PstDC3000 and PssB728a regulated by (p) ppGpp in a similar way. Table S5. List of homologues genes in both PstDC3000 and PssB728a inversely regulated by (p)ppGpp. [file 12864_2020_6701_MOESM3_ESM.zip › Additional file 3 Table S5.pdf]

Table S5. List of homologues genes in both *Pst*DC3000 and *Pss*B728a regulated by (p)ppGpp in an opposite way with p-value <0.05.

Locus tag		Gene description	(p)ppGpp^{0}$_{Pst\mathrm{DC3000}}$ /*Pst*DC3000	(p)ppGpp^{0}$_{Pss\mathrm{B728a}}$ /*Pss*B728a
**DC3000**	**B728a**			
**Translation, ribosomal structure and biogenesis**				
PSPTO_4183	*PSYR_3920*	*rpmE-1*, 50S ribosomal protein L31 type B	**1.90**	**-0.51**
PSPTO_1431	*PSYR_1245*	radical SAM enzyme, Cfr family	**1.61**	**-0.10**
PSPTO_5559	*PSYR_5100*	hypothetical protein PSPTO_5559	**0.26**	**-1.3**
PSPTO_4468	*PSYR_4159*	*cafA*, ribonuclease G	**0.26**	**-1.48**
**Transcription**				
PSPTO_3698	*PSYR_1777*	TetR family transcriptional regulator	**1.34**	**-1.55**
PSPTO_3617	*PSYR_3377*	MarR family transcriptional regulator	**1.06**	**-0.33**
PSPTO_0953	*PSYR_0820*	DNA-binding transcriptional regulator FruR	**0.63**	**-1.02**
PSPTO_0365	*PSYR_4811*	AraC family transcriptional regulator	**0.61**	**-1.31**
PSPTO_4997	*PSYR_0526*	hypothetical protein PSPTO_4997	**0.11**	**-1.91**
PSPTO_2048	*PSYR_1858*	LuxR family transcriptional regulator	**-0.58**	**1.23**
PSPTO_1083	*PSYR_2812*	peptidase, S24 family	**-0.74**	**1.09**
PSPTO_2951	*PSYR_2735*	sigma-54 dependent transcriptional regulator	**-1.14**	**0.28**
PSPTO_2827	*PSYR_2573*	hypothetical protein PSPTO_2827, partial	**-1.19**	**0.53**
PSPTO_0157	*PSYR_0035*	*trpI*, trpBA operon transcriptional activator	**-1.27**	**0.143**
PSPTO_3549	*PSYR_3324*	*aefR*, transcriptional regulator AefR	**-1.30**	**0.76**
PSPTO_4491	*PSYR_4181*	*nusA*, N utilization substance protein A	**-1.42**	**0.15**
PSPTO_4224	*PSYR_3958*	*rpoE*, RNA polymerase sigma-24 factor	**-1.45**	**0.63**
**Replication, recombination and repair**				
PSPTO_1015	*PSYR_1888*	integrase/recombinase XerC	**0.27**	**-1.47**
PSPTO_4814	*PSYR_4354*	*holA*, DNA polymerase III subunit delta	**0.26**	**-1.59**
PSPTO_4997	*PSYR_0526*	hypothetical protein PSPTO_4997	**0.11**	**-1.91**
PSPTO_5344	*PSYR_1226*	site-specific recombinase, phage integrase family	**-0.29**	**1.40**
**Cell cycle control, cell division, chromosome partitioning**				
PSPTO_4469	*PSYR_4160*	*maf-2*, maf protein	**0.40**	**-1.31**
PSPTO_1986	*PSYR_3430*	ParA, family protein	**0.35**	**-1.58**
**Defense mechanisms**				
PSPTO_0681	*PSYR_4471*	permease	**1.25**	**-0.08**
**Signal transduction mechanisms**				
PSPTO_2223	*PSYR_2032*	DNA-binding response regulator	**1.03**	**-0.67**

PSPTO_4998	*PSYR_0525*	lipopolysaccharide biosynthesis protein	**0.37**	**-1.51**
PSPTO_4836	*PSYR_4376*	DNA binding response regulator, LuxR family	**0.30**	**-2.75**
PSPTO_4999	*PSYR_0524*	lipopolysaccharide core biosynthesis protein	**0.23**	**-1.35**
PSPTO_4997	*PSYR_0526*	hypothetical protein PSPTO_4997	**0.11**	**-1.91**
PSPTO_0378	*PSYR_4800*	DNA-binding heavy metal response regulator	**-0.12**	**1.95**
PSPTO_0353	*PSYR_4821*	*ntrB*, nitrogen regulation protein NtrB	**-0.29**	**2.47**
PSPTO_0352	*PSYR_4822*	*ntrC*, nitrogen regulation protein NR(I)	**-0.60**	**1.61**
PSPTO_1910	*PSYR_3497*	hypothetical protein PSPTO_1910	**-0.62**	**2.31**
PSPTO_2326	*PSYR_2113*	extracellular solute-binding protein/sensory box protein, partial	**-0.62**	**1.64**
PSPTO_0379	*PSYR_4799*	heavy metal sensor histidine kinase	**-0.70**	**1.66**
PSPTO_4222	*PSYR_3956*	*mucB*, sigma factor algU regulatory protein MucB	**-1.01**	**0.84**
PSPTO_4223	*PSYR_3957*	*mucA*, sigma factor algU negative regulatory protein MucA	**-1.20**	**0.68**
PSPTO_2131	*PSYR_1941*	sensor histidine kinase	**-1.33**	**0.50**
PSPTO_2129	*PSYR_1939*	sensory box histidine kinase/response regulator	**-2.37**	**0.09**
PSPTO_2130	*PSYR_1940*	LuxR family DNA-binding response regulator	**-2.73**	**0.84**
**Cell wall/membrane/envelope biogenesis**				
PSPTO_4813	*PSYR_4353*	lipoprotein	**1.66**	**-0.67**
PSPTO_4470	*PSYR_4161*	rod shape-determining protein MreD	**1.58**	**-0.65**
PSPTO_1705	*PSYR_3684*	NLP/P60 family protein	**1.16**	**-0.25**
PSPTO_4414	*PSYR_4108*	penicillin-binding protein	**1.04**	**-0.44**
PSPTO_1553	*PSYR_1362*	*kdsA*, 2-dehydro-3-deoxyphosphooctonate aldolase	**0.59**	**-1.03**
PSPTO_4411	*PSYR_4105*	*mraY*, phospho-N-acetylmuramoyl-pentapeptide-transferase	**0.26**	**-1.72**
PSPTO_4413	*PSYR_4107*	*murE*, UDP-N-acetylmuramoylalanyl-D-glutamate--2,6-diaminopimelate ligase	**0.26**	**-1.75**
PSPTO_1543	*PSYR_1352*	outer membrane protein OmpH	**0.19**	**-1.82**
PSPTO_1546	*PSYR_1355*	*lpxA*, UDP-N-acetylglucosamine acyltransferase	**0.17**	**-2.14**
PSPTO_1417	*PSYR_1232*	hypothetical protein PSPTO_1417	**-0.18**	**2.37**
PSPTO_3649	*PSYR_1822*	mechanosensitive ion channel family protein	**-0.37**	**1.19**
PSPTO_1506	*PSYR_1316*	ompA family protein	**-1.26**	**0.28**
PSPTO_1243	*PSYR_1063*	*algD*, GDP-mannose 6-dehydrogenase	**-1.98**	**0.91**

		AlgD		
PSPTO_3648	*PSYR_1823*	*plcA1*, acid phosphatase	**-2.19**	**0.23**
**Cell motility**				
PSPTO_1432	*PSYR_1246*	*pilF*, type IV pilus biogenesis protein PilF	**1.29**	**-0.85**
PSPTO_1940	*PSYR_3475*	*flgG*, flagellar basal-body rod protein FlgG	**0.45**	**-1.27**
PSPTO_1942	*PSYR_3473*	*flgI*, flagellar P-ring protein FlgI	**0.36**	**-1.43**
PSPTO_1973	*PSYR_3443*	*fliQ*, flagellar biosynthetic protein FliQ	**0.22**	**-1.36**
PSPTO_1941	*PSYR_3474*	*flgH*, flagellar L-ring protein FlgH	**0.61**	**-1.36**
**Intracellular trafficking, secretion, and vesicular transport**				
PSPTO_4219	*PSYR_3953*	*lepB*, signal peptidase I	**1.75**	**-0.10**
PSPTO_1432	*PSYR_1246*	*pilF*, type IV pilus biogenesis protein PilF	**1.29**	**-0.85**
PSPTO_1416	*PSYR_1231*	*secF*, protein-export membrane protein SecF	**1.25**	**-0.91**
PSPTO_4869	*PSYR_4409*	membrane protein, MarC family	**1.20**	**-0.41**
PSPTO_0810	*PSYR_0714*	type IV pilus biogenesis protein	**1.18**	**-0.35**
PSPTO_5157	*PSYR_0382*	*tatC*, sec-independent protein translocase TatC	**0.13**	**-1.67**
PSPTO_0927	*PSYR_0799*	type IV pilus biogenesis protein	**0.05**	**-2.38**
PSPTO_3317	*PSYR_3151*	*gspE*, general secretion pathway protein E	**-0.36**	**1.36**
PSPTO_3315	*PSYR_3149*	*gspG*, general secretion pathway protein G	**-0.67**	**1.69**
PSPTO_3316	*PSYR_3150*	*gspF*,general secretion pathway protein F	**-0.69**	**1.34**
PSPTO_3314	*PSYR_3148*	*gspH*, general secretion pathway protein H	**-0.70**	**1.40**
PSPTO_3313	*PSYR_3147*	general secretion pathway protein I	**-0.93**	**1.09**
PSPTO_3312	*PSYR_3146*	general secretion pathway protein J	**-1.01**	**0.80**
PSPTO_3307	*PSYR_3141*	*gspD*, general secretion pathway protein D	**-1.18**	**0.11**
PSPTO_0811	*PSYR_0715*	pillin	**-1.29**	**0.50**
**Posttranslational modification, protein turnover, chaperones**				
PSPTO_1324	*PSYR_1140*	*dsbB*, disulfide oxidoreductase	**-0.13**	**1.17**
PSPTO_4210	*PSYR_3944*	*lon-2*, ATP-dependent protease La	**-1.02**	**0.40**
PSPTO_4940	*PSYR_0574*	*hflK*, hflK protein	**-1.15**	**0.09**
PSPTO_1688	*PSYR_3701*	thioredoxin	**-1.59**	**0.80**
**Energy production and conversion**				
PSPTO_3368	*PSYR_3200*	*nuoE*, NADH dehydrogenase subunit E	**0.50**	**-1.06**
PSPTO_4148	*PSYR_3887*	iron-sulfur cluster-binding protein	**0.13**	**-1.47**
PSPTO_2805	*PSYR_2533*	oxidoreductase, FAD-binding	**-0.12**	**1.02**

PSPTO_3364	*PSYR_3196*	*aceA*, isocitrate lyase	**-0.15**	**2.34**
PSPTO_0092	*PSYR_0227*	aldehyde dehydrogenase family protein	**-0.29**	**1.27**
PSPTO_5460	*PSYR_5013*	CAIB/BAIF family protein	**-0.32**	**1.84**
PSPTO_5231	*PSYR_0313*	oxidoreductase zinc-binding protein	**-0.71**	**1.00**
PSPTO_2288	*PSYR_2086*	*prpC*, 2-methylcitrate synthase	**-1.04**	**0.11**
PSPTO_3323	*PSYR_3157*	aldehyde dehydrogenase family protein	**-1.18**	**0.18**
PSPTO_2510	*PSYR_2314*	*poxB*, pyruvate dehydrogenase	**-1.36**	**0.73**
PSPTO_2697	*PSYR_2431*	oxidoreductase zinc-binding protein	**-1.38**	**0.13**
PSPTO_4256	*PSYR_1557*	NADH:flavin oxidoreductase/NADH oxidase family protein	**-1.42**	**0.31**
PSPTO_1489	*PSYR_1299*	xenobiotic reductase	**-1.62**	**0.33**
PSPTO_5072	*PSYR_0456*	cytochrome b561	**-1.94**	**1.35**
PSPTO_0062	*PSYR_0198*	citrate transporter	**-3.08**	**1.46**
**Carbohydrate transport and metabolism**				
PSPTO_4524	*PSYR_5112*	hypothetical protein PSPTO_4524	**1.82**	**-0.80**
PSPTO_1716	*PSYR_3673*	alpha-ribazole-5\'-phosphate phosphatase	**1.42**	**-1.39**
PSPTO_0959	*PSYR_0826*	*pgi*, glucose-6-phosphate isomerase	**0.26**	**-1.09**
PSPTO_3768	*PSYR_1712*	hypothetical protein PSPTO_3768	**-0.06**	**1.29**
PSPTO_2470	*PSYR_2235*	senescence marker protein-30 family protein	**-1.34**	**1.12**
PSPTO_5165	*PSYR_0374*	*glgP*, glycogen phosphorylase	**-1.41**	**0.49**
PSPTO_1345	*PSYR_0764*	*gnl*, gluconolactonase	**-1.48**	**0.19**
PSPTO_2638	*PSYR_2371*	*abP*, L-arabinose ABC transporter periplasmic L-arabinose-binding protein	**-1.48**	**0.08**
PSPTO_1292	*PSYR_1113*	glucose ABC transporter, periplasmic glucose-binding protein	**-1.77**	**1.04**
**Amino acid transport and metabolism**				
PSPTO_1481	*PSYR_1291*	*thrC*, threonine synthase	**1.43**	**-0.61**
PSPTO_3810	*PSYR_1669*	*metZ*, O-succinylhomoserine sulfhydrylase	**1.31**	**-0.07**
PSPTO_5500	*PSYR_5052*	sodium/alanine transporter	**1.19**	**-0.27**
PSPTO_4155	*PSYR_3892*	*argG*, argininosuccinate synthase	**1.10**	**-0.35**
PSPTO_5501	*PSYR_5053*	*ansA*, L-asparaginase I	**1.09**	**-0.29**
PSPTO_4165	*PSYR_3902*	ABC transporter ATP-binding protein	**1.02**	**-0.29**
PSPTO_5303	*PSYR_4863*	*potG*, putrescine ABC transporter ATP-binding protein	**0.73**	**-1.50**
PSPTO_1133	*PSYR_0973*	amino acid ABC transporter permease	**0.51**	**-1.44**
PSPTO_1444	*PSYR_1257*	*leuA*, 2-isopropylmalate synthase	**0.36**	**-1.31**
PSPTO_3806	*PSYR_1672*	*ilvA-1*, threonine dehydratase	**0.22**	**-1.05**
PSPTO_1134	*PSYR_0974*	amino acid ABC transporter substrate-	**0.16**	**-1.15**

		binding protein		
PSPTO_4885	*PSYR_4426*	branched-chain amino acid ABC transporter substrate-binding protein	**-0.16**	**2.13**
PSPTO_5248	*PSYR_0295*	amino acid ABC transporter ATP-binding protein	**-0.24**	**1.89**
PSPTO_0464	*PSYR_4709*	glycine betaine/L-proline ABC transporter, periplasmic substrate-binding protein	**-0.26**	**2.36**
PSPTO_0301	*PSYR_0090*	*gabT-2*, 4-aminobutyrate aminotransferase	**-0.34**	**1.08**
PSPTO_4577	*PSYR_4251*	glycine betaine/choline OpuC ABC transporter, periplasmic substrate-binding protein	**-1.19**	**0.33**
PSPTO_1633	*PSYR_3747*	*asnB*, asparagine synthetase	**-1.59**	**1.17**
PSPTO_1221	*PSYR_4795*	LysE family transporter	**-1.65**	**0.25**
PSPTO_4136	*PSYR_3874*	amino acid ABC transporter substrate-binding protein	**-1.71**	**0.71**
PSPTO_2775	*PSYR_2503*	amino acid ABC transporter substrate-binding protein	**-2.05**	**0.23**
**Nucleotide transport and metabolism**				
PSPTO_3663	*PSYR_1812*	*guaD,* guanine aminohydrolase	**-1.01**	**0.26**
**Coenzyme transport and metabolism**				
PSPTO_1713	*PSYR_3676*	*cobQ*, cobyric acid synthase	**1.82**	**-0.20**
PSPTO_1715	*PSYR_3674*	*cobT*, nicotinate-nucleotide--dimethylbenzimidazole phosphoribosyltransferase	**1.66**	**-1.08**
PSPTO_1714	*PSYR_3675*	*cobP*, cobinamide kinase/cobinamide phosphate guanylyltransferase	**1.56**	**-0.57**
PSPTO_1717	*PSYR_3672*	*cobS*, cobalamin (5\'-phosphate) synthase	**1.02**	**-1.04**
PSPTO_3553	*PSYR_3330*	*hppD*, 4-hydroxyphenylpyruvate dioxygenase	**-1.29**	**0.58**
PSPTO_2105	*PSYR_1900*	thiamine biosynthesis lipoprotein	**-3.50**	**0.69**
**Lipid transport and metabolism**				
PSPTO_0984	*PSYR_0849*	*pssA-1*, CDP-diacylglycerol--serine O-phosphatidyltransferase	**1.44**	**-0.20**
PSPTO_0496	*PSYR_4685*	*bioH,* bioH protein	**0.16**	**-1.5**
PSPTO_3457	*PSYR_3239*	short-chain fatty acid transporter	**-0.13**	**2.70**
PSPTO_2734	*PSYR_2465*	acetyltransferase family protein	**-0.45**	**1.70**
PSPTO_3527	*PSYR_3300*	hypothetical protein PSPTO_3527	**-0.46**	**1.49**
PSPTO_0744	*PSYR_0645*	acetyl-CoA acetyltransferase	**-0.84**	**1.34**
PSPTO_3503	*PSYR_3277*	cardiolipin synthetase 2	**-1.06**	**0.67**
PSPTO_4781	*PSYR_4330*	hypothetical protein PSPTO_4781	**-1.20**	**0.22**
PSPTO_0743	*PSYR_0644*	3-ketoacyl-(acyl-carrier-protein)	**-1.30**	**0.66**

		reductase		
**Inorganic ion transport and metabolism**				
PSPTO_0653	*PSYR_4521*	bacterioferritin	**1.70**	**-0.07**
PSPTO_0760	*PSYR_0664*	iron(III) dicitrate transport system, ATP-binding protein FecE	**1.30**	**-0.97**
PSPTO_2463	*PSYR_2229*	TonB-dependent siderophore receptor	**1.15**	**-0.33**
PSPTO_0761	*PSYR_0665*	*fecD*, iron(III) dicitrate transport system, permease protein FecD	**1.09**	**-0.34**
PSPTO_2635	*PSYR_2368*	cation ABC transporter substrate-binding protein	**1.04**	**-0.42**
PSPTO_3302	*PSYR_3132*	AcrB/AcrD/AcrF family protein	**0.61**	**-3.58**
PSPTO_3269	*PSYR_3106*	*pstS*, phosphate ABC transporter substrate-binding protein	**0.60**	**-1.27**
PSPTO_5301	*PSYR_4861*	*potI*, putrescine ABC transporter permease	**0.57**	**-2.29**
PSPTO_4432	*PSYR_4126*	*cysN/C*, bifunctional sulfate adenylyltransferase subunit 1/adenylylsulfate kinase protein	**0.48**	**-1.31**
PSPTO_5302	*PSYR_4862*	*potH*, putrescine ABC transporter permease	**0.45**	**-2.32**
PSPTO_5263	*PSYR_0280*	*katE*, catalase	**-0.27**	**1.06**
PSPTO_4533	*PSYR_4210*	*nhaA-1*, sodium-proton antiporter NhaA	**-0.39**	**1.33**
PSPTO_2973	*PSYR_2756*	*modA*, molybdate ABC transporter periplasmic molybdate-binding protein	**-0.79**	**1.49**
PSPTO_5255	*PSYR_0288*	*cynT*, carbonic anhydrase	**-1.02**	**0.03**
PSPTO_1930	*PSYR_3483*	*cynS*, cyanate lyase	**-1.65**	**0.49**
PSPTO_0110	*PSYR_0078*	metallo-beta-lactamase superfamily protein	**-1.70**	**0.30**
**Secondary metabolites biosynthesis, transport and catabolism**				
PSPTO_3332	*PSYR_3163*	alkaline metalloendoprotease	**1.08**	**-0.28**
PSPTO_3551	*PSYR_3326*	*hmgA*, homogentisate 1,2-dioxygenase	**-0.46**	**1.07**
PSPTO_5087	*PSYR_0442*	*mdcA*, malonate decarboxylase subunit alpha	**-2.21**	**0.19**
**Function unknown**				
PSPTO_5527	*PSYR_5076*	hypothetical protein PSPTO_5527	**1.76**	**-0.42**
PSPTO_0968	*PSYR_0095*	ISPsy6, transposase	**1.64**	**-0.22**
PSPTO_5526	*PSYR_5075*	cobalamin synthesis protein/P47K family protein	**1.62**	**-0.41**
PSPTO_2800	*PSYR_2528*	polysaccharide deacetylase family protein	**1.56**	**-0.24**
PSPTO_0795	*PSYR_0698*	membrane protein	**1.50**	**-0.35**
PSPTO_3569	*PSYR_3342*	hypothetical protein PSPTO_3569	**1.43**	**-1.78**
PSPTO_2999	*PSYR_2880*	hypothetical protein PSPTO_2999	**1.34**	**-0.91**

PSPTO_1590	*PSYR_3788*	hypothetical protein PSPTO_1590	**1.31**	**-1.45**
PSPTO_4545	*PSYR_4223*	hypothetical protein PSPTO_4545	**1.25**	**-0.68**
PSPTO_0978	*PSYR_0843*	hypothetical protein PSPTO_0978	**1.25**	**-0.16**
PSPTO_4431	*PSYR_4125*	hypothetical protein PSPTO_4431	**1.18**	**-0.31**
PSPTO_5451	*PSYR_5005*	hypothetical protein PSPTO_5451	**1.16**	**-0.11**
PSPTO_1722	*PSYR_3667*	hypothetical protein PSPTO_1722	**1.13**	**-0.68**
PSPTO_0747	*PSYR_0648*	transporter	**1.10**	**-1.06**
PSPTO_0403	*PSYR_4774*	hypothetical protein PSPTO_0403	**1.09**	**-0.39**
PSPTO_0988	*PSYR_0853*	hypothetical protein PSPTO_0988	**1.08**	**-0.26**
PSPTO_1433	*PSYR_1247*	hypothetical protein PSPTO_1433	**1.08**	**-0.64**
PSPTO_1589	*PSYR_3789*	lipoprotein	**1.06**	**-0.24**
PSPTO_4218	*PSYR_3952*	hypothetical protein PSPTO_4218	**1.04**	**-0.23**
PSPTO_4845	*PSYR_4385*	lipoprotein	**1.03**	**-2.09**
PSPTO_4133	*PSYR_3871*	penicillin amidase family protein	**1.02**	**-0.25**
PSPTO_2000	*PSYR_3417*	prevent-host-death family protein	**1.01**	**-0.15**
PSPTO_4848	*PSYR_4388*	response regulator	**0.84**	**-3.41**
PSPTO_3763	*PSYR_1717*	TPR domain-containing protein	**0.72**	**-1.24**
PSPTO_4988	*PSYR_0532*	hypothetical protein PSPTO_4988	**0.61**	**-1.64**
PSPTO_4989	*PSYR_0531*	hypothetical protein PSPTO_4989	**0.47**	**-1.31**
PSPTO_5410	*PSYR_4949*	hypothetical protein PSPTO_5410	**0.45**	**-1.81**
PSPTO_4990	*PSYR_0530*	hypothetical protein PSPTO_4990	**0.44**	**-1.11**
PSPTO_3481	*PSYR_4974*	hypothetical protein PSPTO_3481	**0.42**	**-2.01**
PSPTO_2784	*PSYR_2512*	hypothetical protein PSPTO_2784	**0.42**	**-1.77**
PSPTO_2615	*PSYR_2355*	GAF domain-containing protein	**0.33**	**-1.05**
PSPTO_5159	*PSYR_0380*	methyl-accepting chemotaxis protein	**0.31**	**-1.41**
PSPTO_3764	*PSYR_1716*	hypothetical protein PSPTO_3764	**0.30**	**-1.34**
PSPTO_3180	*PSYR_3046*	lipoprotein	**0.19**	**-1.16**
PSPTO_2939	*PSYR_2724*	hypothetical protein PSPTO_2939	**0.17**	**-1.32**
PSPTO_0715	*PSYR_0621*	hypothetical protein PSPTO_0715	**0.16**	**-1.08**
PSPTO_4163	*PSYR_3900*	hypothetical protein PSPTO_4163	**0.09**	**-1.63**
PSPTO_0499	*PSYR_4682*	hypothetical protein PSPTO_0499	**0.06**	**-1.86**
PSPTO_4381	*PSYR_4076*	hypothetical protein PSPTO_4381	**-0.12**	**1.85**
PSPTO_4780	*PSYR_4329*	hypothetical protein PSPTO_4780	**-0.13**	**1.21**
PSPTO_5229	*PSYR_0315*	hypothetical protein PSPTO_5229	**-0.15**	**1.03**
PSPTO_3479	*PSYR_3260*	hypothetical protein PSPTO_3479	**-0.19**	**1.10**
PSPTO_5351	*PSYR_4906*	lipoprotein	**-0.19**	**1.55**
PSPTO_2659	*PSYR_2393*	NAD(P)H-flavin oxidoreductase	**-0.26**	**1.57**
PSPTO_3689	*PSYR_1785*	hypothetical protein PSPTO_3689	**-0.27**	**1.35**
PSPTO_4237	*PSYR_3971*	lipoprotein	**-0.31**	**2.92**
PSPTO_0453	*PSYR_4722*	hypothetical protein PSPTO_0453	**-0.34**	**2.04**
PSPTO_4234	*PSYR_3968*	membrane protein TctA	**-0.36**	**1.79**
PSPTO_4233	*PSYR_3967*	membrane protein TctB	**-0.36**	**2.08**
PSPTO_0573	*PSYR_4599*	hypothetical protein PSPTO_0573	**-0.43**	**1.22**
PSPTO_2748	*PSYR_2477*	hypothetical protein PSPTO_2748	**-0.48**	**1.03**

PSPTO_5408	*PSYR_4947*	hypothetical protein PSPTO_5408	**-0.50**	**1.18**
PSPTO_0574	*PSYR_4598*	lipoprotein	**-0.52**	**1.46**
PSPTO_0274	*PSYR_0132*	hypothetical protein PSPTO_0274	**-0.53**	**1.13**
PSPTO_2042	*PSYR_1852*	hypothetical protein PSPTO_2042	**-0.59**	**1.43**
PSPTO_0575	*PSYR_4597*	hypothetical protein PSPTO_0575	**-0.61**	**1.98**
PSPTO_0579	*PSYR_4593*	sigma factor domain protein	**-0.62**	**1.09**
PSPTO_2051	*PSYR_1860*	ankyrin domain protein	**-0.66**	**1.41**
PSPTO_0578	*PSYR_4594*	hypothetical protein PSPTO_0578	**-0.67**	**1.07**
PSPTO_2059	*PSYR_1869*	hypothetical protein PSPTO_2059	**-0.75**	**1.21**
PSPTO_0576	*PSYR_4596*	hypothetical protein PSPTO_0576	**-0.84**	**1.36**
PSPTO_4232	*PSYR_3966*	TctC protein	**-0.87**	**2.87**
PSPTO_3192	*PSYR_3058*	amidase family protein	**-0.97**	**1.13**
PSPTO_3586	*PSYR_3357*	LysR family transcriptional regulator	**-1.01**	**0.62**
PSPTO_5378	*PSYR_0497*	LamB/YcsF family protein	**-1.01**	**0.26**
PSPTO_4657	*PSYR_4289*	zinc metallopeptidase	**-1.02**	**0.62**
PSPTO_0333	*PSYR_0262*	hypothetical protein PSPTO_0333	**-1.05**	**0.70**
PSPTO_0091	*PSYR_0226*	hypothetical protein PSPTO_0091	**-1.06**	**1.06**
PSPTO_1609	*PSYR_3770*	hypothetical protein PSPTO_1609	**-1.08**	**0.45**
PSPTO_2773	*PSYR_2501*	hypothetical protein PSPTO_2773	**-1.12**	**0.09**
PSPTO_2947	*PSYR_2731*	major facilitator family transporter	**-1.13**	**0.52**
PSPTO_3378	*PSYR_3210*	membrane protein	**-1.14**	**1.60**
PSPTO_3889	*PSYR_1595*	hypothetical protein PSPTO_3889	**-1.15**	**0.34**
PSPTO_0109	*PSYR_0079*	hypothetical protein PSPTO_0109	**-1.17**	**0.33**
PSPTO_3605	*PSYR_3376*	lyase	**-1.18**	**0.65**
PSPTO_3700	*PSYR_1775*	oxidoreductase, aldo/keto reductase family	**-1.29**	**0.15**
PSPTO_4289	*PSYR_3992*	hypothetical protein PSPTO_4289	**-1.31**	**0.25**
PSPTO_3174	*PSYR_3039*	hypothetical protein PSPTO_3174	**-1.31**	**0.11**
PSPTO_2506	*PSYR_2310*	lipoprotein	**-1.31**	**0.58**
PSPTO_4546	*PSYR_4224*	hypothetical protein PSPTO_4546	**-1.37**	**0.35**
PSPTO_4103	*PSYR_3841*	hypothetical protein PSPTO_4103	**-1.38**	**0.37**
PSPTO_1035	*PSYR_0883*	colicin/pyosin nuclease family protein	**-1.42**	**0.22**
PSPTO_4602	*PSYR_4264*	putative ABC transporter ATP-binding protein	**-1.44**	**0.21**
PSPTO_4673	*PSYR_4305*	hypothetical protein PSPTO_4673	**-1.48**	**0.09**
PSPTO_0142	*PSYR_0048*	hypothetical protein PSPTO_0142	**-1.51**	**0.89**
PSPTO_5053	*PSYR_0470*	hypothetical protein PSPTO_5053	**-1.52**	**0.16**
PSPTO_2695	*PSYR_2429*	hypothetical protein PSPTO_2695	**-1.58**	**0.34**
PSPTO_1596	*PSYR_3782*	hypothetical protein PSPTO_1596	**-1.64**	**0.86**
PSPTO_1451	*PSYR_0753*	hypothetical protein PSPTO_1451	**-1.65**	**0.31**
PSPTO_1931	*PSYR_3482*	hypothetical protein PSPTO_1931	**-1.75**	**0.12**
PSPTO_3548	*PSYR_3323*	hypothetical protein PSPTO_3548	**-1.78**	**0.17**
PSPTO_1485	*PSYR_1295*	hypothetical protein PSPTO_1485	**-1.81**	**0.90**
PSPTO_1507	*PSYR_1317*	hypothetical protein PSPTO_1507	**-1.82**	**0.26**

PSPTO_2471	*PSYR_2236*	membrane protein	**-1.83**	**0.40**
PSPTO_0156	*PSYR_0036*	hypothetical protein PSPTO_0156	**-1.91**	**0.16**
PSPTO_5071	*PSYR_0457*	hypothetical protein PSPTO_5071	**-1.94**	**1.34**
PSPTO_0154	*PSYR_0038*	hypothetical protein PSPTO_0154	**-2.00**	**0.67**
PSPTO_1344	*PSYR_0765*	hypothetical protein PSPTO_1344	**-2.00**	**0.44**
PSPTO_2952	*PSYR_2736*	glycosidase	**-2.17**	**0.29**
PSPTO_5355	*PSYR_4908*	hypothetical protein PSPTO_5355	**-2.68**	**0.47**
PSPTO_1371	*PSYR_1182*	effector locus protein	**-3.84**	**0.44**
