# Supplementary material for: Comparative transcriptomic analysis of global gene expression mediated by (p) ppGpp reveals common regulatory networks in Pseudomonas syringae
Source: BMC Genomics. 2020 Apr 10;21:296. doi: 10.1186/s12864-020-6701-2 (PMC7146990; doi:10.1186/s12864-020-6701-2)
Supplement: Supplementary file 4 — Additional file 4: Table S6. List of unique genes in PstDC3000 regulated by (p)ppGpp. Table S7. List of unique genes of PssB728a regulated by (p)ppGpp. [file 12864_2020_6701_MOESM4_ESM.pdf]

Table S6. List of unique genes in *Pst*DC3000 regulated by (p)ppGpp with |log_2FC| value ≥1 and an adjusted p-value <0.05 in (p)ppGpp^0$_{PstDC3000}$ than *Pst*DC3000.

Locus tag	**Gene description**	**(p)ppGpp^0$_{PstDC3000}$ /*Pst*DC3000**
**Type III secretion system**		
PSPTO_4594	type III effector HopO1-2	**-1.01**
PSPTO_4592	type III effector HopO1-3	**-1.13**
PSPTO_3292	type III effector HopAH2-1	**-1.14**
PSPTO_0906	type III effector HopAI1	**-1.18**
PSPTO_4593	type III effector HopT1-2	**-1.36**
PSPTO_0474	type III effector HopAS1, partial	**-1.41**
PSPTO_4724	type III effector HopD	**-2.33**
PSPTO_4703	type III effector HopAQ1	**-2.98**
PSPTO_0501	type III effector HopU1	**-3.09**
PSPTO_1387	type III secretion protein HrpF	**-3.50**
PSPTO_0852	type III helper protein HopAJ1	**-3.58**
PSPTO_0883	type III effector HopR1	**-3.60**
PSPTO_0503	type III chaperone protein ShcF	**-3.82**
PSPTO_0061	type III effector HopY1	**-3.85**
PSPTO_4599	type III chaperone ShcS1	**-3.87**
PSPTO_4718	type III effector HopAA1-2	**-3.87**
PSPTO_0877	type III effector HopQ1-1	**-3.87**
PSPTO_4691	type III effector HopAD1	**-3.99**
PSPTO_0589	type III effector HopC1	**-4.07**
PSPTO_5353	type III chaperone protein ShcA	**-4.07**
PSPTO_4722	type III effector HopAO1	**-4.17**
PSPTO_0502	type III effector HopF2	**-4.19**
PSPTO_4589	type III chaperone ShcS2	**-4.31**
PSPTO_1369	type III chaperone protein ShcN	**-4.38**
PSPTO_4597	type III effector HopS1	**-4.38**
PSPTO_1022	type III effector HopAM1-1	**-4.51**
PSPTO_2678	type III helper protein HopP1	**-4.52**
PSPTO_4720	type III effector HopV1	**-4.62**
PSPTO_4588	type III effector HopS2	**-4.63**
PSPTO_1381	type III helper protein HrpA1	**-4.67**
PSPTO_5354	type III effector HopA1	**-4.73**
PSPTO_1370	type III effector HopN1	**-4.82**
PSPTO_1406	type III effector HopB1	**-4.96**
PSPTO_0876	type III effector HopD1	**-5.11**
PSPTO_4721	type III chaperone ShcV	**-5.11**
PSPTO_4331	type III effector HopE1	**-5.68**
PSPTO_4727	type III effector HopG1	**-5.73**
**Translation, ribosomal structure and biogenesis**		
PSPTO_5387	stability cassette protein	**-1.12**

PSPTO_0836	hypothetical protein PSPTO_0836	**-3.58**
**Transcription**		
PSPTO_2444	GNAT family acetyltransferase	**1.96**
PSPTO_3021	LysR family transcriptional regulator	**1.86**
PSPTO_1857	AraC family transcriptional regulator	**1.78**
PSPTO_4755	hypothetical protein PSPTO_4755	**1.77**
PSPTO_3046	sigma-54 dependent transcriptional regulator	**1.34**
PSPTO_4523	AsnC family transcriptional regulator	**1.33**
PSPTO_4010	repressor protein cI	**1.24**
PSPTO_0212	DNA-binding transcriptional regulator LysR	**1.09**
PSPTO_3017	LacI family transcriptional regulator	**1.07**
PSPTO_2549	sigma-54 dependent transcriptional regulator	**-1.04**
PSPTO_4262	TetR family transcriptional regulator	**-1.15**
PSPTO_4528	AraC family transcriptional regulator	**-1.17**
PSPTO_3056	AraC family transcriptional regulator	**-1.26**
PSPTO_0032	hypothetical protein PSPTO_0032	**-1.45**
PSPTO_5424	sigma-54 dependent transcriptional regulator	**-1.74**
PSPTO_0033	ParB family protein	**-2.74**
PSPTO_3086	transcriptional regulator	**-4.13**
**Replication, recombination and repair**		
PSPTO_3220	ISPsy5, transposase	**1.48**
PSPTO_1418	ISPsy6, transposase	**1.27**
PSPTO_4735	ATP-dependent helicase HrpB	**1.23**
PSPTO_5443	ISPsy5, transposase	**1.21**
PSPTO_1439	ISPsy6, transposase	**1.21**
PSPTO_3221	ISPsy5, Orf1	**1.09**
PSPTO_3734	ISPsy6, transposase	**1.06**
PSPTO_4751	UvrD/REP helicase family protein	**-1.01**
PSPTO_4748	site-specific recombinase, phage integrase family	**-1.07**
PSPTO_2857	site-specific recombinase, phage integrase family	**-1.09**
PSPTO_5629	insertion sequence	**-1.17**
PSPTO_2860	helicase domain-containing protein	**-1.19**
PSPTO_2856	site-specific recombinase, phage integrase family	**-1.33**
PSPTO_1407	ISPssy transposase or derivative	**-1.37**
PSPTO_0028	transposase	**-1.46**
PSPTO_0587	site-specific recombinase, phage integrase family	**-1.48**
PSPTO_2388	ISPsy4, transposition helper protein	**-1.49**
PSPTO_0037	helicase domain-containing protein	**-1.51**
PSPTO_3326	group II intron, maturase	**-1.58**
PSPTO_4604	site-specific recombinase, phage integrase family	**-1.59**
PSPTO_0047	UvrD/REP helicase family protein	**-1.76**
PSPTO_3930	retron reverse transcriptase	**-2.15**
**Cell cycle control, cell division, chromosome partitioning**		
PSPTO_0855	ParA family protein	**1.22**

PSPTO_5387	stability cassette protein	**-1.12**
**Defense mechanisms**		
PSPTO_1089	type I restriction-modification enzyme, R subunit	**-1.03**
PSPTO_0005	type I restriction-modification system, M subunit	**-1.06**
PSPTO_0008	HsdR family type I site-specific deoxyribonuclease	**-1.12**
PSPTO_1087	type I restriction-modification system subunit S	**-1.16**
PSPTO_0285	hypothetical protein PSPTO_0285	**-1.43**
PSPTO_0011	hypothetical protein PSPTO_0011	**-1.47**
PSPTO_0006	type I restriction-modification system, S subunit, EcoA family	**-1.47**
PSPTO_2428	multidrug resistance protein NorM	**-1.51**
PSPTO_0370	MATE efflux family protein	**-1.55**
PSPTO_2603	ABC transporter ATP-binding protein/permease	**-2.54**
PSPTO_2604	ABC transporter ATP-binding protein/permease	**-2.57**
**Signal transduction mechanisms**		
PSPTO_0897	LuxR family DNA-binding response regulator	**1.29**
PSPTO_3566	carbon storage regulator	**1.24**
PSPTO_4784	diguanylate cyclase	**-1.34**
PSPTO_4079	sensor histidine kinase/response regulator	**-1.58**
PSPTO_5416	serine/threonine protein kinase	**-1.71**
PSPTO_4705	sensor histidine kinase CorS	**-1.88**
PSPTO_5422	FHA domain-containing protein	**-2.33**
PSPTO_4704	DNA-binding response regulator CorR	**-2.68**
PSPTO_5417	serine/threonine phosphoprotein phosphatase	**-2.90**
PSPTO_4080	LuxR family DNA-binding response regulator	**-3.07**
**Cell wall/membrane/envelope biogenesis**		
PSPTO_1067	glycosyl transferase family protein	**1.29**
PSPTO_2554	hypothetical protein PSPTO_2554	**-1.05**
PSPTO_4385	Rhs element Vgr protein	**-1.33**
PSPTO_1071	glycosyl transferase family protein	**-1.42**
PSPTO_3290	outer membrane porin, OprD family	**-1.76**
PSPTO_5415	Rhs element Vgr protein	**-1.86**
PSPTO_3238	tonB protein	**-1.91**
PSPTO_1918	glycosyl transferase family protein	**-2.27**
PSPTO_5418	hypothetical protein PSPTO_5418	**-2.93**
**Cell motility**		
PSPTO_3237	methyl-accepting chemotaxis protein	**-1.02**
**Intracellular trafficking, secretion, and vesicular transport**		
PSPTO_3975	tolQ protein	**1.67**
PSPTO_3229	filamentous hemagglutinin, intein-containing	**-1.59**
**Posttranslational modification, protein turnover, chaperones**		
PSPTO_2548	clpB protein	**-1.01**
PSPTO_4295	DnaJ domain-containing protein	**-1.47**
PSPTO_4254	glutathione reductase	**-1.50**
PSPTO_3560	GDA1/CD39 family protein	**-1.62**

PSPTO_4260	thioredoxin	**-1.73**
**Energy production and conversion**		
PSPTO_2435	oxidoreductase, FAD-binding	**-1.07**
PSPTO_3075	oxidoreductase, aldo/keto reductase family	**-1.13**
PSPTO_4689	crotonyl-CoA reductase	**-1.33**
PSPTO_2861	4Fe-4S binding protein, partial	**-1.37**
PSPTO_3559	malate synthase G	**-1.45**
PSPTO_3064	aldehyde dehydrogenase family protein	**-1.89**
PSPTO_0834	alcohol dehydrogenase	**-3.78**
**Carbohydrate transport and metabolism**		
PSPTO_2926	multidrug transporter	**-1.04**
PSPTO_2401	transketolase	**-1.07**
PSPTO_2400	ribose ABC transporter permease	**-1.46**
PSPTO_3560	GDA1/CD39 family protein	**-1.62**
PSPTO_0838	major facilitator family transporter	**-1.95**
PSPTO_3018	hypothetical protein PSPTO_3018	**-2.36**
PSPTO_2399	ribose ABC transporter periplasmic ribose-binding protein	**-2.43**
PSPTO_0202	membrane protein	**-2.61**
PSPTO_2601	membrane protein	**-3.51**
**Amino acid transport and metabolism**		
PSPTO_0280	methionine aminopeptidase	**1.18**
PSPTO_3060	glycine betaine/L-proline ABC transporter ATP-binding protein	**-1.06**
PSPTO_3059	glycine betaine/L-proline ABC transporter permease	**-1.07**
PSPTO_2988	branched-chain amino acid ABC transporter permease	**-1.10**
PSPTO_2427	serine hydroxymethyltransferase	**-1.14**
PSPTO_2915	glutamine ABC transporter, permease protein	**-1.17**
PSPTO_3058	glycine betaine transporter periplasmic subunit	**-1.19**
PSPTO_2913	glutamine ABC transporter ATP-binding protein	**-1.28**
PSPTO_3252	dipeptide ABC transporter ATP-binding protein	**-1.43**
PSPTO_0524	peptidase, M20/M25/M40 family	**-1.43**
PSPTO_3251	dipeptide ABC transporter ATP-binding protein	**-1.49**
PSPTO_2504	efflux protein, LysE family	**-1.51**
PSPTO_0203	cysteine synthase	**-1.78**
PSPTO_3063	dihydrodipicolinate synthase	**-1.85**
PSPTO_2430	pyridoxal-phosphate dependent enzyme family protein	**-3.28**
PSPTO_0873	amidinotransferase family protein	**-4.85**
**Nucleotide transport and metabolism**		
PSPTO_3043	5\'-nucleotidase	**-1.05**
PSPTO_0043	cytidine/deoxycytidylate deaminase family protein	**-1.71**
PSPTO_2862	oxidoreductase, molybdopterin-binding subunit	**-1.82**
**Coenzyme transport and metabolism**		
PSPTO_2595	isochorismate synthase	**-2.90**
PSPTO_0835	ribD C-terminal domain protein, partial	**-3.50**

**Lipid transport and metabolism**		
PSPTO_0893	outer membrane protein P1	**1.75**
PSPTO_1766	lipase	**1.52**
PSPTO_2823	hypothetical protein PSPTO_2823	**1.09**
PSPTO_4683	coronafacic acid beta-ketoacyl synthetase component	**-1.01**
PSPTO_4277	esterase/lipase/thioesterase family protein	**-1.02**
PSPTO_0200	hypothetical protein PSPTO_0200	**-3.10**
**Inorganic ion transport and metabolism**		
PSPTO_2846	TonB-dependent siderophore receptor	**-1.02**
PSPTO_1855	TonB-dependent receptor	**-1.04**
PSPTO_1204	regulatory protein	**-1.05**
PSPTO_3250	dipeptide ABC transporter, permease protein DppC	**-1.08**
PSPTO_2605	TonB-dependent siderophore receptor	**-1.13**
PSPTO_1206	TonB-dependent siderophore receptor	**-1.18**
PSPTO_4259	glutathione-regulated potassium-efflux system protein	**-1.93**
PSPTO_2398	ribose ABC transporter ATP-binding protein	**-2.26**
**Secondary metabolites biosynthesis, transport and catabolism**		
PSPTO_1859	isothiocyanate resistance protein SaxB; isochorismatase family	**-1.01**
PSPTO_4683	coronafacic acid beta-ketoacyl synthetase component	**-1.01**
PSPTO_4257	4-oxalocrotonate tautomerase	**-1.02**
PSPTO_4709	coronamic acid synthetase CmaA	**-1.04**
PSPTO_3554	maleylacetoacetate isomerase	**-1.21**
PSPTO_4520	methyltransferase domain protein	**-1.25**
PSPTO_3051	2,4\'-dihydroxyacetophenone dioxygenase	**-1.27**
PSPTO_4261	isomerase	**-1.50**
PSPTO_4712	coronamic acid synthetase, thioesterase component	**-1.76**
PSPTO_2597	yersiniabactin synthetase, salycilate ligase component	**-3.22**
PSPTO_2602	yersiniabactin non-ribosomal peptide synthetase	**-3.32**
PSPTO_2596	isochorismate pyruvate-lyase	**-3.40**
PSPTO_2429	capK domain protein	**-3.60**
PSPTO_2600	yersiniabactin polyketide/non-ribosomal peptide synthetase	**-3.63**
PSPTO_2598	yersiniabactin synthetase, thioesterase component	**-3.82**
PSPTO_2599	yersiniabactin synthetase, thiazolinyl reductase component	**-4.11**
**Function unknown**		
PSPTO_0803	hypothetical protein PSPTO_0803	**2.74**
PSPTO_3896	hypothetical protein PSPTO_3896	**2.40**
PSPTO_3067	hypothetical protein PSPTO_3067	**2.34**
PSPTO_1816	hypothetical protein PSPTO_1816	**2.32**
PSPTO_1595	hypothetical protein PSPTO_1595	**2.20**
PSPTO_4007	hypothetical protein PSPTO_4007	**1.98**
PSPTO_4009	regulatory protein Cro	**1.97**
PSPTO_2356	hypothetical protein PSPTO_2356	**1.95**
PSPTO_0792	hypothetical protein PSPTO_0792	**1.94**
PSPTO_4008	hypothetical protein PSPTO_4008	**1.93**

PSPTO_5059	hypothetical protein PSPTO_5059	**1.92**
PSPTO_3419	C4-type zinc finger protein, DksA/TraR family	**1.67**
PSPTO_4017	hypothetical protein PSPTO_4017	**1.65**
PSPTO_3974	tolR protein	**1.62**
PSPTO_4573	hypothetical protein PSPTO_4573	**1.56**
PSPTO_3420	hypothetical protein PSPTO_3420	**1.52**
PSPTO_0896	sensor histidine kinase/response regulator	**1.48**
PSPTO_0342	hypothetical protein PSPTO_0342	**1.46**
PSPTO_4016	hypothetical protein PSPTO_4016	**1.39**
PSPTO_2648	hypothetical protein PSPTO_2648	**1.37**
PSPTO_4792	ISPsy6, transposase	**1.30**
PSPTO_0206	hypothetical protein PSPTO_0206	**1.29**
PSPTO_3303	hypothetical protein PSPTO_3303	**1.28**
PSPTO_4994	ISPsy5, transposase	**1.26**
PSPTO_0521	DNA-binding protein	**1.26**
PSPTO_4251	ISPsy5, transposase	**1.25**
PSPTO_3996	ISPsy5, transposase	**1.24**
PSPTO_5148	polyhydroxyalkanoate granule-associated protein PhaI	**1.22**
PSPTO_4737	ISPsy5, transposase	**1.22**
PSPTO_4693	ISPsy5, transposase	**1.22**
PSPTO_1189	ISPsy5, transposase	**1.21**
PSPTO_4118	hypothetical protein PSPTO_4118	**1.20**
PSPTO_0196	ISPsy5, transposase	**1.20**
PSPTO_1175	membrane protein	**1.20**
PSPTO_0039	ISPsy5, transposase	**1.19**
PSPTO_2460	ISPsy5, transposase	**1.19**
PSPTO_5543	ISPsy5, transposase	**1.19**
PSPTO_4764	ISPsy5, transposase	**1.19**
PSPTO_5411	ISPsy5, transposase	**1.19**
PSPTO_4389	ISPsy5, transposase	**1.19**
PSPTO_4766	hypothetical protein PSPTO_4766	**1.19**
PSPTO_3651	ISPsy5, transposase	**1.18**
PSPTO_3415	hypothetical protein PSPTO_3415	**1.18**
PSPTO_5212	ISPsy5, transposase	**1.18**
PSPTO_3390	tail protein D	**1.17**
PSPTO_4060	ISPsy6, transposase	**1.16**
PSPTO_1938	hypothetical protein PSPTO_1938	**1.15**
PSPTO_2204	ISPsy6, transposase	**1.15**
PSPTO_1098	ISPsy5, transposase	**1.14**
PSPTO_1477	ISPsy6, transposase	**1.14**
PSPTO_3416	holin	**1.13**
PSPTO_3414	hypothetical protein PSPTO_3414	**1.13**
PSPTO_3236	hypothetical protein PSPTO_3236	**1.12**
PSPTO_3808	ISPsy6, transposase	**1.09**

PSPTO_1854	hypothetical protein PSPTO_1854	**1.08**
PSPTO_0670	ISPsy5, transposase	**1.07**
PSPTO_5591	ISPsy5, transposase	**1.05**
PSPTO_1227	ISPsy5, transposase	**1.04**
PSPTO_2971	ISPsy5, transposase	**1.03**
PSPTO_3999	ISPsy5, transposase	**1.02**
PSPTO_2067	hypothetical protein PSPTO_2067	**1.02**
PSPTO_3407	hypothetical protein PSPTO_3407	**1.01**
PSPTO_4567	ISPsy5, transposase	**1.01**
PSPTO_5445	ISPsy5, transposase	**1.01**
PSPTO_0832	ISPsy4, transposition helper protein	**-1.00**
PSPTO_0023	hypothetical protein PSPTO_0023	**-1.01**
PSPTO_2075	hypothetical protein PSPTO_2075	**-1.02**
PSPTO_0193	ISPsy4, transposition helper protein	**-1.02**
PSPTO_0049	hypothetical protein PSPTO_0049	**-1.02**
PSPTO_4532	hypothetical protein PSPTO_4532	**-1.02**
PSPTO_3092	hypothetical protein PSPTO_3092	**-1.03**
PSPTO_2741	ISPsy4, transposition helper protein	**-1.05**
PSPTO_5620	hypothetical protein PSPTO_5620	**-1.06**
PSPTO_2924	hypothetical protein PSPTO_2924	**-1.07**
PSPTO_0172	hypothetical protein PSPTO_0172	**-1.07**
PSPTO_2086	hypothetical protein PSPTO_2086	**-1.07**
PSPTO_5211	hypothetical protein PSPTO_5211	**-1.07**
PSPTO_0013	hypothetical protein PSPTO_0013	**-1.07**
PSPTO_4750	hypothetical protein PSPTO_4750	**-1.07**
PSPTO_0012	hypothetical protein PSPTO_0012	**-1.08**
PSPTO_5208	hypothetical protein PSPTO_5208	**-1.08**
PSPTO_4327	hypothetical protein PSPTO_4327	**-1.09**
PSPTO_2536	hypothetical protein PSPTO_2536	**-1.09**
PSPTO_3995	abortive infection protein, internal deletion	**-1.11**
PSPTO_2888	hypothetical protein PSPTO_2888	**-1.11**
PSPTO_4761	hypothetical protein PSPTO_4761	**-1.11**
PSPTO_2091	hypothetical protein PSPTO_2091	**-1.12**
PSPTO_1657	hypothetical protein PSPTO_1657	**-1.12**
PSPTO_3055	hypothetical protein PSPTO_3055	**-1.13**
PSPTO_5630	hypothetical protein PSPTO_5630	**-1.13**
PSPTO_4723	hypothetical protein PSPTO_4723	**-1.13**
PSPTO_1603	hypothetical protein PSPTO_1603	**-1.14**
PSPTO_3065	hypothetical protein PSPTO_3065	**-1.15**
PSPTO_3947	hypothetical protein PSPTO_3947	**-1.17**
PSPTO_2318	hypothetical protein PSPTO_2318	**-1.17**
PSPTO_3041	membrane protein	**-1.18**
PSPTO_4348	hypothetical protein PSPTO_4348	**-1.18**
PSPTO_0525	lipoprotein	**-1.19**

PSPTO_0027	hypothetical protein PSPTO_0027	**-1.19**
PSPTO_4609	hypothetical protein PSPTO_4609	**-1.20**
PSPTO_4040	hypothetical protein PSPTO_4040	**-1.21**
PSPTO_4797	hypothetical protein PSPTO_4797	**-1.21**
PSPTO_2631	hypothetical protein PSPTO_2631	**-1.21**
PSPTO_4714	cmaU protein	**-1.23**
PSPTO_3253	hypothetical protein PSPTO_3253	**-1.25**
PSPTO_0297	hypothetical protein PSPTO_0297	**-1.25**
PSPTO_1094	hypothetical protein PSPTO_1094	**-1.26**
PSPTO_1364	hypothetical protein PSPTO_1364	**-1.26**
PSPTO_3054	short chain dehydrogenase/reductase family oxidoreductase	**-1.27**
PSPTO_0477	hypothetical protein PSPTO_0477	**-1.27**
PSPTO_1367	GNAT family acetyltransferase	**-1.30**
PSPTO_1082	hypothetical protein PSPTO_1082	**-1.32**
PSPTO_3061	LysR family transcriptional regulator	**-1.33**
PSPTO_3337	hypothetical protein PSPTO_3337	**-1.34**
PSPTO_3426	hypothetical protein PSPTO_3426	**-1.35**
PSPTO_2165	group II intron, maturase	**-1.36**
PSPTO_3057	MmgE/PrpD family protein	**-1.36**
PSPTO_1060	hypothetical protein PSPTO_1060	**-1.37**
PSPTO_5435	secreted protein Hcp	**-1.37**
PSPTO_1069	membrane protein	**-1.37**
PSPTO_4772	hypothetical protein PSPTO_4772	**-1.38**
PSPTO_5633	hypothetical protein PSPTO_5633	**-1.38**
PSPTO_2977	hypothetical protein PSPTO_2977	**-1.39**
PSPTO_0030	hypothetical protein PSPTO_0030	**-1.39**
PSPTO_1070	lipopolysaccharide biosynthesis protein	**-1.39**
PSPTO_0007	hypothetical protein PSPTO_0007	**-1.40**
PSPTO_4967	hypothetical protein PSPTO_4967	**-1.40**
PSPTO_4067	short-chain dehydrogenase/reductase family oxidoreductase	**-1.40**
PSPTO_1571	hypothetical protein PSPTO_1571	**-1.40**
PSPTO_0015	hypothetical protein PSPTO_0015	**-1.40**
PSPTO_1073	membrane protein	**-1.41**
PSPTO_0031	Ser/Thr protein phosphatase family protein	**-1.43**
PSPTO_4014	ISPsy4, transposition helper protein	**-1.43**
PSPTO_4716	hypothetical protein PSPTO_4716	**-1.43**
PSPTO_2912	glutamine ABC transporter, periplasmic amino acid-binding protein	**-1.44**
PSPTO_1092	mobilization protein MobB	**-1.44**
PSPTO_2322	ISPsy4, transposition helper protein	**-1.45**
PSPTO_2539	secreted protein Hcp	**-1.45**
PSPTO_5631	hypothetical protein PSPTO_5631	**-1.45**
PSPTO_1900	hypothetical protein PSPTO_1900	**-1.45**
PSPTO_2859	hypothetical protein PSPTO_2859	**-1.45**

PSPTO_2085	hypothetical protein PSPTO_2085	**-1.45**
PSPTO_5576	ISPsy4, transposition helper protein	**-1.45**
PSPTO_2553	hypothetical protein PSPTO_2553	**-1.46**
PSPTO_1096	ISPsy4, transposition helper protein	**-1.48**
PSPTO_2942	hypothetical protein PSPTO_2942	**-1.50**
PSPTO_5203	hypothetical protein PSPTO_5203	**-1.50**
PSPTO_3225	ISPsy4, transposition helper protein	**-1.50**
PSPTO_3014	ISPsy4, transposition helper protein	**-1.50**
PSPTO_2537	hypothetical protein PSPTO_2537	**-1.51**
PSPTO_5370	ISPsy4, transposition helper protein	**-1.52**
PSPTO_3946	hypothetical protein PSPTO_3946	**-1.52**
PSPTO_1259	hypothetical protein PSPTO_1259	**-1.53**
PSPTO_0016	hypothetical protein PSPTO_0016	**-1.53**
PSPTO_4626	ISPsy4, transposition helper protein	**-1.53**
PSPTO_4258	NAD(P)H dehydrogenase, quinone family	**-1.54**
PSPTO_0528	hypothetical protein PSPTO_0528	**-1.54**
PSPTO_0056	ISPsy4, transposition helper protein	**-1.55**
PSPTO_3050	AraC family transcriptional regulator	**-1.55**
PSPTO_4270	ISPsy4, transposition helper protein	**-1.58**
PSPTO_5616	hypothetical protein PSPTO_5616	**-1.58**
PSPTO_4039	hypothetical protein PSPTO_4039	**-1.58**
PSPTO_1655	hypothetical protein PSPTO_1655	**-1.59**
PSPTO_2320	hypothetical protein PSPTO_2320	**-1.60**
PSPTO_4711	coronamic acid synthetase CmaC	**-1.60**
PSPTO_5210	EF hand domain-containing protein	**-1.61**
PSPTO_3485	lipase family protein	**-1.62**
PSPTO_0295	hypothetical protein PSPTO_0295	**-1.63**
PSPTO_5373	hypothetical protein PSPTO_5373	**-1.63**
PSPTO_2013	hypothetical protein PSPTO_2013	**-1.64**
PSPTO_2084	hypothetical protein PSPTO_2084	**-1.66**
PSPTO_2137	MbtH-like protein	**-1.66**
PSPTO_3938	hypothetical protein PSPTO_3938	**-1.71**
PSPTO_4717	hypothetical protein PSPTO_4717	**-1.71**
PSPTO_4676	hypothetical protein PSPTO_4676	**-1.71**
PSPTO_5206	EF hand domain-containing protein	**-1.74**
PSPTO_4741	hypothetical protein PSPTO_4741	**-1.74**
PSPTO_4326	hypothetical protein PSPTO_4326	**-1.77**
PSPTO_3728	hypothetical protein PSPTO_3728	**-1.78**
PSPTO_2396	short chain dehydrogenase/reductase family oxidoreductase	**-1.79**
PSPTO_5073	hypothetical protein PSPTO_5073	**-1.79**
PSPTO_4743	hypothetical protein PSPTO_4743	**-1.81**
PSPTO_4747	hypothetical protein PSPTO_4747	**-1.82**
PSPTO_5204	EF hand domain-containing protein	**-1.82**
PSPTO_5430	hypothetical protein PSPTO_5430	**-1.83**

PSPTO_4324	hypothetical protein PSPTO_4324	**-1.84**
PSPTO_3291	methyl-accepting chemotaxis protein	**-1.85**
PSPTO_4713	alanyl tRNA synthetase-related protein	**-1.88**
PSPTO_3218	hypothetical protein PSPTO_3218	**-1.88**
PSPTO_5207	hypothetical protein PSPTO_5207	**-1.92**
PSPTO_2550	hypothetical protein PSPTO_2550	**-1.96**
PSPTO_1093	relaxase/mobilization nuclease domain protein	**-1.97**
PSPTO_2397	short chain dehydrogenase	**-1.99**
PSPTO_1850	hypothetical protein PSPTO_1850	**-1.99**
PSPTO_3019	oxidoreductase, Gfo/Idh/MocA family	**-2.00**
PSPTO_3228	hypothetical protein PSPTO_3228	**-2.01**
PSPTO_2083	hypothetical protein PSPTO_2083	**-2.03**
PSPTO_3937	hypothetical protein PSPTO_3937	**-2.04**
PSPTO_5426	hypothetical protein PSPTO_5426	**-2.04**
PSPTO_1967	hypothetical protein PSPTO_1967	**-2.05**
PSPTO_4754	hypothetical protein PSPTO_4754	**-2.07**
PSPTO_0038	hypothetical protein PSPTO_0038	**-2.08**
PSPTO_5423	hypothetical protein PSPTO_5423	**-2.09**
PSPTO_2641	lipoprotein	**-2.09**
PSPTO_5414	lipoprotein	**-2.10**
PSPTO_1097	membrane protein	**-2.10**
PSPTO_4325	hypothetical protein PSPTO_4325	**-2.13**
PSPTO_4746	site-specific recombinase, phage integrase family domain protein	**-2.16**
PSPTO_2390	hypothetical protein PSPTO_2390	**-2.17**
PSPTO_4634	N-acetylmuramoyl-L-alanine amidase	**-2.17**
PSPTO_0020	hypothetical protein PSPTO_0020	**-2.17**
PSPTO_0029	transposition helper protein	**-2.21**
PSPTO_5427	hypothetical protein PSPTO_5427	**-2.24**
PSPTO_4742	site-specific recombinase, phage integrase family	**-2.24**
PSPTO_4321	hypothetical protein PSPTO_4321	**-2.28**
PSPTO_4733	hypothetical protein PSPTO_4733	**-2.28**
PSPTO_5205	hypothetical protein PSPTO_5205	**-2.30**
PSPTO_0021	hypothetical protein PSPTO_0021	**-2.31**
PSPTO_5365	hypothetical protein PSPTO_5365	**-2.36**
PSPTO_5364	hypothetical protein PSPTO_5364	**-2.37**
PSPTO_2431	hypothetical protein PSPTO_2431	**-2.38**
PSPTO_4607	hypothetical protein PSPTO_4607	**-2.43**
PSPTO_1297	hypothetical protein PSPTO_1297	**-2.44**
PSPTO_4627	hypothetical protein PSPTO_4627	**-2.45**
PSPTO_2458	hypothetical protein PSPTO_2458	**-2.48**
PSPTO_0871	macrolide efflux protein	**-2.51**
PSPTO_1090	hypothetical protein PSPTO_1090	**-2.54**
PSPTO_5420	hypothetical protein PSPTO_5420	**-2.56**

PSPTO_4605	hypothetical protein PSPTO_4605	**-2.58**
PSPTO_1410	exchangeable effector locus protein	**-2.59**
PSPTO_5619	hypothetical protein PSPTO_5619	**-2.67**
PSPTO_3217	hypothetical protein PSPTO_3217	**-2.69**
PSPTO_4606	hypothetical protein PSPTO_4606	**-2.70**
PSPTO_3616	hypothetical protein PSPTO_3616	**-2.71**
PSPTO_4323	hypothetical protein PSPTO_4323	**-2.93**
PSPTO_5419	hypothetical protein PSPTO_5419	**-2.93**
PSPTO_5421	lipoprotein	**-2.93**
PSPTO_5209	hypothetical protein PSPTO_5209	**-2.94**
PSPTO_3929	cold shock domain family protein	**-2.95**
PSPTO_4322	hypothetical protein PSPTO_4322	**-2.96**
PSPTO_5437	hypothetical protein PSPTO_5437	**-3.00**
PSPTO_5413	EF hand domain-containing protein	**-3.08**
PSPTO_0856	hypothetical protein PSPTO_0856	**-3.17**
PSPTO_5645	hypothetical protein PSPTO_5645	**-3.28**
PSPTO_2422	hypothetical protein PSPTO_2422	**-3.43**
PSPTO_0837	hypothetical protein PSPTO_0837	**-3.53**
PSPTO_0201	hypothetical protein PSPTO_0201	**-3.57**
PSPTO_4272	hypothetical protein PSPTO_4272	**-3.59**
PSPTO_4763	hypothetical protein PSPTO_4763	**-3.63**
PSPTO_0371	indoleacetate-lysine ligase	**-3.64**
PSPTO_4386	hypothetical protein PSPTO_4386	**-3.73**
PSPTO_5646	hypothetical protein PSPTO_5646	**-3.79**
PSPTO_4762	von Willebrand factor type A domain-containing protein	**-3.84**
PSPTO_4387	hypothetical protein PSPTO_4387	**-4.10**
PSPTO_1409	hypothetical protein PSPTO_1409	**-4.15**
PSPTO_2679	hypothetical protein PSPTO_2679	**-4.39**
PSPTO_1408	hypothetical protein PSPTO_1408	**-4.52**
PSPTO_5622	hypothetical protein PSPTO_5622	**-4.79**
PSPTO_4332	hypothetical protein PSPTO_4332	**-5.10**
PSPTO_0875	hypothetical protein PSPTO_0875	**-5.18**
PSPTO_0874	nikkomycin biosynthesis domain protein	**-5.23**
PSPTO_2457	hypothetical protein PSPTO_2457	**-5.34**

Table S7. List of unique genes of *Pss*B728a regulated by (p)ppGpp with |log$_2$FC| value ≥1 and an adjusted p-value <0.05 in (p)ppGpp^0$_{PssB728a}$ than *Pss*B728a.

Locus tag	Gene description	(p)ppGpp^0$_{PssB728a}$ /*Pss*B728a
**Type III secretion system**		
PSYR_4269	type III effector HopAE1	**-1.65**
PSYR_1890	type III effector HopAP1	**-1.64**
PSYR_1220	type III effector HopX1	**-3.04**
PSYR_1219	type III effector protein AvrB3	**-4.56**
PSYR_1198	type III secretion protein HrpF	**-4.04**
PSYR_1192	type III helper protein HrpA2	**-5.96**
PSYR_0738	type III effector protein AvrRpm1	**-2.08**
**Translation, ribosomal structure and biogenesis**		
PSYR_1796	N-acetyltransferase GCN5	**-1.15**
PSYR_1462	N-acetyltransferase GCN5	**-1.62**
PSYR_2691	bifunctional UDP-glucuronic acid decarboxylase/UDP-4-amino-4-deoxy-L-arabinose formyltransferase	**-1.71**
**Transcription**		
PSYR_4299	helix-turn-helix, Fis-type	**1.35**
PSYR_2671	regulatory protein LysR	**-1.18**
PSYR_3087	transcriptional regulator GntR	**-1.29**
PSYR_2564	transcriptional regulator GntR	**-1.92**
PSYR_2607	regulatory protein LuxR	**-2.29**
**Replication, recombination and repair**		
PSYR_3804	ATP-dependent DNA helicase RecQ	**-1.06**
PSYR_1433	C-5 cytosine-specific DNA methylase	**-1.09**
PSYR_0736	umuC protein, partial	**-1.14**
PSYR_3805	hypothetical protein PSYR_3805	**-1.15**
PSYR_2914	ATP-dependent DNA helicase RecQ	**-1.23**
PSYR_3984	DNA helicase-like protein	**-1.32**
PSYR_1522	single-stranded DNA-binding protein	**-1.61**
PSYR_2828	C-5 cytosine-specific DNA methylase	**-2.39**
**Cell cycle control, cell division, chromosome partitioning**		
PSYR_1555	cobyrinic acid a,c-diamide synthase	**-3.51**
**Defense mechanisms**		
PSYR_2541	oligopeptide/dipeptide ABC transporter ATP-binding protein-like protein	**-1.23**
PSYR_2622	acriflavin resistance protein	**-1.60**
PSYR_2483	acriflavin resistance protein	**-1.89**
PSYR_2613	cyclic peptide transporter	**-2.16**
PSYR_4924	hypothetical protein PSYR_4924	**-2.17**
PSYR_3076	Type I secretion system ATPase, HlyB	**-2.72**
**Signal transduction mechanisms**		
PSYR_3329	PAS:GGDEF	**-1.01**

PSYR_2939	diguanylate cyclase	**-1.15**
PSYR_0868	histidine kinase, HAMP region: chemotaxis sensory transducer	**-1.17**
PSYR_2188	histidine kinase	**-1.21**
PSYR_1497	heavy metal response regulator	**-1.22**
PSYR_2597	diguanylate cyclase	**-1.38**
PSYR_1100	hypothetical protein PSYR_1100	**-1.55**
PSYR_1099	response regulator receiver	**-2.27**
PSYR_4206	diguanylate cyclase	**-2.79**
**Cell wall/membrane/envelope biogenesis**		
PSYR_0937	mannose-1-phosphate guanylyltransferase/mannose-6-phosphate isomerase	**1.28**
PSYR_3083	RND efflux system, outer membrane lipoprotein, NodT	**1.03**
PSYR_4997	YD repeat-containing protein, partial	**-1.16**
PSYR_2690	glycosyl transferase family protein	**-1.26**
PSYR_2620	RND efflux system, outer membrane lipoprotein, NodT	**-1.35**
PSYR_0929	glycosyl transferase family protein	**-1.54**
PSYR_1516	hypothetical protein PSYR_1516	**-1.60**
PSYR_4994	rhs-like protein	**-1.72**
PSYR_0920	group 1 glycosyl transferase	**-1.91**
PSYR_2693	4-amino-4-deoxy-L-arabinose transferase	**-1.97**
PSYR_2606	RND efflux system, outer membrane lipoprotein, NodT	**-2.02**
PSYR_2321	YD repeat-containing protein	**-2.48**
PSYR_2322	YD repeat-containing protein	**-2.62**
PSYR_4964	OmpA/MotB protein	**-2.95**
PSYR_4986	YD repeat-containing protein	**-3.40**
PSYR_3088	group 1 glycosyl transferase	**-4.56**
**Cell motility**		
PSYR_0868	histidine kinase, HAMP region: chemotaxis sensory transducer	**-1.17**
PSYR_2188	histidine kinase	**-1.21**
PSYR_1511	type II secretion system protein E	**-1.93**
**Intracellular trafficking, secretion, and vesicular transport**		
PSYR_4643	conjugal transfer protein	**1.69**
PSYR_3805	hypothetical protein PSYR_3805	**-1.15**
PSYR_1517	type II and III secretion system protein	**-1.41**
PSYR_3077	Type I secretion outer membrane protein, TolC	**-1.67**
PSYR_1513	type II secretion system protein	**-1.71**
PSYR_1514	type II secretion system protein E	**-1.71**
PSYR_1512	prepilin	**-1.73**
PSYR_1511	type II secretion system protein E	**-1.93**
PSYR_3075	Type I secretion membrane fusion protein, HlyD	**-3.06**
PSYR_4960	hypothetical protein PSYR_4960	**-3.77**
**Posttranslational modification, protein turnover, chaperones**		
PSYR_4977	ADP-ribosylglycohydrolase	**-1.22**
PSYR_3061	glutathione S-transferase	**-1.47**

**Energy production and conversion**		
PSYR_2387	malate:quinone oxidoreductase	**1.04**
PSYR_2302	FAD linked oxidase domain-containing protein	**-1.03**
PSYR_2672	citrate transporter	**-1.21**
PSYR_0333	hypothetical protein PSYR_0333	**-2.82**
**Carbohydrate transport and metabolism**		
PSYR_4487	carbohydrate kinase PfkB	**1.61**
PSYR_2563	HAD family hydrolase	**-1.37**
PSYR_2186	senescence marker protein-30	**-1.55**
PSYR_2588	HpcH/HpaI aldolase	**-1.69**
PSYR_2923	carbohydrate kinase PfkB	**-1.74**
PSYR_2566	Glycerone kinase	**-1.75**
PSYR_2569	periplasmic binding protein/LacI transcriptional regulator	**-1.83**
PSYR_2692	polysaccharide deacetylase	**-1.90**
PSYR_2437	ABC transporter	**-4.45**
**Amino acid transport and metabolism**		
PSYR_3024	peptidase M14, carboxypeptidase A	**1.08**
PSYR_2855	5-methyltetrahydropteroyltriglutamate/homocysteine S-methyltransferase	**-1.03**
PSYR_4321	pyridoxal-5\'-phosphate-dependent enzyme, beta subunit	**-1.05**
PSYR_0117	class I and II aminotransferase	**-1.21**
PSYR_2585	Orn/DAP/Arg decarboxylase 2:Orn/DAP/Arg decarboxylase 2	**-1.22**
PSYR_2619	diaminobutyrate-2-oxoglutarate transaminase	**-1.24**
PSYR_2604	hypothetical protein PSYR_2604	**-1.29**
PSYR_2567	zinc-containing alcohol dehydrogenase superfamily protein	**-2.17**
PSYR_2964	amino acid ABC transporter permease	**-2.33**
PSYR_2962	extracellular solute-binding protein	**-3.13**
**Coenzyme transport and metabolism**		
PSYR_0334	L-ectoine synthase	**-3.37**
**Lipid transport and metabolism**		
PSYR_0706	acyltransferase 3	**2.13**
PSYR_1703	Fatty acid desaturase	**-1.03**
PSYR_1538	propionyl-CoA carboxylase	**-1.44**
PSYR_4311	malonyl CoA-ACP transacylase	**-1.87**
PSYR_0331	AMP-dependent synthetase and ligase	**-2.51**
**Inorganic ion transport and metabolism**		
PSYR_2546	DMT superfamily multiple drug efflux pump	**-1.02**
PSYR_2603	secretion protein HlyD	**-1.02**
PSYR_2586	EmrB/QacA family drug resistance transporter	**-1.23**
PSYR_4826	TonB-dependent receptor:TonB box, N-terminal, partial	**-1.97**
**Secondary metabolites biosynthesis, transport and catabolism**		
PSYR_3785	DSBA oxidoreductase	**2.00**
PSYR_1493	twin-arginine translocation pathway signal:copper-resistance protein CopA	**1.62**

PSYR_2584	IucA/IucC protein	**-1.10**
PSYR_4314	beta-ketoacyl synthase	**-1.21**
PSYR_3983	serralysin	**-1.21**
PSYR_4312	erythronolide synthase	**-1.26**
PSYR_2615	amino acid adenylation	**-1.30**
PSYR_2587	IucA/IucC protein	**-1.33**
PSYR_2611	amino acid adenylation	**-1.35**
PSYR_2616	amino acid adenylation	**-1.39**
PSYR_2614	amino acid adenylation	**-1.39**
PSYR_4313	beta-ketoacyl synthase	**-1.39**
PSYR_4310	hypothetical protein PSYR_4310	**-1.52**
PSYR_1795	taurine dioxygenase	**-1.58**
PSYR_1792	amino acid adenylation	**-1.63**
PSYR_4311	malonyl CoA-ACP transacylase	**-1.87**
PSYR_2608	amino acid adenylation	**-1.93**
PSYR_1793	amino acid adenylation	**-2.15**
PSYR_3074	hemolysin-type calcium-binding protein	**-2.30**
PSYR_1794	amino acid adenylation	**-2.37**
PSYR_3089	hemolysin-type calcium-binding protein	**-3.53**
**Function unknown**		
PSYR_0744	hypothetical protein	**3.14**
PSYR_0745	hypothetical protein	**2.93**
PSYR_4745	hypothetical protein PSYR_4745	**2.79**
PSYR_2324	hypothetical protein PSYR_2324	**2.49**
PSYR_2348	hypothetical protein PSYR_2348	**2.38**
PSYR_2687	PepSY-associated TM helix family protein	**2.34**
PSYR_4604	hypothetical protein PSYR_4604	**2.26**
PSYR_0743	hypothetical protein	**2.16**
PSYR_4744	hypothetical protein PSYR_4744	**2.13**
PSYR_2140	hypothetical protein PSYR_2140	**2.09**
PSYR_0467	hypothetical protein PSYR_0467	**2.07**
PSYR_3783	hypothetical protein PSYR_3783	**1.50**
PSYR_4298	hypothetical protein PSYR_4298	**1.47**
PSYR_0882	hypothetical protein PSYR_0882	**1.44**
PSYR_4582	hypothetical protein PSYR_4582	**1.41**
PSYR_2386	hypothetical protein PSYR_2386	**1.34**
PSYR_2347	hypothetical protein PSYR_2347	**1.30**
PSYR_1866	hypothetical protein PSYR_1866	**1.30**
PSYR_4586	tail protein	**1.29**
PSYR_3870	SecC motif-containing protein	**1.25**
PSYR_4723	hypothetical protein PSYR_4723	**1.22**
PSYR_2641	hypothetical protein PSYR_2641	**1.21**
PSYR_1475	Phage integrase:Phage integrase, N-terminal SAM-like	**1.13**
PSYR_2952	NADP oxidoreductase, coenzyme F420-dependent	**1.12**

PSYR_2813	hypothetical protein PSYR_2813	**1.07**
PSYR_0194	short-chain dehydrogenase	**1.06**
PSYR_4587	baseplate J-like protein	**1.06**
PSYR_3216	hypothetical protein PSYR_3216	**1.03**
PSYR_4806	hypothetical protein PSYR_4806	**1.02**
PSYR_2861	hypothetical protein PSYR_2861	**-1.00**
PSYR_4655	hypothetical protein PSYR_4655	**-1.02**
PSYR_4127	hypothetical protein PSYR_4127	**-1.03**
PSYR_0773	aryldialkylphosphatase	**-1.03**
PSYR_3746	hypothetical protein PSYR_3746	**-1.03**
PSYR_4661	phosphopantetheinyl transferase	**-1.03**
PSYR_4000	hypothetical protein PSYR_4000	**-1.04**
PSYR_0101	hypothetical protein PSYR_0101	**-1.04**
PSYR_0118	hypothetical protein PSYR_0118	**-1.04**
PSYR_1934	hypothetical protein PSYR_1934	**-1.04**
PSYR_2561	hypothetical protein PSYR_2561	**-1.07**
PSYR_2661	hypothetical protein PSYR_2661	**-1.07**
PSYR_2559	hypothetical protein PSYR_2559	**-1.07**
PSYR_2792	Phage head morphosis protein, SPP1 gp7	**-1.09**
PSYR_0341	hypothetical protein PSYR_0341	**-1.09**
PSYR_5114	hypothetical protein PSYR_5114	**-1.10**
PSYR_2797	hypothetical protein PSYR_2797	**-1.10**
PSYR_4309	hypothetical protein PSYR_4309	**-1.10**
PSYR_3494	hypothetical protein PSYR_3494	**-1.11**
PSYR_1935	hypothetical protein PSYR_1935	**-1.11**
PSYR_3341	hypothetical protein PSYR_3341	**-1.13**
PSYR_1527	hypothetical protein PSYR_1527	**-1.16**
PSYR_2299	hypothetical protein PSYR_2299	**-1.16**
PSYR_2663	hypothetical protein PSYR_2663	**-1.16**
PSYR_1554	hypothetical protein PSYR_1554	**-1.18**
PSYR_0940	hypothetical protein	**-1.18**
PSYR_2660	hypothetical protein PSYR_2660	**-1.19**
PSYR_1530	hypothetical protein PSYR_1530	**-1.20**
PSYR_0923	hypothetical protein PSYR_0923	**-1.21**
PSYR_0112	hypothetical protein PSYR_0112	**-1.22**
PSYR_1927	hypothetical protein PSYR_1927	**-1.22**
PSYR_3730	hypothetical protein PSYR_3730	**-1.22**
PSYR_0739	hypothetical protein	**-1.23**
PSYR_2317	hypothetical protein	**-1.23**
PSYR_4486	hypothetical protein PSYR_4486	**-1.24**
PSYR_1524	hypothetical protein PSYR_1524	**-1.27**
PSYR_1931	hypothetical protein PSYR_1931	**-1.27**
PSYR_0070	hypothetical protein PSYR_0070	**-1.28**
PSYR_4437	hypothetical protein PSYR_4437	**-1.31**

PSYR_3728	hypothetical protein PSYR_3728	**-1.32**
PSYR_3729	hypothetical protein PSYR_3729	**-1.32**
PSYR_0939	hypothetical protein	**-1.32**
PSYR_1255	hypothetical protein PSYR_1255	**-1.34**
PSYR_2560	hypothetical protein PSYR_2560	**-1.35**
PSYR_1924	hypothetical protein PSYR_1924	**-1.37**
PSYR_4989	hypothetical protein PSYR_4989	**-1.37**
PSYR_2841	hypothetical protein PSYR_2841	**-1.38**
PSYR_2673	hypothetical protein PSYR_2673	**-1.38**
PSYR_3815	hypothetical protein PSYR_3815	**-1.38**
PSYR_2826	hypothetical protein PSYR_2826	**-1.39**
PSYR_4085	hypothetical protein PSYR_4085	**-1.40**
PSYR_5115	helicase	**-1.41**
PSYR_2316	ral secretion pathway protein I	**-1.41**
PSYR_1523	hypothetical protein PSYR_1523	**-1.42**
PSYR_1926	hypothetical protein PSYR_1926	**-1.43**
PSYR_2799	hypothetical protein PSYR_2799	**-1.45**
PSYR_2674	hypothetical protein PSYR_2674	**-1.45**
PSYR_2609	Alpha/beta hydrolase fold	**-1.45**
PSYR_1529	hypothetical protein PSYR_1529	**-1.47**
PSYR_3816	hypothetical protein PSYR_3816	**-1.48**
PSYR_2800	hypothetical protein PSYR_2800	**-1.49**
PSYR_3133	amidohydrolase 2	**-1.52**
PSYR_2185	hypothetical protein PSYR_2185	**-1.55**
PSYR_3534	histidine kinase, HAMP region:Cache: chemotaxis sensory transducer	**-1.58**
PSYR_0104	hypothetical protein PSYR_0104	**-1.58**
PSYR_0772	hypothetical protein PSYR_0772	**-1.58**
PSYR_1460	hypothetical protein PSYR_1460	**-1.58**
PSYR_1928	regulatory protein LysR	**-1.62**
PSYR_0893	hypothetical protein PSYR_0893	**-1.62**
PSYR_1470	hypothetical protein PSYR_1470	**-1.63**
PSYR_4955	hypothetical protein PSYR_4955	**-1.66**
PSYR_4514	hypothetical protein PSYR_4514	**-1.67**
PSYR_0330	hypothetical protein PSYR_0330	**-1.69**
PSYR_1519	SecC motif-containing protein	**-1.70**
PSYR_1471	hypothetical protein PSYR_1471	**-1.70**
PSYR_1713	hypothetical protein PSYR_1713	**-1.72**
PSYR_4207	N-acetyltransferase GCN5	**-1.72**
PSYR_2840	hypothetical protein PSYR_2840	**-1.73**
PSYR_1263	ATPase	**-1.77**
PSYR_1539	histidine kinase, HAMP region: chemotaxis sensory transducer	**-1.84**
PSYR_2791	hypothetical protein PSYR_2791	**-1.88**
PSYR_3090	hypothetical protein PSYR_3090	**-1.89**

PSYR_3998	heat shock protein DnaJ, N-terminal, partial	**-1.93**
PSYR_0100	hypothetical protein PSYR_0100	**-1.95**
PSYR_1930	hypothetical protein PSYR_1930	**-1.97**
PSYR_0167	hypothetical protein PSYR_0167	**-1.98**
PSYR_4973	hypothetical protein PSYR_4973	**-2.01**
PSYR_1204	hypothetical protein PSYR_1204	**-2.08**
PSYR_4985	hypothetical protein PSYR_4985	**-2.12**
PSYR_2650	hypothetical protein PSYR_2650	**-2.13**
PSYR_4956	hypothetical protein PSYR_4956	**-2.14**
PSYR_2646	radical SAM family protein	**-2.16**
PSYR_1472	hypothetical protein PSYR_1472	**-2.16**
PSYR_4984	hypothetical protein PSYR_4984	**-2.16**
PSYR_4082	hypothetical protein PSYR_4082	**-2.18**
PSYR_2963	amino acid ABC transporter permease	**-2.21**
PSYR_2568	short chain dehydrogenase	**-2.23**
PSYR_4509	hypothetical protein PSYR_4509	**-2.23**
PSYR_2824	hypothetical protein PSYR_2824	**-2.31**
PSYR_1515	hypothetical protein PSYR_1515	**-2.33**
PSYR_2651	hypothetical protein PSYR_2651	**-2.33**
PSYR_2842	hypothetical protein PSYR_2842	**-2.40**
PSYR_0332	hypothetical protein PSYR_0332	**-2.40**
PSYR_2652	hypothetical protein PSYR_2652	**-2.42**
PSYR_2837	hypothetical protein PSYR_2837	**-2.48**
PSYR_4083	hypothetical protein PSYR_4083	**-2.50**
PSYR_0750	hypothetical protein PSYR_0750	**-2.51**
PSYR_4872	hypothetical protein PSYR_4872	**-2.53**
PSYR_0099	hypothetical protein PSYR_0099	**-2.57**
PSYR_4968	hypothetical protein PSYR_4968	**-2.63**
PSYR_4983	Rhs element Vgr protein	**-2.63**
PSYR_1929	hypothetical protein PSYR_1929	**-2.70**
PSYR_2836	hypothetical protein PSYR_2836	**-2.73**
PSYR_0097	hypothetical protein PSYR_0097	**-2.75**
PSYR_4957	hypothetical protein PSYR_4957	**-2.75**
PSYR_3719	hypothetical protein PSYR_3719	**-2.79**
PSYR_1474	hypothetical protein PSYR_1474	**-2.87**
PSYR_2644	hypothetical protein PSYR_2644	**-2.88**
PSYR_3718	hypothetical protein PSYR_3718	**-2.88**
PSYR_4967	hypothetical protein PSYR_4967	**-2.96**
PSYR_4972	Sel1 repeat-containing protein	**-3.00**
PSYR_1203	hypothetical protein PSYR_1203	**-3.06**
PSYR_2648	hypothetical protein PSYR_2648	**-3.06**
PSYR_2647	hypothetical protein PSYR_2647	**-3.11**
PSYR_2612	syrP protein	**-3.19**
PSYR_4963	hypothetical protein PSYR_4963	**-3.23**

PSYR_2649	hypothetical protein PSYR_2649	**-3.28**
PSYR_4959	hypothetical protein PSYR_4959	**-3.38**
PSYR_4982	hypothetical protein PSYR_4982	**-3.39**
PSYR_3807	hypothetical protein PSYR_3807	**-3.58**
PSYR_0928	hypothetical protein PSYR_0928	**-3.71**
PSYR_4966	ImpA-like protein	**-3.85**
PSYR_4510	hypothetical protein PSYR_4510	**-3.85**
PSYR_3809	hypothetical protein PSYR_3809	**-3.87**
PSYR_0737	transmembrane protein	**-3.87**
PSYR_2323	hypothetical protein PSYR_2323	**-4.05**
PSYR_4962	hypothetical protein PSYR_4962	**-4.07**
PSYR_4971	Sel1 repeat-containing protein	**-4.24**
PSYR_0098	hypothetical protein PSYR_0098	**-4.31**
PSYR_0927	hypothetical protein PSYR_0927	**-4.32**
PSYR_4511	hypothetical protein PSYR_4511	**-4.38**
PSYR_4961	hypothetical protein PSYR_4961	**-4.38**
PSYR_4965	hypothetical protein PSYR_4965	**-4.42**
PSYR_3808	hypothetical protein PSYR_3808	**-5.06**
PSYR_4987	hypothetical protein PSYR_4987	**-5.37**
