# Supplementary material for: Comparative transcriptomic analysis of global gene expression mediated by (p) ppGpp reveals common regulatory networks in Pseudomonas syringae
Source: BMC Genomics. 2020 Apr 10;21:296. doi: 10.1186/s12864-020-6701-2 (PMC7146990; doi:10.1186/s12864-020-6701-2)
Supplement: Supplementary file 5 — Additional file 5: Table S8. List of differentially expressed genes (DEGs) related to type III secretion system (T3SS) and type VI secretion system (T6SS). Table S9. List of DEGs related to cell motility, division, and exopolysaccharides (EPS). Table S10. List of DEGs related to DNA replication, RNA processes and ribosomal protein biosynthesis. Table S11. List of DEGs related to nucleotide, amino acid and fatty acid metabolism. Table S12. List of DEGs related to coenzyme and carbon metabolism. Table S13. List of DEGs related to signal transduction and transcription. [file 12864_2020_6701_MOESM5_ESM.zip › Additional file 5 Table S11 & S12 & S13.pdf]

Table S11. List of differentially expressed genes (DEGs) related to nucleotide, fatty acid and amino acid metabolism

**Locus tag**		**Description**	**(p)ppGpp$^{0}_{PstDC3000}$ /*Pst*DC3000**	**(p)ppGpp$^{0}_{PssB728a}$ /*Pss*B728a**
**DC3000**	**B728A**			
**Nucleotide metabolism**				
**Purine**				
PSPTO_1449	*PSYR_1261*	inosine 5\'-monophosphate dehydrogenase GuaB	**1.35**	**0.44**
PSPTO_1459	*PSYR_1269*	phosphoribosylformylglycinamidine synthase PurA	**1.65**	**0.6**
PSPTO_1468	*PSYR_1277*	phosphoribosylglycinamide formyltransferase 2 PurT	**1.74**	**1.14**
PSPTO_1509	*PSYR_1319*	adenylate kinase Adk	**2.19**	**1.6**
PSPTO_1699	*PSYR_3690*	phosphoribosylglycinamide formyltransferase PurN	**2.17**	**0.37**
PSPTO_1700	*PSYR_3689*	phosphoribosylaminoimidazole synthetase PurM	**2.81**	**1.43**
PSPTO_3360	*PSYR_3192*	adenylosuccinate lyase PurB	**1.85**	**0.29**
PSPTO_3811	*PSYR_1668*	amidophosphoribosyltransferase PurF	**1.87**	**0.73**
PSPTO_4314	*PSYR_4018*	formyltetrahydrofolate deformylase PurU3	**1.58**	**1.01**
PSPTO_4937	*PSYR_0577*	adenylosuccinate synthetase PurA	**2.31**	**0.79**
Pyrimidine				
PSPTO_0080	*PSYR_0216*	orotate phosphoribosyltransferase PyrE	**1.54**	**1.08**
PSPTO_1552	*PSYR_1361*	CTP synthase PyrG	**1.05**	**0.16**
PSPTO_2028	*PSYR_1836*	orotidine 5\'-phosphate decarboxylase PyrF	**2.7**	**1.7**
PSPTO_2309	*PSYR_2106*	dihydroorotate dehydrogenase PyrD	**1.96**	**0.15**
PSPTO_4157	*PSYR_3894*	dihydroorotase, homodimeric type PyrC	**1.73**	**1.3**
PSPTO_4502	*PSYR_4192*	carbamoyl-phosphate synthase small subunit CarA	**1.23**	**1.16**
PSPTO_5039	*PSYR_0483*	bifunctional pyrimidine regulatory protein PyrR/uracil phosphoribosyltransferase	**0.4**	**1.48**
PSPTO_5040	*PSYR_0482*	aspartate carbamoyltransferase catalytic subunit PyrB	**0.18**	**1.05**
PSPTO_5282	*PSYR_4840*	thymidylate synthase ThyA	**1.58**	**1.55**
**Fatty acid metabolism**				
**Fatty acid biosynthesis**				
PSPTO_0305	*PSYR_0087*	fatty acid desaturase DesA	**2.33**	**2.06**
PSPTO_1825	*PSYR_3572*	acetyl-CoA synthetase AcsA	**2.46**	**5.01**
PSPTO_2210	*PSYR_2019*	3-oxoacyl-(acyl carrier protein) synthase I FabB	**1.25**	**1.49**
PSPTO_2211	*PSYR_2020*	3-hydroxydecanoyl-(acyl carrier protein) dehydratase FabA	**1.05**	**1.36**
PSPTO_3815	*PSYR_1664*	acetyl-CoA carboxylase subunit beta AccD	**1.29**	**0.69**
PSPTO_3831	*PSYR_1648*	acyl carrier protein AcpP	**1.17**	**0.49**
PSPTO_3833	*PSYR_1646*	malonyl CoA-acyl carrier protein transacylase FabD	**1.01**	**0.11**

PSPTO_5093	*PSYR_0437*	acyl carrier protein	**1.43**	**1.61**
PSPTO_5094	*PSYR_0436*	acyl carrier protein	**1.49**	**1.83**
**Fatty acid degradation**				
PSPTO_1790	*PSYR_3604*	acyl-CoA dehydrogenase family protein	**-1.13**	**-2.13**
PSPTO_2397	*PSYR_2568*	short chain dehydrogenase	**-1.91**	**-2.15**
PSPTO_2492	*PSYR_2297*	short-chain dehydrogenase/reductase family oxidoreductase	**-1.73**	**-1.46**
PSPTO_5184	*PSYR_0354*	acyl-CoA dehydrogenase family protein	**-2.49**	**-3.02**
PSPTO_5185	*PSYR_0353*	acyl-CoA dehydrogenase family protein	**-1.12**	**-1.65**
**Amino acid biosynthesis**				
**Arginine**				
PSPTO_0125	*PSYR_0065*	argininosuccinate lyase ArgH	**1.43**	**0.75**
PSPTO_1826	*PSYR_3571*	arginine/ornithine ABC transporter,	**2.12**	**2.3**
PSPTO_1827	*PSYR_3570*	arginine/ornithine ABC transporter, permease	**1.51**	**1.16**
PSPTO_1828	*PSYR_3569*	arginine/ornithine ABC transporter, permease	**1.65**	**0.69**
PSPTO_4164	*PSYR_3901*	ornithine carbamoyltransferase ArgF	**1.31**	**0.52**
**Aromatic**				
PSPTO_0169	*PSYR_0025*	shikimate 5-dehydrogenase AroE	**1.79**	**0.86**
PSPTO_1041	*PSYR_0888*	3-phosphoshikimate 1-carboxyvinyltransferase AroA	**1.49**	**0.27**
PSPTO_2043	*PSYR_1853*	chorismate synthase AroC	**1.59**	**1.21**
PSPTO_5126	*PSYR_0409*	3-dehydroquinate synthase AroB	**1.26**	**0.95**
**Diaminopimelate**				
PSPTO_0224	*PSYR_0183*	diaminopimelate epimerase DapF	**1.05**	**0.52**
PSPTO_0225	*PSYR_0182*	diaminopimelate decarboxylase LsyA-2	**1.4**	**1.17**
PSPTO_1523	*PSYR_1331*	succinyl-diaminopimelate desuccinylase DapE	**1.43**	**0.83**
PSPTO_3953	*PSYR_1548*	dihydrodipicolinate synthase DapA	**1.91**	**0.88**
PSPTO_4503	*PSYR_4193*	dihydrodipicolinate reductase DapB	**1.91**	**0.44**
**Histidine**				
PSPTO_4439	*PSYR_4134*	ATP phosphoribosyltransferase HisG	**1.04**	**0.41**
PSPTO_5334	*PSYR_4893*	Imidazole glycerol phosphate synthase cyclase subunit HisF	**1.03**	**0.92**
PSPTO_5335	*PSYR_4894*	phosphoribosylformimino-5-aminoimidazole carboxamide ribotide isomerase HisA	**1.17**	**1.14**
PSPTO_5338	*PSYR_4897*	imidazoleglycerol-phosphate dehydratase hisB	**1.02**	**0.89**
**Methionine**				
PSPTO_1744	*PSYR_3648*	initiation factor 2 subunit family	**1.94**	**1.36**
PSPTO_5069	*PSYR_0459*	5,10-methylenetetrahydrofolate reductase metF	**1.71**	**0.74**
PSPTO_5261	*PSYR_0281*	D-methionine ABC transporter permease MetI-2	**2.19**	**1.62**
PSPTO_5262	*PSYR_0282*	DL-methionine transporter ATP-binding subunit MetN-2	**2.32**	**1.35**
**Tryptophan**				
PSPTO_0568	*PSYR_4609*	anthranilate synthase, component I TrpE	**1.52**	**1.35**
PSPTO_0592	*PSYR_4581*	anthranilate synthase component II	**0.4**	**1.36**

PSPTO_0593	*PSYR_4580*	anthranilate phosphoribosyltransferase	**0.9**	**1.28**
PSPTO_3816	*PSYR_1663*	N-(5'-phosphoribosyl)anthranilate isomerase TrpF	**1.43**	**0.62**
**Amino acid transport**				
PSPTO_1255	*PSYR_1072*	amino acid ABC transporter substrate-binding protein	**-2.54**	**-0.79**
PSPTO_1256	*PSYR_1073*	amino acid ABC transporter permease	**-2.77**	**-2.3**
PSPTO_1257	*PSYR_1074*	amino acid ABC transporter permease	**-2.56**	**-2.15**
PSPTO_1258	*PSYR_1075*	amino acid ABC transporter ATP-binding protein	**-2.48**	**-1.71**
PSPTO_2630	*PSYR_2965*	amino acid ABC transporter ATP-binding protein	**-0.6**	**-3.52**
PSPTO_2776	*PSYR_2504*	amino acid ABC transporter permease	**-2.23**	**-0.4**
PSPTO_2777	*PSYR_2505*	amino acid ABC transporter permease	**-2.04**	**-0.38**
PSPTO_2778	*PSYR_2506*	amino acid ABC transporter ATP-binding protein	**-1.42**	**-0.2**
PSPTO_4109	*PSYR_3846*	high-affinity branched-chain amino acid ABC transporter permease	**-2.72**	**-2.33**
PSPTO_4112	*PSYR_3849*	high-affinity amino acid ABC transporter, ATP-binding protein	**-1.13**	**-2.99**
PSPTO_4915	*PSYR_0601*	high affinity branched-chain amino acid ABC transporter ATP-binding protein	**-1.17**	**-1.52**
PSPTO_4916	*PSYR_0600*	high affinity branched-chain amino acid ABC transporter ATP-binding protein	**-1.36**	**-1.42**
PSPTO_4917	*PSYR_0599*	high-affinity branched-chain amino acid ABC transporter, permease protein BraE	**-1.42**	**-1.12**
PSPTO_4918	*PSYR_0598*	high-affinity branched-chain amino acid ABC transporter, permease protein BraD	**-1.45**	**-0.55**
PSPTO_4919	*PSYR_0597*	high affinity branched-chain amino acid ABC transporter periplasmic amino acid-binding protein	**-1.45**	**-0.38**

DEGs were differentially expressed genes in the (p)ppGpp^0$_{Pst\mathrm{DC3000}}$ and (p)ppGpp^0$_{Pss\mathrm{B728a}}$ with p-value <0.05 between the WT and the (p)ppGpp^0 mutants.

Table S12. List of differentially expressed genes (DEGs) related to coenzyme and carbon metabolism

Locus tag		Description	(p)ppGpp[0]*Pst*DC3000 /*Pst*DC3000	(p)ppGpp[0]*Pss*B728a /*Pss*B728a
**DC3000**	**B728A**			
**Coenzyme metabolism**				
**Riboflavin biosynthesis**				
PSPTO_0690	*PSYR_4462*	riboflavin biosynthesis protein RibD	**2.02**	**0.98**
PSPTO_0691	*PSYR_4461*	riboflavin synthase subunit alpha RibE	**2.34**	**1.24**
PSPTO_0692	*PSYR_4460*	GTP cyclohydrolase II-like protein RibBA-1	**1.86**	**0.8**
PSPTO_0696	*PSYR_4456*	GTP cyclohydrolase II RibA	**1.37**	**0.74**
PSPTO_0805	*PSYR_0709*	riboflavin biosynthesis protein RibF	**1.34**	**1.26**
PSPTO_1839	*PSYR_3558*	riboflavin synthase subunit beta RibH	**0.04**	**1.45**
**Niacin biosynthesis**				
PSPTO_3959	*PSYR_1542*	quinolinate synthetase NadA	**3.44**	**1.74**
PSPTO_0948	*PSYR_0815*	nicotinate-nucleotide pyrophosphorylase NadC	**1.25**	**1.25**
PSPTO_4828	*PSYR_4368*	nicotinate (nicotinamide) nucleotide adenylyltransferase NadD	**1.11**	**1.08**
**Heme biosynthesis**				
PSPTO_0170	*PSYR_0024*	coproporphyrinogen III oxidase HemF	**2.3**	**1.22**
PSPTO_4800	*PSYR_4342*	glutamate-1-semialdehyde-2,1-aminomutase HemL	**1.82**	**1.7**
PSPTO_5118	*PSYR_0414*	uroporphyrinogen decarboxylase HemE	**1.72**	**0.82**
PSPTO_0128	*PSYR_0062*	porphobilinogen deaminase HemC	**1.08**	**0.96**
PSPTO_1108	*PSYR_0948*	glutamyl-tRNA reductase HemA	**1.03**	**1.04**
PSPTO_1128	*PSYR_0967*	ferrochelatase HemH	**0.98**	**1.49**
PSPTO_5251	*PSYR_0292*	delta-aminolevulinic acid dehydratase HemB	**0.76**	**1.21**
**Down-regulated**				
PSPTO_3466	*PSYR_3247*	alkanesulfonate monooxygenase ssuD	**-3.1**	**-3.33**
PSPTO_3299	*PSYR_3129*	3-hydroxyacyl-CoA-acyl transferase PhAG-1	**-1.86**	**-4.12**
PSPTO_3148	*PSYR_3014*	magnesium chelatase, subunit ChlI	**-1.1**	**-1.28**
**Carbon metabolism**				
**Mannitol metabolism**				
PSPTO_2703	*PSYR_2436*	D-mannonate oxidoreductase UxuB	**-3.49**	**-5.27**
PSPTO_2705	*PSYR_2438*	mannitol ABC transporter permease	**-2.67**	**-4.23**
PSPTO_2706	*PSYR_2439*	mannitol ABC transporter permease	**-2.06**	**-3.81**
**Xylose metabolism**				
PSPTO_2702	*PSYR_2435*	xylulokinase XylB	**-3.78**	**-5.64**
PSPTO_3002	*PSYR_2883*	xylose isomerase XylA	**-1.3**	**-1.43**
PSPTO_3003	*PSYR_2884*	D-xylose ABC transporter, XylF	**-1.47**	**-1.26**

		periplasmic-D xylose binding protein		
PSPTO_3004	*PSYR_2885*	xylose transporter ATP-binding subunit XylG	**-1.23**	**-1.44**
**Glycogen metabolism**				
PSPTO_2762	*PSYR_2491*	1,4-alpha-glucan-branching protein GlgB	**-1.54**	**-1.2**
PSPTO_3125	*PSYR_2992*	glycogen synthase GlgA	**-1.76**	**-0.85**
PSPTO_3130	*PSYR_2997*	glycogen operon protein GlgX	**-2.13**	**-1.27**
PSPTO_5165	*PSYR_0383*	glycogen phosphorylase GlgP	**-1.34**	**-0.79**
**Up-regulated**				
PSPTO_5289	*PSYR_4847*	ribose 5-phosphate isomerase RpiA	**2.5**	**1.41**
PSPTO_1136	*PSYR_0976*	malate:quinone oxidoreductase Mqo	**2.28**	**1.52**
PSPTO_4494	*PSYR_4184*	triosephosphate isomerase TpiA	**1.79**	**1.06**

DEGs were differentially expressed genes in the (p)ppGpp^0$_{Pst\text{DC3000}}$ and (p)ppGpp^0$_{Pss\text{B728a}}$ with p-value <0.05 between the WT and the (p)ppGpp^0 mutants.

Table S13. List of differentially expressed genes (DEGs) related to signal transduction and transcription

**Locus tag**		**Description**	**(p)ppGpp^0$_{Pst\text{DC3000}}$ /*Pst*DC3000**	**(p)ppGpp^0$_{Pss\text{B728a}}$ /*Pss*B728a**
**DC3000**	**B728A**			
**Up-regulated**				
PSPTO_4180	*PSYR_3917*	transcriptional activator MetR	**2.51**	**1.17**
PSPTO_0689	*PSYR_4463*	transcriptional regulator NrdR	**2.44**	**1.17**
PSPTO_0998	*PSYR_0863*	alkylphosphonate utilization operon protein PhnA	**2.41**	**2.79**
PSPTO_4638	*PSYR_4273*	carbon starvation protein CstA	**2.07**	**2.91**
PSPTO_1733	*PSYR_3659*	bolA protein	**1.64**	**1.39**
PSPTO_2215	*PSYR_2024*	phosphohistidine phosphatase SixA	**1.09**	**1.21**
**Down-regulated**				
PSPTO_1844	*PSYR_3554*	carbon storage regulator CsrA2	**-1.76**	**-1.29**
PSPTO_4784	*PSYR_4206*	diguanylate cyclase	**-1.26**	**-2.69**
PSPTO_2591	*PSYR_2281*	diguanylate cyclase	**-1.27**	**-1.4**
PSPTO_0339	*PSYR_0266*	diguanylate cyclase	**-1.22**	**-2.01**
PSPTO_2370	*PSYR_2154*	ribose operon repressor RbsR	**-1.16**	**-1.22**

DEGs were differentially expressed genes in the (p)ppGpp^0$_{Pst\text{DC3000}}$ and (p)ppGpp^0$_{PssB\text{728a}}$ with p-value <0.05 between the WT and the (p)ppGpp^0 mutants.
