# Supplementary material for: Comparative transcriptomic analysis of global gene expression mediated by (p) ppGpp reveals common regulatory networks in Pseudomonas syringae
Source: BMC Genomics. 2020 Apr 10;21:296. doi: 10.1186/s12864-020-6701-2 (PMC7146990; doi:10.1186/s12864-020-6701-2)
Supplement: Supplementary file 5 — Additional file 5: Table S8. List of differentially expressed genes (DEGs) related to type III secretion system (T3SS) and type VI secretion system (T6SS). Table S9. List of DEGs related to cell motility, division, and exopolysaccharides (EPS). Table S10. List of DEGs related to DNA replication, RNA processes and ribosomal protein biosynthesis. Table S11. List of DEGs related to nucleotide, amino acid and fatty acid metabolism. Table S12. List of DEGs related to coenzyme and carbon metabolism. Table S13. List of DEGs related to signal transduction and transcription. [file 12864_2020_6701_MOESM5_ESM.zip › Additional file 5 Table S8 & S9 & S10.pdf]

Table S8. List of differentially expressed genes (DEGs) related to type III secretion system (T3SS) and type VI secretion system (T6SS)

Locus tag		Description	(p)ppGpp[0] $_{Pst\mathrm{DC3000}}$ /*Pst*DC3000	(p)ppGpp[0] $_{Pss\mathrm{B728a}}$ /*Pss*B728a
**DC3000**	**B728A**			
**Type III secretion system (T3SS)**				
PSPTO_1404	*PSYR_1217*	RNA polymerase sigma factor HrpL	**-2.82**	**-3.78**
PSPTO_0588	*PSYR_1889*	type III effector HopH1	**-4.94**	**-4.24**
PSPTO_0905	*PSYR_0779*	type III effector HopAH1	**-1.5**	**-2.54**
PSPTO_0901	*PSYR_0778*	type III effector HopAG1	**-2.53**	**-1.99**
PSPTO_1372	*PSYR_1183*	type III effector HopAA1-1	**-3.37**	**-5.28**
PSPTO_1375	*PSYR_1186*	type III effector HopM1	**-4.64**	**-1.75**
PSPTO_1377	*PSYR_1188*	type III effector protein AvrE1	**-4.13**	**-2.77**
PSPTO_1568	*PSYR_3813*	type III effector HopAF1	**-2.25**	**-1.91**
PSPTO_3087	*PSYR_4659*	type III effector HopAB2	**-4.14**	**-1.28**
PSPTO_4001	*PSYR_4919*	type III effector protein AvrPto1	**-2.36**	**-6.01**
PSPTO_4776	*PSYR_4326*	type III effector HopI1	**-4.36**	**-2.05**
PSPTO_1383	*PSYR_1194*	type III secretion protein HrpB	**-5.17**	**-5.17**
PSPTO_1384	*PSYR_1195*	type III secretion protein HrcJ	**-5.11**	**-4.24**
PSPTO_1385	*PSYR_1196*	type III secretion protein HrpD	**-4.76**	**-4.24**
PSPTO_1386	*PSYR_1197*	type III secretion protein HrpE	**-4.43**	**-3.46**
PSPTO_1388	*PSYR_1199*	type III secretion protein HrpG	**-3.55**	**-3.63**
PSPTO_1390	*PSYR_1201*	type III secretion protein HrpT	**-4.47**	**-4.07**
PSPTO_1392	*PSYR_1205*	type III secretion protein HrcU	**-3.73**	**-2.74**
PSPTO_1393	*PSYR_1206*	type III secretion protein HrcT	**-3.58**	**-3.58**
PSPTO_1394	*PSYR_1207*	type III secretion protein HrcS	**-3.98**	**-4.32**
PSPTO_1395	*PSYR_1208*	type III secretion protein HrcR	**-4.05**	**-4.69**
PSPTO_1396	*PSYR_1209*	type III secretion protein HrcQb	**-3.82**	**-5.76**
PSPTO_1397	*PSYR_1210*	type III secretion protein HrcQa	**-3.54**	**-4.91**
PSPTO_1398	*PSYR_1211*	type III secretion protein HrpP	**-4.17**	**-6.34**
PSPTO_1399	*PSYR_1212*	type III secretion protein HrpO	**-4.72**	**-5.2**
PSPTO_1400	*PSYR_1213*	type III secretion cytoplasmic ATPase HrcN	**-4.51**	**-3.94**
PSPTO_1401	*PSYR_1214*	type III secretion protein HrpQ	**-4.15**	**-3.59**
PSPTO_1402	*PSYR_1215*	type III secretion protein HrcV	**-3.45**	**-3.37**
PSPTO_1403	*PSYR_1216*	type III secretion protein HrpJ	**-3.46**	**-3.46**
PSPTO_1373	*PSYR_1184*	type III helper protein HrpW1	**-6.12**	**-4.31**
PSPTO_1405	*PSYR_1218*	type III helper protein HrpK1	**-4.36**	**-4.65**
PSPTO_4101	*PSYR_3839*	type III helper protein HopAK1	**-4.59**	**-2.26**
PSPTO_1374	*PSYR_1185*	type III chaperone ShcM	**-4.49**	**-3.35**
PSPTO_1376	*PSYR_1187*	type III chaperone ShcE	**-3.2**	**-0.95**
PSPTO_1378	*PSYR_1189*	membrane-bound lytic murein transglycosylase D, HrpH	**-4.11**	**-2.5**
PSPTO_1382	*PSYR_1193*	type III restriction system	**-5.22**	**-5.36**

		endonuclease HrpZ1		
PSPTO_1389	*PSYR_1200*	outer-membrane type III secretion protein HrcC	**-4.22**	**-2.95**
PSPTO_1391	*PSYR_1202*	negative regulator of hrp expression HrpV	**-4.04**	**-3.91**
**Type VI secretion system (T6SS)**				
PSPTO_2872	*PSYR_2631*	HopL1 protein	**-2.22**	**-4.18**
PSPTO_2873	*PSYR_2630*	hypothetical protein PSPTO_2873	**-1.65**	**-4.73**
PSPTO_2874	*PSYR_2629*	ppkA-related protein	**-2.07**	**-5.03**
PSPTO_2875	*PSYR_2628*	ABC transporter ATP-binding protein	**-1.73**	**-4.67**
PSPTO_2876	*PSYR_2627*	ABC transporter permease	**-1.33**	**-3.2**
PSPTO_2877	*PSYR_2626*	hypothetical protein PSPTO_2877	**-2.09**	**-4.1**
PSPTO_2878	*PSYR_2625*	lipoprotein	**-2.42**	**-3.51**

DEGs were differentially expressed genes in the (p)ppGpp$^{0}_{Pst\mathrm{DC3000}}$ and (p)ppGpp$^{0}_{Pss\mathrm{B728a}}$ with p-value <0.05 between the WT and the (p)ppGpp^{0} mutants.

Table S9. List of differentially expressed genes (DEGs) related to cell motility, division, and exopolysaccharides (EPS)

Locus tag		Description	(p)ppGpp^0$_{Pst\mathrm{DC3000}}$ /*Pst*DC3000	(p)ppGpp^0$_{Pss\mathrm{B728a}}$ /*Pss*B728a
**DC3000**	**B728A**			
**Cell motility**				
PSPTO_1933	*PSYR_3481*	flagellar basal-body rod protein FlgB	**-0.54**	**-2.09**
PSPTO_1934	*PSYR_3480*	flagellar basal body rod protein FlgC	**-0.71**	**-2.18**
PSPTO_1935	*PSYR_3479*	basal-body rod modification protein FlgD	**-1.17**	**-2.42**
PSPTO_1936	*PSYR_3478*	flagellar hook protein FlgE	**-2.36**	**-3.39**
PSPTO_1943	*PSYR_3472*	peptidoglycan hydrolase FlgJ	**-0.34**	**-1.61**
PSPTO_1944	*PSYR_3471*	flagellar hook-associated FlgK	**-1.11**	**-0.88**
PSPTO_1945	*PSYR_3470*	flagellar hook-associated protein FlgL	**-2.07**	**-3.26**
PSPTO_1949	*PSYR_3466*	*fliC*, flagellin	**-2.32**	**-2.14**
PSPTO_1950	*PSYR_3465*	flagellin FlaG	**-2.52**	**-2.98**
PSPTO_1951	*PSYR_3464*	flagellar hook-associated FliD	**-1.4**	**-2.84**
PSPTO_1960	*PSYR_3455*	flagellar assembly protein H	**-0.18**	**-1.33**
PSPTO_1962	*PSYR_3453*	flagellar biosynthesis chaperone	**-0.55**	**-2.04**
PSPTO_1966	*PSYR_3449*	flagellar hook-length control FliK	**-0.15**	**-1**
PSPTO_1974	*PSYR_3442*	flagellar biosynthetic protein FliR	**-0.31**	**-1.57**
PSPTO_1975	*PSYR_3441*	flagellar biosynthetic protein FlhB	**-0.89**	**-1.74**
PSPTO_1984	*PSYR_3432*	flagellar motor protein	**-0.94**	**-2.25**
**Cell division**				
PSPTO_4403	*PSYR_4097*	cell division protein FtsZ	**-2.33**	**-3.01**
PSPTO_4404	*PSYR_4098*	cell division protein FtsA	**-0.98**	**-1.97**
PSPTO_4405	*PSYR_4099*	cell division protein FtsQ	**-1.08**	**-1.83**
PSPTO_3511	*PSYR_3284*	cell division inhibitor	**1.75**	**1.44**
PSPTO_3872	*PSYR_1613*	septum site-determining MinC	**1.03**	**0.66**
PSPTO_3873	*PSYR_1612*	septum site-determining MinD	**1.18**	**1.08**
PSPTO_3874	*PSYR_1611*	cell division topological specificity factor MinE	**1.06**	**0.79**
**Exopolysaccharides(EPS)**				
PSPTO_1232	*PSYR_1052*	alginate biosynthesis protein AlgA	**-5.04**	**-3.25**
PSPTO_1233	*PSYR_1053*	alginate biosynthesis protein AlgF	**-4.74**	**-3.29**
PSPTO_1234	*PSYR_1054*	alginate biosynthesis protein AlgJ	**-4.56**	**-2.24**
PSPTO_1235	*PSYR_1055*	alginate biosynthesis protein AlgI	**-4.1**	**-2.07**
PSPTO_1237	*PSYR_1057*	alginate biosynthesis protein AlgX	**-3.65**	**-0.99**
PSPTO_1238	*PSYR_1058*	alginate biosynthesis protein AlgG	**-3.61**	**-2.15**
PSPTO_1239	*PSYR_1059*	alginate biosynthesis protein AlgE	**-3.49**	**-1.59**
PSPTO_1240	*PSYR_1060*	alginate biosynthesis protein AlgK	**-3.36**	**-1.56**
PSPTO_1241	*PSYR_1061*	alginate biosynthesis protein Alg44	**-2.72**	**-0.89**
PSPTO_1242	*PSYR_1062*	alginate biosynthesis protein Alg8	**-1.99**	**-0.4**

DEGs were differentially expressed genes in the (p)ppGpp^0$_{Pst\mathrm{DC3000}}$ and (p)ppGpp^0$_{Pss\mathrm{B728a}}$ with p-value <0.05 between the WT and the (p)ppGpp^0 mutants.

Table S10. List of differentially expressed genes (DEGs) related to DNA replication, RNA processes and ribosomal protein biosynthesis

Locus tag		Description	(p)ppGpp[0] $_{Pst\text{DC3000}}$ /*Pst*DC3000	(p)ppGpp[0] $_{Pss\text{B728a}}$ /*Pss*B728a
**DC3000**	**B728A**			
**DNA replication**				
PSPTO_0113	*PSYR_0075*	ATP-dependent DNA helicase Rep	**1.59**	**1.13**
PSPTO_0656	*PSYR_4518*	single-stranded DNA-binding protein	**0.94**	**1.07**
PSPTO_1488	*PSYR_1298*	single-stranded-DNA-specific exonuclease RecJ	**1.13**	**0.97**
PSPTO_2478	*PSYR_2244*	DNA topoisomerase III TopB	**1.62**	**1.55**
PSPTO_3711	*PSYR_1764*	DNA polymerase III subunit epsilon DnaQ	**1.66**	**1.65**
PSPTO_3712	*PSYR_1763*	ribonuclease HI RnhA	**1.62**	**1.42**
PSPTO_4120	*PSYR_3856*	DinG family ATP-dependent helicase	**2.16**	**1.66**
PSPTO_4236	*PSYR_3970*	uracil-DNA glycosylase Ung	**1.24**	**1.03**
PSPTO_5516	*PSYR_5065*	DNA helicase II UvrD	**1.26**	**1.25**
**RNA processes**				
PSPTO_1587	*PSYR_3791*	ATP-dependent RNA helicase SrmB	**1.86**	**1.84**
PSPTO_1775	*PSYR_3619*	ATP-dependent RNA helicase, DEAD box family	**2.98**	**1.92**
PSPTO_4664	*PSYR_4297*	ATP-dependent RNA helicase RhlE	**2.01**	**2**
PSPTO_5007	*PSYR_0516*	ATP-independent RNA helicase DbpA	**2.15**	**1.83**
PSPTO_5070	*PSYR_0458*	ATP-dependent RNA helicase RhlE	**1.95**	**1.99**
**Ribosome proteins**				
PSPTO_0539	*PSYR_4639*	30S ribosomal protein S21	**1.87**	**1.04**
PSPTO_0625	*PSYR_4549*	30S ribosomal protein S10	**1.73**	**1.21**
PSPTO_0640	*PSYR_4534*	30S ribosomal protein S8	**1.62**	**0.71**
PSPTO_0643	*PSYR_4531*	30S ribosomal protein S5	**1.32**	**0.19**
PSPTO_0650	*PSYR_4525*	30S ribosomal protein S4	**1.18**	**0.18**
PSPTO_0802	*PSYR_0707*	30S ribosomal protein S20	**2.47**	**1.38**
PSPTO_1473	*PSYR_1282*	30S ribosomal protein S16	**1.76**	**0.72**
PSPTO_1534	*PSYR_1343*	30S ribosomal protein S2	**1.68**	**2.07**
PSPTO_1750	*PSYR_3642*	30S ribosomal protein S1	**1.06**	**0.78**
PSPTO_4425	*PSYR_4119*	30S ribosomal protein S9	**2.31**	**1.45**
PSPTO_4932	*PSYR_0582*	30S ribosomal protein S18	**1.61**	**0.95**
PSPTO_4933	*PSYR_0581*	30S ribosomal protein S6	**1.33**	**0.97**
PSPTO_0090	*PSYR_0225*	50S ribosomal protein L33	**2.71**	**2.14**
PSPTO_0615	*PSYR_4559*	50S ribosomal protein L11	**1.49**	**1.25**
PSPTO_0616	*PSYR_4558*	50S ribosomal protein L1	**1.66**	**1.4**
PSPTO_0617	*PSYR_4557*	50S ribosomal protein L10	**1.49**	**0.53**
PSPTO_0618	*PSYR_4556*	50S ribosomal protein L7/L12	**1.84**	**0.59**
PSPTO_0626	*PSYR_4548*	50S ribosomal protein L3	**1.28**	**1.12**
PSPTO_0627	*PSYR_4547*	50S ribosomal protein L4	**1.08**	**0.93**

PSPTO_0629	*PSYR_4545*	50S ribosomal protein L2	**1.11**	**0.67**
PSPTO_0641	*PSYR_4533*	50S ribosomal protein L6	**1.36**	**0.48**
PSPTO_0642	*PSYR_4532*	50S ribosomal protein L18	**1.19**	**0.4**
PSPTO_0644	*PSYR_4530*	50S ribosomal protein L30	**1.09**	**0.13**
PSPTO_0652	*PSYR_4523*	50S ribosomal protein L17	**1.24**	**0.32**
PSPTO_0797	*PSYR_0701*	50S ribosomal protein L21	**1.4**	**1.66**
PSPTO_1476	*PSYR_1285*	50S ribosomal protein L19	**1.97**	**1.3**
PSPTO_2380	*PSYR_2164*	50S ribosomal protein L35	**0.24**	**1.63**
PSPTO_2381	*PSYR_2165*	50S ribosomal protein L20	**0.72**	**1.66**
PSPTO_3835	*PSYR_1644*	50S ribosomal protein L32	**2.14**	**1.16**
PSPTO_4426	*PSYR_4120*	50S ribosomal protein L13	**2.14**	**1.3**
PSPTO_4930	*PSYR_0584*	50S ribosomal protein L9	**1.94**	**1.02**
PSPTO_5136	*PSYR_0399*	50S ribosomal protein L31	**3.27**	**2.17**
PSPTO_5615	*PSYR_5137*	50S ribosomal protein L34	**2.56**	**1.08**

DEGs were differentially expressed genes in the (p)ppGpp$^{0}_{Pst\mathrm{DC3000}}$ and (p)ppGpp$^{0}_{Pss\mathrm{B728a}}$ with p-value <0.05 between the WT and the (p)ppGpp^{0} mutants.
