# Supplementary material for: Comparative transcriptomic analysis of global gene expression mediated by (p) ppGpp reveals common regulatory networks in Pseudomonas syringae
Source: BMC Genomics. 2020 Apr 10;21:296. doi: 10.1186/s12864-020-6701-2 (PMC7146990; doi:10.1186/s12864-020-6701-2)
Supplement: Supplementary file 6 — Additional file 6: Table S14. Primers for qRT-PCR used in this study. [file 12864_2020_6701_MOESM6_ESM.pdf]

Table S14. Primers for qRT-PCR used in this study

Primer	Sequences (5' to 3')
gltB$_{DC3000}$-rt1	**CCAAGGCCTATGCCGACCTG**
gltB$_{DC3000}$-rt2	**GATCACGTCGAGGCCGGTTT**
desA$_{DC3000}$-rt1	**CACGCCAAATGCGAAACCGT**
desA$_{DC3000}$-rt2	**GCGATACAGCTCGGCACCTT**
pspto1775$_{DC3000}$-rt1	**AGAGCCTTCGGCCATTCAGC**
pspto1775$_{DC3000}$-rt2	**CGGATCGATGCGATGCAGGA**
infA$_{DC3000}$-rt1	**CACCCTGCCCAACACCATGT**
infA$_{DC3000}$-rt2	**AACGAGCGCGGTAGGTGATG**
avrE1$_{DC3000}$-rt1	**CTGCCACGCCGCCCACCGCG**
avrE1$_{DC3000}$-rt2	**GGGGCTGCCTCCGGGTGTAT**
iaaL$_{DC3000}$-rt1	**TGCGCCAACCTGTTCACCAT**
iaaL$_{DC3000}$-rt2	**CACATGCGCACCGCAGTTAC**
*csrA*2$_{DC3000}$-rt1	**CCTGATCATTGGTGATGGG**
*csrA*2$_{DC3000}$-rt2	**ATGGCTTGGTTCTTCGTC**
gltB$_{PssB728a}$-rt1	**CGAAGTGCTGGGCGATCTGT**
gltB$_{PssB728a}$-rt2	**GGTAGGACGCAACGGTCGAG**
cstA$_{PssB728a}$-rt1	**GGGCGTGTACTTCGCCATGA**
cstA$_{PssB728a}$-rt2	**TTTCGCCACAGCCGTCAGTT**
actP$_{PssB728a}$-rt1	**ACGCAACCGGCTTTATCGGT**
actP$_{PssB728a}$-rt2	**AGCGACTGCCGAGATGAAGC**
acsA$_{PssB728a}$-rt1	**AACACGGTCGGCAAGCAGAA**
acsA$_{PssB728a}$-rt2	**GTTGCCCAGGTTGTCCACCA**
mtiD$_{PssB728a}$-rt1	**ACTCAGCGCTTGTTGGCAGT**
mtiD$_{PssB728a}$-rt2	**CAGTGTGCCACTCACACCCA**
cspC$_{PssB728a}$-rt1	**GGCTGAACGTCAGAGCGGTA**
cspC$_{PssB728a}$-rt2	**TCGTCAGCCTGCATGCCTTT**
ksgA$_{PssB728a}$-rt1	**GCGCCTCGTCCCATACGAAA**
ksgA$_{PssB728a}$-rt2	**CGCTGGTTGAACGCTTCACG**
